# An evaluation of synthetic data augmentation for mitigating covariate bias in health data

**DOI:** 10.1016/j.patter.2024.100946

**Published:** 2024-02-29

**Authors:** Lamin Juwara, Alaa El-Hussuna, Khaled El Emam

**Affiliations:** 1 School of Epidemiology and Public Health, University of Ottawa, Ottawa, ON, Canada; 2 Research Institute, Children’s Hospital of Eastern Ontario, Ottawa, ON, Canada; 3 Open Source Research Collaboration, Aalborg, Denmark; 4 Data Science, Replica Analytics Ltd., Ottawa, ON, Canada

**Keywords:** covariate imbalance, data bias, classification, synthetic data generation, fairness, generative model

## Abstract

Data bias is a major concern in biomedical research, especially when evaluating large-scale observational datasets. It leads to imprecise predictions and inconsistent estimates in standard regression models. We compare the performance of commonly used bias-mitigating approaches (resampling, algorithmic, and post hoc approaches) against a synthetic data-augmentation method that utilizes sequential boosted decision trees to synthesize under-represented groups. The approach is called synthetic minority augmentation (SMA). Through simulations and analysis of real health datasets on a logistic regression workload, the approaches are evaluated across various bias scenarios (types and severity levels). Performance was assessed based on area under the curve, calibration (Brier score), precision of parameter estimates, confidence interval overlap, and fairness. Overall, SMA produces the closest results to the ground truth in low to medium bias (50% or less missing proportion). In high bias (80% or more missing proportion), the advantage of SMA is not obvious, with no specific method consistently outperforming others.

## Highlights

• The study compares the efficacy of bias-mitigating approaches in real-world health data
• The synthetic data-augmentation method synthesizes the under-represented group
• Performance is evaluated based on predictive accuracy, parameter precision, and fairness
• Our method improves the performance and fairness in low to medium bias severity

## The bigger picture

Data bias occurs in the sampling processes of many real-world datasets. For health data, gender and ethnicity are common factors causing sampling bias. Models built on datasets with under-represented categories will lead to inaccurate predictions and unfair decisions. To avoid such issues, bias-mitigation methods could be used to handle biased data. Techniques for generating synthetic individuals in the minority groups could augment the biased datasets and reconstruct full and unbiased datasets. If the group distributions in the samples are rebalanced, the results of the models will be generalizable to the original population.

## Graphical abstract

**Figure:**
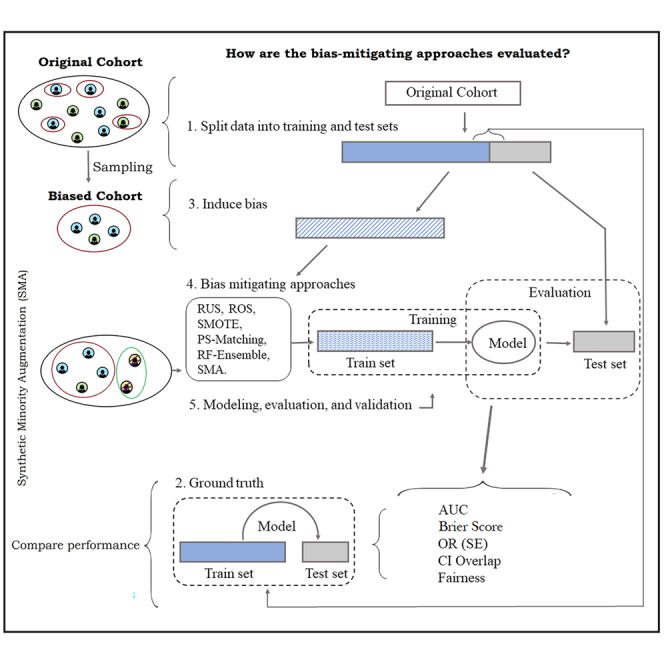

## Teaser

The paper evaluates different methods for mitigating bias in real-world health data. Multiple bias-mitigation approaches are compared, and one based on synthetic data generation is found to address the effects of data bias for low to medium bias severity. This approach simulates additional patients from the under-represented group to augment the data and reduce the under-representation. The study’s outcomes provide a method to reduce the effects of data bias on statistical model quality, accuracy, and fairness.

## Introduction

There is a wide range of biases that can affect datasets,^1^ with data bias being notably common in biomedical research.^2^^,^^3^^,^^4^^,^^5^ In the statistical learning literature, a training sample is said to be biased if its covariate distributions differ from those of the original population.^6^^,^^7^ This results in the under-representation of specific covariate categories or distributions (as opposed to outcome imbalance, which constitutes a different problem). Unlike in a randomized study where participants are assigned to treatment groups to ensure a balanced dataset,^8^^,^^9^ observational datasets are prone to covariate biases.^10^^,^^11^^,^^12^

Covariate bias often stems from selection bias and is introduced at the data-collection or analysis stage, where some participants are systematically excluded from the analytical dataset.^13^ Among the numerous selection-type biases, sampling bias is the most prominent and is sometimes used interchangeably with selection bias in the literature.^14^ Sampling bias results in a sampled dataset that does not reflect the study population and, therefore, models built on such data are difficult to generalize.^15^ While sampling biases are most often attributed to gender and ethnic factors, they can also result from the lack of capture and inclusion of social determinants of health, including geographical and socioeconomic conditions, such as age and place of birth, which drive outcome categorization.^16^ Henceforth in this article, the presence of sample selection bias in the sampled cohorts is referred to as “data bias.”

Data bias is encountered in many real-world applications, including medical diagnosis,^3^ image or facial recognition,^17^^,^^18^ text classification,^19^ and speech recognition.^20^^,^^21^ It can manifest in various analytical settings such as comparative, predictive, and diagnostic scenarios^11^ where it presents substantial moral and ethical implications.^22^ Some examples include algorithms for popular online recommender apps built on datasets in which female participants were under-represented, which in turn gave wrong recommendations to female users.^23^ In a recent study, researchers showed that a widely used algorithm (to allocate health care to patients) in United States (US) hospitals was less likely to refer black people than white people who were equally sick to programs that aim to improve care for patients with complex medical needs.^24^ It was found that bias was introduced in the training set during data collection when patients who self-identified as black were on average assigned lower pain scores than other groups. Many other examples of gender- and race-induced biases in covariate distributions have been highlighted in the literature.^3^^,^^4^ These biases might sometimes lead to algorithmic bias, which has received a lot of attention in recent years, especially in the context of personalized recommender systems.^25^^,^^26^ Notable examples include racial biases in algorithms used in US court systems to predict the likelihood of repeated offenses^27^ and gender biases in Amazon’s hiring software.^28^

In trained models, data bias not only affects the effect estimates of the predictor-outcome association or makes true associations statistically non-significant,^29^ it also reduces the sample precision and, as a result, the prediction accuracy.^30^^,^^31^ Thus, results obtained from models built on biased samples are typically not generalizable to the original population.^16^^,^^32^^,^^33^^,^^34^ Another challenge with analyzing datasets with data bias is the degraded performance on standard performance metrics.^35^ For example, biased samples can be misleading in that they can sometimes produce higher predictive accuracy results than the ground truth.^36^

In recent years, several approaches have been proposed for mitigating the impact of data bias. These approaches cover biases in fields such as healthcare data from clinician interactions, hiring and promotion,^37^ imaging,^38^ text classification,^39^ and transportation,^40^ among others. In particular, rebalancing schemes such as random undersampling (RUS) and random oversampling (ROS) are quite attractive, as they allow researchers to reconstruct balanced datasets a priori before any modeling effort. Although such traditional schemes have been shown to perform well in many settings, they are not always effective, especially when the source of bias is complex or the bias severity is too high. Various data-augmentation methods have also been proposed to mitigate biases in image recognition,^41^ speech,^42^ and vision.^43^ However, they are often model specific, requiring large training cohorts, and might even give a false sense of sampling unseen data from the same underlying distribution as the original training data.^44^

In this paper, we compare a representative set of bias-mitigation approaches for structured tabular health data and propose and evaluate an approach based on synthetic data generation. The contributions of this work are 3-fold.

(1) Evaluate various data bias-mitigation approaches that can be used to correct for covariate bias in health datasets when applied to binary classification tasks.
(2) Describe a bias-mitigation method based on synthetic data-generation techniques to generate synthetic copies of the under-represented category to augment the biased dataset and reconstruct full and unbiased data samples.
(3) Compare the statistical model performance and fairness of the proposed method to the traditional approaches through simulations and applications to four real datasets.

## Background

### Terminology

For biased datasets with a categorical covariate that is unevenly distributed, we will refer to that covariate as the biased covariate. The under-represented category of the biased covariate is termed the minority group, and the other categories are the majority group. The degree of imbalance is determined by the ratio of the minority to the majority group. The extent of bias is with reference to the baseline distribution, which may be the population distribution or a proxy for it. For example, the biased covariate can be “sex,” with “female” being the minority group, “male and other” being the majority group, and the baseline distribution in the population being 50% female.

### Current bias-mitigation methods

The current methods for mitigating data bias can broadly be classified into three categories: rebalancing approaches, algorithmic approaches, and post-processing approaches. We present a brief description of some of the approaches below and refer the reader to Appendix A in supplemental experimental procedures for a more detailed review of the ones that we apply in our study.

Rebalancing approaches attempt to construct datasets that are reflective of the true population. Starting with the biased data, the goal of rebalancing is to produce a balanced dataset that provides a good approximation of the underlying population data. These approaches can take the form of subsampling (e.g., RUS, ROS, and SMOTE [synthetic minority oversampling technique]) or matching (e.g., propensity score [PS] matching).

Among the subsampling approaches, RUS, ROS, and SMOTE have gained a lot of traction in recent years. RUS is a data pre-processing approach to rebalance unevenly distributed groups in imbalanced datasets by randomly removing excess observations in the majority group of the biased covariate.^45^ In the current context, the approach is adopted for rebalancing the group distributions in categorical covariates in biased datasets.^3^ ROS, in contrast, attempts to rebalance biased covariate distributions by repeating the entries of randomly sampled observations of the minority group. The resulting cohort is a larger dataset that is composed of repeated observations and well-represented covariate distributions.^46^ While ROS has the advantage of improving the stability and performance of learning algorithms applied to the dataset by mitigating convergence issues, it is often argued that the duplicated observations contribute minimally to the dataset’s richness or diversity, potentially leading to overfitting.^47^ An alternative approach to replicating instances of the minority group is to generate synthetic copies through interpolation among neighboring minority instances. This approach is termed SMOTE.^48^ Unlike ROS, the main appeal of SMOTE is that it adds new plausible observations that are sampled from the neighborhood of the minority group and not simply repeated entries.

Another rebalancing approach that takes place at the modeling stage is the use of matching. PS adjustment can take place in the form of matching, stratification, or regression (covariance) adjustment. While matching and stratification are usually applied to the dataset before statistical modeling to construct balance and appropriate comparisons among the baseline covariates, adjustment with PSs is employed at the analyses stage by including the scores (as weights) directly into the regression model. In this article, we restrict our evaluation to PS adjustment to rebalance the biased data at the analysis stage.

Algorithmic methods mitigate the problem of covariate imbalance by targeting the learning stage of the analyses. The category encompasses approaches that adapt commonly used learning algorithms to reweight models for learning from the minority group of the biased data. These approaches can broadly be grouped into ensemble-based learning,^49^ cost-sensitive learning,^50^ and single-class learning.^51^

In this article, we use the random forest (RF) ensemble approach presented by Dong et al.^52^ to handle biased data. Their approach incorporates modifications to the RF algorithm, enabling it to draw inferences from highly biased datasets. For example, the model initially takes bootstrap samples from the minority group, followed by an equal number of samples from the majority group, thus balancing the groups. The technique is highly regarded for its capacity to merge the advantages of multiple learners, enhancing overall performance. Additionally, RF ensembles have demonstrated their effectiveness in addressing biased data due to their innate ability to balance group distributions and reduce overfitting.

When the effect of data bias cannot be addressed during pre-processing (e.g., data cleaning) or the modeling stage, it is sometimes possible to employ post hoc adjustment techniques to account for the effect of learning from biased data. For example, in genetic studies of gene-based rare variant associations, bootstrap resampling approaches are often employed to adjust for bias in single-marker tests on common variants in genome-wide association studies.^53^ In most cases, post hoc adjustments are only recommended as last-resort options when bias could not be mitigated in the early stages of the study.^54^

## Results

This section presents the performance results for the full model and the stratified model across the biased datasets and the bias-mitigated datasets.

### Simulation results

In Table 2, we show the simulation results averaged across the 500 iterations. These are shown for some of the bias proportions only to illustrate trends, and the full results are given in Appendix B in supplemental experimental procedures.

**Table 1:** Summary of the real datasets that were used

Dataset		Description	Outcome	Biased covariate	Conditioning covariate
1	Canadian Community Health Survey (CCHS)	63,522 observations	cardiovascular health	gender (female = 45.2%)	CBI: new immigrants
–	–	8 variables	–	–	CBII: marital status
2	N0147 colon cancer	1,543 observations	death	bowel obstruction (No = 83.8%)	CBI: gender
–	–	10 variables	–	–	CBII: BMI
3	Danish Colon Cancer data (DCCG)	12,855 observations	postoperative complications	gender (female = 55.9%)	CBI: P-PN stage
–	–	192 variables (total)	–	–	CBII: ASA
–	–	9 selected	–	–	–
4	Breast cancer	277 observations	BC class	age (20–49 = 45.5%)	CBI: left/right breast
–	–	10 variables	–	–	CBII: menopause

CBI, conditional bias I; CBII, conditional bias II.

**Table 2:** Simulation results across 500 iterations for the full model

Proportion	Approach	Marginal bias			Conditional bias I			Conditional bias II		
		AUC	OR_*Z*_ (SD)	*I*_*Z*_	AUC	OR_*Z*_ (SD)	*I*_*Z*_	AUC	OR_*Z*_ (SD)	*I*_*Z*_
15%	Biased data	0.72	1.30 (0.15)	–	0.72	1.30 (0.16)	–	0.72	1.30 (0.15)	–
	RUS	0.72	1.28 (0.15)	0.98	0.72	1.28 (0.16)	0.94	0.71	1.28 (0.15)	0.95
	ROS	0.72	1.27 (0.14)	0.98	0.72	1.29 (0.14)	0.93	0.72	1.27 (0.14)	0.94
	SMOTE	0.71	1.14 (1.18)	0.74	0.69	1.19 (1.18)	0.88	0.70	1.18 (1.04)	0.84
	PS match	0.72	1.19 (0.16)	0.92	0.72	1.18 (0.16)	0.92	0.72	1.23 (0.16)	0.96
	RF ensemble	0.70	–	–	0.69	–	–	0.69	–	–
	SMA^a^	0.72	1.25 (0.14)	0.98	0.72	1.27 (0.14)	0.95	0.72	1.26 (0.14)	0.95
50%	Biased data	0.71	1.49 (0.19)	–	0.72	1.31 (0.15)	–	0.72	1.43 (0.19)	–
	RUS	0.70	1.38 (0.14)	0.85	0.72	1.30 (0.14)	0.89	0.71	1.41 (0.20)	0.84
	ROS	0.71	1.45 (0.16)	0.78	0.72	1.29 (0.16)	0.90	0.71	1.51 (0.19)	0.82
	SMOTE	0.68	0.94 (0.21)	0.71	0.69	1.02 (0.21)	0.49	0.68	0.91 (0.18)	0.41
	PS match	0.70	1.44 (0.26)	0.72	0.71	1.32 (0.13)	0.95	0.70	1.45 (0.24)	0.78
	RF ensemble	0.69	–	–	0.68	–	–	0.70	–	–
	SMA^a^	0.71	1.24 (0.19)	0.93	0.71	1.29 (0.13)	0.90	0.72	1.39 (0.14)	0.88
80%	Biased data	0.68	1.00 (0.25)	–	0.71	1.32 (0.19)	–	0.70	1.09 (0.22)	–
	RUS	0.68	1.17 (0.28)	0.97	0.71	1.41 (0.21)	0.72	0.70	1.03 (0.27)	0.76
	ROS	0.68	1.02 (0.24)	0.98	0.72	1.28 (0.24)	0.75	0.70	0.97 (0.23)	0.84
	SMOTE	0.67	0.73 (0.21)	0.34	0.69	1.05 (0.20)	0.60	0.70	0.90 (1.15)	0.33
	PS match	0.65	0.50 (0.95)	0.84	0.70	1.54 (0.15)	0.81	0.69	1.08 (0.40)	0.66
	RF ensemble	0.66	–	–	0.68	–	–	0.69	–	–
	SMA^a^	0.69	0.96 (0.51)	0.98	0.70	1.33 (0.24)	0.77	0.70	1.24 (0.16)	0.91

Estimates of the mean AUC, mean odds ratio associated with *Z* and standard deviation (OR_*Z*_ (SD)), and the confidence interval overlap of the biased covariate effect with the ground truth (I_*Z*_) from 500 repetitions. Proportion indicates the proportion of observations removed under each bias setting. For the original data: AUC = 0.72; OR_*Z*_ (SD) = 1.25 (0.15); *I*_*Z*_ = 1.00. Only results for missing proportions of 15%, 50%, and 80% are shown.

a Estimates are averaged from *m* = 100 synthetic copies.

#### Impact of bias

**Figure 1:**
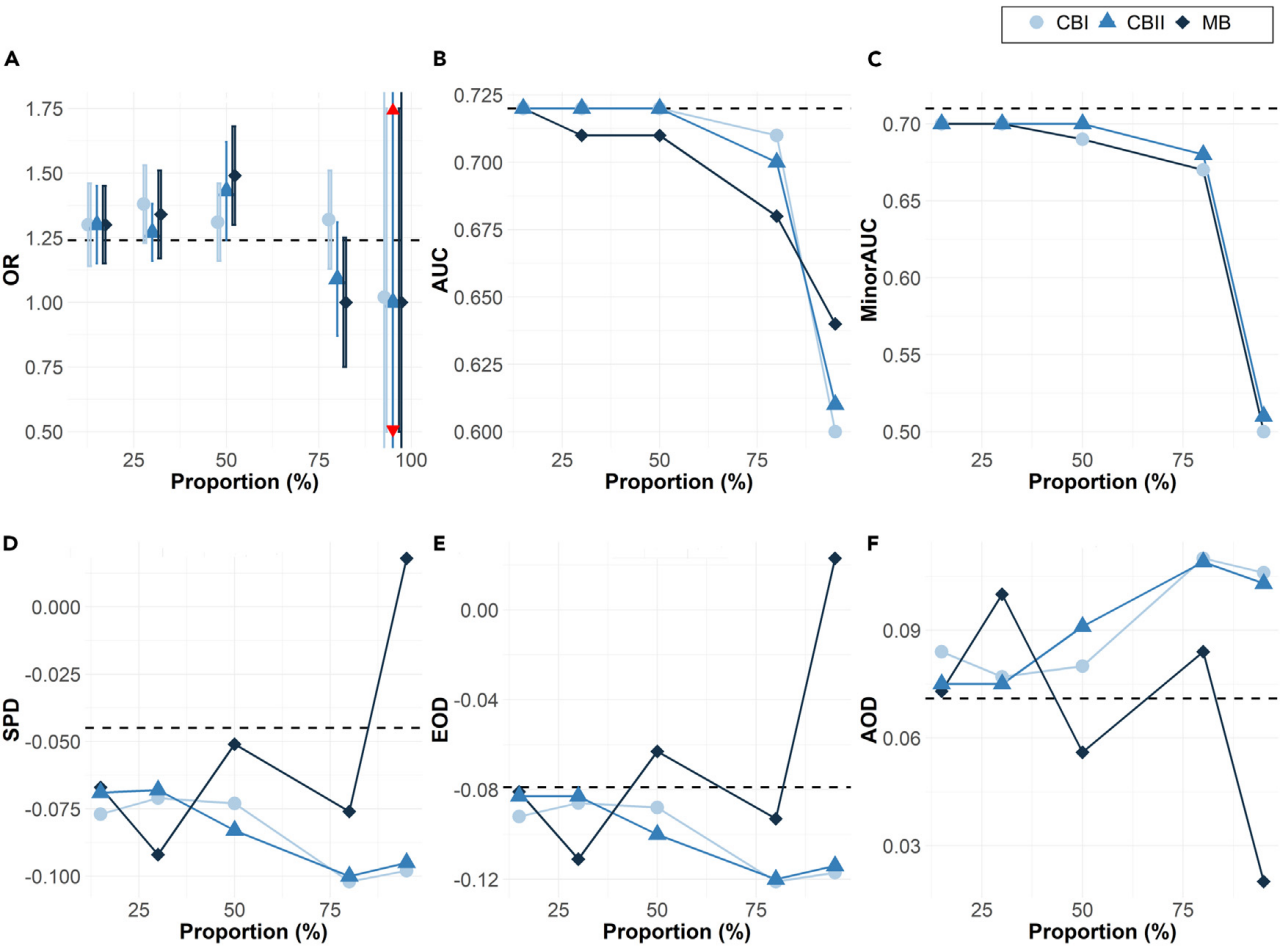
Bias type Simulations results across 500 iterations for the full model showing the impact of bias (increasing missing proportion) on (A) odds ratio (OR) and the standard deviation of the biasing covariate across simulation runs, (B) the overall area under the curve (AUC), (C) minority-group AUC, and fairness metrics: (D) statistical parity difference (SPD), (E) equal opportunity difference (EOD), and (F) average odds difference (AOD). The dashed line represents the ground truth from the original data.

As the proportion of observations excluded is increased from 15% to 95% (Figure 1), the discrepancy between the biased covariate parameter estimates on the biased samples and that of the ground truth also increases.

Relative to the ground-truth odds ratio of the biasing predictor *Z* (OR_*Z*_ = 1.25), the OR_*Z*_ estimates of the biased samples increase from 1.30 to 1.49 in the low to moderate bias setting (missing proportion of 50% or less) before rapidly decreasing toward the null (OR_*Z*_ = 1) in high to extreme bias setting (missing proportion of 80% or more).

The areas under the curve (AUCs) of the biased datasets decrease consistently from the ground truth with increasing missing proportions under marginal bias and decline rapidly for all types of high or extreme bias (Figure 1B). Data bias is more impactful on the performance of the category affected by the missing observations. As more observations are excluded from the minority group, the impact on minority AUC also grows (Figure 1C).

As the extent of bias grows, the three types of fairness metrics increasingly deviate from the ground-truth value, with the greatest deviation at the highest bias (Figures 1D–1F).

#### Performance of SMA relative to ground truth

**Figure 2:**
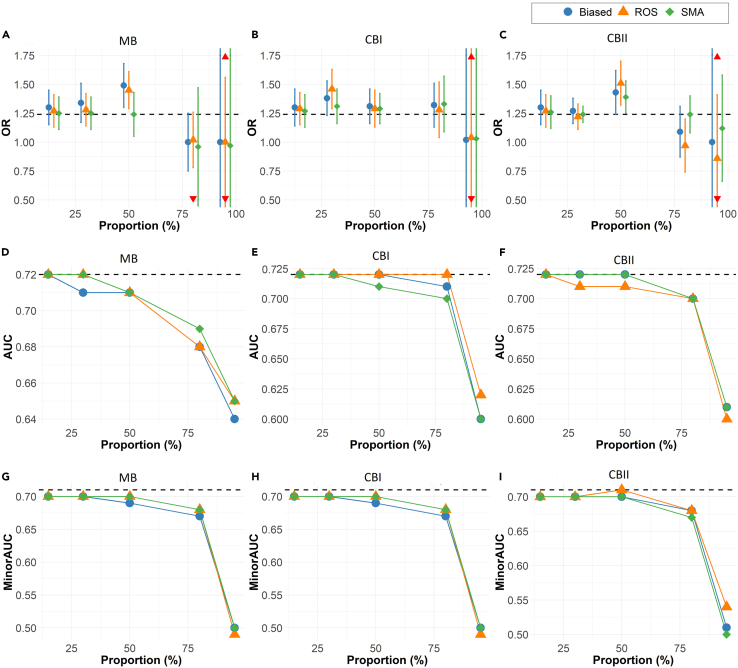
Bias mitigation approach Simulations results across 500 iterations for the full model showing the comparisons of the OR estimates and the standard deviation of the biasing covariate across simulation runs (A–C), overall model AUCs (D–F), and minority-group AUC of the biasing covariate (G–I) for synthetic minority augmentation (SMA), random oversampling (ROS, the best-performing alternative), and biased data. The dashed line represents the ground truth from unbiased data.

The performance of synthetic minority augmentation (SMA) is shown relative to ROS in Figure 2, as this was the best-performing alternative bias-mitigation approach in the simulations.

Unlike alternative approaches (RUS, ROS, SMOTE, RF ensembles, and PS matching), SMA produces OR estimates that are consistently comparable to the ground truth in low to moderate bias scenarios. As the severity of bias increases, SMA continues to offer OR estimates that are more comparable to the ground truth than the other approaches. For high to extreme bias, the results are not always conclusive (Figures 2A–2C).

The parameter estimates corresponding to *β*_*Z*_= (log(0.5),log(1.01),log(2)) and AUCs are presented in Tables S7 and S8 to show the robustness of the approaches under negative associations, approximately null associations, and large positive associations, respectively. In all cases, the results are consistent with the primary analysis results.

Up to moderate bias, SMA has AUC closer to the ground truth than ROS. At higher levels of bias, the results on the best approach for AUC are not conclusive (Figures 2D–2F).

The minority-group AUC results in Figure 2 demonstrate that SMA consistently produces values comparable to the ground truth in low to moderate bias. In cases of extreme data bias (missing proportion of 95% or more), no method can ensure the best accuracy. Compared to other methods, SMA performs to the same degree or better on the Brier scores of the minority group (Table S5).

The confidence interval overlap for SMA also consistently outperforms other approaches, especially in low to moderate bias samples. These results are shown in Table 2.

**Figure 3:**
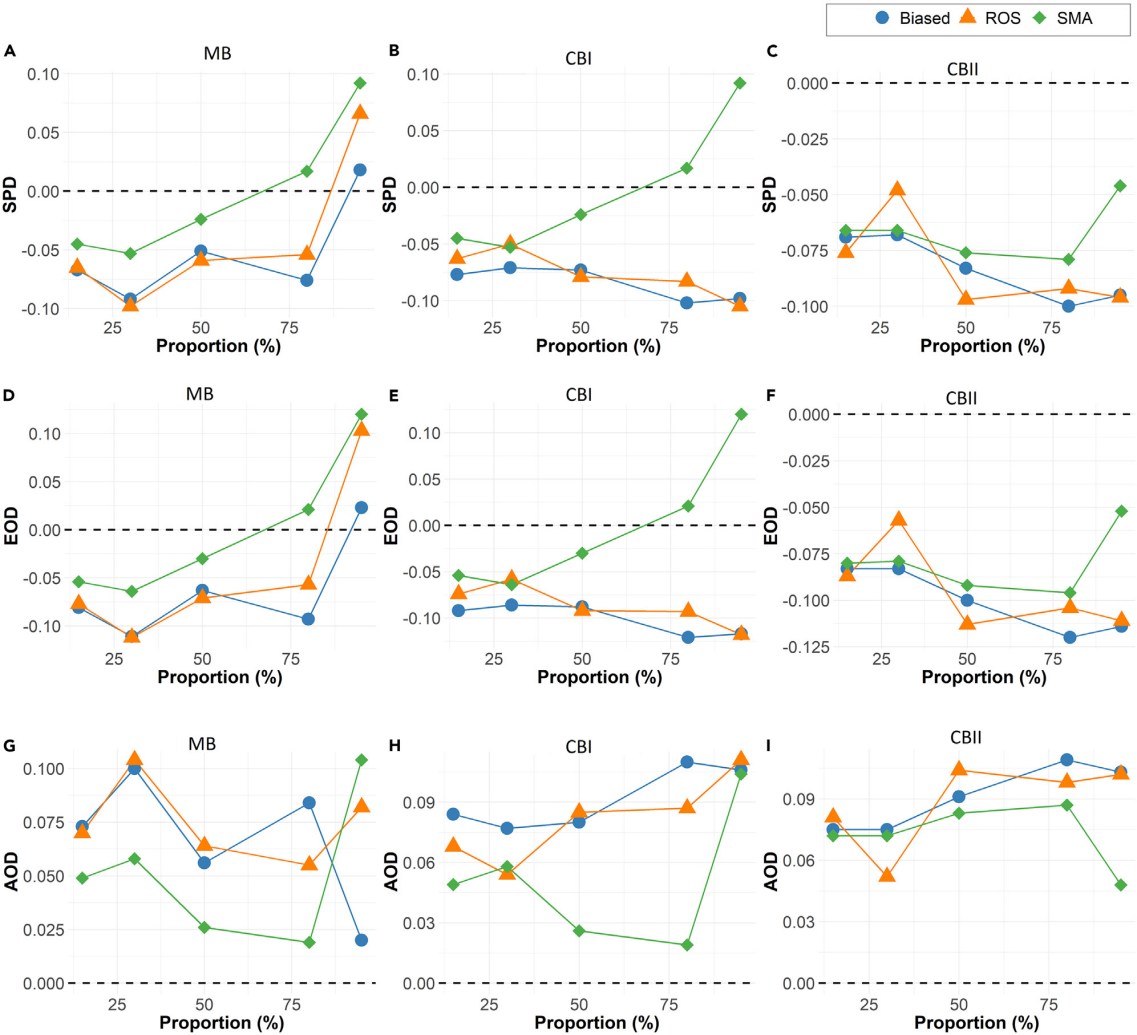
Bias mitigation approach Simulation results across 500 iterations for the full model showing the fairness estimates based on (A–C) statistical parity difference (SPD), (D–F) equal opportunity difference (EOD), and (G–I) average odds difference (AOD). The estimates are reported for biased data, ROS (the best-performing alternative), and SMA. The dashed line represents the optimal fairness value under no data bias.

In Figure 3, the fairness measures (statistical parity difference [SPA], equal opportunity difference [EOD], and average odds difference [AOD]) indicate that SMA has fairness that is generally closer to the ground truth in comparison to other methodologies. Also, SMA tends to have better fairness than the ground truth when it does deviate from it.

In summary, the simulations demonstrate that for low to medium bias SMA yields datasets with the best parameter estimation results (more precise effect estimates, standard errors closer to the ground truth, and larger confidence interval overlap). In terms of predictive modeling (AUC and minority-group AUC estimates), SMA had values consistently close to the ground truth and comparable to the best competitive alternatives. Furthermore, the fairness-related simulation findings indicate that SMA typically generates models exhibiting the most favorable fairness metrics.

#### Performance of SMA relative to biased data

As seen in Figures 2 and 3, the SMA method is an improvement over the biased dataset across all metrics and all types of bias. The OR values are closer to the ground truth with the application of SMA, as are the AUC and the minority AUC. The fairness metrics are also consistently closer to the ground truth than the biased dataset.

### Results for real datasets

The results of the approaches evaluated on the Canadian Community Health Survey (CCHS) data are presented in Table 3. These are shown for selected bias proportions only to illustrate trends, while the full results are given in Appendix D in supplemental experimental procedures. The results for the remaining datasets, which show similar patterns, are also presented in the same appendix.

**Table 3:** Estimates of mean AUC, odds ratio of *Z* (OR_*Z*_ (SE)), and interval overlaps (*I*_*Z*_) of the biased covariate *Z* for each bias-mitigating approach on the CCHS data

CCHS data proportion	Approach	Marginal bias			Conditional bias I			Conditional bias II			
		AUC	OR_*Z*_ (SE)	*I*_*Z*_	AUC	OR_*Z*_ (SE)	*I*_*Z*_	AUC	OR_*Z*_ (SE)	*I*_*Z*_	
15%	Biased data	0.70	1.57 (−)	–	0.70	1.57 (−)	–	0.70	1.58 (−)	–	
	RUS	0.70	1.58 (0.03)	0.94	0.70	1.58 (0.03)	0.94	0.70	1.57 (0.02)	0.97	
	ROS	0.70	1.57 (0.02)	0.97	0.70	1.57 (0.02)	0.95	0.70	1.58 (0.02)	0.97	
	SMOTE	0.70	1.52 (0.04)	0.77	0.70	1.56 (0.04)	0.76	0.70	1.57 (0.04)	0.78	
	PS matching	0.69	1.50 (0.03)	0.56	0.69	1.50 (0.03)	0.53	0.69	1.54 (0.03)	0.73	
	RF ensemble	0.70	–	–	0.70	–	–	0.70	–	–	
	SMA^a^	0.70	1.57 (0.03)	0.97	0.70	1.57 (0.03)	0.94	0.70	1.58 (0.03)	0.97	
50%	Biased data	0.70	1.61 (−)	–	0.69	1.63 (−)	–	0.69	1.61 (−)	–	
	RUS	0.70	1.59 (0.03)	0.83	0.70	1.61 (0.03)	0.81	0.70	1.60 (0.03)	0.82	
	ROS	0.70	1.59 (0.02)	0.87	0.70	1.61 (0.02)	0.77	0.70	1.61 (0.02)	0.74	
	SMOTE	0.68	1.85 (0.02)	0.66	0.69	1.89 (0.02)	0.00	0.69	1.85 (0.02)	0.00	
	PS matching	0.69	1.44 (0.04)	0.29	0.69	1.49 (0.04)	0.60	0.69	1.57 (0.03)	0.89	
	RF ensemble	0.69	–	–	0.69	–	–	0.68	–	–	
	SMA^a^	0.70	1.59 (0.02)	0.89	0.70	1.59 (0.02)	0.90	0.70	1.59 (0.02)	0.90	
80%	Biased data	0.69	1.66 (−)	–	0.69	1.66 (−)	–	0.69	1.61 (−)	–	
	RUS	0.70	1.60 (0.05)	0.75	0.70	1.60 (0.05)	0.75	0.70	1.61 (0.03)	0.77	
	ROS	0.70	1.65 (0.02)	0.59	0.70	1.65 (0.02)	0.49	0.70	1.62 (0.02)	0.67	
	SMOTE	0.68	1.82 (0.03)	0.00	0.69	1.81 (0.03)	0.00	0.69	1.79 (0.02)	0.00	
	PS matching	0.70	1.50 (0.07)	0.67	0.69	1.50 (0.07)	0.67	0.69	1.49 (0.03)	0.49	
	RF ensemble	0.69	–	–	0.69	–	–	0.68	–	–	
	SMA^a^	0.70	1.56 (0.02)	0.78	0.70	1.57 (0.02)	0.82	0.70	1.56 (0.02)	0.80	

For the original data: AUC = 0.70; OR_*Z*_ (SE) = 1.58 (0.02); *I*_*Z*_ = 1.00. Only results for missing proportions of 15%, 50%, and 80% are shown.

a Estimates are averaged from *m* = 100 synthetic copies.

#### Impact of bias

**Figure 4:**
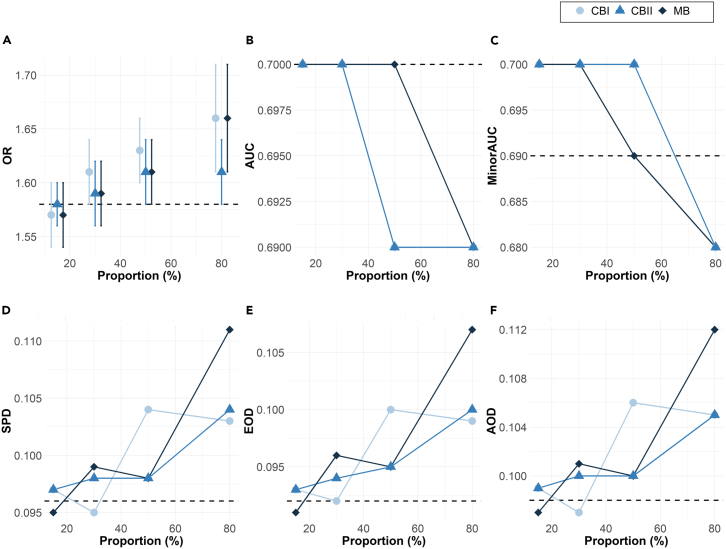
Bias type Impact of bias (increasing missing proportion) on the CCHS data in evaluating the (A) odds ratio (OR) and standard error of the biasing covariate, (B) the overall AUC, (C) minority-group AUC, and three fairness metrics: (D) statistical parity difference (SPD), (E) equal opportunity difference (EOD), and (F) average odds difference (AOD). The bias is assessed under marginal bias (MB), conditional bias I (CBI), and conditional bias II (CBII). The dashed line represents the ground-truth results in the original dataset.

As shown in Figure 4, the results for the impact of bias on the CCHS dataset are similar to what was observed in the simulations. As the proportion of observations excluded is increased from 15% to 95%, the discrepancy between the biased covariate estimates on the biased samples and that of the ground truth also increases.

Compared to the ground truth for CCHS data (odds ratio, OR_*Z*_ = 1.55), the estimated OR_*Z*_ of the biased data increases from 1.57 to 1.66 in the marginal case, similarly from 1.57 to 1.66 in the conditional case I, and from 1.58 to 1.61 in conditional case II. For the remaining datasets presented in Appendix D, a similar pattern of increasing deviations from the ground-truth estimates is observed.

The AUCs of the biased datasets consistently decline away from the ground truth with increasing missing proportions under marginal bias and decline rapidly for all types of high or extreme bias. Bias is generally most impactful on the performance of the category affected by the missing observations. As more observations are excluded from the minority group, minority AUC decreases and the deviation from the ground truth grows.

As the extent of bias grows, the three types of fairness metrics increasingly deviate from the ground-truth value, with the greatest deviation at the highest bias.

#### Performance of SMA relative to ground truth

**Figure 5:**
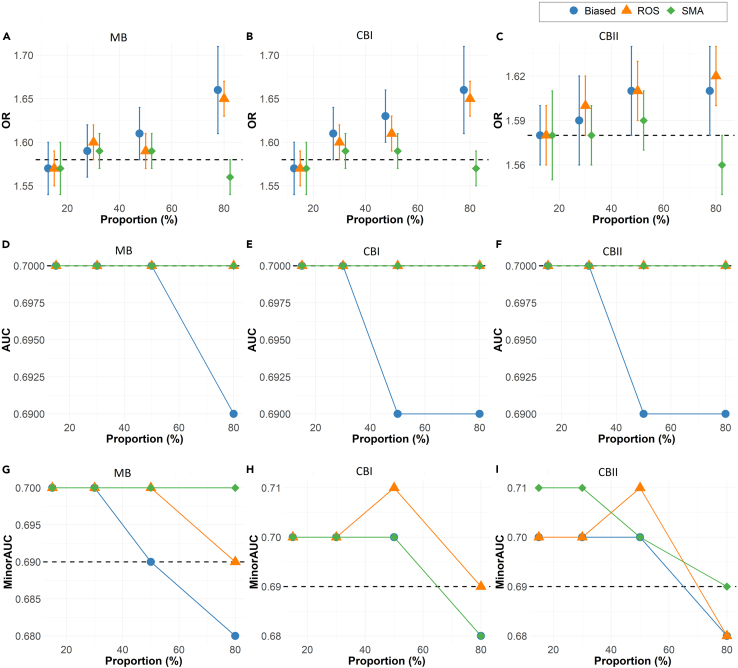
Bias mitigation approach Comparisons on the CCHS dataset of the OR estimates with and standard error of the biasing covariate (A–C), overall model AUCs (D–F), and minority-group AUC of the biasing covariate (G–I) for SMA, random oversampling (ROS, the best-performing alternative), and biased data. The dashed line represents the ground truth from unbiased data.

As shown in Figure 5 with the CCHS dataset results, as the bias is increased from 15% to 80%, the OR of SMA is closer to the ground truth compared to ROS, which is the best-performing alternative approach (the detailed result trends for the other approaches are shown in Table 3) for low to medium bias. The results for AUC show that both SMA and ROS are consistently close to the ground truth as bias increases.

Note that a generative model can increase the diversity of the datasets that are generated, which is the case for SMA. This means that prognostic models trained on these types of augmented datasets can perform better on unseen cases. Consequently, we did not treat an improvement in minority AUC relative to the ground truth as a problematic deviation.

We see similar results for the minority-group AUCs under marginal and conditional bias settings. For low to medium bias, SMA performs well relative to ROS. In high to extreme cases of bias, the results are less clear.

Additionally, in Table 3 we see that the confidence interval overlaps show that SMA offers the greatest overlap with the ground-truth estimate.

**Figure 6:**
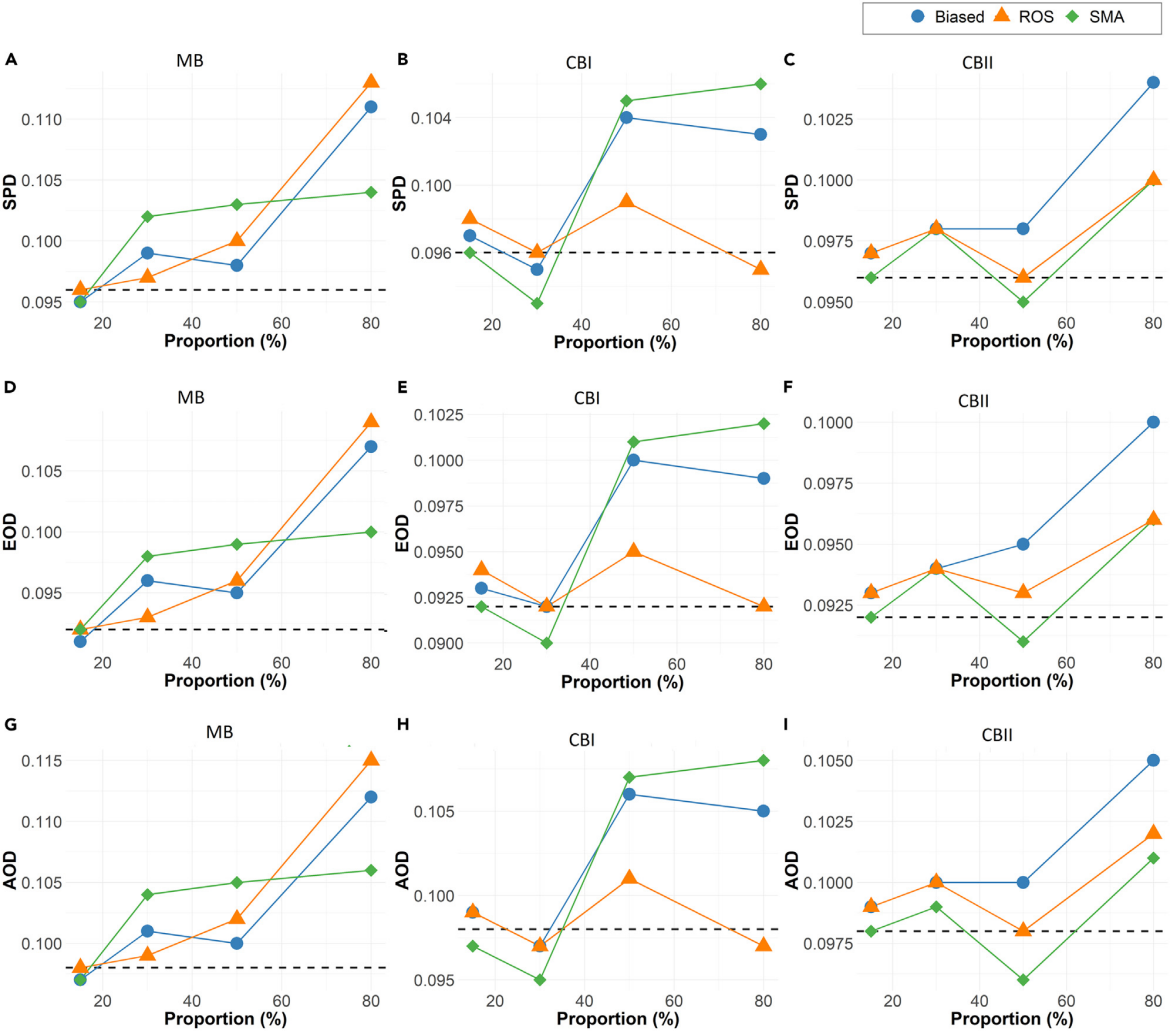
Bias mitigation approach Fairness metrics on the CCHS dataset: (A–C) the statistical parity difference (SPD), (D–F) equal opportunity difference (EOD), and (G–I) average odds difference (AOD) from the logistic regression model on the cardiovascular health data. The proportion of samples removed varied from 15% to 80%. Original cohort: SPD = 0.096, EOD = 0.092, and AOD = 0.098. The symbol **ǂ** indicates that the estimates are averaged from *m* = 100 synthetic copies. The dashed line represents the ground-truth fairness in the original dataset.

In evaluating fairness, we present estimates of fairness metrics evaluated (1) SPD, (2) EOD, and (3) AOD in Figure 6. The findings indicate that SMA generates fairness estimates that are best aligned with ground-truth estimates, as does ROS to some degree. Fairness estimates for the remaining datasets are shown in Tables S10, S13, and S16).

#### Performance of SMA relative to biased data

**Figure 7:**
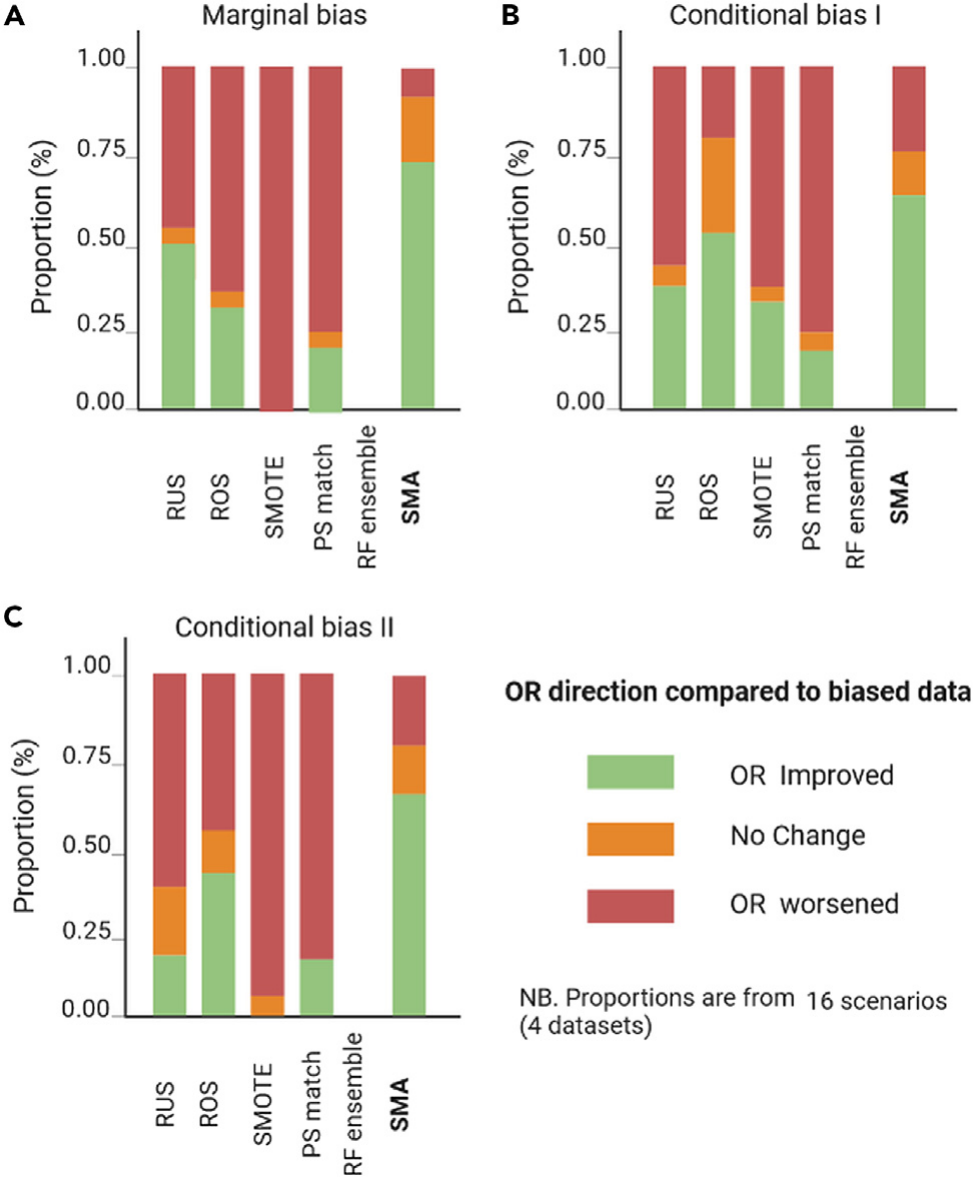
Summaries of the OR of the biasing covariate for the four real datasets The relative performance of each bias-mitigating approach compared to the biased data estimate is shown. The OR direction is considered improved if the difference between the model OR and the ground-truth estimate is less than the difference between the biased data OR and the ground truth. Summaries are over all four datasets, and proportions range from 15% to 80%.

**Figure 8:**
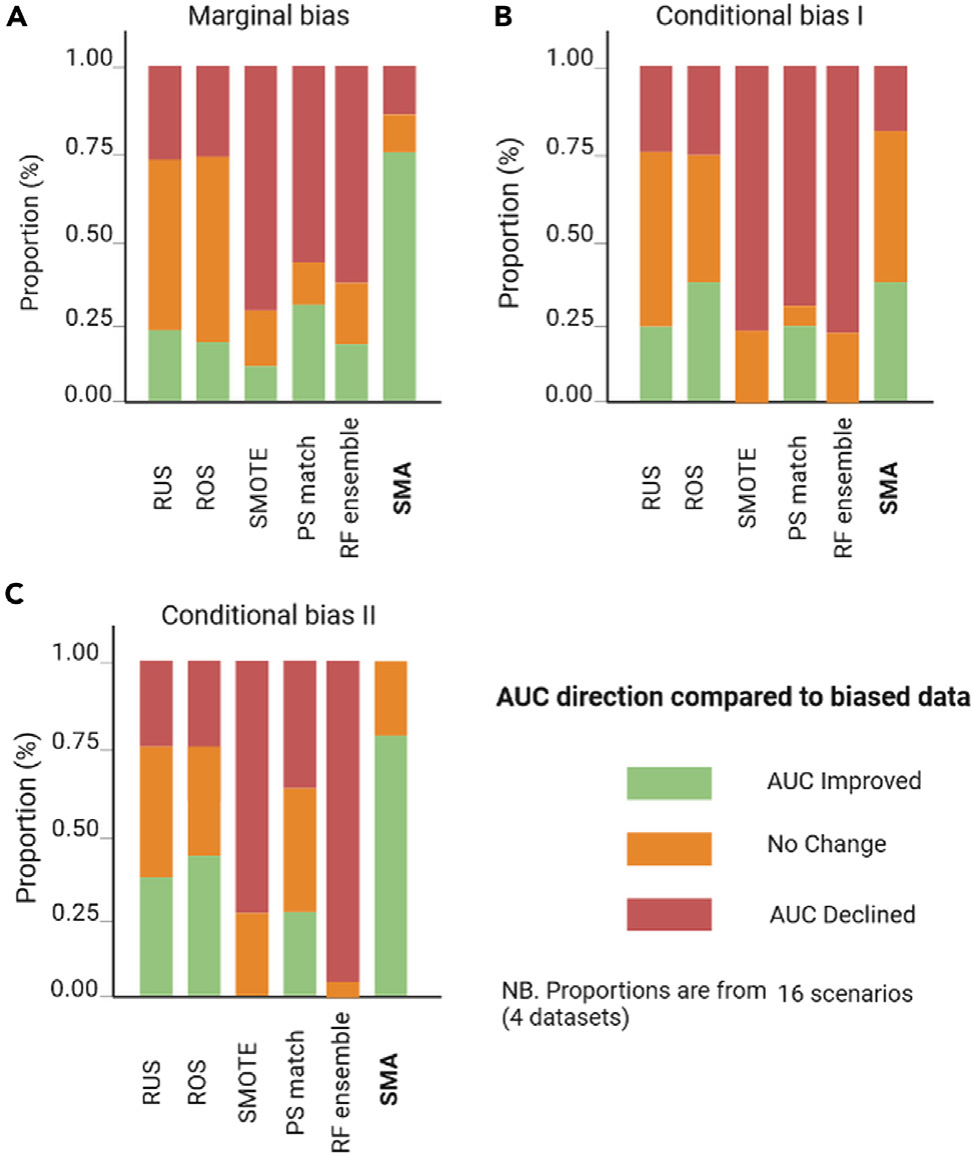
Summaries of the model AUC for the four real datasets The relative performance of each bias-mitigating approach compared to biased data results is shown. The model AUC is considered improved if the difference between the model AUC and the ground-truth estimate is less than the difference between the biased data AUC and the ground truth. Summaries are over all four datasets, and proportions range from 15% to 80%.

**Figure 9:**
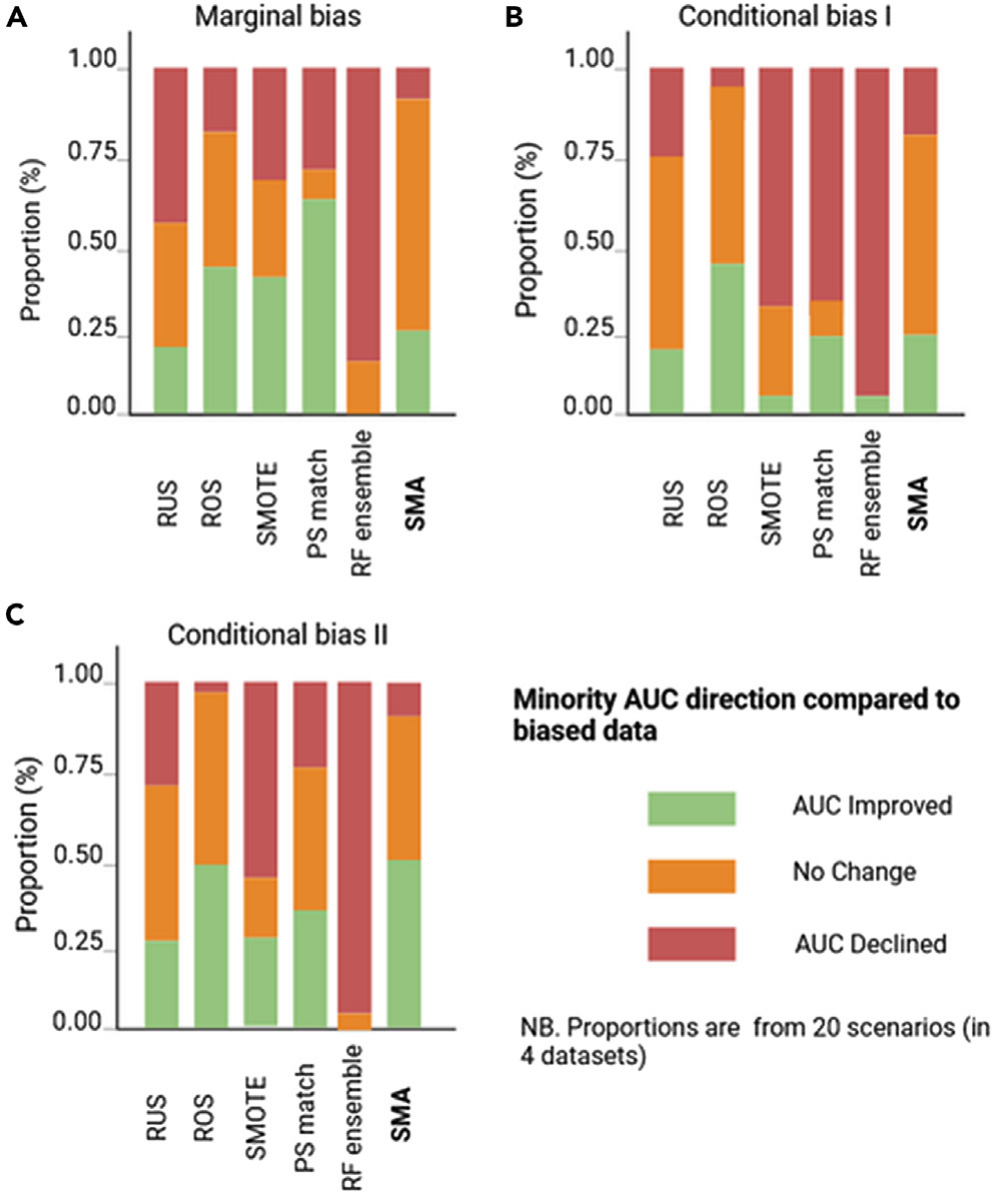
Summaries of the minority-group AUC over the four real datasets The relative performance of each bias-mitigating approach compared to biased data results is shown. The minority AUC is considered improved if the difference between the model minority AUC and the ground-truth estimate is less than the difference between the biased data minority AUC and the ground truth. Summaries are over all bias proportions: 15%, 30%, 50%, 80%, and 95%.

**Figure 10:**
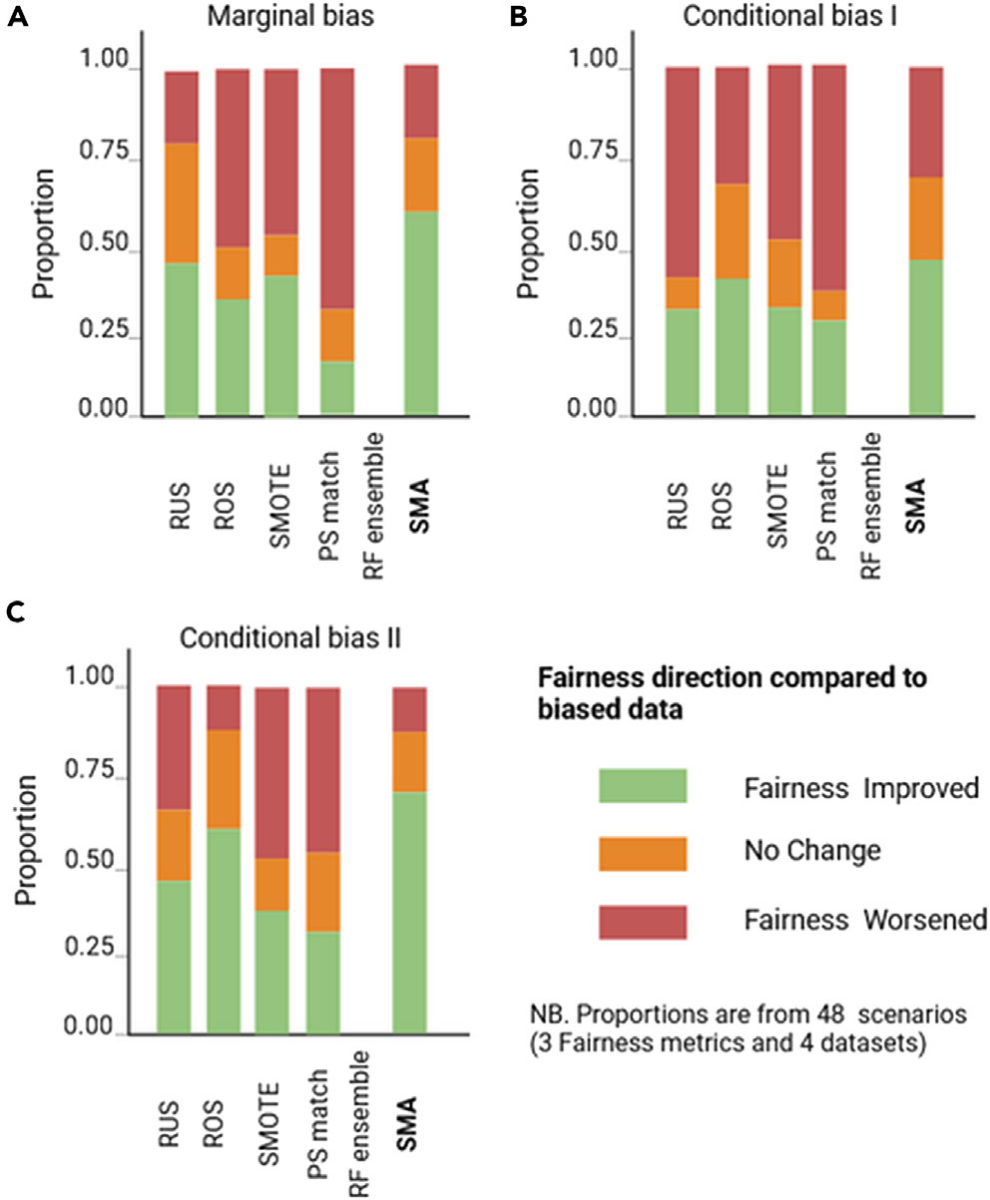
Summaries of the fairness metrics SPD, EOD, and AOD over the four real datasets The relative performance of each bias-mitigating approach compared to biased data results is shown. Fairness is considered improved if the difference between the model fairness and the ground-truth estimate is less than the difference between the biased data fairness and the ground truth. Summaries are over all four datasets, and proportions range from 15% to 80%.

A summary of the relative performance of the bias-mitigating approaches over biased datasets (using the original cohort as the reference) is presented in Figures 7, 8, 9, and 10 for all four datasets. Specifically, we show comparisons of their relative performances based on AUCs, OR estimates, the minority-group AUC, and fairness metrics across all real datasets and levels of bias, demonstrating that SMA consistently outperforms other approaches across multiple scenarios (with missing proportions ranging from 15% to 80% for each of the three bias settings). These results are more pronounced for low to medium bias, and these are shown in Appendix D.5 in supplemental experimental procedures.

Of note, the relative performances of the AUCs and ORs are estimated by comparing the differences between the estimates from each approach and the ground truth against the corresponding difference between the biased data estimates and the ground truth. For example, if the ground-truth AUC (AUC^g^) is estimated as 0.85, the biased data AUC (AUC^b^) as 0.80, and the AUC for SMA (AUC^SMA^) as 0.82, then we say the AUC improved for SMA since |AUC^g^ − AUC^SMA^| < |AUC^g^ − AUC^b^|. Similarly, we say the AUC worsened if |AUC^g^ − AUC^SMA^| > |AUC^g^ − AUC^b^| or that there is no change if the absolute differences are equal. The same convention was applied to the Brier scores, the OR estimates, and the minority-group AUCs.

For fairness, the definition is modified to include closeness to the ground-truth estimate or perfect fairness (SPD = 0, EOD = 0, AOD = 0). Hence, the fairness of an approach is considered improved if the SPD, EOD, and AOD estimates are closer to the ground-truth estimates or zero. Conversely, fairness is said to have worsened if the estimate is not closer to the ground truth and not closer to zero.

We also independently generated the corresponding summary plots for (1) low to medium bias and (2) high bias for each of the metrics evaluated, which are provided in Appendix D in supplemental experimental procedures.

## Discussion

### Summary

Learning from biased data presents a challenging task in biomedical research. The literature lists multiple modeling, prediction, and generalization problems with biased datasets and raises ethical questions about the application of models trained on biased datasets. As a result, prognostics and inferences done on such datasets are not always useful in clinical settings.

In this article, we first evaluated the impact of bias on analytical results. Our results indicated the following.

(1) The parameter estimates in logistic regression models increasingly deviate from the ground truth as the extent of bias grows and becomes evident even at low to moderate levels of data bias. This is the case for different types of bias (marginal and conditional) and is a consistent finding in simulations and on real datasets.
(2) Biased data demonstrate a reduction in fairness metrics, and this grows monotonically with the extent of bias induced on the datasets. This is to be expected as the fairness metrics evaluate bias directly.
(3) Model calibration as assessed using the Brier score is not affected materially by data bias, and these results are consistent even for high rates of bias.
(4) Overall prediction model accuracy decreases when bias is high or extreme, and the minority-group AUC was better than the ground truth for low to medium bias but decreased as bias grows.

We proposed a bias-mitigation approach called SMA. The method augments the biased datasets with synthetically generated copies of the minority group to reconstruct the full bias-mitigated dataset. This proposed method was compared to standard approaches such as RUS, ROS, SMOTE, RF ensembles, and PS matching. Our results through simulations and real datasets on a logistic regression workload indicated that:

(1) SMA has consistently higher confidence interval overlap with the ground truth on the biased covariate parameter.
(2) ciFor low to medium levels of bias, the SMA gave the best performance across methods evaluated in terms of the precision of the parameter estimate, the overall model AUC, and fairness for mitigating the effects on parameter estimates by having the closest values (or better values for fairness) to the ground truth. While SMA sometimes performs well for high bias, there is no other single method that consistently performs better than SMA for the high bias case.
(3) For low to medium levels of bias, SMA had good performance for the prediction and calibration of the minority group.

The performances of the traditional approaches evaluated in this study are consistent with previous findings.^55^^,^^56^^,^^57^ In the presence of marginal bias where observations are missing from the data solely based on their membership to a given predictor category, rebalancing approaches such as ROS, SMOTE, and RUS tend to perform reasonably well in low to moderate bias. However, as the severity of the bias increases, their effectiveness declines. In more complex conditional bias settings, where bias is induced by more than one covariate, these methods are not always reliable or reproducible. The SMA approach overcomes these obstacles by offering more accurate, stable, and comparable results to the ground truth.

While our study focused on biased covariates with binary values, this does not limit the method from being applied in cases where the biased covariate is multi-class with more than two categories. The generative model used for SMA can synthesize variables with multiple classes to add more individuals with the bias classes.

Overall, the synthetic augmentation approach described in this article can be a robust solution to mitigate data bias at the data-analysis stage. This is the case for low to medium levels of data bias and across multiple criteria. Although traditional methods such as ROS, SMOTE (or modifications), and PS matching remain useful, their effectiveness is greatly affected by how the bias is induced in the data and the amount of bias present. For high or extreme bias scenarios, there is no consistently high-performing bias-mitigation method across all evaluation metrics.

### Limitations

The methods presented here require minimal knowledge of the nature of the bias by the analyst: only the variable and category affected, and the extent of bias on that variable. This would make the burden of using these bias-mitigation methods relatively small.

The effectiveness of bias-mitigation approaches may improve if the analyst can also identify the type of bias (whether marginal or conditional on one or more covariates). For example, while ROS was implemented solely based on oversampling observations from the minority group of the biased covariate, its performance in conditional bias settings may be better if the analyst could discern the additional covariate to condition upon. In practice, this is a difficult prerequisite to meet and, therefore, our study arguably makes the most practical assumptions.

The sample size of the biased data used in the SMA synthesis process is another potential limitation. In extreme bias settings or small sample settings, the data-synthesis process may not have enough information on the under-represented group to accurately capture the relationships and patterns in the data. This can result in synthetic data that poorly represent the ground truth and may not effectively mitigate bias. For example, in the breast cancer data, which had a relatively small sample size compared to the other datasets, the proposed SMA approach performed least effectively. In such small samples, even a moderate amount of bias could profoundly impact the effectiveness of the proposed bias-mitigating approaches.

Another question is whether the application of bias-mitigation methods, such as SMA, will create disincentives to recruit under-represented groups into health studies. However, there will be many situations where historical or existing data are being used and, therefore, there is no practical opportunity to recruit additional participants. In those cases, methods such as SMA can help address biases that may be introduced in models using existing data. In situations where new data are being collected, methods such as SMA can be used to perform a sensitivity analysis to inform additional recruitment decisions (by comparing the results from the original biased dataset with the results from the bias-mitigated dataset).

Variations of SMOTE and sampling techniques, including SMOTEBoost,^58^ RUSBoost,^59^ BalancedBoost,^60^ and OverBagging,^61^ have been proposed in the literature. The relative performance of these alternative methods may differ from the default approaches presented in this study. A comprehensive comparison of these variations to SMA represents a promising avenue for future investigation.

## Experimental procedures

### Resource availability

#### Lead contact

Further information and requests for resources should be directed to and will be fulfilled by the lead contact, Khaled El Emam (kelemam@ehealthinformation.ca).

#### Materials availability

This study did not generate new materials.

#### Data and code availability

The N0147 dataset can be requested from Project Data Sphere (https://www.projectdatasphere.org/). The CCHS dataset can be requested from Statistics Canada (https://www.statcan.gc.ca/en/microdata/data-centres). The Danish Colorectal Cancer Group (DCCG) dataset can be requested from the Danish Colon Cancer Group (https://www.dmcg.dk/). The breast cancer dataset is publicly available from the UC Irvine machine-learning repository and also available in the code and data deposit for this paper. The R code and public datasets used for this study are available for download from https://ehealthinformation.ca/blog/EHIL%20Blog/Bias-mitigation and on OSF (https://doi.org/10.17605/OSF.IO/RKF9T).^62^

### Ethics and consent to participate

This study was approved by the CHEO Research Institute Research Ethics Board protocol CHEOREB #23/34X. All research was performed in accordance with relevant guidelines/regulations. Patient consent was not required by the Institutional Review Board.

### Proposed approach: Synthetic minority augmentation

#### SMA process

**Figure 11:**
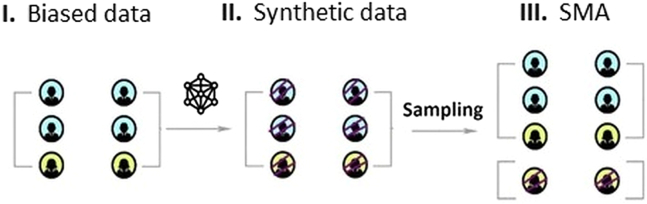
A schematic for synthetic data augmentation from biased data From (I) to (II), a synthetic version of the biased data is generated. In step (III), the synthetic minority group is sampled from (II) and augmented with the biased data in (I) to generate a rebalanced dataset.

We propose a bias-mitigating approach that is based on synthetic data generation. By augmenting the minority group in biased datasets with synthetic records, we reconstruct full data samples that are reflective of the baseline distribution. The approach is termed SMA. A schematic of the SMA approach is provided in Figure 11.

Starting with a biased sample, SMA can be implemented in three stages.

(1) Synthesis stage: construct a synthetic version of the biased data using sequential synthesis based on gradient-boosted decision trees.
(2) Sampling stage: sample observations from the minority group of the generated synthetic dataset.
(3) Augmentation stage: add the sampled observations from step 2 to the biased data to create a complete, bias-mitigated dataset.

##### Step 1: Synthesis stage

The type of generative model used was a sequential tree-based synthesizer.^63^ This type of generative model has been used to synthesize health and social sciences data,^64^^,^^65^^,^^66^^,^^67^^,^^68^^,^^69^^,^^70^^,^^71^^,^^72^ and has been applied in research studies on synthetic data.^64^^,^^73^^,^^74^

To account for the stochastic nature of the generative model, we synthesize *m* copies of the biased data, which are later combined in the downstream analysis. A summary of the algorithm is presented below.

##### Step 2: Sampling stage

The next step is to draw synthetic records of the minority group from the data synthesized. For each of the *m* synthetic datasets generated, we sample *k* records from the minority group so that its distribution is the same as the baseline distribution. For example, in a biased dataset with under-represented female participants, we can construct synthetic samples of only the minority group by sampling synthetic copies of female observations. It is this minority-group sample that is used in stage 3.

##### Step 3: Augmentation stage

In the final stage, we augment the starting biased sample with the sampled synthetic observations of the minority group. We thus end up with a larger dataset involving additional observations from the under-represented category, ensuring a distribution consistent with the baseline distribution. As the released data are composed of a mixture of both original biased data and synthetic data, this technique can be viewed as a form of partial data synthesis.

For each of the *m* sampled synthetic minority groups, we create a new, rebalanced dataset for analysis. Each dataset is independently analyzed, and the results are subsequently combined according to a clearly defined combining rule. For example, regression effect estimates can be reported by taking the average across the *m* samples, or average AUC estimates could be reported for predictive performance.

#### SMA synthetic data-generation algorithm

For data synthesis, we apply decision trees sequentially. Each model in the sequence was trained using a gradient-boosted decision tree,^75^^,^^76^ with Bayesian optimization and 5-fold cross-validation for hyperparameter selection.^77^ The variable sequence is optimized using a particle swarm algorithm.^63^

**Figure 12:**
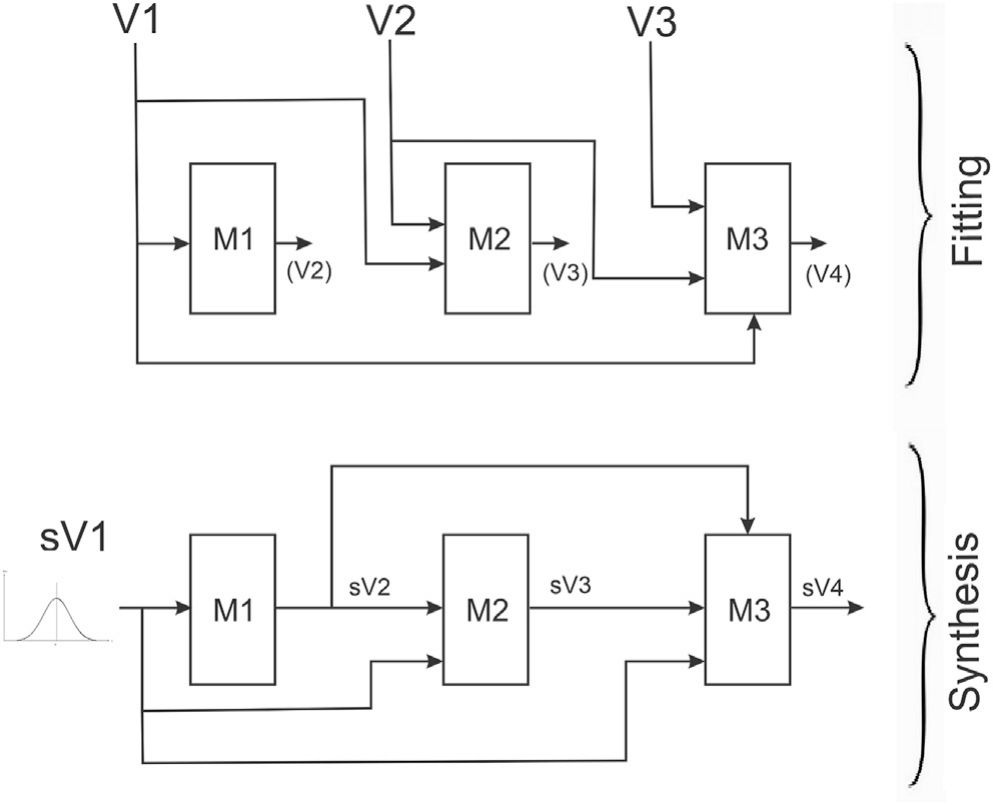
Illustration of the synthesis process for a four-variable dataset

The process is illustrated in Figure 12 for a four-variable dataset, V1–V4. In the fitting phase, three models are constructed, M1–M3. As shown, the first model takes as input V1 and produces V2 as the outcome. The nature of the variables, whether categorical or continuous, does not affect the process, as the model adjusts to become either a classification tree or a regression tree accordingly. The second model in the sequence takes V1 and V2 as input with V3 as the outcome, and so on.

The synthesis step is initiated by sampling from the actual or fitted distribution of the first variable, V1. This creates the synthetic version of that variable, sV1. Sampled values are then entered into the first model to generate the distribution of sV2 in the predicted terminal node of the tree. The synthetic value of sV2 is either sampled according to the predicted probabilities (for categorical variables) or smoothed using a kernel density estimator with boundary correction (for continuous variables),^78^ with bandwidth computed from the original data.

Having generated two synthetic values, sV1 and sV2, these form the input for model M2 to produce the distribution of sV3. Again, the generated synthetic value is either sampled from that predicted distribution or smoothed. The process proceeds in this manner until all variables are synthesized.

There are advantages to using gradient-boosted decision trees. Previous studies have shown that discriminative and regression models on heterogeneous datasets (i.e., those with continuous and categorical values, and with missingness) using boosted trees perform better than artificial neural network architectures on tabular datasets.^79^^,^^80^^,^^81^^,^^82^^,^^83^ Sequential synthesis implements multiple boosted decision trees in a specific order, and existing evaluations suggest that this is a relatively competitive type of generative model relative to artificial neural networks for tabular data.^84^

#### Synthesis pre- and post-processing

Pre- and post-processing are important to ensure that a generative model can handle realistic datasets. The following summarizes the steps in the SMA implementation.

For the synthesis of categorical variables, synthetic values are generated based on predicted probabilities. In general, boosted trees do not output correct probabilities and these need to be calibrated, especially as the number of iterations increases.^85^ In addition, for imbalanced categorical outcomes, rebalancing of the classes gives incorrect probabilities. Therefore, the predicted probabilities are adjusted using beta calibration.^86^

For each continuous variable *V*_*i*_ being synthesized, we first convert them to a Gaussian distribution before synthesis. The empirical cdf was applied to each variable *F*_*i*_(*V*_*i*_), then the quantile function for the standard normal was applied, Φ^−1^(*F*_*i*_(*V*_*i*_)), which is used for synthesis. After synthesis, the generated values ${sV}_{i}$ are converted back as ${F_{i}}^{-1}\left(\Phi \left({sV}_{i}\right)\right)$. This approach improves the modeling of continuous variables with long tails and multi-modal variables.

The variables used have a shadow variable added with a missingness indicator. This allows for the modeling of missingness in the output data.

### Evaluation protocol

The analytic workload that we assume in our evaluations is logistic regression (LR). LR models are common in health research for diagnostic and prognostic modeling.^87^ A recent systematic review has shown that LR performance is comparable to the use of machine-learning models for clinical prediction workloads.^88^ Therefore, it represents a realistic workload to assess covariate bias.

**Figure 13:**
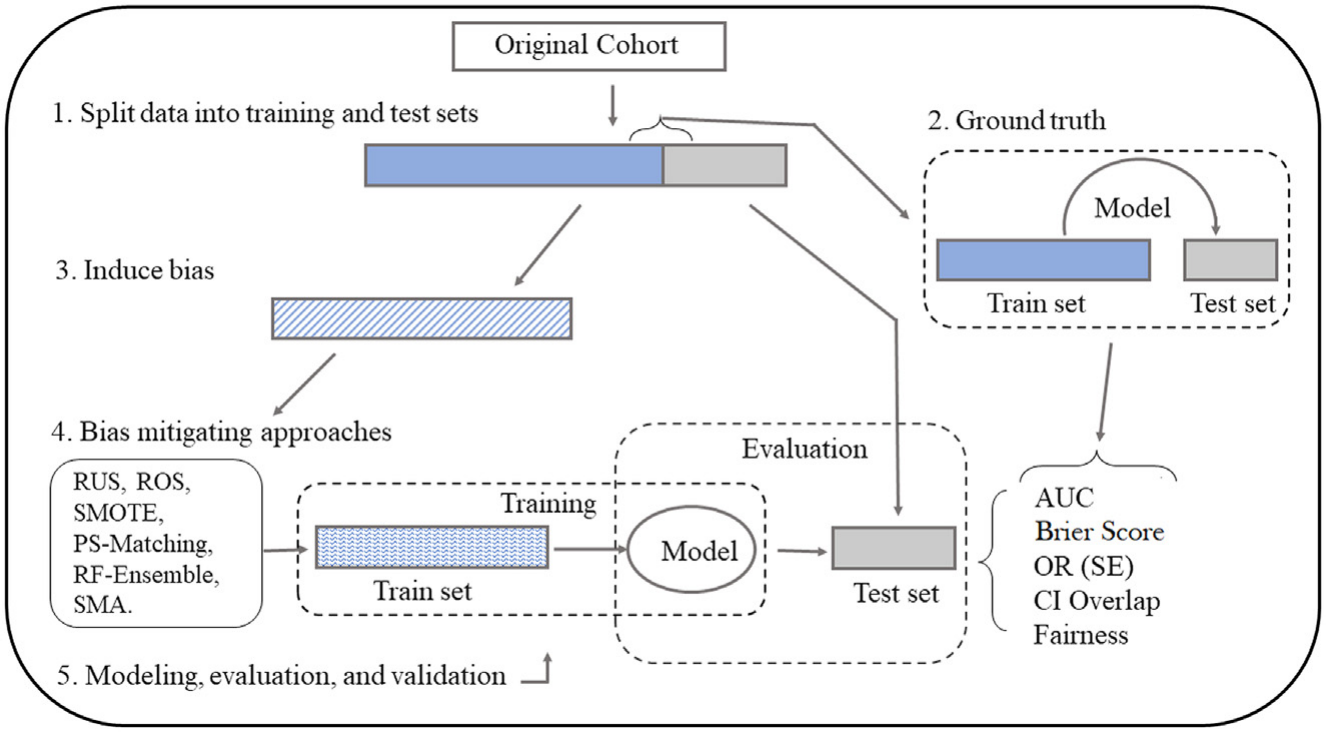
Schematic for training and evaluating bias-mitigating approaches

To evaluate the methods for mitigating data bias, we follow the process summarized in Figure 13. Starting with the original cohort which we assume represents the baseline distribution, we randomly split the dataset into training and holdout partitions using 70:30 proportions. A baseline “ground truth” LR is performed on the original training partition, and predictions are evaluated on the holdout partition.

We then induce different types of data bias into the training partition to obtain biased training partitions. An LR model of the same form is then fitted to the biased training data, and its prediction performance is assessed using the holdout partition. Subsequently, we apply different mitigation techniques to the training data, train the LR models on the mitigated datasets, and evaluate their prediction performance on the same holdout dataset.

It is important to note that the same holdout partition is employed for all analyses to ensure consistent results.

Two types of validation were carried out. The “full model” validation computed the performance metrics on all the training/test datasets. The disaggregated or “stratified model” validation computed the prediction performance separately for the minority and the majority groups of the biased covariate on the holdout dataset. The latter validation determines how model performance varies by group.

The performance of bias-mitigation methods was compared to the ground truth. The bias-mitigation methods evaluated were RUS, ROS, SMOTE, PS matching, RF ensembles, and synthetic data augmentation. The selected approaches represent each of the main classifications of the bias-mitigating techniques in the literature and are readily accessible through standard statistical programming packages. They are described further in Appendix A in supplemental experimental procedures.

For all bias-mitigation methods, the only information that is assumed known by the analyst is the extent of bias and the biased covariate category. For example, the analyst might know that the biased covariate is “sex,” the under-represented group is “female,” and the extent of under-representation relative to the baseline distribution is 15%. In practice, it is typical for analysts to have access to just this level of detail and not much else about the nature of the biased covariate, such as any conditioning information.

### Inducing data bias

To induce sample selection bias, we mimic the two scenarios of biased data presented in Winship and Mare^15^ and Heitjan and Basu.^89^ The first bias scenario occurs when we explicitly leave out observations from a specific category of the biased covariate, independent of any other covariate in the data. For example, Goldberger^90^ explored the problem of estimating the effect of education (biased covariate) on income (outcome) where only individuals with incomes below $15,000 were deemed to have met the inclusion criteria. The second bias scenario involves selecting observations based on a combination of the biased covariate and other measured covariates. For example, by conditioning on gender, the same study excluded female participants whose incomes were below $15,000. More specifically, we classify these biases as follows.

(1) Marginal bias: this represents the simplest form of sampling bias where the biased covariate categories are randomly removed, irrespective of the values of other covariates in the model.
(2) Conditional bias: here, observations are removed from one or more biased covariate categories based on other covariates in the dataset. If the biased covariate is *Z*, then observations in one category of *Z*, say *Z* = 1, are excluded provided another covariate, *X*, is greater than the threshold *x∗*. We consider two types:
(3) Conditional bias I: when the odds of *X* in *Z* range from weak to moderate (equivalently, the biased covariate *X* is not strongly correlated with the biased covariate *Z*); and
(4) Conditional bias II: when the odds of *X* in *Z* are high, e.g., observations are removed from the predictor category *Z* = 1 if they are strongly correlated with the covariate *X*.

The strength of the associations between the biased covariate *Z* and the conditioned covariate *X* is determined by conducting a univariable LR between *Z* and *X*. While the sampling biases presented in (1) and (2) can be viewed as forms of missing-at-random (MAR) conditions,^91^ the main difference between our setting and the traditional MAR setup is that we are primarily focused on missing observations instead of missing covariate values in some observations. In each (marginal or conditional bias) case, the sampling proportion could be varied to create training datasets of different imbalance ratios. Additionally, both positive and negative associations were considered under conditional bias, regardless of the strength of the association between *Z* and *X*.

For uniformity, the imbalance ratio of the biased covariate group is taken to determine the severity of the dataset bias. Thus, the bias percentage is only determined by the proportion of observations removed from the biased covariate, even under conditional bias.

In our analysis, the amount of bias induced on the biased covariate was varied by changing the proportion of observations removed in the biased covariate group from low (proportion = 15% or 30%), moderate (proportion = 50%), high (proportion = 80%), and extreme (proportion = 95%).

### Evaluation metrics for model performance and fairness

The metrics evaluated for comparing the performances of the LR models are: (1) the overall model AUCs computed on the holdout partition; (2) the estimate of the biased covariate coefficient and the associated standard error; (3) the 95% confidence interval overlap of the biased covariate coefficient of the original cohort (ground truth) with each bias-mitigation approach^92^; (4) AUCs and Brier scores for predicting only the minority group of the biased covariate (this is the stratified model); and (5) model fairness.

Note that the biased dataset (with no bias-mitigation approaches applied) will have fewer observations than all of the other training datasets. This means that the standard errors will be higher by definition, and the confidence intervals will be wider. Since this is partially an artifact of the sample size, we do not present these values for that particular dataset.

Additionally, several commonly used fairness metrics were evaluated to assess the differences in classifications between the categories of the biased covariate. Specifically, we estimate the (1) SPD,^93^ (2) EOD,^94^ and (3) AOD.^95^

SPD compares the probability of the outcome given the one category of the biased covariate to the probability of the outcome given the other predictor category. SPD can be expressed as

(Equation 1) $$\text{SPD}=\mathrm{Pr}\left[\hat{Y}=1|Z=1,X=x\right]-\;\mathrm{Pr}\left[\hat{Y}=1|Z=0,X=x\right]\text{,}$$

where $\hat{Y}$ is the predicted value of the outcome, *Z* is the binary predictor of interest, and *X* represents the remaining covariates. An SPD estimate of zero indicates that the classification algorithm provides equal probabilities of the outcomes for both groups of *Z*. Thus, the closer the estimate is to zero, the fairer the approach.

EOD compares the true-positive rates (TPRs) between the categories of the biased covariate. The metric aims to ensure that individuals from different groups have equal chances of being correctly classified for a positive outcome when they should indeed receive it. EOD is given by the expression

(Equation 2) $$\text{EOD}=\mathrm{Pr}\left[\hat{Y}|\;Y=1,Z=1,X=x\right]\;-\;\mathrm{Pr}\left[\hat{Y}|Y=1,Z=0,X=x\right]\text{,}$$

where an EOD of zero indicates that the classification algorithm provides equal opportunity for both groups, as they have the same TPRs (TPR = *Pr*[$\hat{Y}$ = 1|*Y* = 1,*Z*]) for *Z* = 1 and *Z* = 0.

AOD estimates the average differences between the false-positive rate and the TPR of the biased covariate categories. AOD can be expressed as

(Equation 3) $$\text{AOD}=\left|\left({\text{FPR}}_{\text{MN}}\;–\;{\text{FPR}}_{\text{MJ}}\right)+\left({\text{TPR}}_{\text{MN}}-{\text{TPR}}_{\text{MJ}}\right)\right|/2\text{,}$$

where the false-positive rates of the minor (MN) category FPR_MN_ = *Pr*[$\hat{Y}$ = 1|*Y* = 0,*Z* = 1] and that of the major (MJ) category FPR_MJ_ = *Pr*[$\hat{Y}$ = 1|*Y* = 0,*Z* = 0]. The TPRs of the minority group TPR_MN_ = *Pr*[$\hat{Y}=1$|*Y* = 1,*Z* = 1] and that of the major TPR_MJ_ = *Pr*[$\hat{Y}=1$|*Y* = 1,*Z* = 0]).

Despite the optimal score for the three fairness metrics being zero, our analysis utilizes the ground-truth estimates of these metrics as the benchmark. For SPD and EOD, deviations from zero, whether positive or negative, indicate the degree of dissimilarity between the groups.

### Datasets

#### Simulated data

First, we consider a simulation setting involving a binary outcome *Y*, binary predictor variable *Z* sampled from the binomial(p = 0.5), two binary variables *X*_1_ and *X*_2_ with the observations of *X*_2_ given *Z* = 1 sampled from the binomial(p = 0.4), while the remaining observations of *X*_2_ given *Z* = 0 were sampled from binomial(p = 0.35). All of the observations of *X*_1_ were sampled from binomial(p = 0.6), independent of *Z*. We also considered a continuous predictor of the outcome *U* generated from the log-normal(12,3.5) and independent of the other covariates. An effect modifier term for the interaction between *Z* and *X*_2_ was also included. We define the LR model as:

(Equation 4) $$P\left(Y=1\right)=\text{expit}\left(\alpha +\beta_{Z}Z+\beta_{X1}X_{1}+\beta_{X2}X_{2}+\beta_{ZX2}{ZX}_{2}+\beta_{U}U\right)\text{,}$$

where expit(*t*) = exp(*t*)/(1 + exp(*t*)). For the set of fixed parameters (*α* = log(0.5), *β*_*Z*_ = log(1.25), *β*_*X*1_ = log(0.3), *β*_*X*2_ = log(2), *β*_*ZX*2_ = −0.47, *β*_U_ = log(0.5)), we construct the full cohort data of 5,000 observations before inducing bias in the sampled training dataset. The simulation is repeated to generate 500 datasets. For all simulated samples, the full cohort (with equal proportions of *Z* = 1 and *Z* = 0) was considered the ground truth. A summary of the distributions of the baseline variables, stratified by predictor *Z*, is provided in Appendix B in supplemental experimental procedures.

To induce marginal bias in the training dataset, observations were randomly sampled and removed from the minority-group predictor category *Z* = 0, independent of the observed values of the other predictors in the dataset. For the conditional bias I case, observations were randomly removed from the minority-group predictor category *Z* = 0 if they had the covariate value *X*_1_ = 0. That is, we conditioned the exclusion of observations in the training cohort based on a covariate *X*_1_ that is weakly associated with the biased covariate *Z* (*β* = 0.068, p value = 0.325). For the second conditional case (conditional bias II), we condition inclusion based on a covariate *X*_2_ that is strongly associated with the predictor *Z* (*β* = 0.187, p value = 0.009). Thus, the observations excluded from the training cohort had predictor values *Z* = 0 and *X*_2_ = 0. All of the primary analyses were based on these settings.

In addition to the fixed parameter (i.e., *β*_*Z*_ = log(1.25)) used for the biased covariate in the primary analysis, the value of *β*_*Z*_ was varied to assess the robustness of the simulation results. In particular, the effect size of the biased covariate *β*_*Z*_ is also varied from small to large, where the definitions of small to large effect sizes are based on previous recommendations.^96^^,^^97^ A null effect value was also evaluated (i.e., *β*_*Z*_ = log(1.01)) to allow us to simulate the case when a biased covariate has no impact on the outcome (and hence bias-mitigation approaches would be expected to have no impact).

For each simulated dataset, we follow the evaluation protocol in “proposed approach: synthetic minority augmentation” to estimate the AUC, the effect estimates of the biased covariate, the standard deviation over the simulations, and the corresponding confidence interval overlap of each simulated data with the ground truth estimate. We also estimate the statistical parity, the EOD, and the AOD, as defined in “inducing data bias.”

#### Real datasets

We compared the approaches on four biomedical datasets: the cardiovascular health data from the CCHS of 2014,^98^ the N0147 colon cancer trial data from the Project Data Sphere initiative,^99^ the Danish colon cancer registry dataset from the DCCG database,^100^ and the breast cancer data from the UCI machine-learning repository.^101^ In Table 1, we present a summary of these datasets. The results for the CCHS dataset are presented in the main text while the results for the remaining three datasets are shown in Appendix D in supplemental experimental procedures. Overall summaries across all datasets are also presented.

The datasets considered had varying proportions of imbalance in the biased covariate and different sample sizes to reflect the diversity of datasets in real-world settings. Our analysis thus assumes each dataset as the ground truth before evaluating the impact of additional covariate bias. As in the simulated data setting, marginal bias was induced in each training set by removing observations from the minority group of the biased covariate (Table 1, column 4), independent of any other covariate. To induce the first conditional bias (conditional bias I), we condition the removal of observations from the minority-group predictor category based on a predictor that is weakly or moderately associated with the biased covariate (CBI in column 5 of Table 1). For the second conditional case (conditional bias II), we condition exclusion on the predictor labeled CBII (column 5), which is strongly associated with the biased covariate. A detailed description of the model-fitting procedure utilized for each dataset is provided in Appendix C in supplemental experimental procedures.

#### Inducing data bias

The proportions of observations excluded under each bias setting were set at 15%, 30%, 50%, and 80%, respectively. For datasets with small samples, removing 95% of observations in the biasing group is not always realistic, as the remaining samples might be too small to perform parameter estimation. Thus, for uniformity, the 95% missing proportion was excluded in the real dataset examples. We follow the evaluation protocol in “proposed approach: synthetic minority augmentation” to compare the performance of the approaches on the datasets.

For the CCHS dataset, a marginal bias case was induced by randomly sampling and removing observations from the training set if they were female, irrespective of their other covariates. The two conditional bias settings were induced by conditioning on covariates that were weakly and strongly associated with gender, respectively. In the first conditional case, female participants were only excluded from the training set if they were not new immigrants (new immigration is weakly associated with gender, *β* = −0.059, p value = 0.064). In the second case, observations were removed from the training set if they were female and belonged to a specific marital category (e.g., marital status = yes). Marital status was selected as it is strongly associated with gender (*β* = 0.158, p value <0.001). A detailed description of the variables conditioned on in each of the remaining datasets is presented in Appendix C in supplemental experimental procedures.

## Supplemental information

Document S1. Supplemental experimental procedures, Figures S1–S15, and Tables S1–S20

Document S2. Article plus supplemental information
