# Supplementary material for: An evaluation of synthetic data augmentation for mitigating covariate bias in health data
Source: Patterns (N Y). 2024 Feb 29;5(4):100946. doi: 10.1016/j.patter.2024.100946 (PMC11026977; doi:10.1016/j.patter.2024.100946)
Supplement: Document S1. Supplemental experimental procedures, Figures S1–S15, and Tables S1–S20 [file mmc1.pdf]

Patterns, Volume *5*

Supplemental information

An evaluation of synthetic data augmentation for mitigating covariate bias in health data

Lamin Juwara, Alaa El-Hussuna, and Khaled El Emam

Supplemental Experimental Procedures

Appendix A: Review of approaches for mitigating data bias

Bias mitigation techniques can broadly be classified into three categories: rebalancing approaches, algorithmic approaches, and post-processing approaches. Some of these approaches are review below.

A.1 Rebalancing Approaches

Rebalancing approaches attempt to construct datasets that are reflective of the true population. Starting with the imbalance dataset D^I, the goal is to produce a balanced dataset D^b that provides a good approximation for the underlying data population D. The approaches can take the form of subsampling (e.g., random undersampling, oversampling, and SMOTE) or matching (e.g., propensity score matching) to remove the effect of imbalance introduced by the biased covariate. Although subsampling remains a popular method for addressing outcome imbalance, we restrict our usage of the approach to rebalance imbalanced covariates distributions.

A.1.1 Random Undersampling (RUS)

RUS is a data pre-processing approach to rebalance unevenly distributed classes in imbalanced datasets. It attains balance by randomly removing excess observations in the majority class of the primary biased covariate [1]. RUS is often extensively applied when analyzing uneven outcome class distributions [2], however, we adopt its use for rebalancing the class distributions in categorical covariates in biased datasets [3]. Although RUS has the advantage of reducing the computational cost associated with learning from excess data, it also introduces several challenges. It has the potential to distort the distributions of other relevant covariates, reduce statistical power especially in extreme class imbalance cases, and can even introduce bias in other covariates in the dataset [4].

A.1.2 Random Oversampling (ROS)

ROS attempts to rebalance covariate distributions by repeating the entries of randomly sampled observations of the minority class. The resulting cohort is a larger dataset that is comprised of repeated observations and well-represented covariate distributions [5]. While ROS has the advantage of improving the stability and performance of learning algorithms applied to the dataset by mitigating convergence issues, it is usually argued that the repeated observations barely add to the accuracy during statistical learning (e.g., classification). This is especially true when the minority class is not well-represented in the biased dataset. Another way of rationalizing the idea of oversampling is that we are assigning higher weights to the minority classes during statistical learning [6].

A.1.3 Synthetic Minority Oversampling Technique (SMOTE)

Sampling with replacement of the minority class of the imbalanced covariate often creates data cohorts that overfit during statistical learning [7]. Hence, an alternative approach to replicating instances of the minority class is to generate synthetic copies through interpolation among neighboring minority instances. This approach is termed SMOTE [8]. The approach is widely used by the data mining community as the standard pre-processing tool for imbalanced datasets.

Unlike ROS, the main appeal of SMOTE is that it adds new plausible observations that are sampled from the neighborhood of the minority class -- and not simply repeated entries. Since the seminal paper by Chawla et al, various modifications and advancements of SMOTE have seen proposed. A thorough review of the major advancements in SMOTE-based approaches to mitigate imbalanced datasets is presented in [9,10].

A.1.4 Propensity Score Matching

While global balancing might not always be possible, it is sometimes more feasible to balance the dataset within strata. Denote the set of disjoint strata making up X by $S = (S_1, S_2, \ldots, S_k)$. Suppose that X is independent of the binary predictor Z (0,1), we can say that the dataset is balanced if

$$f_{X|Z:S}(X=x|Z=1 : x \in S) = f_{X|Z:S}(X=x|Z=0 : x \in S). \qquad \text{(A1)}$$

As it turns out, this is feasible if S could be written in terms of the statistics $e(X)$ such that the conditional distribution $f_{X|Z,e(X)}(x/z,e)$ is functionally independent of z. That is, we have that $Z \perp\!\!\!\perp X | e(X)$. The term $e(X)$ can be expressed as the probability of being treated conditional on the individual's background (baseline) characteristics:

$$e(x) = \Pr[Z=1|x] = \text{expit}(x\beta), \qquad \text{(A2)}$$

where the function expit(t)=exp(t)/(1+exp(t)). Intuitively, the propensity score is a measure of the likelihood that an individual would have been treated based on his or her baseline characteristics. It is usually estimated using logistic regression with baseline characteristics as the predictor variables in the model.

Propensity score adjustment can take place in the form of matching, stratification, or regression (covariance) adjustment. While matching and stratification are usually applied to the dataset before statistical modeling to construct balance and appropriate comparisons among the baseline covariate, adjustment with propensity scores is employed at the analyses stage by including the scores (as weights) directly into the regression model.

In this manuscript, we restrict our evaluation to propensity score adjustment to rebalance the biased data during the analysis phase. This approach estimates the likelihood of an individual's group assignment (minority or majority group) based on the observed covariates, resulting in a propensity score. Subsequently, we match individuals with comparable propensity scores from minority and majority groups of the biasing covariate, effectively minimizing the influence of biased data.

A.2 Algorithmic approaches for mitigating covariate imbalance

Algorithmic methods mitigate the problem of covariate imbalance by targeting the learning stage of the analyses. It encompasses approaches that adapt commonly used learning algorithms to penalize models for learning from the minority class of the imbalanced data. These approaches can broadly be grouped into ensemble-based learning [11], cost-sensitive learning [12], and single-class learning [13]. We briefly describe each learning approach and refer readers to some comprehensive reviews.

A.2.1 Ensemble-based learning

In the data mining literature, ensemble learning refers to a group of methods that combine multiple base learners (or inducers) to make decisions. For biased dataset with imbalanced covariate distribution, the base learners are usually built on decision trees or neural network models due to their flexibility and robustness in handling complex datasets. The approach is often hailed for its ability to utilize the performance of multiple learners to improve overall performance. Dong et al. [14] provide an extensive survey of ensemble-based learning methods for dealing with complex datasets, including imbalanced covariates. Thus, the manuscript evaluates the performance of ensemble-based learning for mitigating the impact of bias in biased datasets.

A.2.2 Other algorithmic methods

Two other methods that we summarize here in this category but do not evaluate are: cost-sensitive learning [15–17] and single class learning.

The former takes the cost of misclassification (e.g., the error rate) into account during model training and is particularly well-suited for developing predictive models. That is, they are built on the idea of improving prediction accuracy rather than ensuring the stability or robustness of the estimated parameters. For example, CS-SVM algorithms allocate different costs for learning from the majority class versus learning from the minority class [18,19]. In a high imbalance binary covariate, this would imply assigning a high misclassification cost for the minority group while giving less weight to misclassifying the majority group. For a detailed survey of CS-L learning methods, see the reviews [12,20].

In the data mining literature, one-class learning is defined as a data modeling approach that attempts to construct an algorithm by learning primarily on a training set comprising only one class [21,22]. In some ways, single-class learning can be viewed as a stratified analysis of the data cohort involving only the minority or majority class [23,24]. For the most part, these algorithms focus on learning the data cohort of the majority class. However, some approaches have also been developed for well-structured cohorts of the minority class [13].

A.3 Post hoc approaches for imbalanced data

When the effect of data bias cannot be addressed during pre-processing (e.g., data cleaning) or the modeling stage, it is sometimes possible to employ post hoc adjustment techniques to account for the effect of learning from biased data. For example, in genetic studies of gene-based rare variant associations, bootstrap resampling approaches are often employed to adjust for bias in single-marker tests on common variants in GWAS studies [25]. In most cases, post hoc adjustments are only recommended as last-resort options when bias could not be mitigated in the early stages of the study [26].

Appendix B: Simulated Data

B.1 Data generation process

First, we consider a simulation setting involving a binary outcome Y, binary predictor variable Z sampled from the binomial(p=0.5), two binary variables X_1 and X_2 with the observations of X_2 given Z=1 sampled from the binomial(p=0.4) while the remaining observations of X2 given Z=0 were sampled from binomial(p=0.35). We also considered a relevant continuous predictor of the outcome U generated from the log-Normal(12,3.5) and independent of the other covariates. An effect modifier term Z*X2 for the interaction between Z and X_2 was also included. We postulate the logistic regression model:

$$P(Y=1) = \text{expit}(\alpha + \beta_Z Z + \beta_{X1} X_1 + \beta_{X2} X_2 + \beta_{ZX2} Z X_2 + \beta_U U), \qquad \text{(B1)}$$

where expit(t) = exp(t)/(1+exp(t)). For the set of fixed parameters {α=log(0.5), β_Z= log(1.25), β_{X1}= log(0.3), β_{X2}= log(2), β_{ZX2}=-0.47, β_U= log(0.5)}, we construct the full cohort data (the ground truth) before inducing bias in the sampled training cohort. The simulation is repeated to generate 500 datasets. A summary of the distributions of the simulated cohort stratified by the predictor variable categories in provided in the Table below.

	level	Stratified by Z		
		0	1	p test
n		2,521	2,479	
Y (mean (SD))		0.04 (0.19)	0.04 (0.21)	0.182
X1 (%)	0	1055 (41.8)	1014 (40.9)	0.516
	1	1466 (58.2)	1465 (59.1)	
X2 (%)	0	1746 (69.3)	1610 (64.9)	0.001
	1	775 (30.7)	869 (35.1)	
ZX2 (%)	0	2521 (100.0)	1610 (64.9)	<0.001
	1	0 (0.0)	869 (35.1)	
U (mean (SD))		3.40 (0.62)	3.43 (0.60)	0.080

Table S1: Full cohort data. Distributions of simulated observations stratified by the biased covariate variable Z=(0,1). Observations are simulated such that the binary covariate X1 is weakly/moderately correlated with the biased covariate Z while X2 is strongly correlated with the binary predictor. Z is balanced among the outcome categories.

We also present the distributions of the variables in the training cohort (sampled simulated example) and the distributions of the corresponding biased datasets (i.e., the marginal and conditionally biased datasets).

		level	Stratified by Z 0	1	p test
Training set	n		1,894	1,856	
	Y (mean (SD))		0.05 (0.21)	0.05 (0.21)	0.921
	X1 (%)	0	781 (41.2)	738 (39.8)	0.376
		1	1113 (58.8)	1118 (60.2)	
	X2 (%)	0	1311 (69.2)	1219 (65.7)	0.023
		1	583 (30.8)	637 (34.3)	
	ZX2 (%)	0	1894 (100.0)	1219 (65.7)	<0.001
		1	0 (0.0)	637 (34.3)	
	U (mean (SD))		3.41 (0.66)	3.43 (0.62)	0.316
Marginal bias	n		947	1,856	
	Y (mean (SD))		0.05 (0.22)	0.05 (0.21)	0.376
	X1 (%)	0	400 (42.2)	738 (39.8)	0.222
		1	547 (57.8)	1118 (60.2)	
	X2 (%)	0	641 (67.7)	1219 (65.7)	0.307
		1	306 (32.3)	637 (34.3)	
	ZX2 (%)	0	947 (100.0)	1219 (65.7)	<0.001
		1	0 (0.0)	637 (34.3)	
	U (mean (SD))		3.40 (0.69)	3.43 (0.62)	0.135
Conditional bias I	n		1,504	1,856	
	Y (mean (SD))		0.04 (0.20)	0.05 (0.21)	0.704
	X1 (%)	0	391 (26.0)	738 (39.8)	<0.001
		1	1113 (74.0)	1118 (60.2)	
	X2 (%)	0	1041 (69.2)	1219 (65.7)	0.033
		1	463 (30.8)	637 (34.3)	
	ZX2 (%)	0	1504 (100.0)	1219 (65.7)	<0.001
		1	0 (0.0)	637 (34.3)	
	U (mean (SD))		3.41 (0.66)	3.43 (0.62)	0.363
Conditional bias II	n		1,894	1,856	
	Y (mean (SD))		0.05 (0.21)	0.05 (0.21)	0.921
	X1 (%)	0	781 (41.2)	738 (39.8)	0.376
		1	1113 (58.8)	1118 (60.2)	
	X2 (%)	0	1311 (69.2)	1219 (65.7)	0.023
		1	583 (30.8)	637 (34.3)	
	ZX2 (%)	0	1894 (100.0)	1219 (65.7)	<0.001
		1	0 (0.0)	637 (34.3)	
	U (mean (SD))		3.41 (0.66)	3.43 (0.62)	0.316

Table S2: Distributed of training samples (original training set, marginal bias, and conditional bias) stratified by the biased covariate Z.

B.2 Additional results for simulated data

Missing	Approach	Marginal bias			Conditional bias I			Conditional bias II		
		AUC	OR_Z (SD)	I_Z	AUC	OR_Z (SD)	I_Z	AUC	OR_Z (SD)	I_Z
15%	Biased	0.72	1.30 (0.15)	-	0.72	1.30 (0.16)	-	0.72	1.30 (0.15)	-
	RUS	0.72	1.28 (0.15)	0.98	0.72	1.28 (0.16)	0.94	0.71	1.28 (0.15)	0.95
	ROS	0.72	1.27 (0.14)	0.98	0.72	1.29 (0.14)	0.93	0.72	1.27 (0.14)	0.94
	SMOTE	0.71	1.14 (1.18)	0.74	0.69	1.19 (1.18)	0.88	0.70	1.18 (1.04)	0.84
	PS-match	0.72	1.19 (0.16)	0.92	0.72	1.18 (0.16)	0.92	0.72	1.23 (0.16)	0.96
	RF	0.70	-	-	0.69	-	-	0.69	-	-
	SMA‡	0.72	1.25 (0.14)	0.98	0.72	1.27 (0.14)	0.95	0.72	1.26 (0.14)	0.95
30%	Biased	0.71	1.34 (0.17)	-	0.72	1.38 (0.15)	-	0.72	1.27 (0.11)	-
	RUS	0.72	1.36 (0.14)	0.97	0.71	1.36 (0.15)	0.87	0.71	1.26 (0.12)	0.89
	ROS	0.72	1.28 (0.14)	0.98	0.72	1.46 (0.17)	0.84	0.71	1.22 (0.11)	0.90
	SMOTE	0.70	0.88 (1.17)	0.34	0.70	1.02 (0.19)	0.60	0.70	0.90 (0.14)	0.24
	PS-match	0.71	1.13 (0.15)	0.84	0.71	1.41 (0.14)	0.94	0.71	1.37 (0.11)	0.71
	RF	0.69	-	-	0.69	-	-	0.69	-	-
	SMA‡	0.72	1.25 (0.14)	0.98	0.72	1.31 (0.15)	0.95	0.72	1.24 (0.07)	0.91
50%	Biased	0.71	1.49 (0.19)	-	0.72	1.31 (0.15)	-	0.72	1.43 (0.19)	-
	RUS	0.70	1.38 (0.14)	0.85	0.72	1.30 (0.14)	0.89	0.71	1.41 (0.20)	0.84
	ROS	0.71	1.45 (0.16)	0.78	0.72	1.29 (0.16)	0.90	0.71	1.51 (0.19)	0.82
	SMOTE	0.68	0.94 (0.21)	0.71	0.69	1.02 (0.21)	0.49	0.68	0.91 (0.18)	0.41
	PS-match	0.70	1.44 (0.26)	0.72	0.71	1.32 (0.13)	0.95	0.70	1.45 (0.24)	0.78
	RF	0.69	-	-	0.68	-	-	0.70	-	-
	SMA‡	0.71	1.24 (0.19)	0.93	0.71	1.29 (0.13)	0.90	0.72	1.39 (0.14)	0.88
80%	Biased	0.68	1.00 (0.25)	-	0.71	1.32 (0.19)	-	0.70	1.09 (0.22)	-
	RUS	0.68	1.17 (0.28)	0.97	0.71	1.41 (0.21)	0.72	0.70	1.03 (0.27)	0.76
	ROS	0.68	1.02 (0.24)	0.98	0.72	1.28 (0.24)	0.75	0.70	0.97 (0.23)	0.84
	SMOTE	0.67	0.73 (0.21)	0.34	0.69	1.05 (0.20)	0.60	0.70	0.90 (1.15)	0.33
	PS-match	0.65	0.50 (0.95)	0.84	0.70	1.54 (0.15)	0.81	0.69	1.08 (0.40)	0.66
	RF	0.66	-	-	0.68	-	-	0.69	-	-
	SMA‡	0.69	0.96 (0.51)	0.98	0.70	1.33 (0.24)	0.77	0.70	1.24 (0.16)	0.91
95%	Biased	0.64	1.00 (0.94)	-	0.60	1.02 (1.05)	-	0.61	1.00 (0.82)	-
	RUS	0.64	1.17 (0.73)	0.55	0.60	1.01 (1.03)	0.52	0.60	1.01 (0.67)	0.46
	ROS	0.65	1.00 (0.56)	0.60	0.62	1.04 (1.02)	0.55	0.60	0.86 (0.55)	0.49
	SMOTE	0.67	0.69 (1.74)	0.44	0.59	1.05 (1.21)	0.52	0.58	0.98 (1.64)	0.53
	PS-match	0.65	0.64 (1.68)	0.49	0.60	1.04 (0.99)	0.54	0.59	1.01 (0.63)	0.52
	RF	0.61	-	-	0.58	-	-	0.60	-	-
	SMA‡	0.65	0.97 (1.05)	0.68	0.60	1.03 (0.81)	0.67	0.61	1.12 (0.46)	0.78

NB: Synthetic Minor Augmentation (SMA)

Table S3: Simulations for the full model. Estimates of the mean AUC, mean Odds Ratio associated with Z and the standard deviation (OR_Z(SD)), and the confidence interval overlaps of the biased covariate effect with the ground truth (I_Z) from 500 repetitions. Missing prop indicates the proportion of observations removed under each bias setting. For the Original data: AUC = 0.72; OR_Z(SD) = 1.24(0.15); I_Z =1.00. The proportion ranges from 15% to 95%. ǂ indicates that the estimates are averaged from m=100 synthetic copies.

**Missing**	**15%**			**30%**			**50%**			**80%**			**95%**		
**Approach**	**MB**	**CB1**	**CBII**	**MB**	**CBI**	**CBII**	**MB**	**CBI**	**CBII**	**MB**	**CBI**	**CBII**	**MB**	**CBI**	**CBII**
Biased	0.70	0.70	0.70	0.70	0.70	0.70	0.69	0.69	0.70	0.67	0.67	0.68	0.50	0.50	0.51
RUS	0.70	0.70	0.70	0.70	0.70	0.70	0.70	0.70	0.70	0.60	0.60	0.61	0.49	0.49	0.52
ROS	0.70	0.70	0.70	0.70	0.70	0.70	0.70	0.70	0.71	0.68	0.68	0.68	0.49	0.49	0.54
SMOTE	0.69	0.69	0.69	0.69	0.69	0.69	0.69	0.69	0.69	0.69	0.69	0.70	0.50	0.50	0.50
PSM	0.71	0.71	0.70	0.71	0.71	0.70	0.70	0.70	0.70	0.62	0.62	0.65	0.50	0.50	0.50
RF	0.69	0.69	0.69	0.67	0.67	0.67	0.66	0.66	0.67	0.65	0.65	0.65	0.51	0.51	0.49
SMAǂ	0.70	0.70	0.70	0.70	0.70	0.70	0.70	0.70	0.70	0.68	0.68	0.67	0.50	0.50	0.50

Table S4: Simulations for the stratified model indicating the AUCs for predicting the minority group of the biased covariate under each bias mitigation method. The proportion of observations removed ranges from 15% to 95%. The reference estimates for the minority category of the original cohort (β_Z = log(1.25)) is provided: Minority category AUC = 0.71.

**Missing prop**	**15%**			**30%**			**50%**			**80%**			**95%**		
**Approach**	**MB**	**CB1**	**CBII**	**MB**	**MB**	**CBI**	**MB**	**CBI**	**CBII**	**MB**	**CBI**	**CBII**	**MB**	**CBI**	**CBII**
Biased data	0.04	0.04	0.04	0.04	0.04	0.04	0.04	0.04	0.04	0.04	0.04	0.04	0.04	0.04	0.04
RUS	0.04	0.04	0.04	0.04	0.04	0.04	0.04	0.04	0.04	0.04	0.04	0.04	0.04	0.04	0.04
ROS	0.04	0.04	0.04	0.04	0.04	0.04	0.04	0.04	0.04	0.04	0.04	0.04	0.04	0.04	0.04
SMOTE	0.04	0.04	0.04	0.04	0.04	0.04	0.04	0.04	0.04	0.05	0.04	0.04	0.04	0.05	0.05
PS-matching	0.04	0.04	0.04	0.04	0.04	0.04	0.04	0.04	0.04	0.04	0.04	0.04	0.04	0.04	0.04
RF ensemble	0.06	0.06	0.06	0.06	0.06	0.06	0.06	0.06	0.06	0.06	0.06	0.06	0.06	0.06	0.06
SMA	0.04	0.03	0.03	0.04	0.03	0.03	0.04	0.03	0.03	0.04	0.03	0.03	0.04	0.03	0.03

Table S5: Simulations for the stratified model indicating the Brier scores for predicting the minority group of the biased covariate under each bias mitigation method. The proportion of observations removed ranges from 15% to 95%. The reference estimates for the minority category of the original cohort (β_Z = log(1.25)) is provided: Minority category Brier Score = 0.03.

Fairness	Type	Missing	Biased	RUS	ROS	SMOTE	PSM	SMA‡
SPD Original = -0.045	Marginal	15%	-0.067	-0.037	-0.065	-0.187	0.069	-0.045
		30%	-0.092	-0.094	-0.098	-0.182	-0.020	-0.053
		50%	-0.051	-0.065	-0.059	-0.132	0.003	-0.024
		80%	-0.076	-0.117	-0.054	-0.091	0.611	0.017
		95%	0.018	0.289	0.066	0.031	0.358	0.092
	Conditional I	15%	-0.077	-0.053	-0.063	-0.123	0.003	-0.045
		30%	-0.071	-0.106	-0.050	-0.055	0.032	-0.053
		50%	-0.073	-0.067	-0.079	-0.072	0.075	-0.024
		80%	-0.102	-0.197	-0.083	-0.066	-0.062	0.017
		95%	-0.098	-0.109	-0.105	-0.162	0.551	0.092
	Conditional II	15%	-0.069	-0.086	-0.076	-0.136	0.035	-0.066
		30%	-0.068	-0.062	-0.048	-0.170	0.037	-0.066
		50%	-0.083	-0.101	-0.097	-0.106	0.013	-0.076
		80%	-0.100	-0.083	-0.092	-0.060	-0.012	-0.079
		95%	-0.095	-0.142	-0.096	-0.119	0.571	-0.046
EOD Original = -0.079	Marginal	15%	-0.081	-0.044	-0.077	-0.208	0.081	-0.054
		30%	-0.111	-0.101	-0.112	-0.201	-0.021	-0.064
		50%	-0.063	-0.071	-0.071	-0.151	0.004	-0.030
		80%	-0.093	-0.104	-0.057	-0.103	0.666	0.021
		95%	0.023	0.353	0.103	0.044	0.407	0.120
	Conditional I	15%	-0.092	-0.064	-0.074	-0.128	0.004	-0.054
		30%	-0.086	-0.114	-0.058	-0.058	0.040	-0.064
		50%	-0.088	-0.076	-0.092	-0.086	0.098	-0.030
		80%	-0.121	-0.197	-0.093	-0.067	-0.066	0.021
		95%	-0.117	-0.115	-0.118	-0.169	0.819	0.120
	Conditional II	15%	-0.083	-0.098	-0.087	-0.138	0.040	-0.080
		30%	-0.083	-0.068	-0.057	-0.171	0.047	-0.079
		50%	-0.100	-0.124	-0.113	-0.123	0.016	-0.092
		80%	-0.120	-0.098	-0.104	-0.067	-0.013	-0.096
		95%	-0.114	-0.149	-0.111	-0.127	0.822	-0.052
AOD Original = 0.071	Marginal	15%	0.073	0.040	0.070	0.196	0.075	0.049
		30%	0.100	0.097	0.104	0.191	0.020	0.058
		50%	0.056	0.068	0.064	0.141	0.004	0.026
		80%	0.084	0.111	0.055	0.096	0.637	0.019
		95%	0.020	0.317	0.082	0.037	0.382	0.104
		15%	0.084	0.058	0.068	0.125	0.003	0.049

	Conditional I	30%	0.077	0.110	0.054	0.057	0.036	0.058
		50%	0.080	0.071	0.085	0.078	0.086	0.026
		80%	0.110	0.197	0.087	0.066	0.064	0.019
		95%	0.106	0.111	0.111	0.165	0.679	0.104
	Conditional II	15%	0.075	0.091	0.081	0.137	0.038	0.072
		30%	0.075	0.065	0.052	0.171	0.042	0.072
		50%	0.091	0.112	0.104	0.113	0.014	0.083
		80%	0.109	0.090	0.098	0.063	0.013	0.087
		95%	0.103	0.145	0.102	0.123	0.691	0.048

Table S6. Estimates of group fairness based on statistical parity difference (SPD), equal opportunity difference (EOD), and average odds difference (AOD). The estimates reported for each metric represent average values computed over 500 simulated samples. Fairness estimates shown for the original data (ground truth) are considered as the benchmark. ǂ indicates that the estimates are averaged from m=100 synthetic copies. PSM is Propensity Score matching.

B.3 Sensitivity analyses results

In this section, we provide additional sensitivity analysis results for estimating the mean Odds ratio of the predictor Z. We conducted 500 repetitions with various effect sizes β_Z =(log(0.5), log(1.01), log(2)), and considered missing proportions ranging from 30% to 80%. All estimates using the Simple Moving Average (SMA) technique were aggregated from a total of m=100 synthetic data samples.

Missing prop	Approach	β_2 = log (0.5)			β_2 = log (1.01)			β_2 = log (2.0)		
		MB	CB1	CBII	MB	CB1	CBII	MB	CB1	CBII
15%	Biased data	0.50	0.48	0.47	1.04	1.07	0.88	1.99	1.90	2.26
	RUS	0.48	0.38	0.55	0.99	1.06	0.91	1.95	2.05	1.90
	ROS	0.47	0.42	0.48	1.05	0.99	0.97	2.31	2.02	2.11
	SMOTE	0.44	0.29	0.34	1.02	0.95	1.14	1.32	1.74	1.98
	PS-	0.56	0.49	0.58	1.00	0.99	1.32	1.97	2.30	2.23
	RF	-	-	-	-	-	-	-	-	-
	SMA	0.50	0.49	0.51	1.03	0.97	0.95	1.99	2.08	1.94
30%	Biased data	0.51	0.47	0.34	1.01	0.95	1.11	2.06	1.82	1.75
	RUS	0.37	0.48	0.38	0.92	0.97	1.10	2.03	1.77	1.65
	ROS	0.57	0.46	0.31	0.85	1.00	1.15	2.03	1.78	1.87
	SMOTE	0.37	0.43	0.30	0.71	0.75	0.73	1.66	1.21	1.50
	PS-	0.57	0.49	0.35	1.01	0.89	1.33	2.20	1.91	1.51
	RF	-	-	-	-	-	-	-	-	-
	SMA	0.51	0.48	0.33	1.01	0.99	1.11	2.13	1.83	1.87
50%	Biased data	0.44	0.51	0.39	1.01	0.97	1.46	1.88	2.14	1.75
	RUS	0.33	0.54	0.40	0.96	0.98	1.40	1.80	1.93	1.82
	ROS	0.43	0.51	0.39	0.75	0.93	1.09	1.83	2.15	1.89
	SMOTE	0.49	0.46	0.26	0.53	0.76	0.93	1.84	1.84	1.36
	PS-	0.36	0.54	0.37	0.95	1.00	1.32	2.02	2.39	1.69
	RF	-	-	-	-	-	-	-	-	-
	SMA	0.51	0.49	0.43	1.01	0.85	1.58	1.88	2.36	1.63
80%	Biased data	0.41	0.48	0.53	0.94	1.01	1.16	1.98	1.59	1.65
	RUS	0.39	0.46	0.55	0.72	0.89	0.95	2.06	1.69	1.92
	ROS	0.42	0.51	0.42	0.80	0.91	1.09	2.04	1.56	1.92
	SMOTE	0.42	0.45	0.23	0.64	0.74	0.83	1.78	1.72	1.13
	PS-	0.39	0.53	0.57	1.34	1.02	0.97	2.70	1.72	1.37
	RF	-	-	-	-	-	-	-	-	-
	SMA	0.47	0.52	0.82	0.98	0.89	1.01	1.57	1.32	1.21
95%	Biased data	0.40	0.38	0.37	1.14	1.07	0.78	1.79	1.50	2.36
	RUS	0.38	0.33	0.71	0.79	1.06	0.91	1.85	2.55	1.50
	ROS	0.37	0.37	0.33	1.20	0.95	0.77	2.31	2.42	2.21
	SMOTE	0.34	0.21	0.24	1.12	0.99	0.49	1.32	1.74	1.98
	PS-	0.61	0.44	0.78	1.15	0.65	1.54	1.79	1.30	2.43
	RF	-	-	-	-	-	-	-	-	-
	SMA	0.45	0.36	0.41	1.11	0.87	0.75	1.59	2.38	1.19

Table S7: Estimates of the Odds Ratio of Z of the primary predictor Z from 500 repetitions. For the ground truth, the fixed values of β_Z = (log(0.5), log(1.01), log(2)). The proportion of observations removed ranges from 15% to 95%. The ground truth OR estimates for Z = (log(0.5), log(1.01), log(2)) were 0.50, 1.01, and 1.99, respectively.

Missing prop	Approach	β_Z = log(0.5)			β_Z = log(1.01)			β_Z = log(2.0)		
		MB	CB1	CBII	MB	CBI	CBII	MB	CBI	CBII
15%	Biased data	0.70	0.70	0.70	0.70	0.70	0.70	0.70	0.70	0.70
	RUS	0.69	0.70	0.70	0.70	0.70	0.70	0.70	0.70	0.70
	ROS	0.70	0.70	0.71	0.70	0.70	0.70	0.70	0.70	0.70
	SMOTE	0.71	0.69	0.69	0.70	0.71	0.71	0.69	0.69	0.69
	PS-matching	0.71	0.70	0.70	0.69	0.69	0.69	0.71	0.70	0.70
	RF ensemble	0.66	0.67	0.67	0.70	0.70	0.70	0.69	0.69	0.68
	SMA	0.70	0.69	0.71	0.70	0.70	0.71	0.70	0.70	0.71
30%	Biased data	0.70	0.69	0.69	0.70	0.70	0.70	0.70	0.70	0.70
	RUS	0.69	0.70	0.70	0.70	0.70	0.70	0.70	0.70	0.70
	ROS	0.70	0.70	0.70	0.70	0.70	0.70	0.70	0.70	0.70
	SMOTE	0.71	0.69	0.69	0.69	0.69	0.69	0.69	0.69	0.69
	PS-matching	0.71	0.70	0.70	0.71	0.70	0.70	0.71	0.70	0.70
	RF ensemble	0.66	0.67	0.67	0.67	0.67	0.67	0.67	0.67	0.67
	SMA	0.70	0.69	0.71	0.70	0.70	0.70	0.70	0.70	0.71
50%	Biased data	0.69	0.69	0.69	0.70	0.70	0.70	0.69	0.70	0.70
	RUS	0.70	0.70	0.70	0.70	0.70	0.70	0.70	0.70	0.70
	ROS	0.70	0.71	0.71	0.70	0.71	0.71	0.70	0.71	0.71
	SMOTE	0.69	0.69	0.69	0.69	0.69	0.69	0.69	0.69	0.69
	PS-matching	0.70	0.70	0.70	0.70	0.70	0.70	0.70	0.70	0.70
	RF ensemble	0.66	0.67	0.67	0.66	0.67	0.67	0.66	0.67	0.67
	SMA	0.70	0.70	0.70	0.70	0.70	0.70	0.70	0.70	0.70
80%	Biased data	0.66	0.66	0.65	0.69	0.69	0.68	0.67	0.68	0.67
	RUS	0.60	0.61	0.60	0.60	0.61	0.58	0.60	0.61	0.58
	ROS	0.67	0.67	0.66	0.68	0.68	0.67	0.68	0.68	0.67
	SMOTE	0.69	0.65	0.69	0.69	0.70	0.69	0.69	0.70	0.69
	PS-matching	0.60	0.63	0.68	0.62	0.65	0.70	0.62	0.65	0.70
	RF ensemble	0.65	0.65	0.65	0.65	0.65	0.65	0.65	0.65	0.65
	SMA	0.66	0.67	0.65	0.68	0.67	0.68	0.68	0.67	0.65
95%	Biased data	0.61	0.61	0.53	0.50	0.50	0.52	0.50	0.51	0.51
	RUS	0.59	0.60	0.56	0.50	0.50	0.55	0.49	0.52	0.48
	ROS	0.62	0.54	0.63	0.51	0.49	0.53	0.49	0.54	0.52
	SMOTE	0.61	0.60	0.65	0.50	0.50	0.51	0.50	0.50	0.52
	PS-matching	0.60	0.57	0.59	0.50	0.50	0.52	0.50	0.50	0.55
	RF ensemble	0.59	0.56	0.55	0.50	0.50	0.55	0.51	0.49	0.53
	SMA	0.60	0.59	0.56	0.51	0.51	0.53	0.50	0.50	0.51

Table S8: Simulations for the stratified model. AUCs for predicting the minority group of the biased covariate under each bias mitigation method are shown. The proportion of observations removed ranges from 15% to 95%. The ground truth AUC estimates for Z = (log(0.5), log(1.01), log(2)) were 0.72, 0.71, and 0.72, respectively.

Appendix C: Real Datasets

This section presents a detailed description of four case studies and how data bias (marginal and conditional) is induced. While the overall summaries of the results are included in the main manuscript, the evaluations of the individual datasets have been relegated to the appendix for brevity.

C.1 Colon Cancer N0147 trial

N0147 was a Colon Cancer trial conducted between 2004 and 2009 that assigned patients with ``stage III colon cancer to adjuvant regimens of folinic acid, fluorouracil, and oxaliplatin or fluorouracil, leucovorin, and irinotecan, with or without cetuximab." Patient-level data from the control arms (chemotherapy-only) were obtained which comprised 1543 observations and 10 variables. Our analysis considered death as the outcome variable while the biased covariate of interest was bowel obstruction. The remaining predictors were all included in the postulated model except the time variable.

In the marginal bias setting, observations belonging to one arm of bowel obstruction (e.g., no bowel obstruction) were randomly sampled and removed from the training data, independent of other covariates. For the first conditional bias case, gender (which is weakly associated with bowel obstruction, $\beta = 0.167$, p-value = 0.1968) is conditional on the type I case. Thus, observations were randomly sampled and excluded from the training set if they were female and had no bowel obstruction. In the second conditional bias case, we used bmi (which is strongly associated with bowel obstruction, $\beta = 1.092$, p-value < 0.001) as the conditioning variable to induce bias in the training data. For marginal bias, observations that had no bowel obstruction were excluded from the training data, independent of other covariates in the dataset.

The amount of sampling bias was also varied by increasing the proportion of samples (sampled observations) removed under each biasing scheme. In the marginal bias case, for example, this amounts to increasing the proportion of observations removed in the minority class from low to high. In the conditional bias cases, the proportion of observations removed from each conditional case is increased from low to high.

C.2 Danish Colorectal Cancer data (DCCG)

The Danish Colorectal Cancer Group (DCCG) database is a prospectively maintained dataset of all danish patients with a first-time diagnosis of right-sided colonic cancer between 2001 and 2018. The dataset is comprised of 12855 observations and 192 attributes. Our analyses included 9 relevant predictors of "post medication complications" based on previous studies.

In the marginal bias setting, observations were randomly sampled and removed from the training set if they were female irrespective of the other covariates. We next considered two conditional bias settings by conditioning on covariates that were weakly or strongly associated with gender. In the first instance, female participants were only excluded from the training set if they were in the first category of P-PN stadium. In the second conditional case, observations were removed from the training set if they were female and had ASA. The amount of sampling

bias was also varied by increasing the proportion of samples (sampled observations) removed under each biasing scheme.

C.3 Breast Cancer (UCI)

This UCI data is provided by the Oncology Institute to predict the breast cancer [27]. It is comprised of 277 observations and 10 attributes. Our analyses included all predictors based on previous studies.

In the marginal bias case, observations were randomly sampled and removed from the training set if they belonged to the 20-49 group age category, irrespective of the other covariates. We considered two conditional bias settings: In the first conditional bias case, 20–49-year-old participants were only excluded from the training set if they the breast category was left. In the second conditional case, observations were removed from the biased covariate category (20-49 years) if they had menopause. The amount of sampling bias was also varied by increasing the proportion of samples (sampled observations) removed under each biasing scheme.

C.4 Cardiovascular Health data from CCHS-2014

For the cardiovascular health dataset, the outcome considered was the binary variable for cardiovascular health status and the biased covariate of interest was gender. The model included other relevant predictors (age, education, household income, household size, and whether the participant is a new immigrant or not) which were selected based on previous studies [28,29].

In the marginal bias case, observations were randomly sampled and removed from the training set if they were female irrespective of their other covariate values (or conversely, the same could be applied to male participants). We next considered two conditional bias settings by conditioning on covariates that were weakly or strongly associated with gender. In the first instance, female participants were only excluded from the training set if they were not new immigrants (new immigration is weakly associated with gender, p-value 0.294). In the second case, observations were removed from the training set if they were female and belonged to a specific marital category (e.g., marital status = 1). Marital status was selected as it is strongly associated with gender.

The amount of sampling bias was also varied by increasing the proportion of samples (sampled observations) removed under each biasing scheme. In the marginal bias case, for example, this amounts to increasing the proportion of observations removed in the minority class from low to high. In the conditional bias cases, the proportion of observations removed from each conditional case is increased from low to high.

Appendix D: Results for real datasets

D.1 Colon cancer N0147 trial

Missing prop	Approach	Marginal bias			Conditional bias I			Conditional bias II		
		AUC	OR_Z (SE)	I_Z	AUC	OR_Z (SE)	I_Z	AUC	OR_Z (SE)	I_Z
15%	Biased data	0.69	0.59(-)	-	0.70	0.56(-)	-	0.69	0.52(-)	-
	RUS	0.67	0.66(0.36)	0.82	0.70	0.49(0.35)	0.86	0.66	0.74(0.32)	0.71
	ROS	0.69	0.65(0.14)	0.86	0.65	0.55(0.14)	0.80	0.66	0.51(0.14)	0.76
	SMOTE	0.68	0.35(0.09)	0.33	0.69	0.55 (0.23)	0.98	0.65	0.30(0.23)	0.31
	PS-matching	0.68	1.03(0.66)	0.58	0.65	1.15(0.62)	0.57	0.65	1.13(0.61)	0.58
	RF ensemble	0.67	-	-	0.66	-	-	0.66	-	-
	SMA	0.70	0.58(0.23)	0.94	0.70	0.58(0.23)	0.94	0.70	0.53(0.23)	0.93
30%	Biased data	0.69	0.51(-)	-	0.70	0.52(-)	-	0.69	0.52(-)	-
	RUS	0.66	0.44(0.52)	0.82	0.68	0.54(0.35)	0.82	0.69	0.44(0.52)	0.82
	ROS	0.68	0.85(0.14)	0.78	0.67	0.49(0.14)	0.76	0.66	0.85(0.14)	0.78
	SMOTE	0.68	0.29(0.09)	0.00	0.66	0.29(0.09)	0.00	0.67	0.19(0.27)	0.00
	PS-matching	0.71	1.28(0.80)	0.54	0.60	1.28(0.80)	0.54	0.60	1.43(0.67)	0.52
	RF ensemble	0.66	-	-	0.65	-	-	0.65	-	-
	SMA	0.70	0.58(0.23)	0.94	0.70	0.58(0.24)	0.93	0.70	0.54(0.23)	0.98
50%	Biased data	0.69	0.62(-)	-	0.70	0.49(-)	-	0.70	0.50(-)	-
	RUS	0.67	0.57(0.52)	0.69	0.66	0.52(0.34)	0.81	0.65	0.40(0.41)	0.87
	ROS	0.67	0.85(0.15)	0.46	0.67	0.51(0.14)	0.77	0.71	0.66(0.14)	0.86
	SMOTE	0.66	0.38(0.11)	0.45	0.69	0.51(0.24)	0.90	0.66	0.63(0.24)	0.84
	PS-matching	0.63	2.48(1.08)	0.51	0.69	1.02(0.77)	0.56	0.62	1.64(0.89)	0.53
	RF ensemble	0.64	-	-	0.64	-	-	0.63	-	-
	SMA	0.70	0.59(0.24)	0.86	0.70	0.61(0.25)	0.88	0.70	0.53(0.24)	0.97
80%	Biased data	0.67	0.78(-)	-	0.70	0.49(-)	-	0.69	0.50(-)	-
	RUS	0.67	3.06(0.80)	0.24	0.71	0.68(0.42)	0.71	0.67	0.62(0.42)	0.72
	ROS	0.70	1.22(0.17)	0.01	0.68	0.55(0.14)	0.81	0.68	0.52(0.14)	0.79
	SMOTE	0.72	1.11(0.20)	0.19	0.68	0.49(0.26)	0.89	0.64	0.60(0.28)	0.87
	PS-matching	0.67	2.59(1.24)	0.50	0.58	1.99(0.78)	0.51	0.55	2.42(1.24)	0.51
	RF ensemble	0.63	-	-	0.60	-	-	0.56	-	-
	SMA	0.68	1.17(0.96)	0.06	0.67	1.69(0.52)	0.00	0.70	0.55(0.26)	0.94

Table S9: Estimates of mean AUC, Odds Ratio of Z [OR_Z(SE)], and interval overlaps (I_Z) of the biased covariate Z for each bias mitigating approach on the Colon Cancer data. For the Original data: AUC = 0.70; OR_Z(SE) = 0.55 (0.23); I_Z =1.00. Note. Synthetic Minor Augmentation (SMA).

Colon Cancer **Missing Prop**	**Approach**	**Marginal bias**			**Conditional bias I**			**Conditional bias II**		
		**SPD**	**EOD**	**AOD**	**SPD**	**EOD**	**AOD**	**SPD**	**EOD**	**AOD**
15%	Biased	-0.067	-0.081	0.073	-0.077	-0.092	0.084	-0.069	-0.083	0.075
	RUS	-0.037	-0.044	0.040	-0.053	-0.064	0.058	-0.086	-0.098	0.091
	ROS	-0.065	-0.077	0.070	-0.063	-0.074	0.068	-0.076	-0.087	0.081
	PS-matching	0.069	0.081	0.075	0.003	0.004	0.003	0.035	0.040	0.038
	SMOTE	-0.187	-0.208	0.196	-0.123	-0.128	0.125	-0.136	-0.138	0.137
	SMA	-0.045	-0.054	0.049	-0.045	-0.054	0.049	-0.066	-0.080	0.072
30%	Biased	-0.092	-0.111	0.100	-0.071	-0.086	0.077	-0.068	-0.083	0.075
	RUS	-0.094	-0.101	0.097	-0.106	-0.114	0.110	-0.062	-0.068	0.065
	ROS	-0.098	-0.112	0.104	-0.050	-0.058	0.054	-0.048	-0.057	0.052
	PS-matching	-0.020	-0.021	0.020	0.032	0.040	0.036	0.037	0.047	0.042
	SMOTE	-0.182	-0.201	0.191	-0.055	-0.058	0.057	-0.170	-0.171	0.171
	SMA	-0.053	0.079	0.058	-0.053	-0.064	0.058	-0.066	-0.079	0.072
50%	Biased	-0.051	-0.063	0.056	-0.073	-0.088	0.080	-0.083	-0.100	0.091
	RUS	-0.065	-0.071	0.068	-0.067	-0.076	0.071	-0.101	-0.124	0.112
	ROS	-0.059	-0.071	0.064	-0.079	-0.092	0.085	-0.097	-0.113	0.104
	PS-matching	0.003	0.004	0.004	0.075	0.098	0.086	0.013	0.016	0.014
	SMOTE	-0.132	-0.151	0.141	-0.072	-0.086	0.078	-0.106	-0.123	0.113
	SMA	-0.024	-0.030	0.026	-0.024	-0.030	0.026	-0.076	-0.092	0.083
	Biased	-0.076	-0.093	0.084	-0.102	-0.121	0.110	-0.100	-0.120	0.109
	RUS	-0.117	-0.104	0.111	-0.197	-0.197	0.197	-0.083	-0.098	0.090
	ROS	-0.054	-0.057	0.055	-0.083	-0.093	0.087	-0.092	-0.104	0.098
	PS-matching	0.611	0.666	0.637	-0.062	-0.066	0.064	-0.012	-0.013	0.013
	SMOTE	-0.091	-0.103	0.096	-0.066	-0.067	0.066	-0.060	-0.067	0.063
80%	SMA	0.017	0.021	0.019	0.017	0.021	0.019	-0.079	-0.096	0.087

Table S10: Fairness metrics: the statistical parity difference (SPD), equal opportunity difference (EOD), and average odds difference (AOD) from the logistic regression model on the colon cancer data. The proportion of samples removed varied from 15% to 80%. Original cohort: SPD = -0.066, EOD = -0.079, and AOD = 0.071

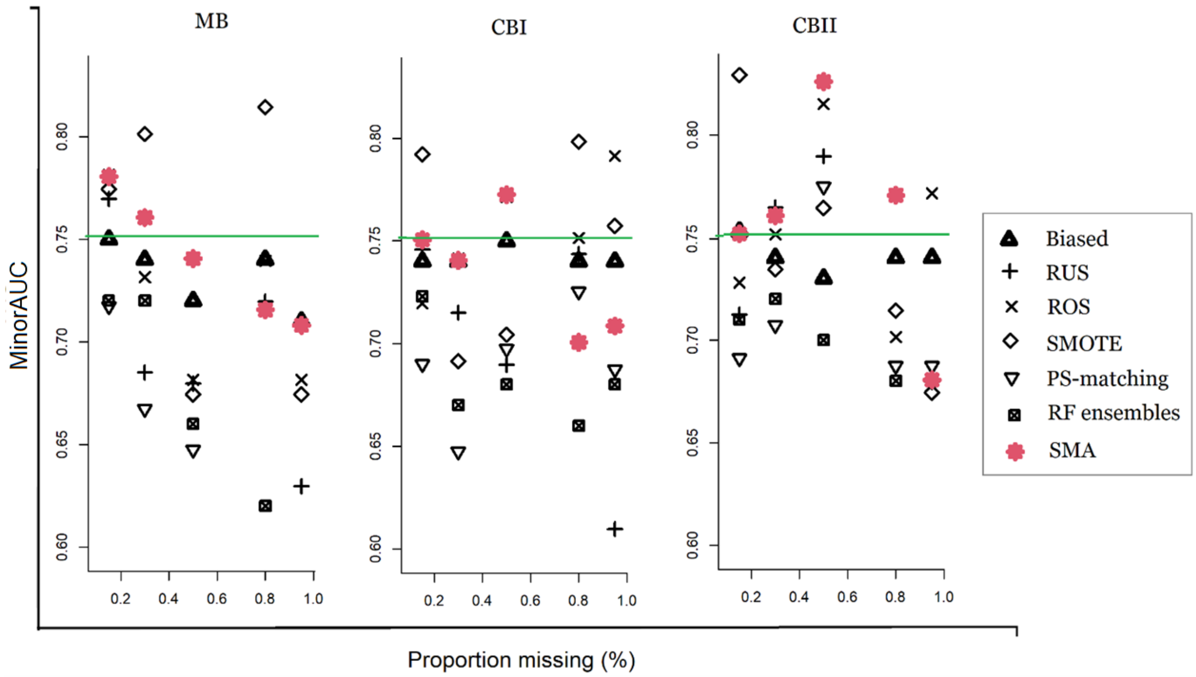

Figure S1: Colon Cancer data used for the stratified model. Plots of AUC estimates of the minor categories of the biased covariate. MB denotes marginal bias, CBI for conditional bias I, and CBII for conditional bias II. The horizontal line is the ground truth estimate (AUC =0.76).

Missing	***15%***			***30%***			***50%***			***80%***			***95%***		
**Approach**	**MB**	**CB1**	**CBII**	**MB**	**MB**	**CBI**	**MB**	**CBI**	**CBII**	**MB**	**CBI**	**CBII**	**MB**	**CBI**	**CBII**
Biased data	0.04	0.04	0.04	0.04	0.04	0.04	0.04	0.04	0.04	0.04	0.04	0.04	0.04	0.04	0.04
RUS	0.04	0.04	0.04	0.04	0.04	0.04	0.04	0.04	0.04	0.04	0.04	0.04	0.04	0.04	0.04
ROS	0.04	0.04	0.04	0.04	0.04	0.04	0.04	0.04	0.04	0.04	0.04	0.04	0.04	0.04	0.04
SMOTE	0.05	0.05	0.05	0.05	0.07	0.06	0.05	0.05	0.05	0.05	0.04	0.06	0.05	0.04	0.04
PS-	0.04	0.04	0.03	0.03	0.04	0.03	0.04	0.04	0.03	0.04	0.03	0.03	0.04	0.04	0.03
RF	0.06	0.06	0.06	0.06	0.06	0.06	0.06	0.06	0.06	0.06	0.06	0.05	0.05	0.05	0.06
SMA	0.03	0.03	0.04	0.03	0.03	0.03	0.03	0.03	0.03	0.04	0.04	0.04	0.04	0.04	0.04

Table S11: Brier scores for predicting the minority group of the biased covariate under each bias mitigation method. The proportion of observations removed ranges from 15% to 95%. The reference value for the brier score of the minority group of the original cohort for the Colon Cancer N0147 trial data was 0.03. MB-CBII denotes marginal bias, conditional bias I, and conditional bias II, respectively.

D.2 Danish colorectal cancer data

DCCG	Approach	Marginal bias			Conditional bias I			Conditional bias II		
		**AUC**	**OR_Z (SE)**	**I_Z**	**AUC**	**OR_Z (SE)**	**I_Z**	**AUC**	**OR_Z (SE)**	**I_Z**
Missing prop = 15%	Biased data	0.71	1.50(-)	-	0.71	1.41(-)	-	0.71	1.50(-)	-
	RUS	0.71	1.59(0.11)	0.83	0.71	1.40(0.10)	0.84	0.71	1.53(0.10)	0.93
	ROS	0.71	1.49(0.10)	0.97	0.71	1.41(0.10)	0.87	0.72	1.49(0.09)	0.98
	SMOTE	0.54	1.14(0.05)	0.00	0.67	3.67(0.06)	0.00	0.67	3.48(0.06)	0.00
	PS-matching	0.70	1.38(0.11)	0.84	0.70	1.40(0.10)	0.85	0.71	1.42(0.10)	0.88
	RF ensemble	0.69	-	-	0.69	-	-	0.66	-	-
	SMA	0.72	1.48(0.10)	0.97	0.72	1.44(0.10)	0.94	0.72	1.49(0.09)	0.99
Missing prop = 30%	Biased data	0.71	1.51(-)	-	0.71	1.41(-)	-	0.71	1.51(0.10)	-
	RUS	0.71	1.59(0.11)	0.83	0.71	1.40(0.10)	0.85	0.71	1.53(0.10)	0.94
	ROS	0.71	1.49(0.10)	0.97	0.71	1.41(0.10)	0.87	0.72	1.49(0.09)	0.98
	SMOTE	0.53	2.20(0.02)	0.00	0.67	3.67(0.06)	0.00	0.67	3.48(0.06)	0.00
	PS-matching	0.70	1.38(0.11)	0.84	0.70	1.40(0.10)	0.85	0.71	1.42(0.10)	0.88
	RF ensemble	0.69	-	-	0.69	-	-	0.66	-	-
	SMA	0.72	1.48(0.10)	0.97	0.72	1.44(0.10)	0.94	0.71	1.49(0.09)	0.99
Missing prop = 50%	Biased data	0.71	1.55(-)	-	0.72	1.50(0.11)	-	0.71	1.45(-)	-
	RUS	0.71	1.53(0.13)	0.86	0.72	1.56(0.11)	0.87	0.72	1.53(0.10)	0.93
	ROS	0.70	1.56(0.10)	0.87	0.72	1.53(0.10)	0.91	0.71	1.52(0.12)	0.91
	SMOTE	0.54	0.26(0.03)	0.00	0.59	0.41(0.06)	0.00	0.66	4.10(0.06)	0.00
	PS-matching	0.70	1.58(0.15)	0.80	0.71	1.49(0.12)	0.90	0.71	1.40(0.14)	0.85
	RF ensemble	0.69	-	-	0.68	-	-	0.65	-	-
	SMA	0.70	1.53(0.10)	0.90	0.70	1.54(0.10)	0.91	0.71	1.49(0.10)	0.95
Missing prop = 80%	Biased data	0.71	1.44(-)	-	0.71	1.53(-)	-	0.71	1.49(-)	-
	RUS	0.71	1.64(0.20)	0.72	0.71	1.70(0.14)	0.72	0.72	1.46(0.10)	0.95
	ROS	0.71	1.39(0.10)	0.82	0.72	1.55(0.11)	0.89	0.71	1.48(0.10)	0.98
	SMOTE	0.53	0.27(0.04)	0.00	0.60	0.47(0.08)	0.00	0.66	3.58(0.06)	0.00
	PS-matching	0.69	1.75(0.29)	0.63	0.70	1.70(0.21)	0.69	0.71	1.45(0.10)	0.93
	RF ensemble	0.68	-	-	0.68	-	-	0.66	-	-
	SMA	0.68	1.62(0.15)	0.81	0.68	1.61(0.10)	0.83	0.71	1.51(0.09)	0.91

Table S12: Danish Colorectal Cancer data. Estimates of mean AUC, Odds Ratio of Z [OR_Z(SE)], and interval overlaps (I_Z) of the biased covariate Z for each bias mitigating approach. For the Original data: AUC = 0.72; OR_Z(SE) = 1.49 (0.09); I_Z =1.00.

		Marginal bias			Conditional bias I			Conditional bias II		
		SPD	EOD	AOD	SPD	EOD	AOD	SPD	EOD	AOD
15%	Original	0.03468991	0.04381693	0.03846661	0.03468991	0.04381693	0.03846661	0.03468991	0.04381693	0.03846661
	Biased	0.03767215	0.04733844	0.04167199	0.03373957	0.04275114	0.03746850	0.03512007	0.04442725	0.03897132
	RUS	0.03867908	0.04887051	0.04289622	0.03344996	0.04229745	0.03711099	0.03767345	0.04816579	0.04201511
	ROS	0.03687484	0.04608132	0.04068442	0.03161668	0.03988646	0.03503866	0.03715609	0.04770401	0.04152074
	PS-matching	0.03677701	0.04627522	0.04070730	0.03334411	0.04236484	0.03707683	0.03491752	0.04433677	0.03881514
	SMOTE	0.17227266	0.19202503	0.18044605	0.05116598	0.06426332	0.05658557	0.04165021	0.05138717	0.04567930
	SMA	0.03992645	0.05056718	0.04432951	0.03992645	0.05056718	0.04432951	0.03538592	0.04494336	0.03934072
		SPD	EOD	AOD	SPD	EOD	AOD	SPD	EOD	AOD
30%	Original	0.03468991	0.04381693	0.03846661	0.03468991	0.04381693	0.03846661	0.03468991	0.04381693	0.03846661
	Biased	0.03203714	0.04030872	0.03545986	0.03481905	0.04384714	0.03855481	0.03498329	0.04417260	0.03878577
	RUS	0.03128850	0.03966488	0.03475459	0.03366507	0.04236279	0.03726413	0.03919482	0.04985141	0.04360445
	ROS	0.03177978	0.04011701	0.03522967	0.03444180	0.04336943	0.03813599	0.03743532	0.04720489	0.04147790
	PS-matching	0.02206079	0.02755445	0.02433403	0.03435630	0.04328908	0.03805262	0.03724712	0.04733350	0.04142080
	SMOTE	-0.10321145	-0.11510958	0.10813481	0.07077568	0.08715794	0.07755454	0.04244586	0.05431576	0.04735754
	SMA	0.03726544	0.04709545	0.04133303	0.03726544	0.04709545	0.04133303	0.03723513	0.04728223	0.04139255
		SPD	EOD	AOD	SPD	EOD	AOD	SPD	EOD	AOD
50%	Original	0.03468991	0.04381693	0.03846661	0.03468991	0.04381693	0.03846661	0.03468991	0.04381693	0.03846661
	Biased	0.03845515	0.04887808	0.04276809	0.03940540	0.04999730	0.04378825	0.03415492	0.04310667	0.03785909
	RUS	0.03657366	0.04634529	0.04061710	0.04181364	0.05390640	0.04681754	0.03315650	0.04162578	0.03666103
	ROS	0.04088668	0.05180323	0.04540387	0.04175157	0.05306473	0.04643288	0.03171702	0.04060627	0.03539533
	PS-matching	0.02616516	0.03313336	0.02904855	0.04096670	0.05189513	0.04548881	0.03283309	0.04152021	0.03642776
	SMOTE	-0.10770606	-0.12434163	0.11458975	0.01041892	0.01291407	0.01145140	0.03622737	0.04423755	0.03954193
	SMA	0.03883736	0.04988791	0.04341000	0.03883736	0.04988791	0.04341000	0.03365625	0.04270241	0.03739949
		SPD	EOD	AOD	SPD	EOD	AOD	SPD	EOD	AOD
80%	Original	0.03468991	0.04381693	0.03846661	0.03468991	0.04381693	0.03846661	0.03468991	0.04381693	0.03846661
	Biased	0.04247477	0.05394048	0.04721921	0.04048667	0.05144015	0.04501915	0.03341556	0.04196684	0.03695402
	RUS	0.03157968	0.04077064	0.03538283	0.03924626	0.04972774	0.04358343	0.03386296	0.04250255	0.03743796
	ROS	0.04372780	0.05599645	0.04880448	0.03897504	0.04882099	0.04304923	0.03400812	0.04291132	0.03769220
	PS-matching	0.01331130	0.01633023	0.01456051	0.05925348	0.07499481	0.06576714	0.03225525	0.04056767	0.03569487
	SMOTE	-0.10802334	-0.11898754	0.11256025	0.04887621	0.05969831	0.05335432	0.04344487	0.05363942	0.04766330
	SMA	0.05119520	0.06634382	0.05746359	0.05119520	0.06634382	0.05746359	0.03214566	0.04082427	0.03573681

Table S13: Measure of fairness. Estimates of the statistical parity difference (SPD), equal opportunity difference (EOD), and average odds difference (AOD) on the Danish Colorectal Cancer data.

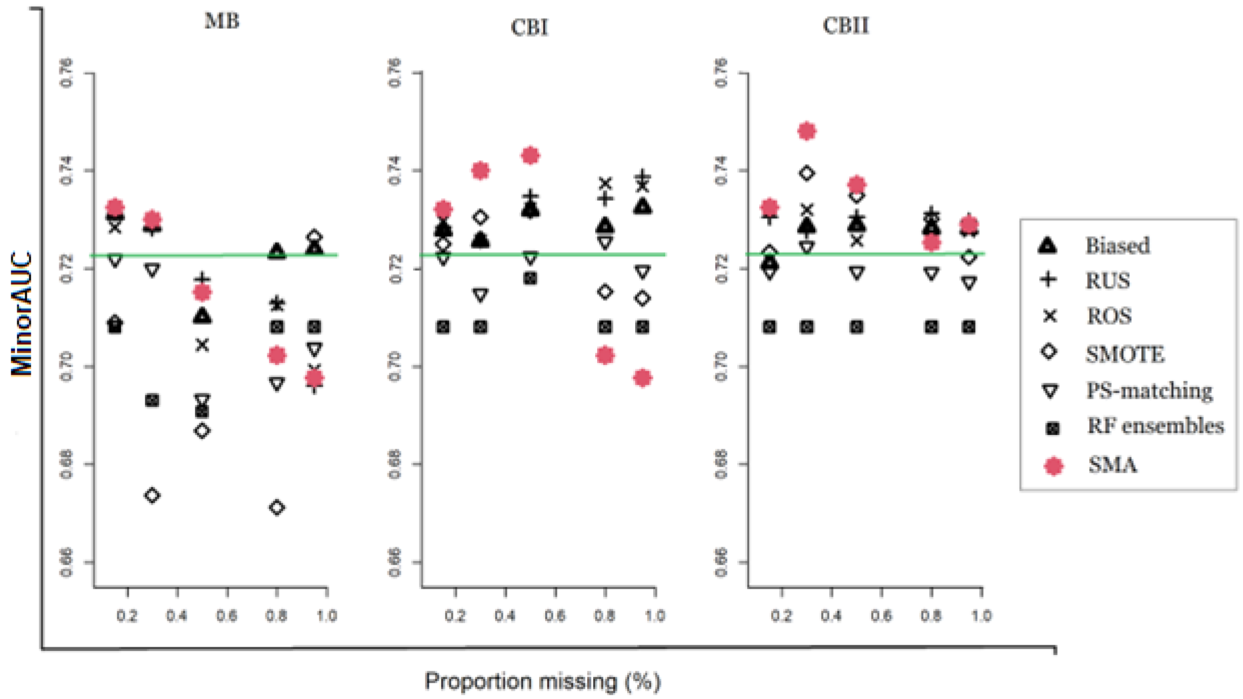

Figure S2. Danish Colorectal Cancer data. Plots of AUC estimates of the minority group of the biased covariate variable. MB denotes marginal bias, CBI for conditional bias I, and CBII for conditional bias II. Horizontal line is the ground truth estimate.

Missing	***15%***			***30%***			***50%***			***80%***			***95%***		
**Approach**	**MB**	**CB1**	**CBII**	**MB**	**MB**	**CBI**	**MB**	**CBI**	**CBII**	**MB**	**CBI**	**CBII**	**MB**	**CBI**	**CBII**
Biased data	0.07	0.07	0.07	0.07	0.07	0.07	0.07	0.07	0.06	0.06	0.06	0.06	0.07	0.07	0.07
RUS	0.07	0.07	0.07	0.07	0.07	0.07	0.07	0.07	0.07	0.07	0.07	0.07	0.07	0.07	0.07
ROS	0.07	0.07	0.07	0.07	0.07	0.07	0.07	0.07	0.07	0.07	0.07	0.07	0.07	0.07	0.07
SMOTE	0.08	0.08	0.07	0.08	0.09	0.09	0.08	0.08	0.08	0.09	0.07	0.08	0.07	0.07	0.09
PS-	0.07	0.07	0.07	0.05	0.05	0.05	0.07	0.07	0.07	0.07	0.07	0.07	0.07	0.07	0.07
RF	0.09	0.09	0.09	0.09	0.09	0.09	0.09	0.09	0.09	0.09	0.09	0.09	0.09	0.08	0.09
SMA	0.07	0.07	0.07	0.07	0.07	0.06	0.06	0.06	0.06	0.07	0.07	0.07	0.07	0.07	0.07

Table S14: Brier scores for predicting the minority group of the biased covariate under each bias mitigation method. The proportion of observations removed ranges from 15% to 95%. The reference value for the brier score of the minority group of the original cohort for Danish Colorectal Cancer data was 0.07. MB-CBII denotes marginal bias, conditional bias I, and conditional bias II, respectively.

D.3 Breast Cancer data

Breast Cancer	Approach	Marginal bias			Conditional bias I			Conditional bias II		
		AUC	OR_Z (SE)	I_Z	AUC	OR_Z (SE)	I_Z	AUC	OR_Z (SE)	I_Z
Missing prop = 15%	Biased data	0.81	0.81(-)	-	0.83	0.82 (-)	-	0.85	0.77(-)	-
	RUS	0.86	0.83(0.61)	0.83	0.82	1.11(0.52)	0.74	0.86	0.86(0.49)	0.89
	ROS	0.81	0.53(0.45)	0.77	0.84	0.82(0.46)	0.89	0.84	0.75(0.47)	0.99
	SMOTE	0.78	0.62(0.19)	0.68	0.78	0.52(0.13)	0.59	0.80	0.36(0.36)	0.52
	PS-matching	0.85	0.87(0.52)	0.87	0.85	0.81(0.89)	0.68	0.88	0.78(0.70)	0.77
	RF ensemble	0.82	-	-	0.82	-	-	0.83	-	-
	SMA	0.83	0.75(0.44)	0.96	0.84	0.75(0.51)	0.93	0.85	0.76(0.47)	0.99
Missing prop = 30%	Biased data	0.81	0.73(-)	-	0.83	0.82 (-)	-	0.84	0.83(-)	-
	RUS	0.82	0.84(0.61)	0.82	0.83	1.11(0.52)	0.74	0.86	0.83(0.51)	0.87
	ROS	0.81	0.53(0.45)	0.77	0.84	0.89(0.46)	0.89	0.84	0.75(0.47)	0.98
	SMOTE	0.78	0.52(0.13)	0.59	0.83	0.77(0.31)	0.82	0.81	0.36(0.36)	0.52
	PS-matching	0.86	0.67(0.52)	0.86	0.85	0.81(0.89)	0.68	0.88	0.78(0.70)	0.77
	RF ensemble	0.82	-	-	0.82	-	-	0.83	-	-
	SMA	0.83	0.75(0.45)	0.95	0.84	0.75(0.51)	0.93	0.85	0.76(0.47)	0.99
Missing prop = 50%	Biased data	0.80	0.58(-)	-	0.83	0.80 (-)	-	0.84	0.68(-)	-
	RUS	0.83	0.69(0.72)	0.82	0.82	1.11(0.52)	0.74	0.83	0.53 (0.52)	0.84
	ROS	0.79	0.67(0.46)	0.91	0.82	0.78(0.46)	0.98	0.85	0.67(0.49)	0.94
	SMOTE	0.78	0.52(0.13)	0.59	0.82	0.47(0.38)	0.66	0.81	0.36(0.36)	0.52
	PS-matching	0.87	0.41(1.44)	0.61	0.81	0.40(1.04)	0.76	0.89	0.42(0.72)	0.91
	RF ensemble	0.83	-	-	0.81	-	-	0.80	-	-
	SMA	0.83	0.75(0.47)	0.94	0.82	0.77(0.51)	0.98	0.85	0.70(0.49)	0.95
Missing prop = 80%	Biased data	0.82	1.48(-)	-	0.81	0.82 (-)	-	0.84	0.77(-)	-
	RUS	0.80	0.83(0.61)	0.82	0.79	0.75(0.52)	0.80	0.84	0.70(0.55)	0.94
	ROS	0.86	1.58(0.58)	0.59	0.82	0.81(0.43)	1.00	0.86	0.76(0.50)	0.95
	SMOTE	0.78	2.77(0.13)	0.24	0.79	0.77(0.31)	0.82	0.80	0.60(0.35)	0.78
	PS-matching	0.86	1.05(1.46)	0.54	0.82	0.08(0.89)	0.66	0.87	0.82(0.70)	0.68
	RF ensemble	0.80	-	-	0.79	-	-	0.77	-	-
	SMA	0.86	0.95(0.54)	0.65	0.81	0.75(0.51)	0.95	0.87	0.75(0.51)	0.94

Table S15: Breast Cancer data. Estimates of mean AUC, Odds Ratio of Z [OR_Z(SE)], and confidence interval overlaps (I_Z) of the biased covariate Z for each bias mitigating approach. For the original data: AUC = 0.84; OR_Z(SE) = 0.76 (0.46); I_Z =1.00.

		Marginal bias			Conditional bias I			Conditional bias II		
		SPD	EOD	AOD	SPD	EOD	AOD	SPD	EOD	AOD
15%	Original	-0.04843516	-0.05803158	0.05093023	-0.04843516	-0.05803158	0.05093023	-0.04843516	-0.05803158	0.05093023
	Biased	-0.07819081	-0.09010742	0.08128913	-0.06545581	-0.07753269	0.06859580	-0.03465149	-0.04224985	0.03662706
	RUS	-0.12121716	-0.12780123	0.12292902	-0.05770824	-0.06944574	0.06075999	-0.04565305	-0.05474187	0.04801614
	ROS	-0.11683552	-0.12741212	0.11958544	-0.07594085	-0.08867105	0.07925070	-0.04922790	-0.05894357	0.05175397
	PS-matching	-0.23071950	-0.24536109	0.23474593	-0.09304108	-0.10899817	0.09742928	-0.07263817	-0.08916006	0.07718169
	SMOTE	-0.07528525	-0.08502369	0.07781724	-0.10525716	-0.11531926	0.10787331	0.07940146	0.07911960	0.07932817
	SMA	-0.05224629	-0.06207177	0.05480091	-0.05224629	-0.06207177	0.05480091	-0.03596337	-0.04402445	0.03805925
		SPD	EOD	AOD	SPD	EOD	AOD	SPD	EOD	AOD
30%	Original	-0.04843516	-0.05803158	0.05093023	-0.04843516	-0.05803158	0.05093023	-0.04843516	-0.05803158	0.05093023
	Biased	-0.10026835	-0.11657459	0.10450797	-0.04297181	-0.05070168	0.04498158	-0.07586980	-0.08798721	0.07902033
	RUS	-0.07528107	-0.08822990	0.07864777	-0.08769870	-0.09711186	0.09014612	-0.06596229	-0.07732908	0.06891765
	ROS	-0.12281645	-0.14568468	0.12876219	-0.02887323	-0.03477914	0.03040877	-0.07953287	-0.09209151	0.08279811
	PS-matching	-0.34030215	-0.36761277	0.34781257	-0.14585204	-0.16110126	0.15004558	-0.13021999	-0.14707246	0.13485442
	SMOTE	-0.11134809	-0.12739281	0.11551971	-0.35858289	-0.38468826	0.36537029	-0.19846967	-0.20572591	0.20035629
	SMA	-0.21124876	-0.22964741	0.21603241	-0.21124876	-0.22964741	0.21603241	-0.10403607	-0.11621859	0.10720353
		SPD	EOD	AOD	SPD	EOD	AOD	SPD	EOD	AOD
50%	Original	-0.04843516	-0.05803158	0.05093023	-0.048435164	-0.058031579	0.050930232	-0.04843516	-0.05803158	0.05093023
	Biased	-0.02430764	-0.02825330	0.02533351	0.002928406	0.003599259	0.003102828	-0.06694935	-0.07822892	0.06988204
	RUS	0.04723793	0.04789455	0.04740865	0.017476690	0.021570967	0.018541202	-0.05959601	-0.06826943	0.06185110
	ROS	-0.03311307	-0.03832999	0.03446947	-0.030395213	-0.035537398	0.031732181	-0.11362300	-0.12639483	0.11694368
	PS-matching	-0.02998319	-0.04404133	0.03384918	-0.152208783	-0.184100211	0.160978925	-0.16306363	-0.18107604	0.16801704
	SMOTE	-0.05402994	-0.06255927	0.05624756	-0.124482415	-0.163294073	0.134573446	-0.29961613	-0.31886018	0.30461958
	SMA	-0.03691470	-0.04057452	0.03786626	-0.036914705	-0.040574521	0.037866257	-0.07102527	-0.08056575	0.07350579
		SPD	EOD	AOD	SPD	EOD	AOD	SPD	EOD	AOD
80%	Original	-0.04843516	-0.05803158	0.05093023	-0.048435164	-0.05803158	0.05093023	-0.04843516	-0.05803158	0.05093023
	Biased	-0.06735357	-0.07465980	0.06925319	-0.050101338	-0.05913962	0.05245129	-0.07408776	-0.08668486	0.07736301
	RUS	-0.12122316	-0.14977156	0.12864575	0.008897549	0.01024975	0.00924912	-0.12564678	-0.14446418	0.13053930
	ROS	-0.03893138	-0.04656619	0.04091643	-0.100314735	-0.11559274	0.10428702	-0.08189089	-0.09590478	0.08553450
	PS-matching	0.24724283	0.29330562	0.25991010	-0.375004880	-0.37887048	0.37606792	-0.11436521	-0.13173354	0.11914150
	SMOTE	-0.06828342	-0.08546733	0.07275124	-0.354386306	-0.32044730	0.34556216	-0.19241255	-0.18105866	0.18946054
	SMA	-0.05486941	-0.06432072	0.05732675	-0.054869410	-0.06432072	0.05732675	-0.07344795	-0.08422348	0.07624959

Table S16: Measure of fairness. Estimates of the statistical parity difference (SPD), equal opportunity difference (EOD), and average odds difference (AOD) on the Breast Cancer data.

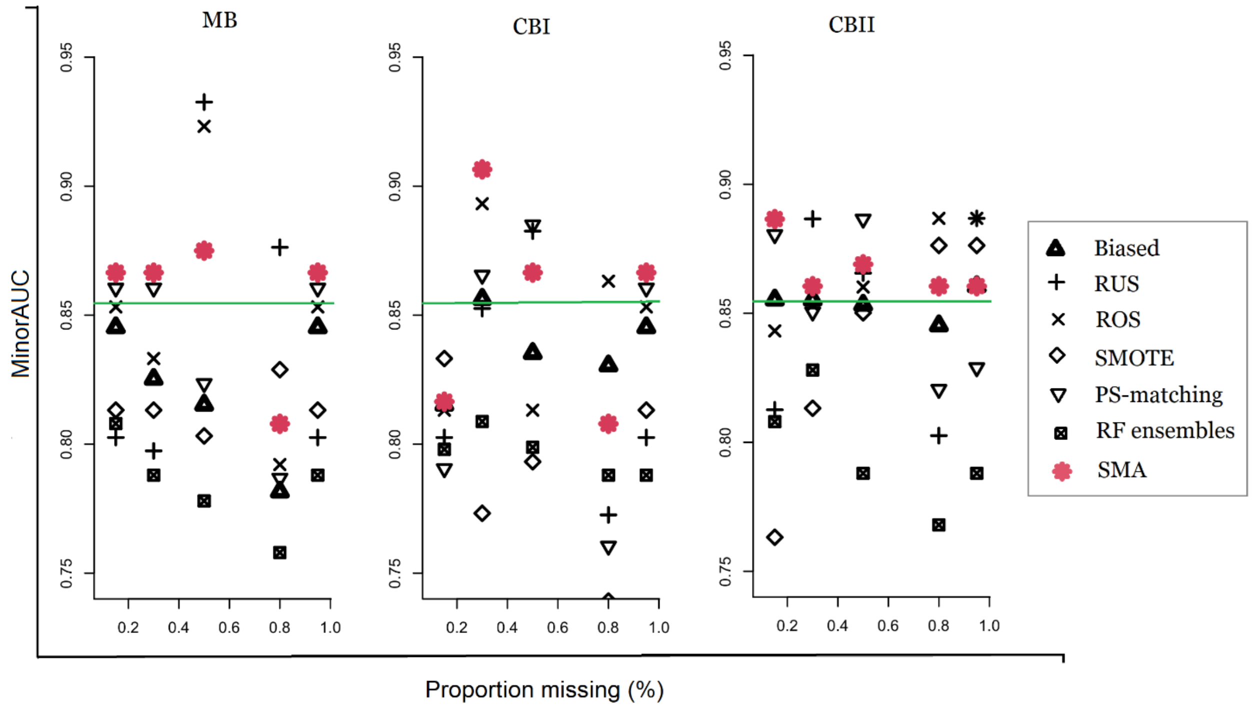

Figure S3. Breast Cancer data. Plot of AUC estimates of the minor categories of the biased covariate. MB denotes marginal bias, CBI for conditional bias I, and CBII for conditional bias II. Horizontal line is the ground truth estimate.

Missing	*15%*			*30%*			*50%*			*80%*			*95%*		
**Approach**	**MB**	**CB1**	**CBII**	**MB**	**MB**	**CBI**	**MB**	**CBI**	**CBII**	**MB**	**CBI**	**CBII**	**MB**	**CBI**	**CBII**
Biased data	0.07	0.07	0.07	0.07	0.07	0.07	0.07	0.07	0.07	0.07	0.07	0.07	0.07	0.07	0.07
RUS	0.07	0.07	0.07	0.07	0.07	0.07	0.07	0.07	0.07	0.07	0.07	0.07	0.07	0.07	0.07
ROS	0.07	0.07	0.07	0.07	0.07	0.07	0.07	0.07	0.07	0.07	0.07	0.07	0.07	0.07	0.07
SMOTE	0.09	0.09	0.09	0.09	0.12	0.1	0.09	0.09	0.09	0.09	0.07	0.1	0.09	0.07	0.07
PS-	0.07	0.07	0.05	0.05	0.07	0.05	0.07	0.07	0.05	0.07	0.05	0.05	0.07	0.07	0.05
RF	0.1	0.1	0.1	0.1	0.1	0.1	0.1	0.1	0.1	0.1	0.1	0.09	0.09	0.09	0.1
SMA	0.05	0.05	0.07	0.05	0.05	0.05	0.05	0.05	0.05	0.07	0.07	0.07	0.07	0.07	0.07

Table S17: Brier scores for predicting the minority group of the biased covariate under each bias mitigation method. The proportion of observations removed ranges from 15% to 95%. The reference value for the brier score of the minority group of the original cohort for Breast Cancer data was 0.05. MB-CBII denotes marginal bias, conditional bias I, and conditional bias II, respectively.

D.4 Cardiovascular Health data

CCHS data Missing Prop	Approach	Marginal bias			Conditional bias I			Conditional bias II		
		AUC	OR_Z (SE)	I_Z	AUC	OR_Z (SE)	I_Z	AUC	OR_Z (SE)	I_Z
15%	Biased data	0.70	1.57(-)	-	0.70	1.57(-)	-	0.70	1.58(-)	-
	RUS	0.70	1.58(0.03)	0.94	0.70	1.58(0.03)	0.94	0.70	1.57(0.02)	0.97
	ROS	0.70	1.57(0.02)	0.97	0.70	1.57(0.02)	0.95	0.70	1.58(0.02)	0.97
	SMOTE	0.70	1.52(0.04)	0.77	0.70	1.56(0.04)	0.76	0.70	1.57(0.04)	0.78
	PS-matching	0.69	1.50(0.03)	0.56	0.69	1.50(0.03)	0.53	0.69	1.54(0.03)	0.73
	RF ensemble	0.70	-	-	0.70	-	-	0.70	-	-
	SMA‡	0.70	1.57(0.03)	0.97	0.70	1.57(0.03)	0.94	0.70	1.58(0.03)	0.97
30%	Biased data	0.70	1.59(-)	-	0.70	1.61(-)	-	0.70	1.59(-)	-
	RUS	0.70	1.59(0.03)	0.89	0.70	1.61(0.03)	0.78	0.70	1.58(0.03)	0.94
	ROS	0.70	1.60(0.02)	0.86	0.70	1.60 (0.02)	0.84	0.70	1.60 (0.02)	0.84
	SMOTE	0.69	1.81(0.02)	0.00	0.69	1.88(0.02)	0.00	0.70	1.83(0.04)	0.00
	PS-matching	0.69	1.48(0.03)	0.44	0.69	1.51(0.03)	0.54	0.69	1.56(0.03)	0.92
	RF ensemble	0.70	-	-	0.68	-	-	0.69	-	-
	SMA‡	0.70	1.59(0.02)	0.91	0.70	1.59(0.02)	0.94	0.70	1.80(0.02)	0.95
50%	Biased data	0.70	1.61(-)	-	0.69	1.63(-)	-	0.69	1.61(-)	-
	RUS	0.70	1.59(0.03)	0.83	0.70	1.61(0.03)	0.81	0.70	1.60(0.03)	0.82
	ROS	0.70	1.59(0.02)	0.87	0.70	1.61(0.02)	0.77	0.70	1.61(0.02)	0.74
	SMOTE	0.68	1.85(0.02)	0.66	0.69	1.89(0.02)	0.00	0.69	1.85(0.02)	0.00
	PS-matching	0.69	1.44(0.04)	0.29	0.69	1.49(0.04)	0.60	0.69	1.57(0.03)	0.89
	RF ensemble	0.69	-	-	0.69	-	-	0.68	-	-
	SMA‡	0.70	1.59(0.02)	0.89	0.70	1.59(0.02)	0.90	0.70	1.59(0.02)	0.90
80%	Biased data	0.69	1.66(-)	-	0.69	1.66(-)	-	0.69	1.61(-)	-
	RUS	0.70	1.60(0.05)	0.75	0.70	1.60(0.05)	0.75	0.70	1.61(0.03)	0.77
	ROS	0.70	1.65(0.02)	0.59	0.70	1.65(0.02)	0.49	0.70	1.62(0.02)	0.67
	SMOTE	0.68	1.82(0.03)	0.00	0.69	1.81(0.03)	0.00	0.69	1.79(0.02)	0.00
	PS-matching	0.70	1.50(0.07)	0.67	0.69	1.50 (0.07)	0.67	0.69	1.49(0.03)	0.49
	RF ensemble	0.69	-	-	0.69	-	-	0.68	-	-
	SMA‡	0.70	1.56(0.02)	0.78	0.70	1.57(0.02)	0.82	0.70	1.56(0.02)	0.80

Table S18: Estimates of mean AUC, Odds Ratio of Z [OR_Z(SE)], and interval overlaps (I_Z) of the biased covariate Z for each bias mitigating approach on the Cardiovascular Health (CCHS) data. For the Original data: AUC = 0.70; OR_Z(SE) = 1.58 (0.02); I_Z =1.00. Note. Synthetic Minor Augmentation (SMA). ‡ indicates that the estimates are averaged from m=100 synthetic copies.

Missing Prop	Approach	SPD			EOD			AOD		
		MB	CB1	CBII	MB	CB1	CBII	MB	CB1	CBII
15%	Biased	0.095	0.097	0.097	0.091	0.093	0.093	0.097	0.099	0.099
	RUS	0.097	0.098	0.096	0.093	0.094	0.092	0.099	0.099	0.097
	ROS	0.096	0.098	0.096	0.092	0.094	0.092	0.098	0.099	0.098
	PS-matching	0.091	0.090	0.094	0.089	0.088	0.092	0.092	0.091	0.095
	SMOTE	0.098	0.092	0.108	0.095	0.089	0.104	0.100	0.093	0.110
	SMA‡	0.095	0.096	0.097	0.092	0.092	0.093	0.097	0.097	0.099
30%	Biased	0.099	0.095	0.098	0.096	0.092	0.094	0.101	0.097	0.100
	RUS	0.099	0.093	0.097	0.095	0.089	0.094	0.101	0.094	0.099
	ROS	0.097	0.096	0.098	0.093	0.092	0.094	0.099	0.097	0.099
	PS-matching	0.088	0.087	0.096	0.085	0.084	0.094	0.088	0.087	0.097
	SMOTE	0.115	0.097	0.107	0.110	0.094	0.103	0.117	0.099	0.108
	SMA‡	0.102	0.093	0.098	0.094	0.090	0.094	0.104	0.095	0.100
50%	Biased	0.098	0.104	0.098	0.095	0.100	0.095	0.100	0.106	0.100
	RUS	0.096	0.101	0.096	0.092	0.097	0.093	0.098	0.103	0.098
	ROS	0.100	0.099	0.095	0.096	0.095	0.091	0.102	0.101	0.096
	PS-matching	0.088	0.089	0.092	0.085	0.086	0.090	0.088	0.089	0.093
	SMOTE	0.098	0.097	0.097	0.094	0.093	0.094	0.099	0.098	0.098
	SMA‡	0.103	0.101	0.096	0.099	0.094	0.093	0.105	0.103	0.098
80%	Biased	0.111	0.103	0.104	0.107	0.099	0.100	0.112	0.105	0.105
	RUS	0.106	0.098	0.105	0.102	0.094	0.102	0.107	0.099	0.107
	ROS	0.113	0.095	0.100	0.109	0.092	0.096	0.115	0.097	0.101
	PS-matching	0.115	0.092	0.092	0.112	0.090	0.090	0.116	0.093	0.093
	SMOTE	0.094	0.100	0.104	0.091	0.097	0.101	0.095	0.102	0.106
	SMA‡	0.104	0.106	0.100	0.100	0.102	0.096	0.106	0.108	0.102

Table S19: Fairness metrics: the statistical parity difference (SPD), equal opportunity difference (EOD), and average odds difference (AOD) from the logistic regression model on the cardiovascular health data. The proportion of samples removed varied from 15% to 80%. Original cohort: SPD = 0.096, EOD = 0.092, and AOD = 0.098. **‡** indicates that the estimates are averaged from m=100 synthetic copies.

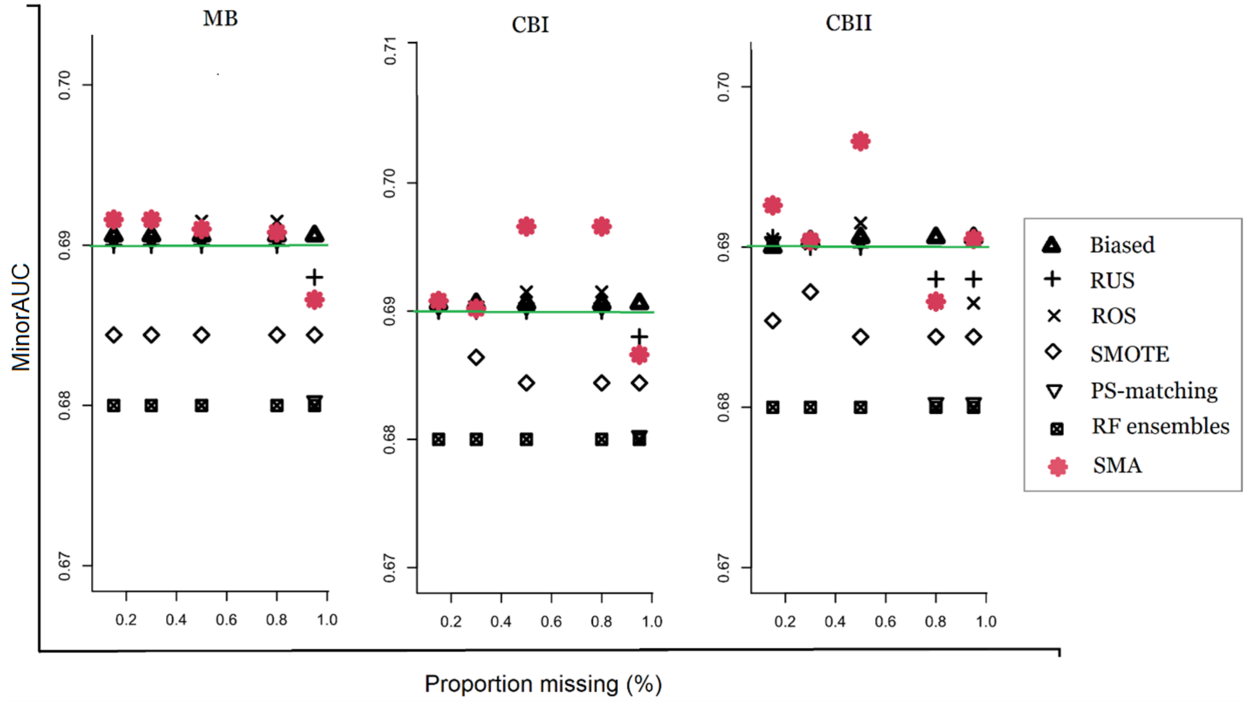

Figure S4: Cardiovascular Health data used for the stratified model. Plots of AUC estimates of the minor categories of the biased covariate. MB denotes marginal bias, CBI for conditional bias I, and CBII for conditional bias II. The horizontal line is the ground truth estimate (AUC =0.69).

Missing	***15%***			***30%***			***50%***			***80%***			***95%***		
**Approach**	**MB**	**CB1**	**CBII**	**MB**	**MB**	**CBI**	**MB**	**CBI**	**CBII**	**MB**	**CBI**	**CBII**	**MB**	**CBI**	**CBII**
Biased data	0.04	0.05	0.05	0.04	0.05	0.05	0.04	0.05	0.05	0.04	0.05	0.05	0.04	0.05	0.05
RUS	0.04	0.04	0.04	0.04	0.04	0.04	0.04	0.04	0.04	0.04	0.04	0.04	0.04	0.04	0.04
ROS	0.04	0.04	0.04	0.04	0.04	0.04	0.04	0.04	0.04	0.04	0.04	0.04	0.04	0.04	0.04
SMOTE	0.05	0.05	0.04	0.05	0.05	0.05	0.05	0.05	0.05	0.07	0.04	0.04	0.04	0.04	0.08
PS-	0.04	0.04	0.04	0.04	0.04	0.04	0.04	0.04	0.04	0.04	0.04	0.04	0.04	0.04	0.04
RF	0.05	0.05	0.05	0.05	0.05	0.05	0.06	0.05	0.05	0.06	0.05	0.06	0.06	0.05	0.06
SMA	0.04	0.04	0.04	0.04	0.04	0.04	0.04	0.04	0.04	0.04	0.04	0.04	0.04	0.04	0.04

Table S20: Brier scores for predicting the minority group of the biased covariate under each bias mitigation method. The proportion of observations removed ranges from 15% to 95%. The reference value for the brier score of the minority group of the original cohort for Cardiovascular health data was 0.04, respectively. MB-CBII denotes marginal bias, conditional bias I, and conditional bias II, respectively.

D.5 Summaries of all real data results

We present plots of the relative performance of the metrics over the four real datasets for (I) low to medium bias, and (II) high bias. The plots include summaries for overall model effect estimates (Odds ratios), AUCs, Brier scores, Fairness estimates, and AUC of the minority group.

D.5.1 Overall AUCs

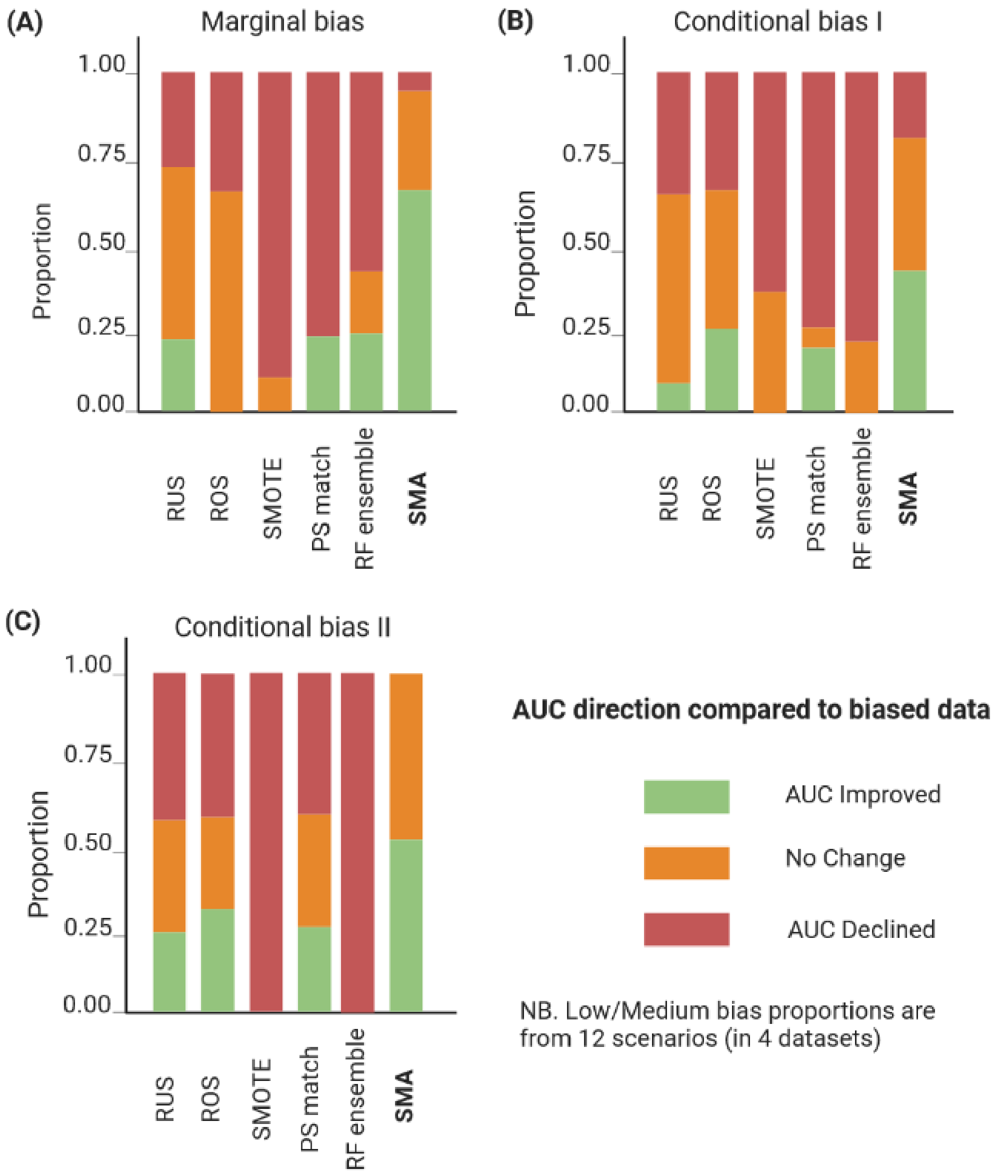

Figure S5: Low to Medium bias setting. Summaries of the model AUC for **low to medium bias** over the four real datasets. The relative performance of each bias mitigating approach compared to biased data results is shown. The model AUC is considered improved if the difference between the model AUC and the ground truth estimate is less than the difference between the biased data AUC and the ground truth. Summaries are over bias proportions: 15%, 30%, and 50%.

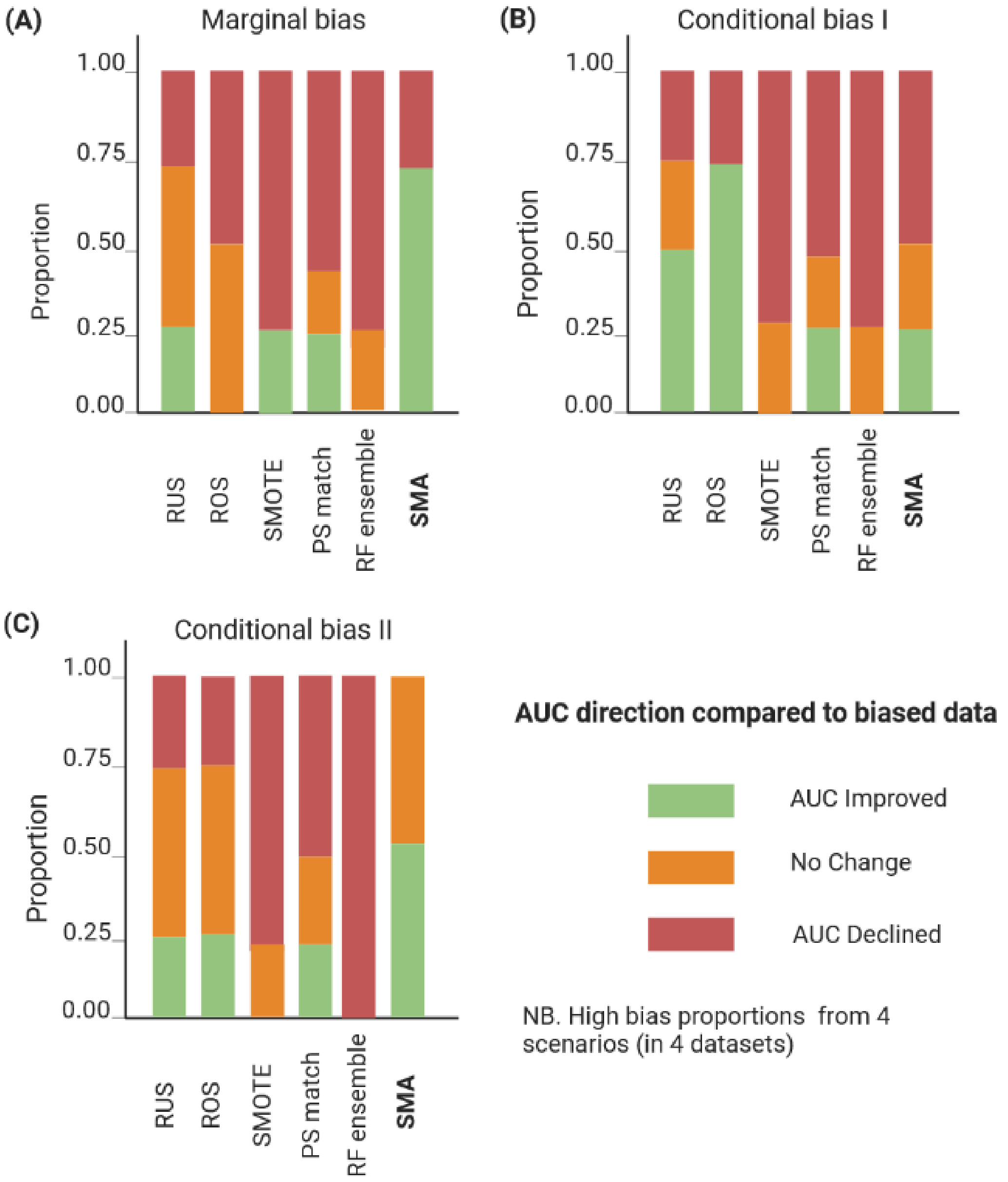

Figure S6: High bias setting. Summaries of the model AUC for **high bias** over the four real datasets. The relative performance of each bias mitigating approach compared to biased data results is shown. The model AUC is considered improved if the difference between the model AUC and the ground truth estimate is less than the difference between the biased data AUC and the ground truth. Summaries are over bias proportion of 80%.

D.5.2 Odds Ratio

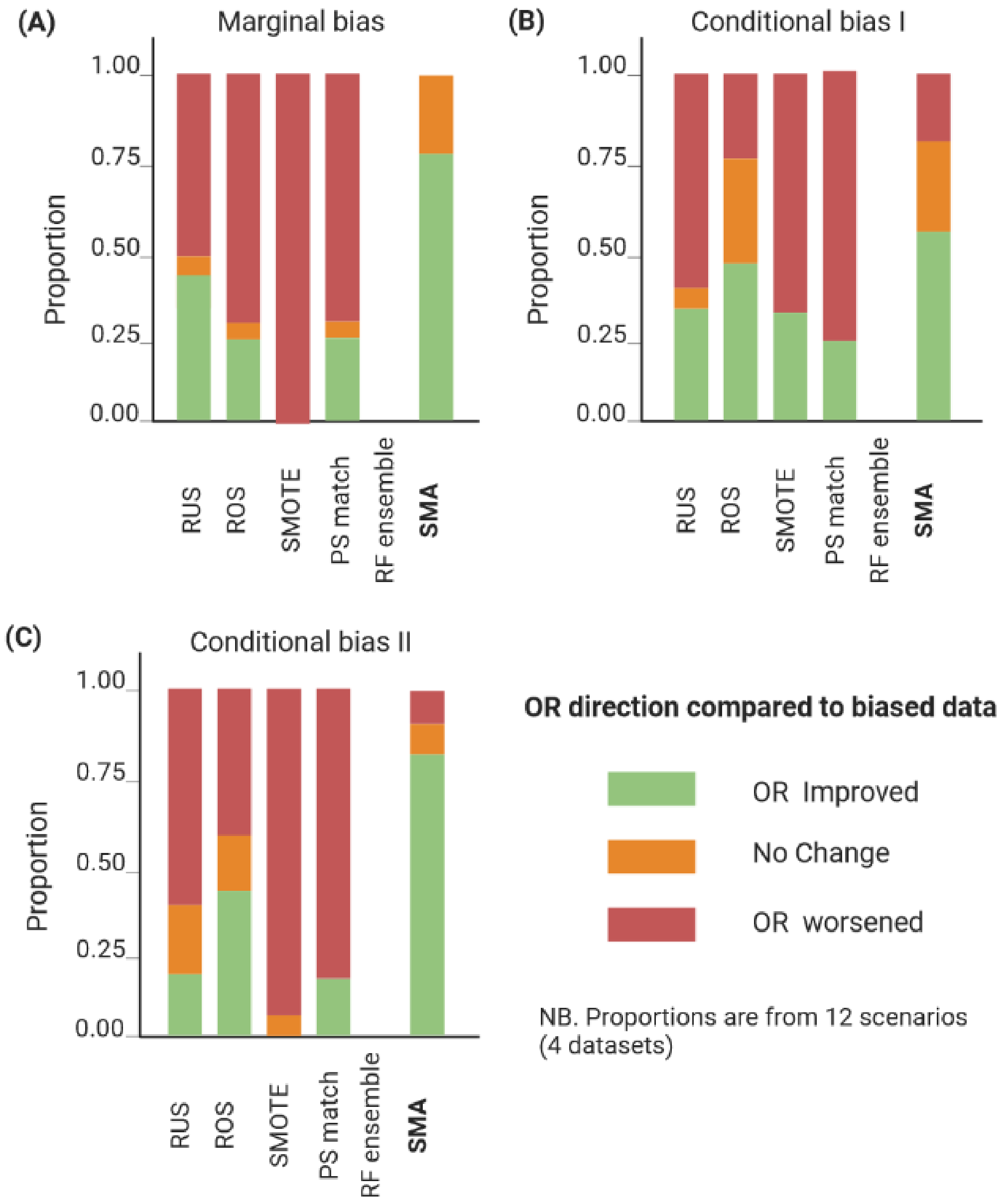

Figure S7: Low to Medium bias setting. Summaries of the Odds Ratio (OR) of the biasing covariate for **low to medium bias** over the four real datasets. The relative performance of each bias mitigating approach compared to the biased data results is shown. The OR direction is considered improved if the difference between the model OR and the ground truth estimate is less than the difference between the biased data OR and the ground truth. Summaries are over bias proportions: 15%, 30%, and 50%.

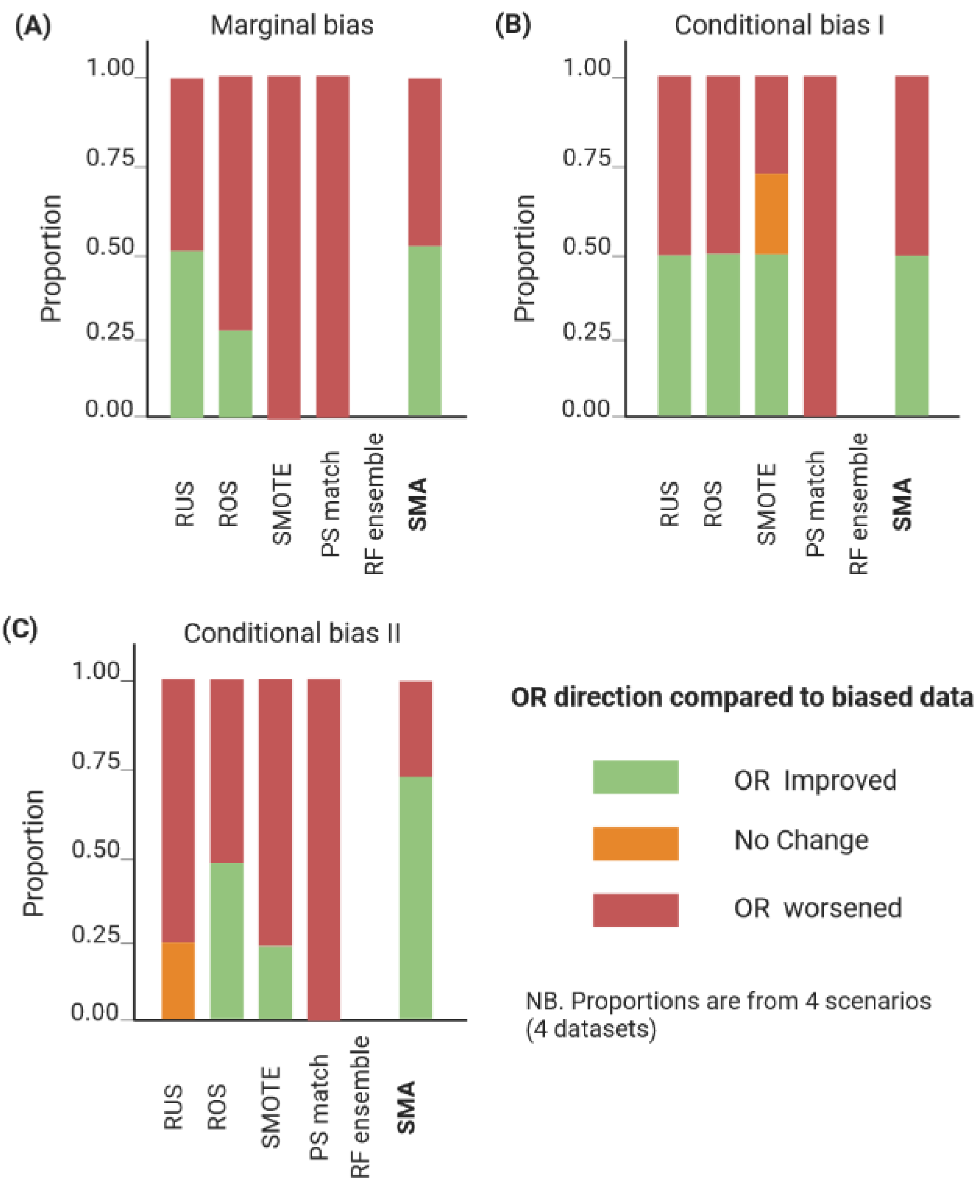

Figure S8: High bias setting. Summaries of the Odds Ratio (OR) of the biasing covariate for **high bias** over the four real datasets. The relative performance of each bias mitigating approach compared to the biased data results is shown. The OR direction is considered improved if the difference between the model OR and the ground truth estimate is less than the difference between the biased data OR and the ground truth. Summaries are over bias proportion of 80%.

D.5.3 Brier Score

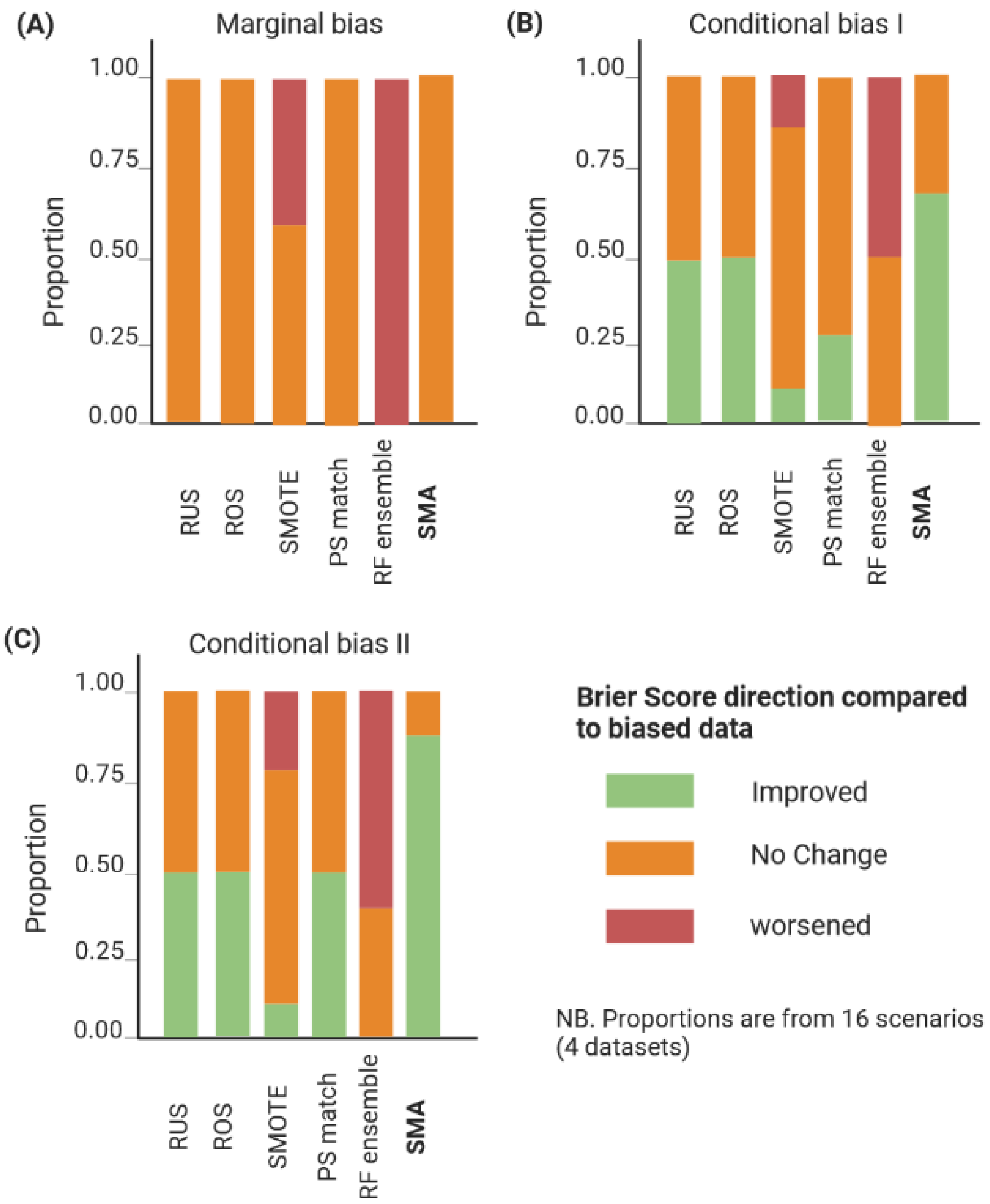

Figure S9: Overall summaries of the model Brier scores over the four real datasets. The relative performance of each bias mitigating approach compared to biased data results is shown. The Brier score is considered improved if the difference between the model brier score and the ground truth estimate is less than the difference between the biased data Brier score and the ground truth. Summaries are over all bias proportions: 15%, 30%, 50%, and 80%.

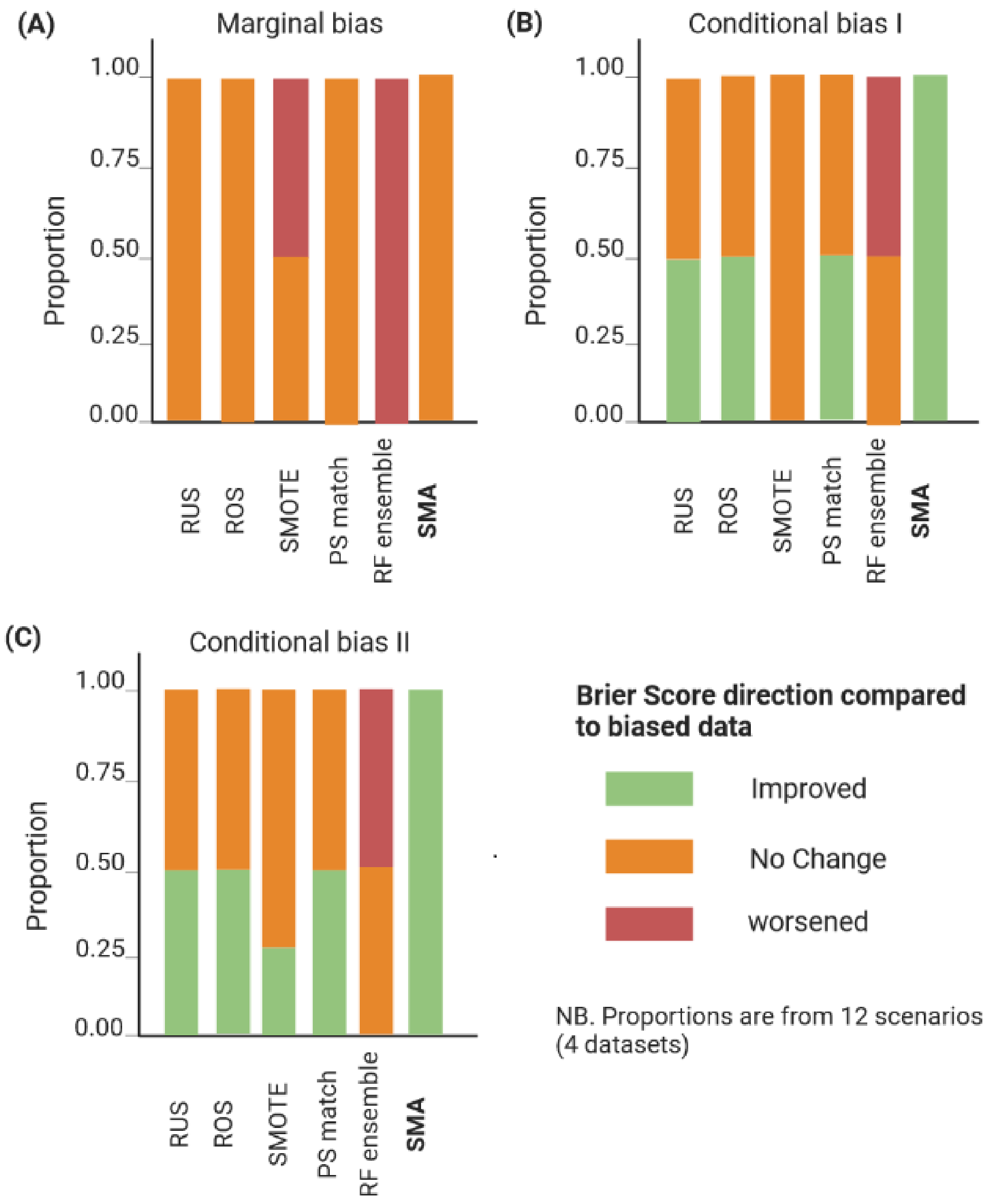

Figure S10: Low to Medium bias setting. Summaries of the model Brier scores over the four real datasets for **low to medium bias**. The relative performance of each bias mitigating approach compared to biased data results is shown. The Brier score is considered improved if the difference between the model brier score and the ground truth estimate is less than the difference between the biased data Brier score and the ground truth. Summaries are over all bias proportions: 15%, 30%, and 50%.

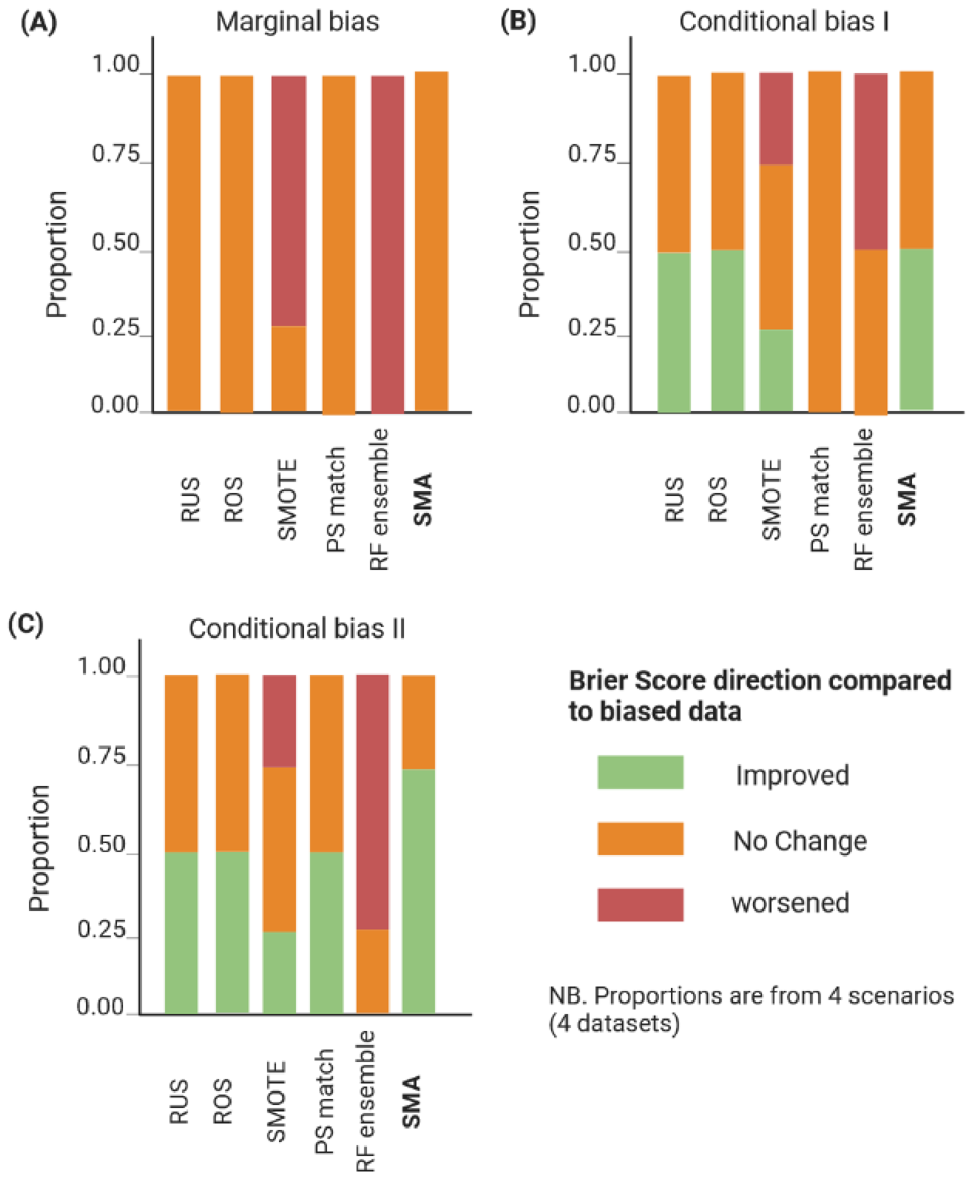

Figure S11: High bias setting. Summaries of the model Brier scores over the four real datasets for **high bias**. The relative performance of each bias mitigating approach compared to biased data results is shown. The Brier score is considered improved if the difference between the model brier score and the ground truth estimate is less than the difference between the biased data Brier score and the ground truth. Summaries are over bias proportion of 80%.

D.5.4 Fairness Metrics

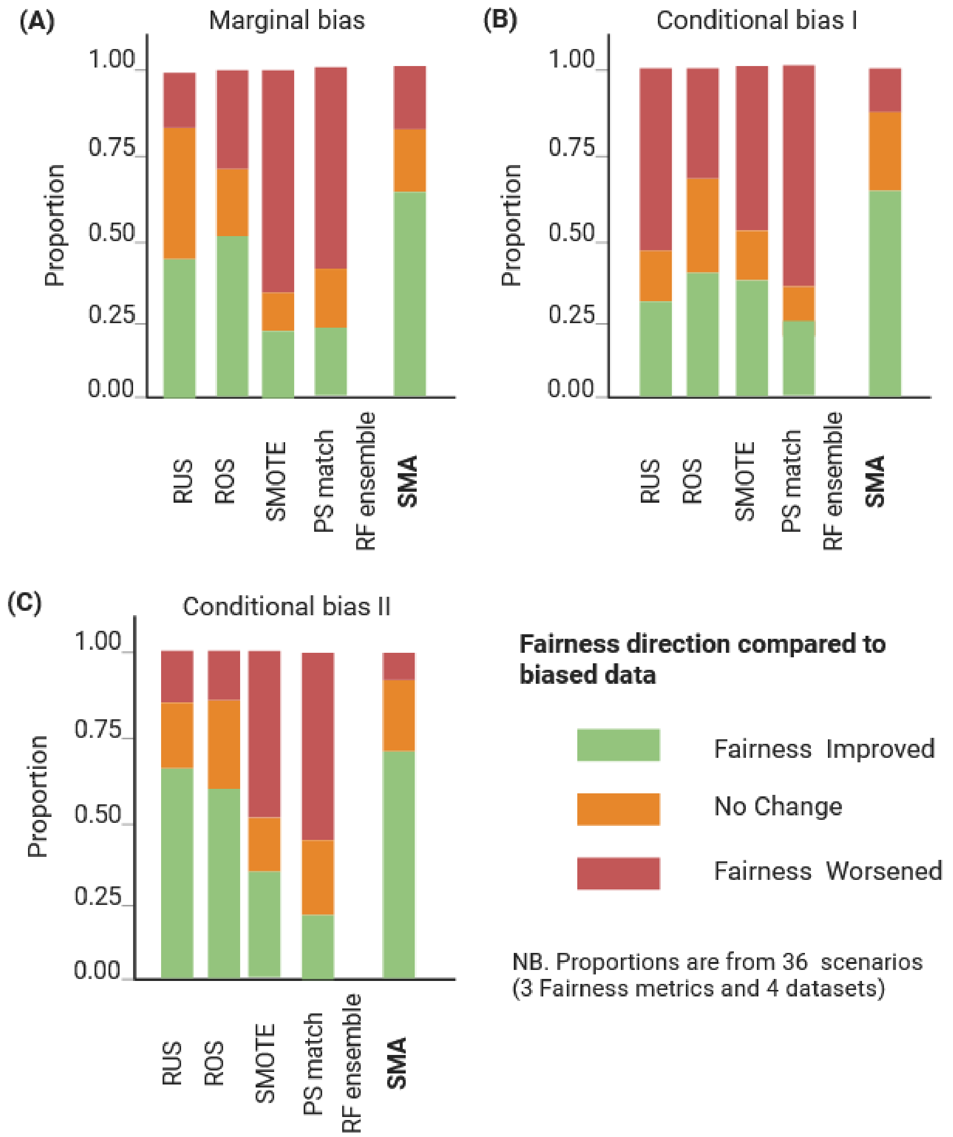

Figure S12: Low to Medium bias setting. Summaries of the Fairness metrics: the statistical parity difference (SPD), equal opportunity difference (EOD), and average odds difference (AOD) over the four real datasets for **low to medium bias**. The relative performance of each bias approach compared to biased data results is shown. Fairness is considered improved if the difference between the model Fairness and the ground truth estimate is less than the difference between the biased data Fairness and the ground truth. Summaries are over all bias proportions: 15%, 30%, and 50%.

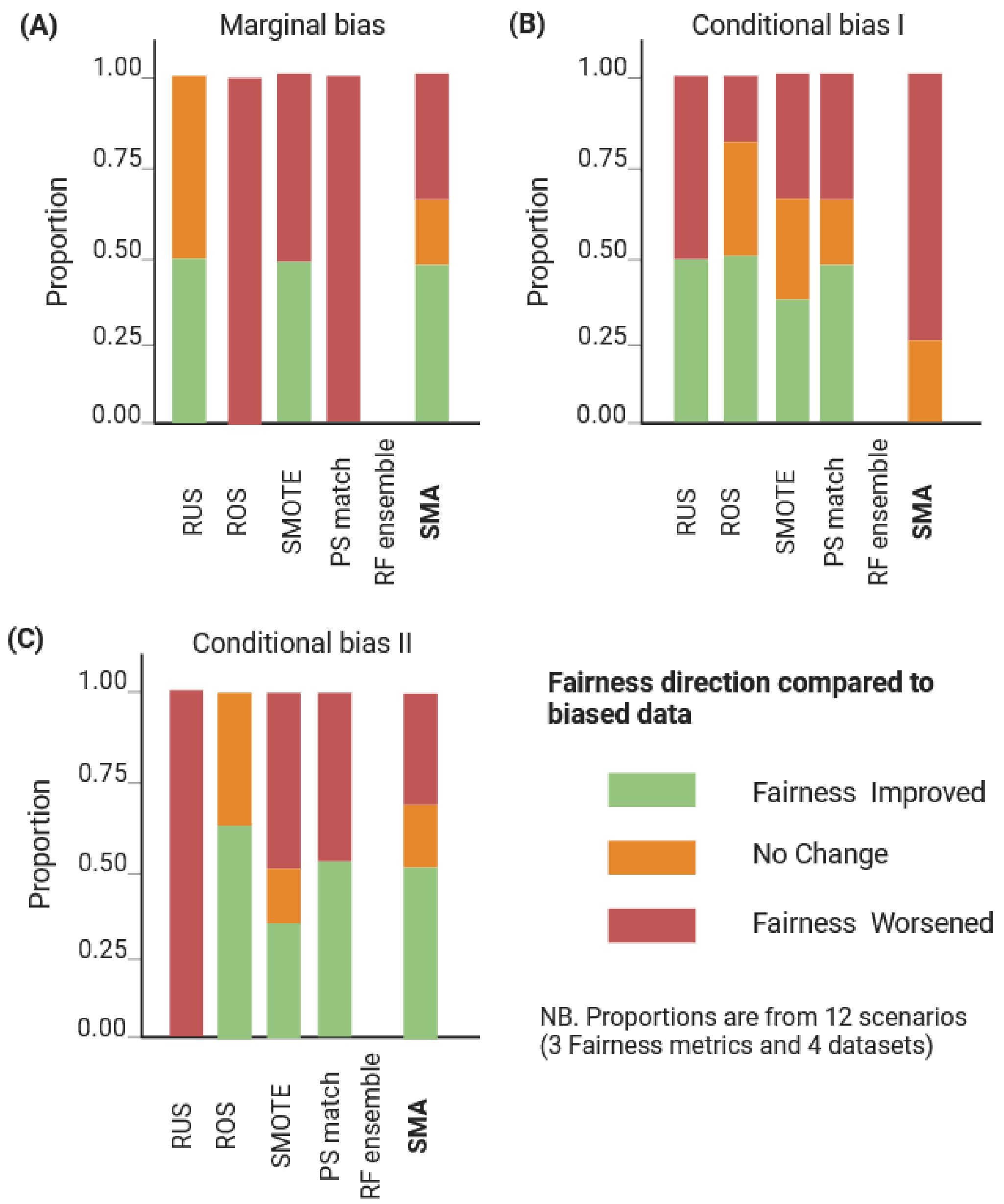

Figure S13: High bias setting. Summaries of the Fairness metrics: the statistical parity difference (SPD), equal opportunity difference (EOD), and average odds difference (AOD) over the four real datasets for **high bias**. The relative performance of each bias approach compared to biased data results is shown. Fairness is considered improved if the difference between the model Fairness and the ground truth estimate is less than the difference between the biased data Fairness and the ground truth. Summaries are over bias proportion of 80%.

D.5.5 AUC for the minority group of the biasing covariate

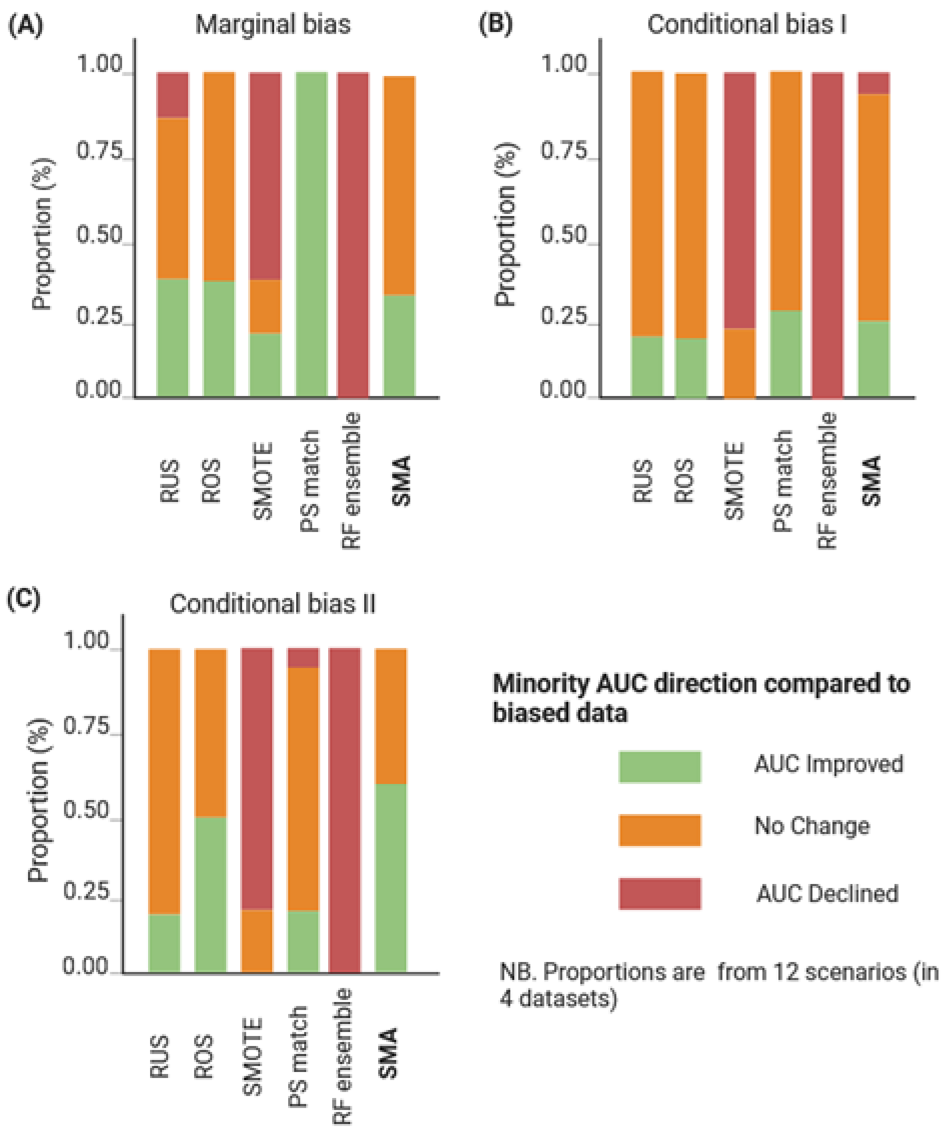

Figure S14: Low to Medium bias setting. Summaries of the model minority AUC for low to medium bias over the four real datasets for **low to medium bias**. The relative performance of each bias mitigating approach compared to biased data results is shown. The minority AUC is considered improved if the difference between the model minority AUC and the ground truth estimate is less than the difference between the biased data minority AUC and the ground truth. Summaries are over all bias proportions: 15%, 30%, and 50%.

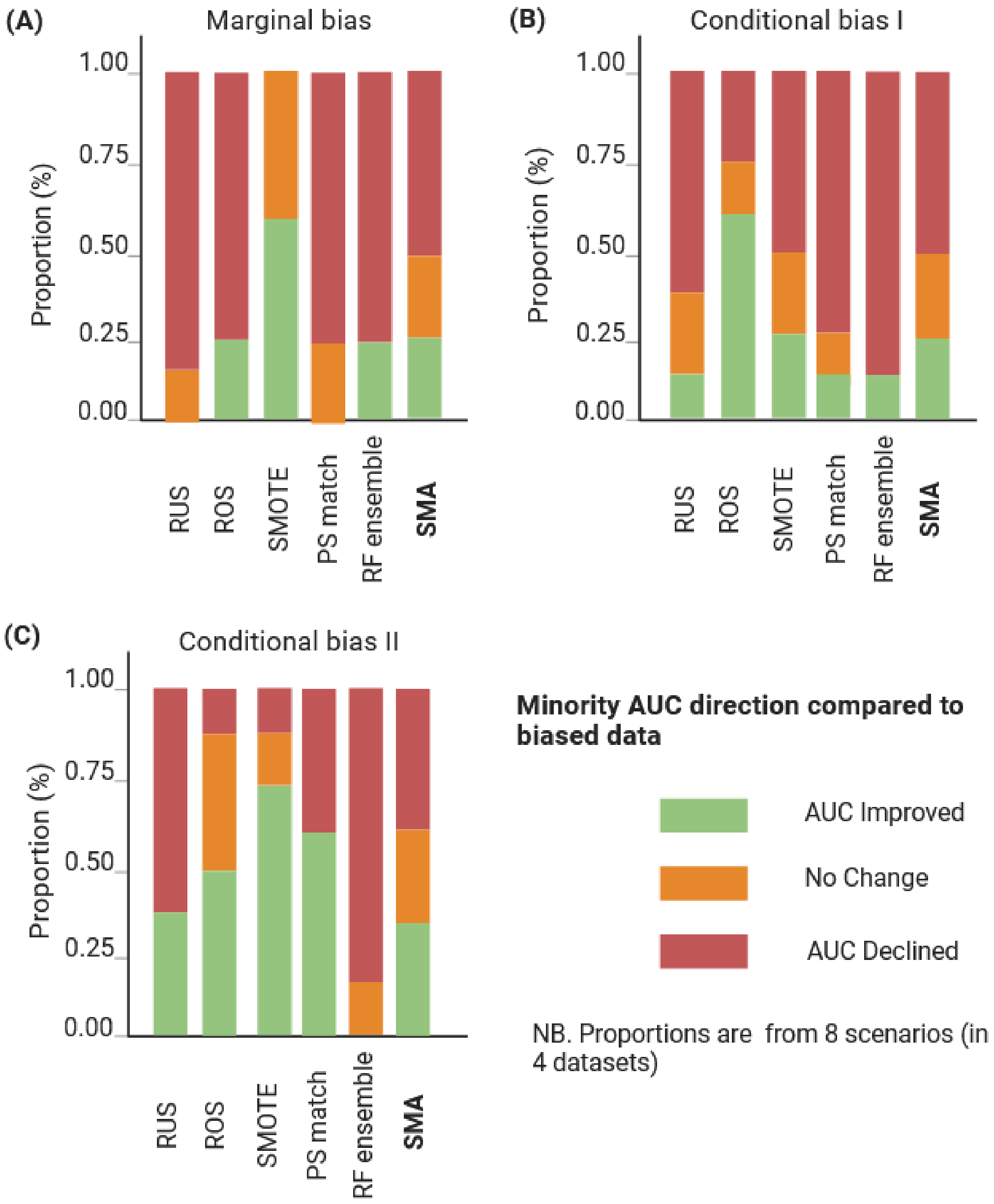

Figure S15: High bias setting. Summaries of the model minority AUC for high bias over the four real datasets for **high bias**. The relative performance of each bias mitigating approach compared to biased data results is shown. The minority AUC is considered improved if the difference between the model minority AUC and the ground truth estimate is less than the difference between the biased data minority AUC and the ground truth. Summaries are over bias proportion of 80% and 95%.

References

1. Chawla, N.V. (2009). Data mining for imbalanced datasets: An overview. Data Min. Knowl. Discov. Handb., 875–886.

2. Sun, Y., Wong, A.K.C., and Kamel, M.S. (2009). Classification of imbalanced data: a review. Int. J. Pattern Recognit. Artif. Intell. *23*, 687–719. 10.1142/S0218001409007326.

3. Glauner, P., Valtchev, P., and State, R. (2018). Impact of Biases in Big Data. Preprint at arXiv, 10.48550/arXiv.1803.00897 10.48550/arXiv.1803.00897.

4. Blagus, R., and Lusa, L. (2013). SMOTE for high-dimensional class-imbalanced data. BMC Bioinformatics *14*, 106. 10.1186/1471-2105-14-106.

5. Kotsiantis, S., Kanellopoulos, D., and Pintelas, P. (2006). Handling imbalanced datasets: A review. 13.

6. Mohammed, R., Rawashdeh, J., and Abdullah, M. (2020). Machine Learning with Oversampling and Undersampling Techniques: Overview Study and Experimental Results. In 2020 11th International Conference on Information and Communication Systems (ICICS), pp. 243–248. 10.1109/ICICS49469.2020.239556.

7. Megahed, F.M., Chen, Y.-J., Megahed, A., Ong, Y., Altman, N., and Krzywinski, M. (2021). The class imbalance problem. Nat. Methods *18*, 1270–1272. 10.1038/s41592-021-01302-4.

8. Chawla, N.V., Bowyer, K.W., Hall, L.O., and Kegelmeyer, W.P. (2002). SMOTE: Synthetic Minority Over-sampling Technique. J. Artif. Intell. Res. *16*, 321–357. 10.1613/jair.953.

9. Fernandez, A., Garcia, S., Herrera, F., and Chawla, N.V. (2018). SMOTE for Learning from Imbalanced Data: Progress and Challenges, Marking the 15-year Anniversary. J. Artif. Intell. Res. *61*, 863–905. 10.1613/jair.1.11192.

10. Raghuwanshi, B.S., and Shukla, S. (2020). SMOTE based class-specific extreme learning machine for imbalanced learning. Knowl.-Based Syst. *187*, 104814. 10.1016/j.knosys.2019.06.022.

11. Galar, M., Fernandez, A., Barrenechea, E., Bustince, H., and Herrera, F. (2012). A Review on Ensembles for the Class Imbalance Problem: Bagging-, Boosting-, and Hybrid-Based Approaches. IEEE Trans. Syst. Man Cybern. Part C Appl. Rev. *42*, 463–484. 10.1109/TSMCC.2011.2161285.

12. Lomax, S., and Vadera, S. (2013). A survey of cost-sensitive decision tree induction algorithms. ACM Comput. Surv. CSUR *45*, 1–35.

13. Khan, S.S., and Madden, M.G. (2010). A Survey of Recent Trends in One Class Classification. In Artificial Intelligence and Cognitive Science Lecture Notes in Computer Science., L. Coyle and J. Freyne, eds. (Springer), pp. 188–197. 10.1007/978-3-642-17080-5_21.

14. Dong, X., Yu, Z., Cao, W., Shi, Y., and Ma, Q. (2020). A survey on ensemble learning. Front. Comput. Sci. *14*, 241–258. 10.1007/s11704-019-8208-z.

15. Witten, I.H., Frank, E., Hall, M.A., Pal, C.J., and DATA, M. (2005). Practical machine learning tools and techniques. In Data Mining.

16. Chai, X., Deng, L., Yang, Q., and Ling, C.X. (2004). Test-cost sensitive naive Bayes classification. In Fourth IEEE International Conference on Data Mining (ICDM'04), pp. 51–58. 10.1109/ICDM.2004.10092.

17. Drummond, C., and Holte, R.C. Exploiting the Cost (In)sensitivity of Decision Tree Splitting Criteria. 8.

18. Fumera, G., and Roli, F. (2002). Cost-sensitive learning in support vector machines. VIII Convegno Assoc. Ital. L'Intelligenza Artif.

19. Cao, P., Zhao, D., and Zaiane, O. (2013). An optimized cost-sensitive SVM for imbalanced data learning. In Pacific-Asia conference on knowledge discovery and data mining (Springer), pp. 280–292.

20. Ling, C.X., and Sheng, V.S. (2007). Comparative study of cost-sensitive classifiers. Jisuanji XuebaoChinese J. Comput. *30*, 1203–1212.

21. Oza, P., and Patel, V.M. (2019). One-Class Convolutional Neural Network. IEEE Signal Process. Lett. *26*, 277–281. 10.1109/LSP.2018.2889273.

22. Binbusayyis, A., and Vaiyapuri, T. (2021). Unsupervised deep learning approach for network intrusion detection combining convolutional autoencoder and one-class SVM. Appl. Intell. *51*, 7094–7108. 10.1007/s10489-021-02205-9.

23. May, R.J., Maier, H.R., and Dandy, G.C. (2010). Data splitting for artificial neural networks using SOM-based stratified sampling. Neural Netw. *23*, 283–294. 10.1016/j.neunet.2009.11.009.

24. Mac Namee, B., Cunningham, P., Byrne, S., and Corrigan, O.I. (2002). The problem of bias in training data in regression problems in medical decision support. Artif. Intell. Med. *24*, 51–70. 10.1016/S0933-3657(01)00092-6.

25. Sun, L., and Bull, S.B. (2005). Reduction of selection bias in genomewide studies by resampling. Genet. Epidemiol. *28*, 352–367. 10.1002/gepi.20068.

26. Gray, L. (2016). The importance of post hoc approaches for overcoming non-response and attrition bias in population-sampled studies. Soc. Psychiatry Psychiatr. Epidemiol. *51*, 155–157. 10.1007/s00127-015-1153-8.

27. Zwitter, M., and Soklic, M. (2015). University Medical Centre, Institute of Oncology, Ljubljana, Yugoslavia. UCI Mach. Learn. Repos. Irvine CA USA Available Online Httpsarchive Ics Uci Edumldatasetsbreast Cancer Accessed 1 June 2021.

28. Tu, J.V., Chu, A., Donovan, L.R., Ko, D.T., Booth, G.L., Tu, K., Maclagan, L.C., Guo, H., Austin, P.C., Hogg, W., et al. (2015). The Cardiovascular Health in Ambulatory Care Research Team (CANHEART). Circ. Cardiovasc. Qual. Outcomes *8*, 204–212. 10.1161/CIRCOUTCOMES.114.001416.

29. Azizi, Z., Lindner, S., Shiba, Y., Raparelli, V., Norris, C.M., Kublickiene, K., Herrero, M.T., Kautzky-Willer, A., Klimek, P., Gisinger, T., et al. (2023). A comparison of synthetic data generation and federated analysis for enabling international evaluations of cardiovascular health. Sci. Rep. *13*, 11540. 10.1038/s41598-023-38457-3.
