# Supplementary material for: An evaluation of synthetic data augmentation for mitigating covariate bias in health data
Source: Patterns (N Y). 2024 Feb 29;5(4):100946. doi: 10.1016/j.patter.2024.100946 (PMC11026977; doi:10.1016/j.patter.2024.100946)
Supplement: Document S2. Article plus supplemental information [file mmc2.pdf]

Patterns

Article

An evaluation of synthetic data augmentation for mitigating covariate bias in health data

Graphical abstract

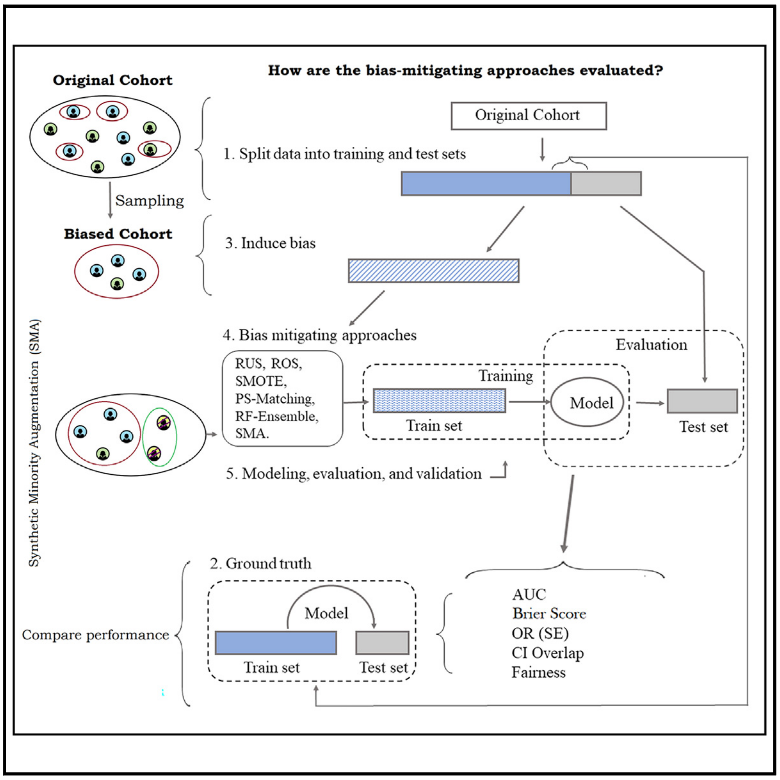

Highlights

- The study compares the efficacy of bias-mitigating approaches in real-world health data
- The synthetic data-augmentation method synthesizes the under-represented group
- Performance is evaluated based on predictive accuracy, parameter precision, and fairness
- Our method improves the performance and fairness in low to medium bias severity

Authors

Lamin Juwara, Alaa El-Hussuna, Khaled El Emam

Correspondence

kelemam@ehealthinformation.ca

In brief

The paper evaluates different methods for mitigating bias in real-world health data. Multiple bias-mitigation approaches are compared, and one based on synthetic data generation is found to address the effects of data bias for low to medium bias severity. This approach simulates additional patients from the under-represented group to augment the data and reduce the under-representation. The study's outcomes provide a method to reduce the effects of data bias on statistical model quality, accuracy, and fairness.

Juwara et al., 2024, Patterns *5*, 100946
April 12, 2024 © 2024 The Author(s).
https://doi.org/10.1016/j.patter.2024.100946

CellPress

Patterns

CellPress
OPEN ACCESS

Article

An evaluation of synthetic data augmentation for mitigating covariate bias in health data

Lamin Juwara,[1,2] Alaa El-Hussuna,[3] and Khaled El Emam[1,2,4,5,*]
[1]School of Epidemiology and Public Health, University of Ottawa, Ottawa, ON, Canada
[2]Research Institute, Children's Hospital of Eastern Ontario, Ottawa, ON, Canada
[3]Open Source Research Collaboration, Aalborg, Denmark
[4]Data Science, Replica Analytics Ltd., Ottawa, ON, Canada
[5]Lead contact
*Correspondence: kelemam@ehealthinformation.ca
https://doi.org/10.1016/j.patter.2024.100946

THE BIGGER PICTURE Data bias occurs in the sampling processes of many real-world datasets. For health data, gender and ethnicity are common factors causing sampling bias. Models built on datasets with under-represented categories will lead to inaccurate predictions and unfair decisions. To avoid such issues, bias-mitigation methods could be used to handle biased data. Techniques for generating synthetic individuals in the minority groups could augment the biased datasets and reconstruct full and unbiased datasets. If the group distributions in the samples are rebalanced, the results of the models will be generalizable to the original population.

SUMMARY

Data bias is a major concern in biomedical research, especially when evaluating large-scale observational datasets. It leads to imprecise predictions and inconsistent estimates in standard regression models. We compare the performance of commonly used bias-mitigating approaches (resampling, algorithmic, and post hoc approaches) against a synthetic data-augmentation method that utilizes sequential boosted decision trees to synthesize under-represented groups. The approach is called synthetic minority augmentation (SMA). Through simulations and analysis of real health datasets on a logistic regression workload, the approaches are evaluated across various bias scenarios (types and severity levels). Performance was assessed based on area under the curve, calibration (Brier score), precision of parameter estimates, confidence interval overlap, and fairness. Overall, SMA produces the closest results to the ground truth in low to medium bias (50% or less missing proportion). In high bias (80% or more missing proportion), the advantage of SMA is not obvious, with no specific method consistently outperforming others.

INTRODUCTION

There is a wide range of biases that can affect datasets,[1] with data bias being notably common in biomedical research.[2–5] In the statistical learning literature, a training sample is said to be biased if its covariate distributions differ from those of the original population.[6,7] This results in the under-representation of specific covariate categories or distributions (as opposed to outcome imbalance, which constitutes a different problem). Unlike in a randomized study where participants are assigned to treatment groups to ensure a balanced dataset,[8,9] observational datasets are prone to covariate biases.[10–12]

Covariate bias often stems from selection bias and is introduced at the data-collection or analysis stage, where some participants are systematically excluded from the analytical dataset.[13] Among the numerous selection-type biases, sampling bias is the most prominent and is sometimes used interchangeably with selection bias in the literature.[14] Sampling bias results in a sampled dataset that does not reflect the study population and, therefore, models built on such data are difficult to generalize.[15] While sampling biases are most often attributed to gender and ethnic factors, they can also result from the lack of capture and inclusion of social determinants of health, including geographical and socioeconomic conditions, such as age and place of birth, which drive outcome categorization.[16] Henceforth in this article, the presence of sample selection bias in the sampled cohorts is referred to as "data bias."

Data bias is encountered in many real-world applications, including medical diagnosis,[3] image or facial recognition,[17,18] text classification,[19] and speech recognition.[20,21] It can manifest

This is an open access article under the CC BY license (http://creativecommons.org/licenses/by/4.0/).

in various analytical settings such as comparative, predictive, and diagnostic scenarios[11] where it presents substantial moral and ethical implications.[22] Some examples include algorithms for popular online recommender apps built on datasets in which female participants were under-represented, which in turn gave wrong recommendations to female users.[23] In a recent study, researchers showed that a widely used algorithm (to allocate health care to patients) in United States (US) hospitals was less likely to refer black people than white people who were equally sick to programs that aim to improve care for patients with complex medical needs.[24] It was found that bias was introduced in the training set during data collection when patients who self-identified as black were on average assigned lower pain scores than other groups. Many other examples of gender- and race-induced biases in covariate distributions have been highlighted in the literature.[3,4] These biases might sometimes lead to algorithmic bias, which has received a lot of attention in recent years, especially in the context of personalized recommender systems.[25,26] Notable examples include racial biases in algorithms used in US court systems to predict the likelihood of repeated offenses[27] and gender biases in Amazon's hiring software.[28]

In trained models, data bias not only affects the effect estimates of the predictor-outcome association or makes true associations statistically non-significant,[29] it also reduces the sample precision and, as a result, the prediction accuracy.[30,31] Thus, results obtained from models built on biased samples are typically not generalizable to the original population.[16,32–34] Another challenge with analyzing datasets with data bias is the degraded performance on standard performance metrics.[35] For example, biased samples can be misleading in that they can sometimes produce higher predictive accuracy results than the ground truth.[36]

In recent years, several approaches have been proposed for mitigating the impact of data bias. These approaches cover biases in fields such as healthcare data from clinician interactions, hiring and promotion,[37] imaging,[38] text classification,[39] and transportation,[40] among others. In particular, rebalancing schemes such as random undersampling (RUS) and random oversampling (ROS) are quite attractive, as they allow researchers to reconstruct balanced datasets a priori before any modeling effort. Although such traditional schemes have been shown to perform well in many settings, they are not always effective, especially when the source of bias is complex or the bias severity is too high. Various data-augmentation methods have also been proposed to mitigate biases in image recognition,[41] speech,[42] and vision.[43] However, they are often model specific, requiring large training cohorts, and might even give a false sense of sampling unseen data from the same underlying distribution as the original training data.[44]

In this paper, we compare a representative set of bias-mitigation approaches for structured tabular health data and propose and evaluate an approach based on synthetic data generation. The contributions of this work are 3-fold.

(1) Evaluate various data bias-mitigation approaches that can be used to correct for covariate bias in health datasets when applied to binary classification tasks.
(2) Describe a bias-mitigation method based on synthetic data-generation techniques to generate synthetic copies of the under-represented category to augment the biased dataset and reconstruct full and unbiased data samples.
(3) Compare the statistical model performance and fairness of the proposed method to the traditional approaches through simulations and applications to four real datasets.

BACKGROUND

Terminology

For biased datasets with a categorical covariate that is unevenly distributed, we will refer to that covariate as the biased covariate. The under-represented category of the biased covariate is termed the minority group, and the other categories are the majority group. The degree of imbalance is determined by the ratio of the minority to the majority group. The extent of bias is with reference to the baseline distribution, which may be the population distribution or a proxy for it. For example, the biased covariate can be "sex," with "female" being the minority group, "male and other" being the majority group, and the baseline distribution in the population being 50% female.

Current bias-mitigation methods

The current methods for mitigating data bias can broadly be classified into three categories: rebalancing approaches, algorithmic approaches, and post-processing approaches. We present a brief description of some of the approaches below and refer the reader to Appendix A in supplemental experimental procedures for a more detailed review of the ones that we apply in our study.

Rebalancing approaches attempt to construct datasets that are reflective of the true population. Starting with the biased data, the goal of rebalancing is to produce a balanced dataset that provides a good approximation of the underlying population data. These approaches can take the form of subsampling (e.g., RUS, ROS, and SMOTE [synthetic minority oversampling technique]) or matching (e.g., propensity score [PS] matching).

Among the subsampling approaches, RUS, ROS, and SMOTE have gained a lot of traction in recent years. RUS is a data preprocessing approach to rebalance unevenly distributed groups in imbalanced datasets by randomly removing excess observations in the majority group of the biased covariate.[45] In the current context, the approach is adopted for rebalancing the group distributions in categorical covariates in biased datasets.[3] ROS, in contrast, attempts to rebalance biased covariate distributions by repeating the entries of randomly sampled observations of the minority group. The resulting cohort is a larger dataset that is composed of repeated observations and well-represented covariate distributions.[46] While ROS has the advantage of improving the stability and performance of learning algorithms applied to the dataset by mitigating convergence issues, it is often argued that the duplicated observations contribute minimally to the dataset's richness or diversity, potentially leading to overfitting.[47] An alternative approach to replicating instances of the minority group is to generate synthetic copies through interpolation among neighboring minority instances. This approach is termed SMOTE.[48] Unlike ROS, the main appeal of SMOTE is that it adds new plausible observations that are sampled from the neighborhood of the minority group and not simply repeated entries.

Table 1. Summary of the real datasets that were used

Dataset		Description	Outcome	Biased covariate	Conditioning covariate
1	Canadian Community Health Survey (CCHS)	63,522 observations	cardiovascular health	gender (female = 45.2%)	CBI: new immigrants
–	–	8 variables	–	–	CBII: marital status
2	N0147 colon cancer	1,543 observations	death	bowel obstruction (No = 83.8%)	CBI: gender
–	–	10 variables	–	–	CBII: BMI
3	Danish Colon Cancer data (DCCG)	12,855 observations	postoperative complications	gender (female = 55.9%)	CBI: P-PN stage
–	–	192 variables (total)	–	–	CBII: ASA
–	–	9 selected	–	–	–
4	Breast cancer	277 observations	BC class	age (20–49 = 45.5%)	CBI: left/right breast
–	–	10 variables	–	–	CBII: menopause

CBI, conditional bias I; CBII, conditional bias II.

Another rebalancing approach that takes place at the modeling stage is the use of matching. PS adjustment can take place in the form of matching, stratification, or regression (covariance) adjustment. While matching and stratification are usually applied to the dataset before statistical modeling to construct balance and appropriate comparisons among the baseline covariates, adjustment with PSs is employed at the analyses stage by including the scores (as weights) directly into the regression model. In this article, we restrict our evaluation to PS adjustment to rebalance the biased data at the analysis stage.

Algorithmic methods mitigate the problem of covariate imbalance by targeting the learning stage of the analyses. The category encompasses approaches that adapt commonly used learning algorithms to reweight models for learning from the

Table 2. Simulation results across 500 iterations for the full model

		Marginal bias			Conditional bias I			Conditional bias II		
Proportion	Approach	AUC	OR_Z (SD)	I_Z	AUC	OR_Z (SD)	I_Z	AUC	OR_Z (SD)	I_Z
15%	Biased data	0.72	1.30 (0.15)	–	0.72	1.30 (0.16)	–	0.72	1.30 (0.15)	–
	RUS	0.72	1.28 (0.15)	0.98	0.72	1.28 (0.16)	0.94	0.71	1.28 (0.15)	0.95
	ROS	0.72	1.27 (0.14)	0.98	0.72	1.29 (0.14)	0.93	0.72	1.27 (0.14)	0.94
	SMOTE	0.71	1.14 (1.18)	0.74	0.69	1.19 (1.18)	0.88	0.70	1.18 (1.04)	0.84
	PS match	0.72	1.19 (0.16)	0.92	0.72	1.18 (0.16)	0.92	0.72	1.23 (0.16)	0.96
	RF ensemble	0.70	–	–	0.69	–	–	0.69	–	–
	SMA[a]	0.72	1.25 (0.14)	0.98	0.72	1.27 (0.14)	0.95	0.72	1.26 (0.14)	0.95
50%	Biased data	0.71	1.49 (0.19)	–	0.72	1.31 (0.15)	–	0.72	1.43 (0.19)	–
	RUS	0.70	1.38 (0.14)	0.85	0.72	1.30 (0.14)	0.89	0.71	1.41 (0.20)	0.84
	ROS	0.71	1.45 (0.16)	0.78	0.72	1.29 (0.16)	0.90	0.71	1.51 (0.19)	0.82
	SMOTE	0.68	0.94 (0.21)	0.71	0.69	1.02 (0.21)	0.49	0.68	0.91 (0.18)	0.41
	PS match	0.70	1.44 (0.26)	0.72	0.71	1.32 (0.13)	0.95	0.70	1.45 (0.24)	0.78
	RF ensemble	0.69	–	–	0.68	–	–	0.70	–	–
	SMA[a]	0.71	1.24 (0.19)	0.93	0.71	1.29 (0.13)	0.90	0.72	1.39 (0.14)	0.88
80%	Biased data	0.68	1.00 (0.25)	–	0.71	1.32 (0.19)	–	0.70	1.09 (0.22)	–
	RUS	0.68	1.17 (0.28)	0.97	0.71	1.41 (0.21)	0.72	0.70	1.03 (0.27)	0.76
	ROS	0.68	1.02 (0.24)	0.98	0.72	1.28 (0.24)	0.75	0.70	0.97 (0.23)	0.84
	SMOTE	0.67	0.73 (0.21)	0.34	0.69	1.05 (0.20)	0.60	0.70	0.90 (1.15)	0.33
	PS match	0.65	0.50 (0.95)	0.84	0.70	1.54 (0.15)	0.81	0.69	1.08 (0.40)	0.66
	RF ensemble	0.66	–	–	0.68	–	–	0.69	–	–
	SMA[a]	0.69	0.96 (0.51)	0.98	0.70	1.33 (0.24)	0.77	0.70	1.24 (0.16)	0.91

Estimates of the mean AUC, mean odds ratio associated with Z and standard deviation (OR_Z (SD)), and the confidence interval overlap of the biased covariate effect with the ground truth (I_Z) from 500 repetitions. Proportion indicates the proportion of observations removed under each bias setting. For the original data: AUC = 0.72; OR_Z (SD) = 1.25 (0.15); I_Z = 1.00. Only results for missing proportions of 15%, 50%, and 80% are shown.
[a]Estimates are averaged from m = 100 synthetic copies.

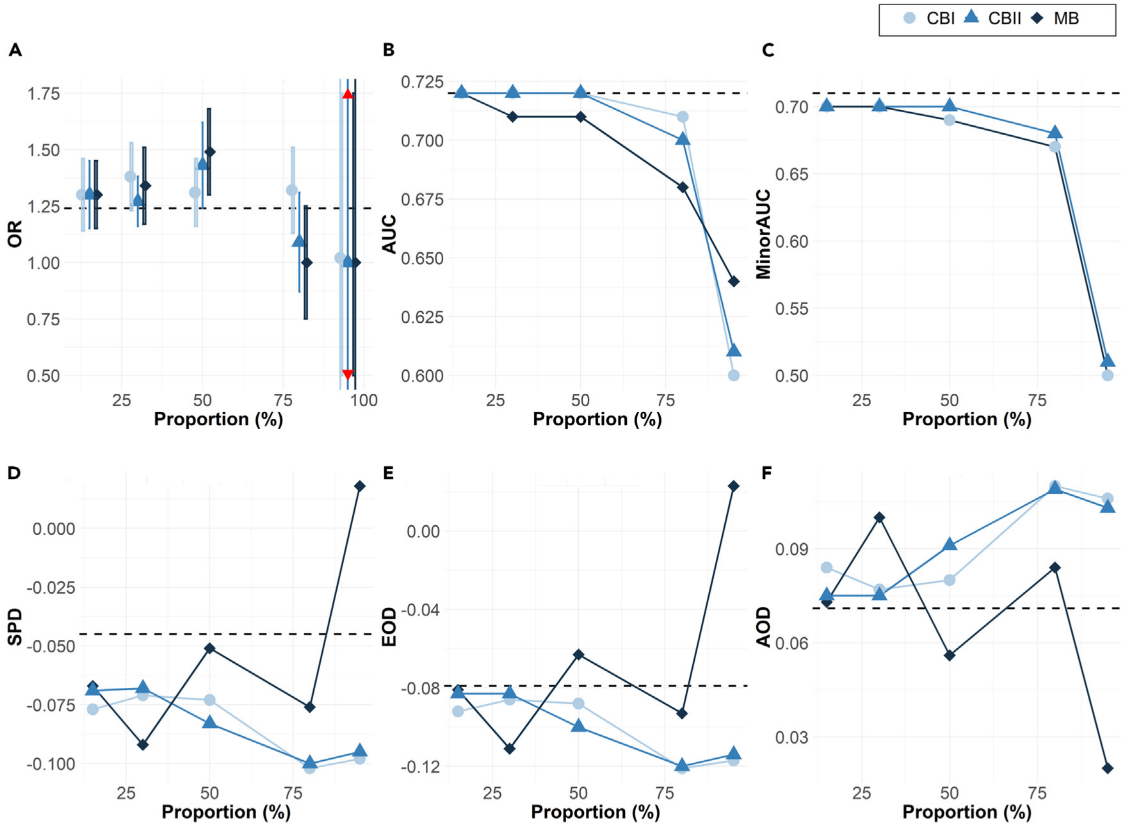

Figure 1. Bias type

Simulations results across 500 iterations for the full model showing the impact of bias (increasing missing proportion) on (A) odds ratio (OR) and the standard deviation of the biasing covariate across simulation runs, (B) the overall area under the curve (AUC), (C) minority-group AUC, and fairness metrics: (D) statistical parity difference (SPD), (E) equal opportunity difference (EOD), and (F) average odds difference (AOD). The dashed line represents the ground truth from the original data.

minority group of the biased data. These approaches can broadly be grouped into ensemble-based learning,[49] cost-sensitive learning,[50] and single-class learning.[51]

In this article, we use the random forest (RF) ensemble approach presented by Dong et al.[52] to handle biased data. Their approach incorporates modifications to the RF algorithm, enabling it to draw inferences from highly biased datasets. For example, the model initially takes bootstrap samples from the minority group, followed by an equal number of samples from the majority group, thus balancing the groups. The technique is highly regarded for its capacity to merge the advantages of multiple learners, enhancing overall performance. Additionally, RF ensembles have demonstrated their effectiveness in addressing biased data due to their innate ability to balance group distributions and reduce overfitting.

When the effect of data bias cannot be addressed during preprocessing (e.g., data cleaning) or the modeling stage, it is sometimes possible to employ post hoc adjustment techniques to account for the effect of learning from biased data. For example, in genetic studies of gene-based rare variant associations, bootstrap resampling approaches are often employed to adjust for bias in single-marker tests on common variants in genome-wide association studies.[53] In most cases, post hoc adjustments are only recommended as last-resort options when bias could not be mitigated in the early stages of the study.[54]

RESULTS

This section presents the performance results for the full model and the stratified model across the biased datasets and the bias-mitigated datasets.

Simulation results

In Table 2, we show the simulation results averaged across the 500 iterations. These are shown for some of the bias proportions only to illustrate trends, and the full results are given in Appendix B in supplemental experimental procedures.

Impact of bias

As the proportion of observations excluded is increased from 15% to 95% (Figure 1), the discrepancy between the biased covariate parameter estimates on the biased samples and that of the ground truth also increases.

Relative to the ground-truth odds ratio of the biasing predictor Z (OR_Z = 1.25), the OR_Z estimates of the biased samples increase from 1.30 to 1.49 in the low to moderate bias setting (missing proportion of 50% or less) before rapidly decreasing toward the null (OR_Z = 1) in high to extreme bias setting (missing proportion of 80% or more).

The areas under the curve (AUCs) of the biased datasets decrease consistently from the ground truth with increasing

CellPress
OPEN ACCESS

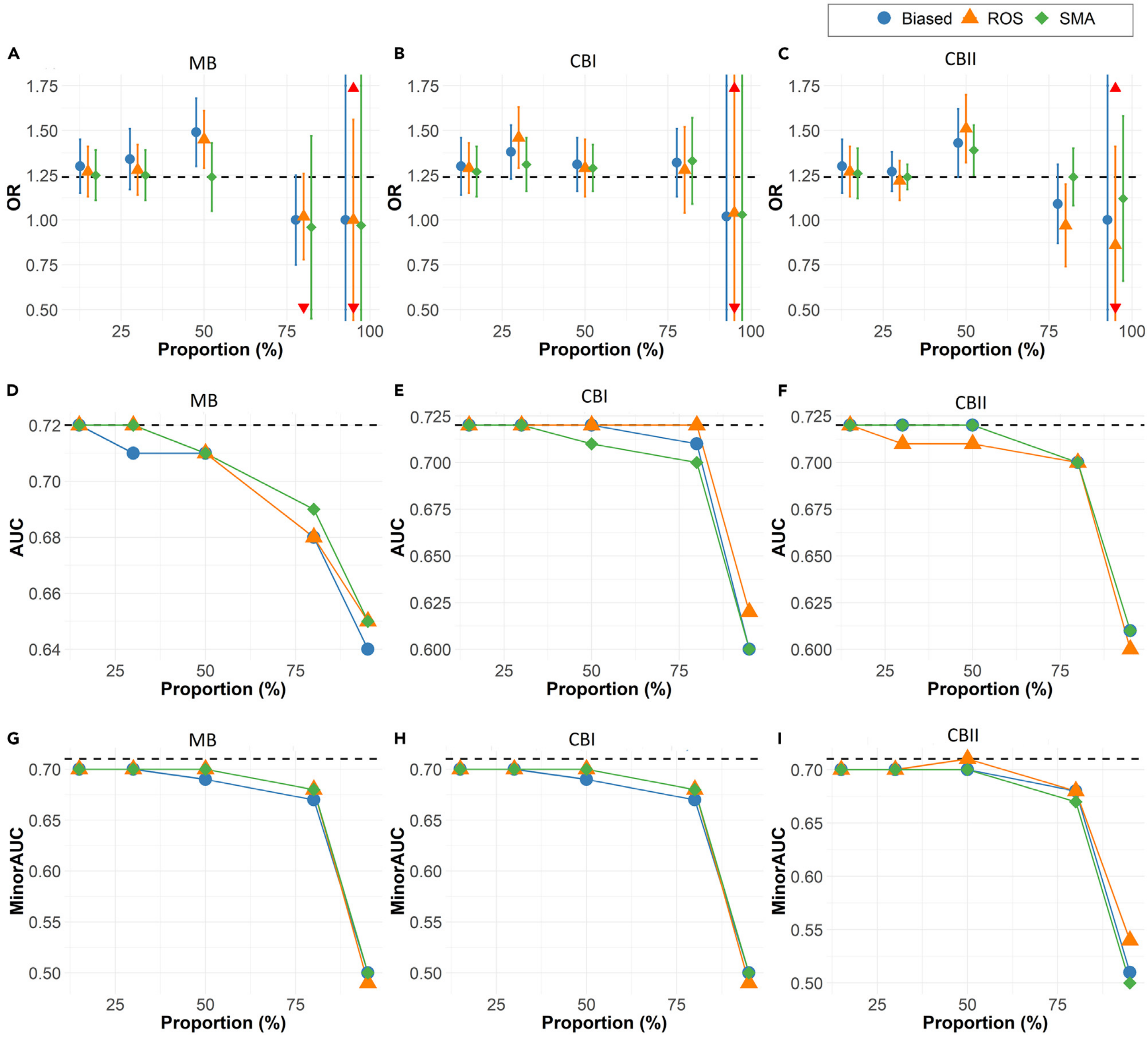

Figure 2. Bias mitigation approach

Simulations results across 500 iterations for the full model showing the comparisons of the OR estimates and the standard deviation of the biasing covariate across simulation runs (A–C), overall model AUCs (D–F), and minority-group AUC of the biasing covariate (G–I) for synthetic minority augmentation (SMA), random oversampling (ROS, the best-performing alternative), and biased data. The dashed line represents the ground truth from unbiased data.

missing proportions under marginal bias and decline rapidly for all types of high or extreme bias (Figure 1B). Data bias is more impactful on the performance of the category affected by the missing observations. As more observations are excluded from the minority group, the impact on minority AUC also grows (Figure 1C).

As the extent of bias grows, the three types of fairness metrics increasingly deviate from the ground-truth value, with the greatest deviation at the highest bias (Figures 1D–1F).

Performance of SMA relative to ground truth

The performance of synthetic minority augmentation (SMA) is shown relative to ROS in Figure 2, as this was the best-performing alternative bias-mitigation approach in the simulations.

Unlike alternative approaches (RUS, ROS, SMOTE, RF ensembles, and PS matching), SMA produces OR estimates that are consistently comparable to the ground truth in low to moderate bias scenarios. As the severity of bias increases, SMA continues to offer OR estimates that are more comparable to the ground truth than the other approaches. For high to extreme bias, the results are not always conclusive (Figures 2A–2C).

The parameter estimates corresponding to β_Z= (log(0.5),log(1.01),log(2)) and AUCs are presented in Tables S7 and S8 to show the robustness of the approaches under negative associations, approximately null associations, and large positive associations, respectively. In all cases, the results are consistent with the primary analysis results.

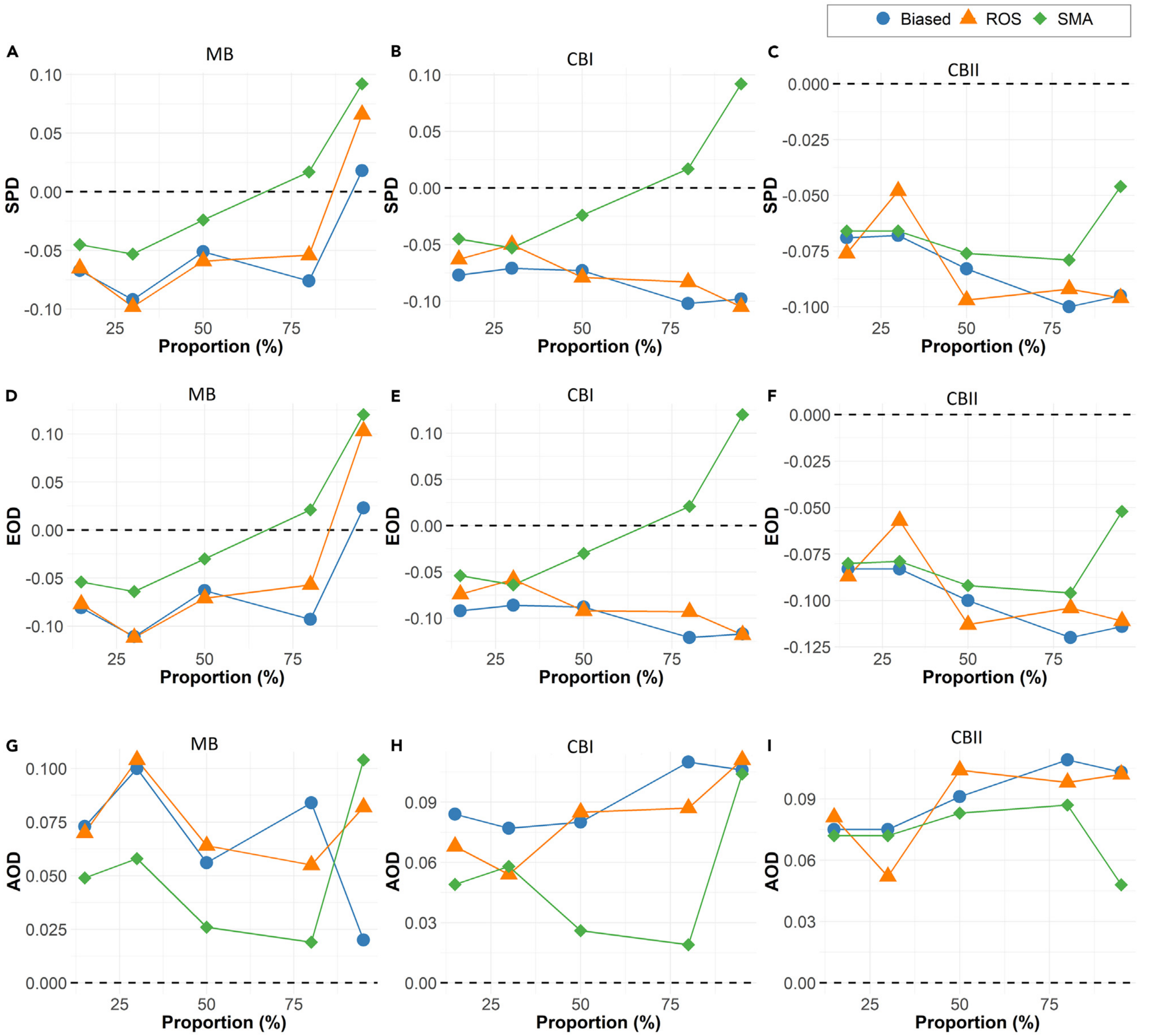

Figure 3. Bias mitigation approach

Simulation results across 500 iterations for the full model showing the fairness estimates based on (A–C) statistical parity difference (SPD), (D–F) equal opportunity difference (EOD), and (G–I) average odds difference (AOD). The estimates are reported for biased data, ROS (the best-performing alternative), and SMA. The dashed line represents the optimal fairness value under no data bias.

Up to moderate bias, SMA has AUC closer to the ground truth than ROS. At higher levels of bias, the results on the best approach for AUC are not conclusive (Figures 2D–2F).

The minority-group AUC results in Figure 2 demonstrate that SMA consistently produces values comparable to the ground truth in low to moderate bias. In cases of extreme data bias (missing proportion of 95% or more), no method can ensure the best accuracy. Compared to other methods, SMA performs to the same degree or better on the Brier scores of the minority group (Table S5).

The confidence interval overlap for SMA also consistently outperforms other approaches, especially in low to moderate bias samples. These results are shown in Table 2.

In Figure 3, the fairness measures (statistical parity difference [SPA], equal opportunity difference [EOD], and average odds difference [AOD]) indicate that SMA has fairness that is generally closer to the ground truth in comparison to other methodologies. Also, SMA tends to have better fairness than the ground truth when it does deviate from it.

In summary, the simulations demonstrate that for low to medium bias SMA yields datasets with the best parameter estimation results (more precise effect estimates, standard errors closer to the ground truth, and larger confidence interval overlap). In terms of predictive modeling (AUC and minority-group AUC estimates), SMA had values consistently close to the ground truth and comparable to the best competitive alternatives. Furthermore, the

Table 3. Estimates of mean AUC, odds ratio of Z (OR_Z (SE)), and interval overlaps (I_Z) of the biased covariate Z for each bias-mitigating approach on the CCHS data

		Marginal bias			Conditional bias I			Conditional bias II		
CCHS data proportion	Approach	AUC	OR_Z (SE)	I_Z	AUC	OR_Z (SE)	I_Z	AUC	OR_Z (SE)	I_Z
15%	Biased data	0.70	1.57 (–)	–	0.70	1.57 (–)	–	0.70	1.58 (–)	–
	RUS	0.70	1.58 (0.03)	0.94	0.70	1.58 (0.03)	0.94	0.70	1.57 (0.02)	0.97
	ROS	0.70	1.57 (0.02)	0.97	0.70	1.57 (0.02)	0.95	0.70	1.58 (0.02)	0.97
	SMOTE	0.70	1.52 (0.04)	0.77	0.70	1.56 (0.04)	0.76	0.70	1.57 (0.04)	0.78
	PS matching	0.69	1.50 (0.03)	0.56	0.69	1.50 (0.03)	0.53	0.69	1.54 (0.03)	0.73
	RF ensemble	0.70	–	–	0.70	–	–	0.70	–	–
	SMA[a]	0.70	1.57 (0.03)	0.97	0.70	1.57 (0.03)	0.94	0.70	1.58 (0.03)	0.97
50%	Biased data	0.70	1.61 (–)	–	0.69	1.63 (–)	–	0.69	1.61 (–)	–
	RUS	0.70	1.59 (0.03)	0.83	0.70	1.61 (0.03)	0.81	0.70	1.60 (0.03)	0.82
	ROS	0.70	1.59 (0.02)	0.87	0.70	1.61 (0.02)	0.77	0.70	1.61 (0.02)	0.74
	SMOTE	0.68	1.85 (0.02)	0.66	0.69	1.89 (0.02)	0.00	0.69	1.85 (0.02)	0.00
	PS matching	0.69	1.44 (0.04)	0.29	0.69	1.49 (0.04)	0.60	0.69	1.57 (0.03)	0.89
	RF ensemble	0.69	–	–	0.69	–	–	0.68	–	–
	SMA[a]	0.70	1.59 (0.02)	0.89	0.70	1.59 (0.02)	0.90	0.70	1.59 (0.02)	0.90
80%	Biased data	0.69	1.66 (–)	–	0.69	1.66 (–)	–	0.69	1.61 (–)	–
	RUS	0.70	1.60 (0.05)	0.75	0.70	1.60 (0.05)	0.75	0.70	1.61 (0.03)	0.77
	ROS	0.70	1.65 (0.02)	0.59	0.70	1.65 (0.02)	0.49	0.70	1.62 (0.02)	0.67
	SMOTE	0.68	1.82 (0.03)	0.00	0.69	1.81 (0.03)	0.00	0.69	1.79 (0.02)	0.00
	PS matching	0.70	1.50 (0.07)	0.67	0.69	1.50 (0.07)	0.67	0.69	1.49 (0.03)	0.49
	RF ensemble	0.69	–	–	0.69	–	–	0.68	–	–
	SMA[a]	0.70	1.56 (0.02)	0.78	0.70	1.57 (0.02)	0.82	0.70	1.56 (0.02)	0.80

For the original data: AUC = 0.70; OR_Z (SE) = 1.58 (0.02); I_Z = 1.00. Only results for missing proportions of 15%, 50%, and 80% are shown.
[a]Estimates are averaged from m = 100 synthetic copies.

fairness-related simulation findings indicate that SMA typically generates models exhibiting the most favorable fairness metrics.

Performance of SMA relative to biased data

As seen in Figures 2 and 3, the SMA method is an improvement over the biased dataset across all metrics and all types of bias. The OR values are closer to the ground truth with the application of SMA, as are the AUC and the minority AUC. The fairness metrics are also consistently closer to the ground truth than the biased dataset.

Results for real datasets

The results of the approaches evaluated on the Canadian Community Health Survey (CCHS) data are presented in Table 3. These are shown for selected bias proportions only to illustrate trends, while the full results are given in Appendix D in supplemental experimental procedures. The results for the remaining datasets, which show similar patterns, are also presented in the same appendix.

Impact of bias

As shown in Figure 4, the results for the impact of bias on the CCHS dataset are similar to what was observed in the simulations. As the proportion of observations excluded is increased from 15% to 95%, the discrepancy between the biased covariate estimates on the biased samples and that of the ground truth also increases.

Compared to the ground truth for CCHS data (odds ratio, OR_Z = 1.55), the estimated OR_Z of the biased data increases from 1.57 to 1.66 in the marginal case, similarly from 1.57 to 1.66 in the conditional case I, and from 1.58 to 1.61 in conditional case II. For the remaining datasets presented in Appendix D, a similar pattern of increasing deviations from the ground-truth estimates is observed.

The AUCs of the biased datasets consistently decline away from the ground truth with increasing missing proportions under marginal bias and decline rapidly for all types of high or extreme bias. Bias is generally most impactful on the performance of the category affected by the missing observations. As more observations are excluded from the minority group, minority AUC decreases and the deviation from the ground truth grows.

As the extent of bias grows, the three types of fairness metrics increasingly deviate from the ground-truth value, with the greatest deviation at the highest bias.

Performance of SMA relative to ground truth

As shown in Figure 5 with the CCHS dataset results, as the bias is increased from 15% to 80%, the OR of SMA is closer to the ground truth compared to ROS, which is the best-performing alternative approach (the detailed result trends for the other approaches are shown in Table 3) for low to medium bias. The results for AUC show that both SMA and ROS are consistently close to the ground truth as bias increases.

Note that a generative model can increase the diversity of the datasets that are generated, which is the case for SMA. This means that prognostic models trained on these types of augmented datasets can perform better on unseen cases.

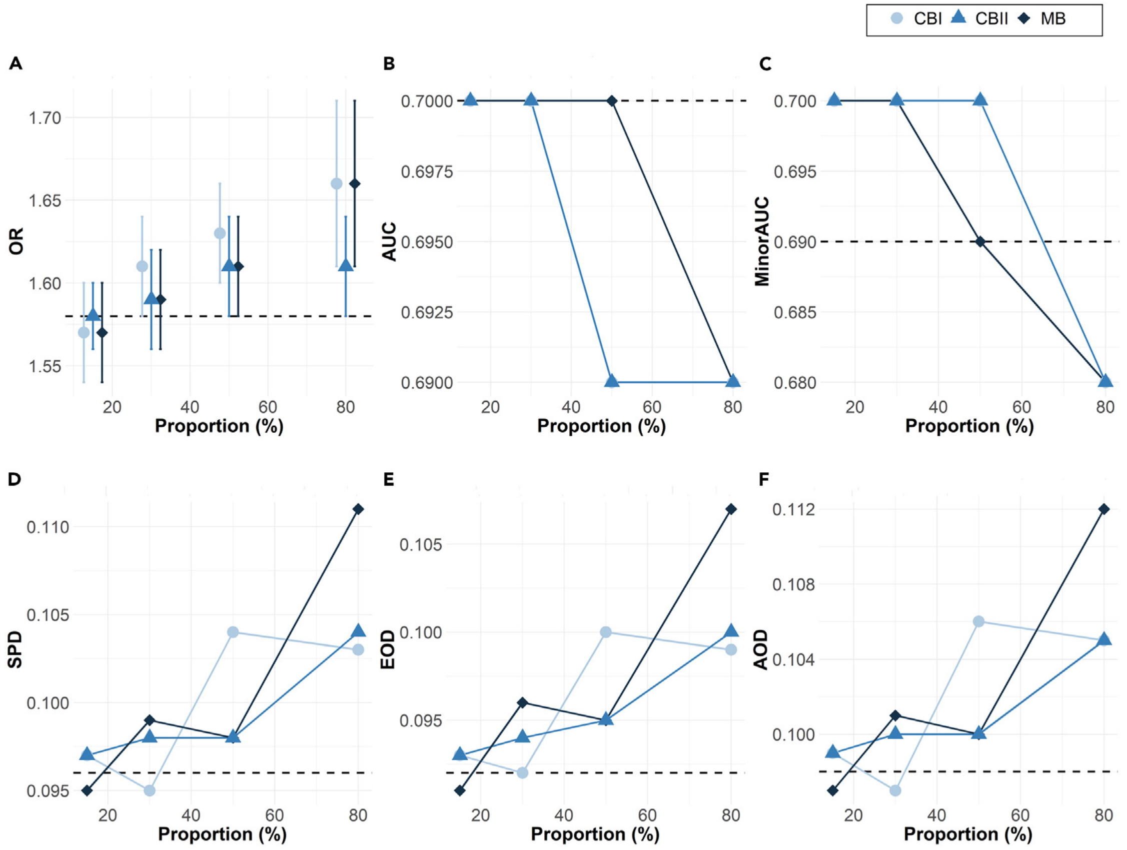

Figure 4. Bias type

Impact of bias (increasing missing proportion) on the CCHS data in evaluating the (A) odds ratio (OR) and standard error of the biasing covariate, (B) the overall AUC, (C) minority-group AUC, and three fairness metrics: (D) statistical parity difference (SPD), (E) equal opportunity difference (EOD), and (F) average odds difference (AOD). The bias is assessed under marginal bias (MB), conditional bias I (CBI), and conditional bias II (CBII). The dashed line represents the ground-truth results in the original dataset.

Consequently, we did not treat an improvement in minority AUC relative to the ground truth as a problematic deviation.

We see similar results for the minority-group AUCs under marginal and conditional bias settings. For low to medium bias, SMA performs well relative to ROS. In high to extreme cases of bias, the results are less clear.

Additionally, in Table 3 we see that the confidence interval overlaps show that SMA offers the greatest overlap with the ground-truth estimate.

In evaluating fairness, we present estimates of fairness metrics evaluated (1) SPD, (2) EOD, and (3) AOD in Figure 6. The findings indicate that SMA generates fairness estimates that are best aligned with ground-truth estimates, as does ROS to some degree. Fairness estimates for the remaining datasets are shown in Tables S10, S13, and S16).

Performance of SMA relative to biased data

A summary of the relative performance of the bias-mitigating approaches over biased datasets (using the original cohort as the reference) is presented in Figures 7, 8, 9, and 10 for all four datasets. Specifically, we show comparisons of their relative performances based on AUCs, OR estimates, the minority-group AUC, and fairness metrics across all real datasets and levels of bias, demonstrating that SMA consistently outperforms other approaches across multiple scenarios (with missing proportions ranging from 15% to 80% for each of the three bias settings). These results are more pronounced for low to medium bias, and these are shown in Appendix D.5 in supplemental experimental procedures.

Of note, the relative performances of the AUCs and ORs are estimated by comparing the differences between the estimates from each approach and the ground truth against the corresponding difference between the biased data estimates and the ground truth. For example, if the ground-truth AUC (AUC^g) is estimated as 0.85, the biased data AUC (AUC^b) as 0.80, and the AUC for SMA (AUC^{SMA}) as 0.82, then we say the AUC improved for SMA since $|AUC^g - AUC^{SMA}| < |AUC^g - AUC^b|$. Similarly, we say the AUC worsened if $|AUC^g - AUC^{SMA}| > |AUC^g - AUC^b|$ or that there is no change if the absolute differences are equal. The same convention was applied to the Brier scores, the OR estimates, and the minority-group AUCs.

For fairness, the definition is modified to include closeness to the ground-truth estimate or perfect fairness (SPD = 0, EOD = 0, AOD = 0). Hence, the fairness of an approach is considered improved if the SPD, EOD, and AOD estimates are closer to the ground-truth estimates or zero. Conversely, fairness is said to

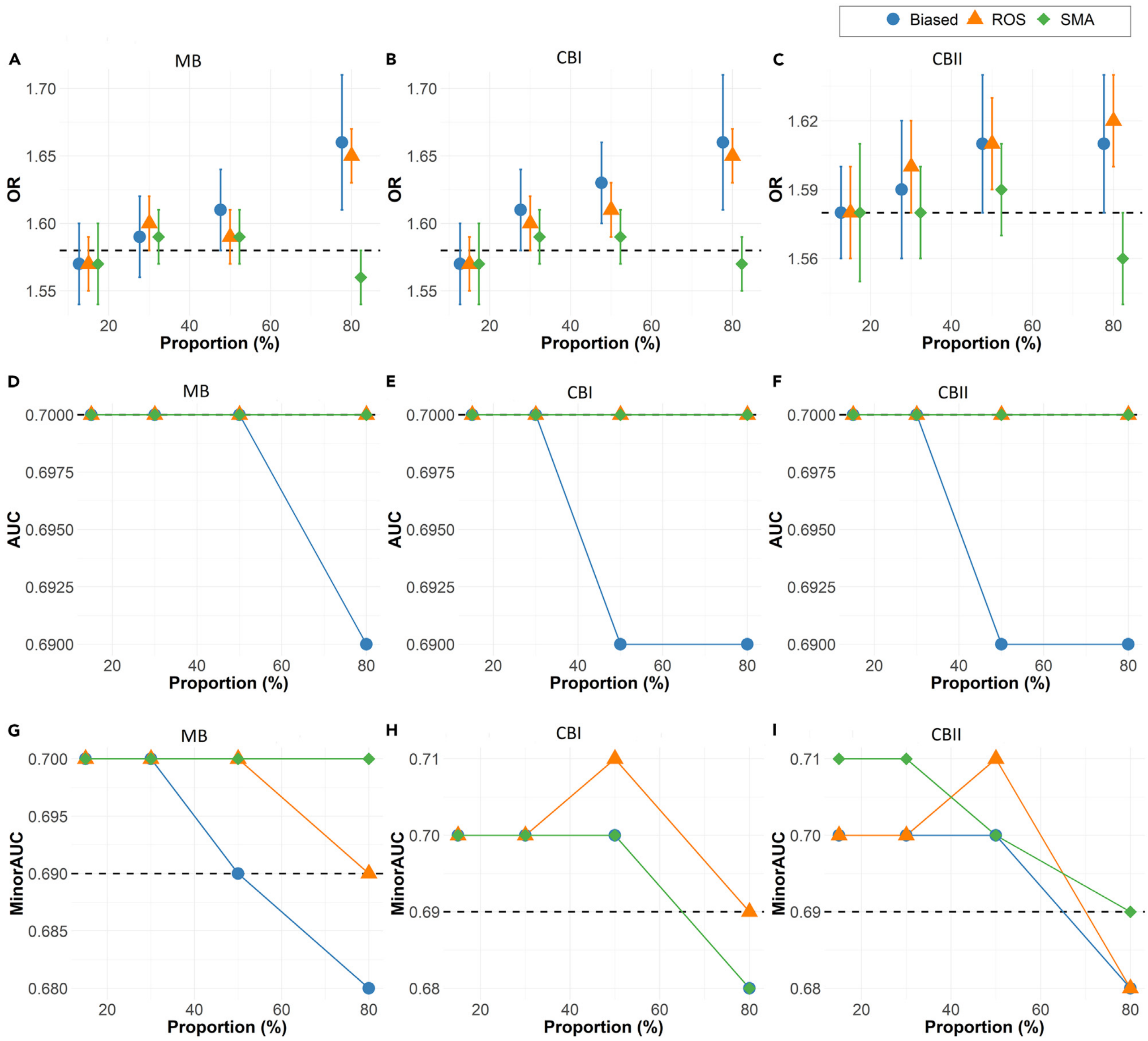

Figure 5. Bias mitigation approach

Comparisons on the CCHS dataset of the OR estimates with and standard error of the biasing covariate (A–C), overall model AUCs (D–F), and minority-group AUC of the biasing covariate (G–I) for SMA, random oversampling (ROS, the best-performing alternative), and biased data. The dashed line represents the ground truth from unbiased data.

have worsened if the estimate is not closer to the ground truth and not closer to zero.

We also independently generated the corresponding summary plots for (1) low to medium bias and (2) high bias for each of the metrics evaluated, which are provided in Appendix D in supplemental experimental procedures.

DISCUSSION

Summary

Learning from biased data presents a challenging task in biomedical research. The literature lists multiple modeling, prediction, and generalization problems with biased datasets and raises ethical questions about the application of models trained on biased datasets. As a result, prognostics and inferences done on such datasets are not always useful in clinical settings.

In this article, we first evaluated the impact of bias on analytical results. Our results indicated the following.

(1) The parameter estimates in logistic regression models increasingly deviate from the ground truth as the extent of bias grows and becomes evident even at low to moderate levels of data bias. This is the case for different types of bias (marginal and conditional) and is a consistent finding in simulations and on real datasets.
(2) Biased data demonstrate a reduction in fairness metrics, and this grows monotonically with the extent of bias

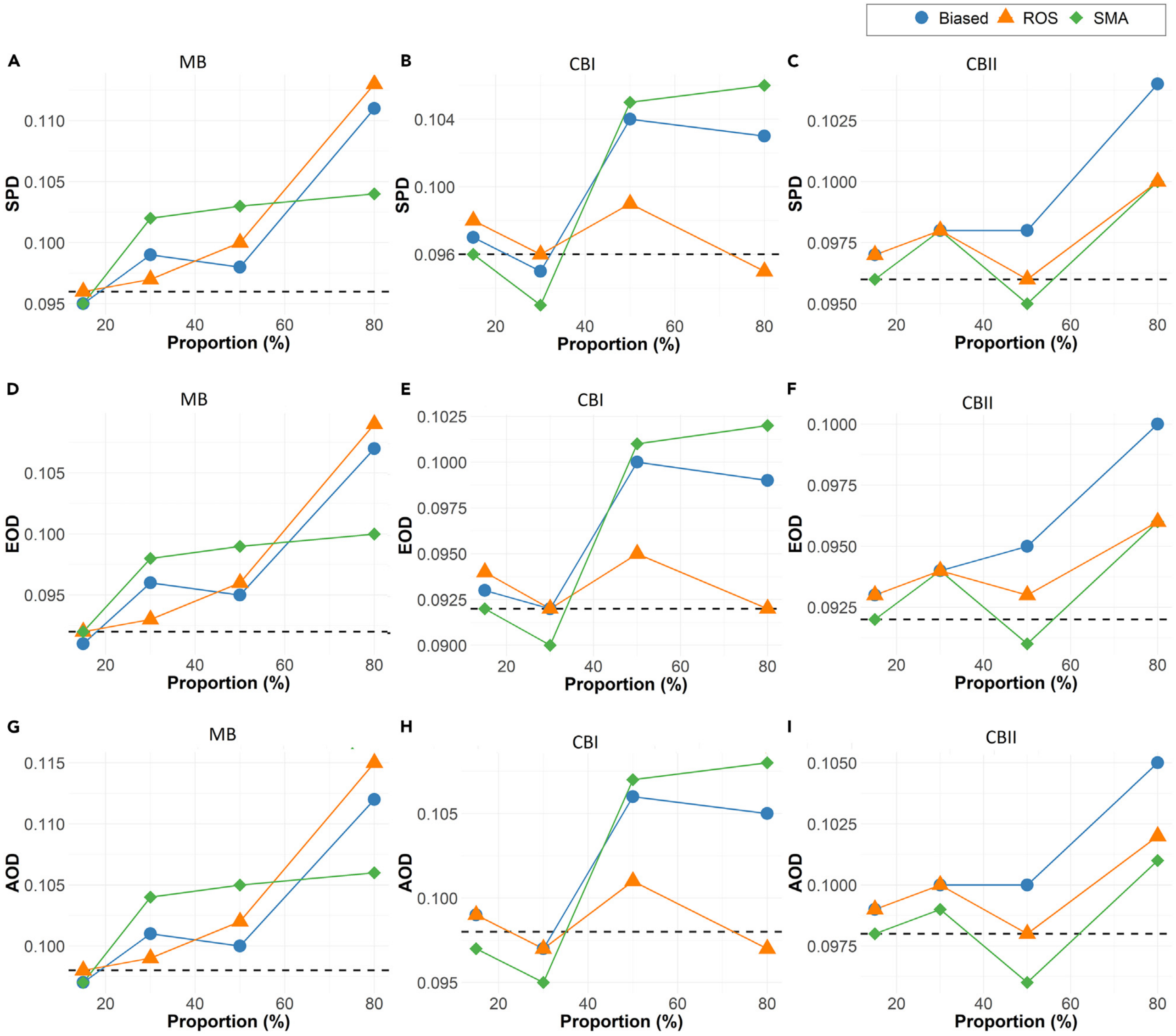

Figure 6. Bias mitigation approach

Fairness metrics on the CCHS dataset: (A–C) the statistical parity difference (SPD), (D–F) equal opportunity difference (EOD), and (G–I) average odds difference (AOD) from the logistic regression model on the cardiovascular health data. The proportion of samples removed varied from 15% to 80%. Original cohort: SPD = 0.096, EOD = 0.092, and AOD = 0.098. The symbol ǂ indicates that the estimates are averaged from m = 100 synthetic copies. The dashed line represents the ground-truth fairness in the original dataset.

induced on the datasets. This is to be expected as the fairness metrics evaluate bias directly.

(3) Model calibration as assessed using the Brier score is not affected materially by data bias, and these results are consistent even for high rates of bias.
(4) Overall prediction model accuracy decreases when bias is high or extreme, and the minority-group AUC was better than the ground truth for low to medium bias but decreased as bias grows.

We proposed a bias-mitigation approach called SMA. The method augments the biased datasets with synthetically generated copies of the minority group to reconstruct the full bias-mitigated dataset. This proposed method was compared to standard approaches such as RUS, ROS, SMOTE, RF ensembles, and PS matching. Our results through simulations and real datasets on a logistic regression workload indicated that:

(1) SMA has consistently higher confidence interval overlap with the ground truth on the biased covariate parameter.
(2) ciFor low to medium levels of bias, the SMA gave the best performance across methods evaluated in terms of the precision of the parameter estimate, the overall model AUC, and fairness for mitigating the effects on parameter estimates by having the closest values (or better values for fairness) to the ground truth. While SMA sometimes

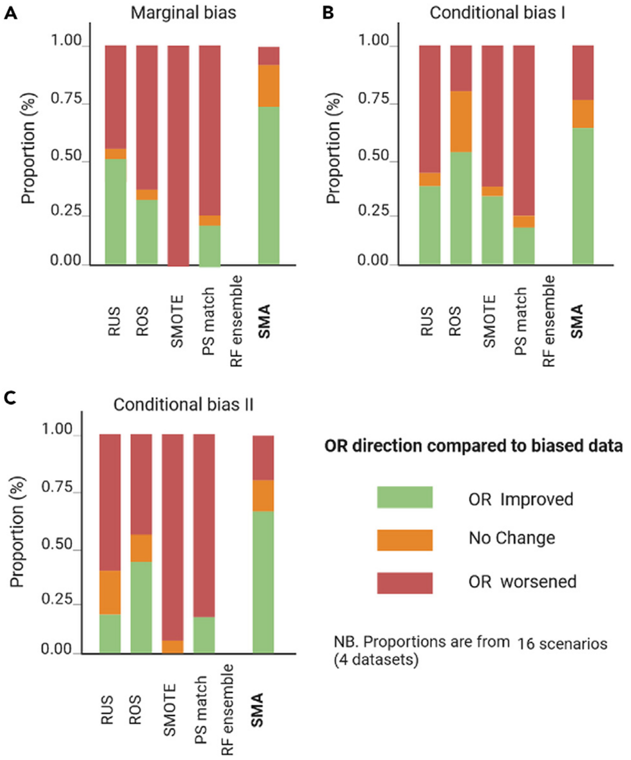

Figure 7. Summaries of the OR of the biasing covariate for the four real datasets
The relative performance of each bias-mitigating approach compared to the biased data estimate is shown. The OR direction is considered improved if the difference between the model OR and the ground-truth estimate is less than the difference between the biased data OR and the ground truth. Summaries are over all four datasets, and proportions range from 15% to 80%.

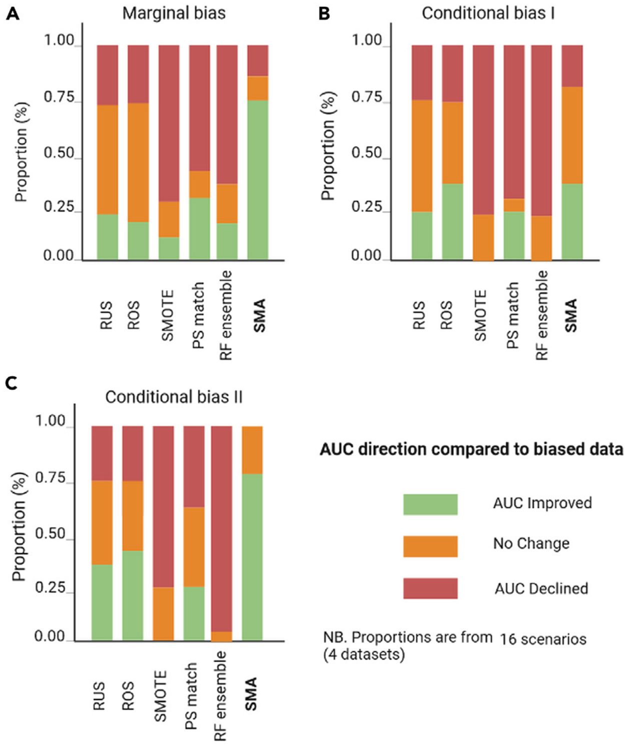

Figure 8. Summaries of the model AUC for the four real datasets
The relative performance of each bias-mitigating approach compared to biased data results is shown. The model AUC is considered improved if the difference between the model AUC and the ground-truth estimate is less than the difference between the biased data AUC and the ground truth. Summaries are over all four datasets, and proportions range from 15% to 80%.

performs well for high bias, there is no other single method that consistently performs better than SMA for the high bias case.

(3) For low to medium levels of bias, SMA had good performance for the prediction and calibration of the minority group.

The performances of the traditional approaches evaluated in this study are consistent with previous findings.[55–57] In the presence of marginal bias where observations are missing from the data solely based on their membership to a given predictor category, rebalancing approaches such as ROS, SMOTE, and RUS tend to perform reasonably well in low to moderate bias. However, as the severity of the bias increases, their effectiveness declines. In more complex conditional bias settings, where bias is induced by more than one covariate, these methods are not always reliable or reproducible. The SMA approach overcomes these obstacles by offering more accurate, stable, and comparable results to the ground truth.

While our study focused on biased covariates with binary values, this does not limit the method from being applied in cases where the biased covariate is multi-class with more than two categories. The generative model used for SMA can synthesize variables with multiple classes to add more individuals with the bias classes.

Overall, the synthetic augmentation approach described in this article can be a robust solution to mitigate data bias at the data-analysis stage. This is the case for low to medium levels of data bias and across multiple criteria. Although traditional methods such as ROS, SMOTE (or modifications), and PS matching remain useful, their effectiveness is greatly affected by how the bias is induced in the data and the amount of bias present. For high or extreme bias scenarios, there is no consistently high-performing bias-mitigation method across all evaluation metrics.

Limitations

The methods presented here require minimal knowledge of the nature of the bias by the analyst: only the variable and category affected, and the extent of bias on that variable. This would make the burden of using these bias-mitigation methods relatively small.

The effectiveness of bias-mitigation approaches may improve if the analyst can also identify the type of bias (whether marginal or conditional on one or more covariates). For example, while ROS was implemented solely based on oversampling observations from the minority group of the biased covariate, its performance in conditional bias settings may be better if the analyst could discern the additional covariate to condition upon. In practice, this is a difficult prerequisite to meet and, therefore, our study arguably makes the most practical assumptions.

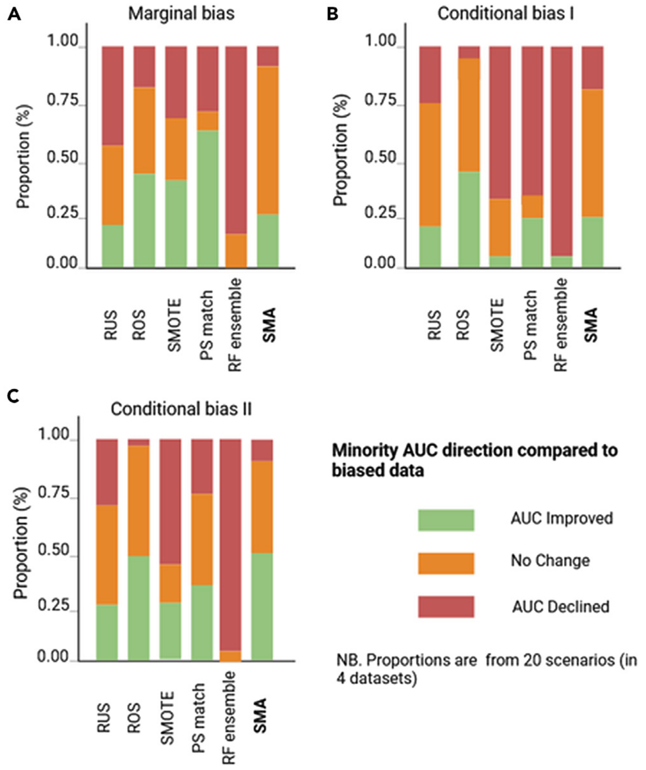

Figure 9. Summaries of the minority-group AUC over the four real datasets

The relative performance of each bias-mitigating approach compared to biased data results is shown. The minority AUC is considered improved if the difference between the model minority AUC and the ground-truth estimate is less than the difference between the biased data minority AUC and the ground truth. Summaries are over all bias proportions: 15%, 30%, 50%, 80%, and 95%.

The sample size of the biased data used in the SMA synthesis process is another potential limitation. In extreme bias settings or small sample settings, the data-synthesis process may not have enough information on the under-represented group to accurately capture the relationships and patterns in the data. This can result in synthetic data that poorly represent the ground truth and may not effectively mitigate bias. For example, in the breast cancer data, which had a relatively small sample size compared to the other datasets, the proposed SMA approach performed least effectively. In such small samples, even a moderate amount of bias could profoundly impact the effectiveness of the proposed bias-mitigating approaches.

Another question is whether the application of bias-mitigation methods, such as SMA, will create disincentives to recruit under-represented groups into health studies. However, there will be many situations where historical or existing data are being used and, therefore, there is no practical opportunity to recruit additional participants. In those cases, methods such as SMA can help address biases that may be introduced in models using existing data. In situations where new data are being collected, methods such as SMA can be used to perform a sensitivity analysis to inform additional recruitment decisions (by comparing the results from the original biased dataset with the results from the bias-mitigated dataset).

Variations of SMOTE and sampling techniques, including SMOTEBoost,[58] RUSBoost,[59] BalancedBoost,[60] and OverBagging,[61] have been proposed in the literature. The relative performance of these alternative methods may differ from the default approaches presented in this study. A comprehensive comparison of these variations to SMA represents a promising avenue for future investigation.

EXPERIMENTAL PROCEDURES

Resource availability

Lead contact

Further information and requests for resources should be directed to and will be fulfilled by the lead contact, Khaled El Emam (kelemam@ehealthinformation.ca).

Materials availability

This study did not generate new materials.

Data and code availability

The N0147 dataset can be requested from Project Data Sphere (https://www.projectdatasphere.org/). The CCHS dataset can be requested from Statistics Canada (https://www.statcan.gc.ca/en/microdata/data-centres). The Danish Colorectal Cancer Group (DCCG) dataset can be requested from the Danish Colon Cancer Group (https://www.dmcg.dk/). The breast cancer dataset is publicly available from the UC Irvine machine-learning repository and also available in the code and data deposit for this paper. The R code and public datasets used for this study are available for download from https://ehealthinformation.ca/blog/EHIL%20Blog/Bias-mitigation and on OSF (https://doi.org/10.17605/OSF.IO/RKF9T).[62]

Ethics and consent to participate

This study was approved by the CHEO Research Institute Research Ethics Board protocol CHEOREB #23/34X. All research was performed in accordance with relevant guidelines/regulations. Patient consent was not required by the Institutional Review Board.

Proposed approach: Synthetic minority augmentation

SMA process

We propose a bias-mitigating approach that is based on synthetic data generation. By augmenting the minority group in biased datasets with synthetic records, we reconstruct full data samples that are reflective of the baseline distribution. The approach is termed SMA. A schematic of the SMA approach is provided in Figure 11.

Starting with a biased sample, SMA can be implemented in three stages.

(1) Synthesis stage: construct a synthetic version of the biased data using sequential synthesis based on gradient-boosted decision trees.
(2) Sampling stage: sample observations from the minority group of the generated synthetic dataset.
(3) Augmentation stage: add the sampled observations from step 2 to the biased data to create a complete, bias-mitigated dataset.

Step 1: Synthesis stage. The type of generative model used was a sequential tree-based synthesizer.[63] This type of generative model has been used to synthesize health and social sciences data,[64–72] and has been applied in research studies on synthetic data.[64,73,74]

To account for the stochastic nature of the generative model, we synthesize m copies of the biased data, which are later combined in the downstream analysis. A summary of the algorithm is presented below.

Step 2: Sampling stage. The next step is to draw synthetic records of the minority group from the data synthesized. For each of the m synthetic datasets generated, we sample k records from the minority group so that its distribution is the same as the baseline distribution. For example, in a biased dataset with under-represented female participants, we can construct synthetic samples of only the minority group by sampling synthetic copies of female observations. It is this minority-group sample that is used in stage 3.

Step 3: Augmentation stage. In the final stage, we augment the starting biased sample with the sampled synthetic observations of the minority group. We thus end up with a larger dataset involving additional observations from the

CellPress
OPEN ACCESS

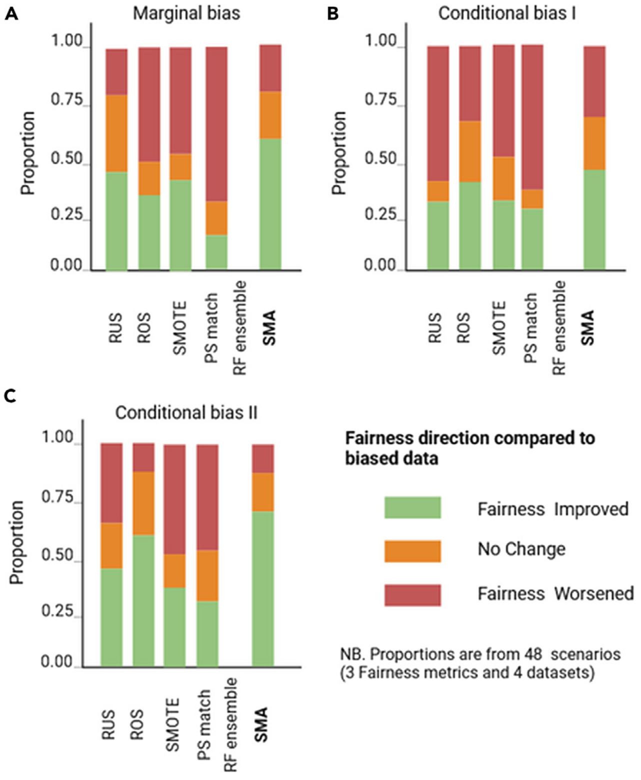

Figure 10. Summaries of the fairness metrics SPD, EOD, and AOD over the four real datasets

The relative performance of each bias-mitigating approach compared to biased data results is shown. Fairness is considered improved if the difference between the model fairness and the ground-truth estimate is less than the difference between the biased data fairness and the ground truth. Summaries are over all four datasets, and proportions range from 15% to 80%.

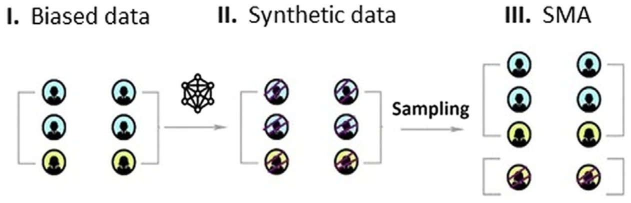

Figure 11. A schematic for synthetic data augmentation from biased data

From (I) to (II), a synthetic version of the biased data is generated. In step (III), the synthetic minority group is sampled from (II) and augmented with the biased data in (I) to generate a rebalanced dataset.

under-represented category, ensuring a distribution consistent with the baseline distribution. As the released data are composed of a mixture of both original biased data and synthetic data, this technique can be viewed as a form of partial data synthesis.

For each of the *m* sampled synthetic minority groups, we create a new, rebalanced dataset for analysis. Each dataset is independently analyzed, and the results are subsequently combined according to a clearly defined combining rule. For example, regression effect estimates can be reported by taking the average across the *m* samples, or average AUC estimates could be reported for predictive performance.

SMA synthetic data-generation algorithm

For data synthesis, we apply decision trees sequentially. Each model in the sequence was trained using a gradient-boosted decision tree,[75,76] with Bayesian optimization and 5-fold cross-validation for hyperparameter selection.[77] The variable sequence is optimized using a particle swarm algorithm.[63]

The process is illustrated in Figure 12 for a four-variable dataset, V1–V4. In the fitting phase, three models are constructed, M1–M3. As shown, the first model takes as input V1 and produces V2 as the outcome. The nature of the variables, whether categorical or continuous, does not affect the process, as the model adjusts to become either a classification tree or a regression tree accordingly. The second model in the sequence takes V1 and V2 as input with V3 as the outcome, and so on.

The synthesis step is initiated by sampling from the actual or fitted distribution of the first variable, V1. This creates the synthetic version of that variable, sV1. Sampled values are then entered into the first model to generate the distribution of sV2 in the predicted terminal node of the tree. The synthetic value of sV2 is either sampled according to the predicted probabilities (for categorical variables) or smoothed using a kernel density estimator with boundary correction (for continuous variables),[78] with bandwidth computed from the original data.

Having generated two synthetic values, sV1 and sV2, these form the input for model M2 to produce the distribution of sV3. Again, the generated synthetic value is either sampled from that predicted distribution or smoothed. The process proceeds in this manner until all variables are synthesized.

There are advantages to using gradient-boosted decision trees. Previous studies have shown that discriminative and regression models on heterogeneous datasets (i.e., those with continuous and categorical values, and with missingness) using boosted trees perform better than artificial neural network architectures on tabular datasets.[79–83] Sequential synthesis implements multiple boosted decision trees in a specific order, and existing evaluations suggest that this is a relatively competitive type of generative model relative to artificial neural networks for tabular data.[84]

Synthesis pre- and post-processing

Pre- and post-processing are important to ensure that a generative model can handle realistic datasets. The following summarizes the steps in the SMA implementation.

For the synthesis of categorical variables, synthetic values are generated based on predicted probabilities. In general, boosted trees do not output correct probabilities and these need to be calibrated, especially as the number of iterations increases.[85] In addition, for imbalanced categorical outcomes, rebalancing of the classes gives incorrect probabilities. Therefore, the predicted probabilities are adjusted using beta calibration.[86]

For each continuous variable V_i being synthesized, we first convert them to a Gaussian distribution before synthesis. The empirical cdf was applied to each variable $F_i(V_i)$, then the quantile function for the standard normal was applied, $\Phi^{-1}(F_i(V_i))$, which is used for synthesis. After synthesis, the generated values sV_i are converted back as $F_i^{-1}(\Phi(sV_i))$. This approach improves the modeling of continuous variables with long tails and multi-modal variables.

The variables used have a shadow variable added with a missingness indicator. This allows for the modeling of missingness in the output data.

Evaluation protocol

The analytic workload that we assume in our evaluations is logistic regression (LR). LR models are common in health research for diagnostic and prognostic modeling.[87] A recent systematic review has shown that LR performance is comparable to the use of machine-learning models for clinical prediction workloads.[88] Therefore, it represents a realistic workload to assess covariate bias.

To evaluate the methods for mitigating data bias, we follow the process summarized in Figure 13. Starting with the original cohort which we assume represents the baseline distribution, we randomly split the dataset into training and holdout partitions using 70:30 proportions. A baseline "ground truth" LR is performed on the original training partition, and predictions are evaluated on the holdout partition.

We then induce different types of data bias into the training partition to obtain biased training partitions. An LR model of the same form is then fitted to the biased training data, and its prediction performance is assessed using the holdout partition. Subsequently, we apply different mitigation techniques to the training data, train the LR models on the mitigated datasets, and evaluate their prediction performance on the same holdout dataset.

It is important to note that the same holdout partition is employed for all analyses to ensure consistent results.

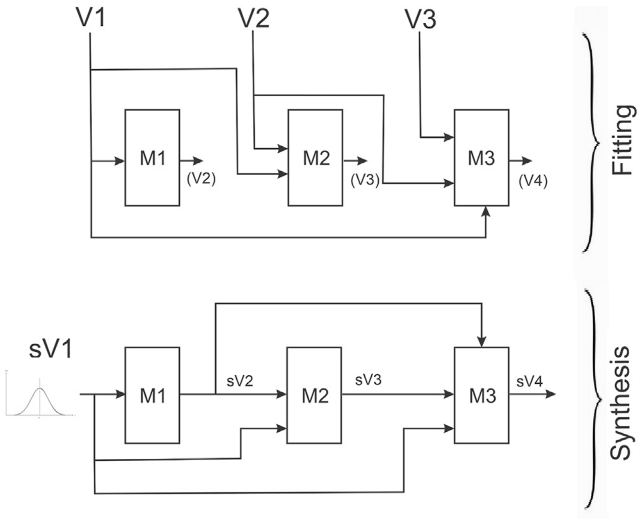

Figure 12. Illustration of the synthesis process for a four-variable dataset

Two types of validation were carried out. The "full model" validation computed the performance metrics on all the training/test datasets. The disaggregated or "stratified model" validation computed the prediction performance separately for the minority and the majority groups of the biased covariate on the holdout dataset. The latter validation determines how model performance varies by group.

The performance of bias-mitigation methods was compared to the ground truth. The bias-mitigation methods evaluated were RUS, ROS, SMOTE, PS matching, RF ensembles, and synthetic data augmentation. The selected approaches represent each of the main classifications of the bias-mitigating techniques in the literature and are readily accessible through standard statistical programming packages. They are described further in Appendix A in supplemental experimental procedures.

For all bias-mitigation methods, the only information that is assumed known by the analyst is the extent of bias and the biased covariate category. For example, the analyst might know that the biased covariate is "sex," the under-represented group is "female," and the extent of under-representation relative to the baseline distribution is 15%. In practice, it is typical for analysts to have access to just this level of detail and not much else about the nature of the biased covariate, such as any conditioning information.

Inducing data bias

To induce sample selection bias, we mimic the two scenarios of biased data presented in Winship and Mare[15] and Heitjan and Basu.[89] The first bias scenario occurs when we explicitly leave out observations from a specific category of the biased covariate, independent of any other covariate in the data. For example, Goldberger[90] explored the problem of estimating the effect of education (biased covariate) on income (outcome) where only individuals with incomes below $15,000 were deemed to have met the inclusion criteria. The second bias scenario involves selecting observations based on a combination of the biased covariate and other measured covariates. For example, by conditioning on gender, the same study excluded female participants whose incomes were below $15,000. More specifically, we classify these biases as follows.

(1) Marginal bias: this represents the simplest form of sampling bias where the biased covariate categories are randomly removed, irrespective of the values of other covariates in the model.
(2) Conditional bias: here, observations are removed from one or more biased covariate categories based on other covariates in the dataset. If the biased covariate is Z, then observations in one category of Z, say $Z = 1$, are excluded provided another covariate, X, is greater than the threshold x^*. We consider two types:
(3) Conditional bias I: when the odds of X in Z range from weak to moderate (equivalently, the biased covariate X is not strongly correlated with the biased covariate Z); and
(4) Conditional bias II: when the odds of X in Z are high, e.g., observations are removed from the predictor category $Z = 1$ if they are strongly correlated with the covariate X.

The strength of the associations between the biased covariate Z and the conditioned covariate X is determined by conducting a univariable LR between Z and X. While the sampling biases presented in (1) and (2) can be viewed as forms of missing-at-random (MAR) conditions,[91] the main difference between our setting and the traditional MAR setup is that we are primarily focused on missing observations instead of missing covariate values in some observations. In each (marginal or conditional bias) case, the sampling proportion could be varied to create training datasets of different imbalance ratios. Additionally, both positive and negative associations were considered under conditional bias, regardless of the strength of the association between Z and X.

For uniformity, the imbalance ratio of the biased covariate group is taken to determine the severity of the dataset bias. Thus, the bias percentage is only determined by the proportion of observations removed from the biased covariate, even under conditional bias.

In our analysis, the amount of bias induced on the biased covariate was varied by changing the proportion of observations removed in the biased covariate group from low (proportion = 15% or 30%), moderate (proportion = 50%), high (proportion = 80%), and extreme (proportion = 95%).

Evaluation metrics for model performance and fairness

The metrics evaluated for comparing the performances of the LR models are: (1) the overall model AUCs computed on the holdout partition; (2) the estimate of the biased covariate coefficient and the associated standard error; (3) the 95% confidence interval overlap of the biased covariate coefficient of the original cohort (ground truth) with each bias-mitigation approach[92]; (4) AUCs and Brier scores for predicting only the minority group of the biased covariate (this is the stratified model); and (5) model fairness.

Note that the biased dataset (with no bias-mitigation approaches applied) will have fewer observations than all of the other training datasets. This means that the standard errors will be higher by definition, and the confidence intervals will be wider. Since this is partially an artifact of the sample size, we do not present these values for that particular dataset.

Additionally, several commonly used fairness metrics were evaluated to assess the differences in classifications between the categories of the biased covariate. Specifically, we estimate the (1) SPD,[93] (2) EOD,[94] and (3) AOD.[95]

SPD compares the probability of the outcome given the one category of the biased covariate to the probability of the outcome given the other predictor category. SPD can be expressed as

$$\text{SPD} = Pr[\hat{Y} = 1|Z = 1, X = x] - Pr[\hat{Y} = 1|Z = 0, X = x], \quad \text{(Equation 1)}$$

where $\hat{Y}$ is the predicted value of the outcome, Z is the binary predictor of interest, and X represents the remaining covariates. An SPD estimate of zero indicates that the classification algorithm provides equal probabilities of the outcomes for both groups of Z. Thus, the closer the estimate is to zero, the fairer the approach.

EOD compares the true-positive rates (TPRs) between the categories of the biased covariate. The metric aims to ensure that individuals from different groups have equal chances of being correctly classified for a positive outcome when they should indeed receive it. EOD is given by the expression

$$\text{EOD} = Pr[\hat{Y}| Y = 1, Z = 1, X = x] - Pr[\hat{Y}|Y = 1, Z = 0, X = x], \quad \text{(Equation 2)}$$

where an EOD of zero indicates that the classification algorithm provides equal opportunity for both groups, as they have the same TPRs ($\text{TPR} = Pr[\hat{Y} = 1|Y = 1, Z]$) for $Z = 1$ and $Z = 0$.

AOD estimates the average differences between the false-positive rate and the TPR of the biased covariate categories. AOD can be expressed as

$$\text{AOD} = |(\text{FPR}_{MN} - \text{FPR}_{MJ}) + (\text{TPR}_{MN} - \text{TPR}_{MJ})| / 2, \quad \text{(Equation 3)}$$

CellPress
OPEN ACCESS

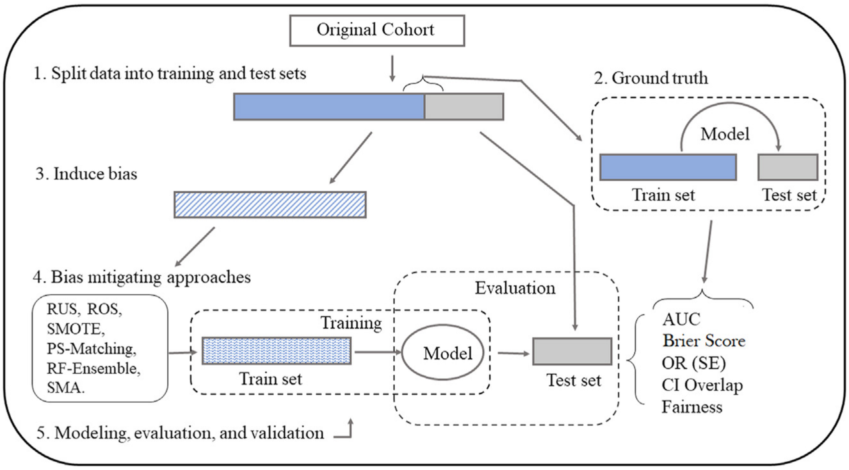

Figure 13. Schematic for training and evaluating bias-mitigating approaches

where the false-positive rates of the minor (MN) category $FPR_{MN} = Pr[\widehat{Y} = 1|Y = 0, Z = 1]$ and that of the major (MJ) category $FPR_{MJ} = Pr[\widehat{Y} = 1|Y = 0, Z = 0]$. The TPRs of the minority group $TPR_{MN} = Pr[\widehat{Y} = 1|Y = 1, Z = 1]$ and that of the major $TPR_{MJ} = Pr[\widehat{Y} = 1|Y = 1, Z = 0]$).

Despite the optimal score for the three fairness metrics being zero, our analysis utilizes the ground-truth estimates of these metrics as the benchmark. For SPD and EOD, deviations from zero, whether positive or negative, indicate the degree of dissimilarity between the groups.

Datasets

Simulated data

First, we consider a simulation setting involving a binary outcome Y, binary predictor variable Z sampled from the binomial(p = 0.5), two binary variables X_1 and X_2 with the observations of X_2 given $Z = 1$ sampled from the binomial(p = 0.4), while the remaining observations of X_2 given $Z = 0$ were sampled from binomial(p = 0.35). All of the observations of X_1 were sampled from binomial(p = 0.6), independent of Z. We also considered a continuous predictor of the outcome U generated from the log-normal(12,3.5) and independent of the other covariates. An effect modifier term for the interaction between Z and X_2 was also included. We define the LR model as:

$$P(Y = 1) = \text{expit}(\alpha + \beta_Z Z + \beta_{X1} X_1 + \beta_{X2} X_2 + \beta_{ZX2} Z X_2 + \beta_U U), \quad \text{(Equation 4)}$$

where $\text{expit}(t) = \exp(t)/(1 + \exp(t))$. For the set of fixed parameters ($\alpha = \log(0.5)$, $\beta_Z = \log(1.25)$, $\beta_{X1} = \log(0.3)$, $\beta_{X2} = \log(2)$, $\beta_{ZX2} = -0.47$, $\beta_U = \log(0.5)$), we construct the full cohort data of 5,000 observations before inducing bias in the sampled training dataset. The simulation is repeated to generate 500 datasets. For all simulated samples, the full cohort (with equal proportions of $Z = 1$ and $Z = 0$) was considered the ground truth. A summary of the distributions of the baseline variables, stratified by predictor Z, is provided in Appendix B in supplemental experimental procedures.

To induce marginal bias in the training dataset, observations were randomly sampled and removed from the minority-group predictor category $Z = 0$, independent of the observed values of the other predictors in the dataset. For the conditional bias I case, observations were randomly removed from the minority-group predictor category $Z = 0$ if they had the covariate value $X_1 = 0$. That is, we conditioned the exclusion of observations in the training cohort based on a covariate X_1 that is weakly associated with the biased covariate Z ($\beta = 0.068$, p value = 0.325). For the second conditional case (conditional bias II), we condition inclusion based on a covariate X_2 that is strongly associated with the predictor Z ($\beta = 0.187$, p value = 0.009). Thus, the observations excluded from the training cohort had predictor values $Z = 0$ and $X_2 = 0$. All of the primary analyses were based on these settings.

In addition to the fixed parameter (i.e., $\beta_Z = \log(1.25)$) used for the biased covariate in the primary analysis, the value of β_Z was varied to assess the robustness of the simulation results. In particular, the effect size of the biased covariate β_Z is also varied from small to large, where the definitions of small to large effect sizes are based on previous recommendations.[96,97] A null effect value was also evaluated (i.e., $\beta_Z = \log(1.01)$) to allow us to simulate the case when a biased covariate has no impact on the outcome (and hence bias-mitigation approaches would be expected to have no impact).

For each simulated dataset, we follow the evaluation protocol in "proposed approach: synthetic minority augmentation" to estimate the AUC, the effect estimates of the biased covariate, the standard deviation over the simulations, and the corresponding confidence interval overlap of each simulated data with the ground truth estimate. We also estimate the statistical parity, the EOD, and the AOD, as defined in "inducing data bias."

Real datasets

We compared the approaches on four biomedical datasets: the cardiovascular health data from the CCHS of 2014,[98] the N0147 colon cancer trial data from the Project Data Sphere initiative,[99] the Danish colon cancer registry dataset from the DCCG database,[100] and the breast cancer data from the UCI machine-learning repository.[101] In Table 1, we present a summary of these datasets. The results for the CCHS dataset are presented in the main text while the results for the remaining three datasets are shown in Appendix D in supplemental experimental procedures. Overall summaries across all datasets are also presented.

The datasets considered had varying proportions of imbalance in the biased covariate and different sample sizes to reflect the diversity of datasets in real-world settings. Our analysis thus assumes each dataset as the ground truth before evaluating the impact of additional covariate bias. As in the simulated data setting, marginal bias was induced in each training set by removing observations from the minority group of the biased covariate (Table 1, column 4), independent of any other covariate. To induce the first conditional bias (conditional bias I), we condition the removal of observations from the minority-group predictor category based on a predictor that is weakly or moderately associated with the biased covariate (CBI in column 5 of Table 1). For the second conditional case (conditional bias II), we condition exclusion on the predictor labeled CBII (column 5), which is strongly associated with the biased covariate. A detailed description of the model-fitting procedure utilized for each dataset is provided in Appendix C in supplemental experimental procedures.

Inducing data bias

The proportions of observations excluded under each bias setting were set at 15%, 30%, 50%, and 80%, respectively. For datasets with small samples, removing 95% of observations in the biasing group is not always realistic, as the remaining samples might be too small to perform parameter estimation. Thus, for uniformity, the 95% missing proportion was excluded in the real dataset examples. We follow the evaluation protocol in "proposed approach: synthetic minority augmentation" to compare the performance of the approaches on the datasets.

For the CCHS dataset, a marginal bias case was induced by randomly sampling and removing observations from the training set if they were female, irrespective of their other covariates. The two conditional bias settings were induced by conditioning on covariates that were weakly and strongly associated with gender, respectively. In the first conditional case, female participants were only excluded from the training set if they were not new immigrants (new immigration is weakly associated with gender, $\beta = -0.059$, p value = 0.064). In the second case, observations were removed from the training set if they were female and belonged to a specific marital category (e.g., marital status = yes). Marital status was selected as it is strongly associated with gender ($\beta = 0.158$,

p value <0.001). A detailed description of the variables conditioned on in each of the remaining datasets is presented in Appendix C in supplemental experimental procedures.

SUPPLEMENTAL INFORMATION

Supplemental information can be found online at https://doi.org/10.1016/j.patter.2024.100946.

ACKNOWLEDGMENTS

This research was enabled in part by computational support provided by Compute Ontario (computeontario.ca) and Compute Canada (www.computecanada.ca). This work was partially funded by the Canada Research Chairs program through the Canadian Institutes of Health Research, a Discovery Grant RGPIN-2022-04811 from the Natural Sciences and Engineering Research Council of Canada. LJ was funded by the Precision Child and Youth Mental Health initiative at the CHEO Research Institute.

AUTHOR CONTRIBUTIONS

L.J. and K.E.E. designed the study, obtained the data, performed the analysis, and contributed to writing the paper. A.E.-H. contributed to obtaining the data and writing the paper.

DECLARATION OF INTERESTS

This work was performed in collaboration with Replica Analytics Ltd. This company is a spin-off from the Children's Hospital of Eastern Ontario Research Institute. K.E.E. is co-founder and has equity in this company.

Received: August 21, 2023
Revised: October 23, 2023
Accepted: February 8, 2024
Published: February 29, 2024

REFERENCES

1. Yadav, P., Steinbach, M., Kumar, V., and Simon, G. (2018). Mining Electronic Health Records (EHRs): A Survey. ACM Comput. Surv. *50*, 1–40. https://doi.org/10.1145/3127881.
2. Detsky, A.S. (2006). Sources of bias for authors of clinical practice guidelines. CMAJ (Can. Med. Assoc. J.) *175*, 1033–1035. https://doi.org/10.1503/cmaj.061181.
3. Glauner, P., Valtchev, P., and State, R. (2018). Impact of biases in big data. Preprint at arXiv. https://doi.org/10.48550/arXiv.1803.00897.
4. Cirillo, D., Catuara-Solarz, S., Morey, C., Guney, E., Subirats, L., Mellino, S., Gigante, A., Valencia, A., Rementeria, M.J., Chadha, A.S., and Mavridis, N. (2020). Sex and gender differences and biases in artificial intelligence for biomedicine and healthcare. Npj Digit. Med. *3*, 81. https://doi.org/10.1038/s41746-020-0288-5.
5. Pandis, N. (2014). Bias in observational studies. Am. J. Orthod. Dentofacial Orthop. *145*, 542–543. https://doi.org/10.1016/j.ajodo.2014.01.008.
6. Huang, J., Gretton, A., Borgwardt, K.M., Schölkopf, B., and Smola, A.J. (2006). Correcting Sample Selection Bias by Unlabeled Data. NeurIPS (Advances in Neural Information Processing Systems *19*, 601–608. https://papers.nips.cc/paper_files/paper/2006.
7. Panzeri, S., Magri, C., and Carraro, L. (2008). Sampling bias. Scholarpedia *3*, 4258. https://doi.org/10.4249/scholarpedia.4258.
8. Signorini, D.F., Leung, O., Simes, R.J., Beller, E., Gebski, V.J., and Callaghan, T. (1993). Dynamic balanced randomization for clinical trials. Stat. Med. *12*, 2343–2350. https://doi.org/10.1002/sim.4780122410.
9. Lim, C.-Y., and In, J. (2019). Randomization in clinical studies. Korean J. Anesthesiol. *72*, 221–232. https://doi.org/10.4097/kja.19049.
10. Hripcsak, G., Knirsch, C., Zhou, L., Wilcox, A., and Melton, G. (2011). Bias Associated with Mining Electronic Health Records. J. Biomed. Discov. Collab. *6*, 48–52. https://doi.org/10.5210/disco.v6i0.3581.
11. Kaplan, R.M., Chambers, D.A., and Glasgow, R.E. (2014). Big Data and Large Sample Size: A Cautionary Note on the Potential for Bias. Clin. Transl. Sci. *7*, 342–346. https://doi.org/10.1111/cts.12178.
12. Agniel, D., Kohane, I.S., and Weber, G.M. (2018). Biases in electronic health record data due to processes within the healthcare system: retrospective observational study. BMJ *361*, k1479. https://doi.org/10.1136/bmj.k1479.
13. Berk, R.A. (1983). An Introduction to Sample Selection Bias in Sociological Data. Am. Socio. Rev. *48*, 386–398. https://doi.org/10.2307/2095230.
14. Tripepi, G., Jager, K.J., Dekker, F.W., and Zoccali, C. (2010). Selection Bias and Information Bias in Clinical Research. Nephron Clin. Pract. *115*, c94–c99. https://doi.org/10.1159/000312871.
15. Winship, C., and Mare, R.D. (1992). Models for Sample Selection Bias. Annu. Rev. Sociol. *18*, 327–350. https://doi.org/10.1146/annurev.so.18.080192.001551.
16. Gianfrancesco, M.A., Tamang, S., Yazdany, J., and Schmajuk, G. (2018). Potential Biases in Machine Learning Algorithms Using Electronic Health Record Data. JAMA Intern. Med. *178*, 1544–1547. https://doi.org/10.1001/jamainternmed.2018.3763.
17. Lohr, S. (2022). Facial Recognition Is Accurate, If You're a White Guy. In Ethics of Data and Analytics (Auerbach Publications)), pp. 143–147. https://doi.org/10.1201/9781003278290-22.
18. Jacoba, C.M.P., Celi, L.A., Lorch, A.C., Fickweiler, W., Sobrin, L., Gichoya, J.W., Aiello, L.P., and Silva, P.S. (2023). Bias and Non-Diversity of Big Data in Artificial Intelligence: Focus on Retinal Diseases. Semin. Ophthalmol. *38*, 433–441. https://doi.org/10.1080/08820538.2023.2168486.
19. Tang, L., and Liu, H. (2005). Bias analysis in text classification for highly skewed data. In Fifth IEEE International Conference on Data Mining (ICDM'05), p. 4. https://doi.org/10.1109/ICDM.2005.34.
20. Meyer, J., Rauchenstein, L., Eisenberg, J.D., and Howell, N. (2020). Artie Bias Corpus: An Open Dataset for Detecting Demographic Bias in Speech Applications. In Proceedings of the 12th Language Resources and Evaluation Conference (European Language Resources Association)), pp. 6462–6468. https://aclanthology.org/2020.lrec-1.796/.
21. Feng, S., Kudina, O., Halpern, B.M., and Scharenborg, O. (2021). Quantifying Bias in Automatic Speech Recognition. Preprint at arXiv. https://doi.org/10.48550/arXiv.2103.15122.
22. Rothstein, M.A. (2015). Ethical Issues in Big Data Health Research. J. Law Med. Ethics *43*, 425–429. https://doi.org/10.1111/jlme.12258.
23. Niethammer, C. (2020). AI Bias Could Put Women's Lives at Risk - A Challenge for Regulators (Forbes). https://www.forbes.com/sites/carmenniethammer/2020/03/02/ai-bias-could-put-womens-lives-at-riska-challenge-for-regulators/.
24. Obermeyer, Z., Powers, B., Vogeli, C., and Mullainathan, S. (2019). Dissecting racial bias in an algorithm used to manage the health of populations. Science *366*, 447–453. https://doi.org/10.1126/science.aax2342.
25. Boratto, L., Fenu, G., and Marras, M. (2019). The Effect of Algorithmic Bias on Recommender Systems for Massive Open Online Courses. In Advances in Information Retrieval Lecture Notes in Computer Science, L. Azzopardi, B. Stein, N. Fuhr, P. Mayr, C. Hauff, and D. Hiemstra, eds. (Springer International Publishing), pp. 457–472. https://doi.org/10.1007/978-3-030-15712-8_30.
26. Baeza-Yates, R. (2020). Bias in search and recommender systems. In Fourteenth ACM Conference on Recommender Systems (Association for Computing Machinery), p. 2. https://doi.org/10.1145/3383313.3418435.
27. Danielle, K., Guo, P., and Kessler, S. (2017). Algorithms in the Criminal Justice System: Assessing the Use of Risk Assessments in Sentencing.

Responsive Communities Initiat. Berkman Klein Cent. Internet Soc. Harv. Law Sch. https://dash.harvard.edu/handle/1/33746041.

28. Panch, T., Mattie, H., and Atun, R. (2019). Artificial intelligence and algorithmic bias: implications for health systems. J. Glob. Health *9*, 010318. https://doi.org/10.7189/jogh.09.020318.
29. Hutcheon, J.A., Chiolero, A., and Hanley, J.A. (2010). Random measurement error and regression dilution bias. The BMJ *340*, c2289. https://doi.org/10.1136/bmj.c2289.
30. Egbewale, B.E., Lewis, M., and Sim, J. (2014). Bias, precision and statistical power of analysis of covariance in the analysis of randomized trials with baseline imbalance: a simulation study. BMC Med. Res. Methodol. *14*, 49. https://doi.org/10.1186/1471-2288-14-49.
31. Long, J.P., and Ha, M.J. (2022). Sample selection bias in evaluation of prediction performance of causal models. Stat. Anal. Data Min. *15*, 5–14. https://doi.org/10.1002/sam.11559.
32. Liu, A., and Ziebart, B. (2014). Robust Classification Under Sample Selection Bias. NeurIPS (Advances in Neural Information Processing Systems *27*, 37–45. https://proceedings.neurips.cc/paper_files/paper/2014/hash/d67d8ab4f4c10bf22aa353e27879133c-Abstract.html.
33. Negassa, A., and Hanley, J.A. (2007). The effect of omitted covariates on confidence interval and study power in binary outcome analysis: A simulation study. Contemp. Clin. Trials *28*, 242–248. https://doi.org/10.1016/j.cct.2006.08.007.
34. Skelly, A.C., Dettori, J.R., and Brodt, E.D. (2012). Assessing bias: the importance of considering confounding. Evid. Base Spine Care J. *3*, 9–12. https://doi.org/10.1055/s-0031-1298595.
35. Wahl, S., Boulesteix, A.-L., Zierer, A., Thorand, B., and van de Wiel, M.A. (2016). Assessment of predictive performance in incomplete data by combining internal validation and multiple imputation. BMC Med. Res. Methodol. *16*, 144. https://doi.org/10.1186/s12874-016-0239-7.
36. Mehrabi, N., Morstatter, F., Saxena, N., Lerman, K., and Galstyan, A. (2021). A Survey on Bias and Fairness in Machine Learning. ACM Comput. Surv. *54*, 1–35. https://doi.org/10.1145/3457607.
37. Marcelin, J.R., Siraj, D.S., Victor, R., Kotadia, S., and Maldonado, Y.A. (2019). The Impact of Unconscious Bias in Healthcare: How to Recognize and Mitigate It. J. Infect. Dis. *220*, S62–S73. https://doi.org/10.1093/infdis/jiz214.
38. Rouzrokh, P., Khosravi, B., Faghani, S., Moassefi, M., Vera Garcia, D.V., Singh, Y., Zhang, K., Conte, G.M., and Erickson, B.J. (2022). Mitigating Bias in Radiology Machine Learning: 1. Data Handling. Radiol. Artif. Intell. *4*, e210290. https://doi.org/10.1148/ryai.210290.
39. Dixon, L., Li, J., Sorensen, J., Thain, N., and Vasserman, L. (2018). Measuring and Mitigating Unintended Bias in Text Classification. In Proceedings of the 2018 AAAI/ACM Conference on AI, Ethics, and Society (ACM), pp. 67–73. https://doi.org/10.1145/3278721.3278729.
40. Griffin, G.P., Mulhall, M., Simek, C., and Riggs, W.W. (2020). Mitigating Bias in Big Data for Transportation. J. Big Data Anal. Transp. *2*, 49–59. https://doi.org/10.1007/s42421-020-00013-0.
41. Hao, X., Liu, L., Yang, R., Yin, L., Zhang, L., and Li, X. (2023). A Review of Data Augmentation Methods of Remote Sensing Image Target Recognition. Remote Sens *15*, 827. https://doi.org/10.3390/rs15030827.
42. Park, D.S., Chan, W., Zhang, Y., Chiu, C.-C., Zoph, B., Cubuk, E.D., and Le, Q.V. (2019). SpecAugment: A Simple Data Augmentation Method for Automatic Speech Recognition. Interspeech, 2613–2617. https://doi.org/10.21437/Interspeech.2019-2680.
43. Hao, X., Zhu, Y., Appalaraju, S., Zhang, A., Zhang, W., Li, B., and Li, M. (2023). MixGen: A New Multi-Modal Data Augmentation. Proceedings of the IEEE/CVF, 379–389. https://doi.org/10.1109/WACVW58289.2023.00042.
44. Jain, N., Olmo, A., Sengupta, S., Manikonda, L., and Kambhampati, S. (2022). Imperfect ImaGANation: Implications of GANs exacerbating biases on facial data augmentation and snapchat face lenses. Artif. Intell. *304*, 103652. https://doi.org/10.1016/j.artint.2021.103652.
45. Chawla, N.V. (2010). Data mining for imbalanced datasets: An overview. In Data mining and knowledge discovery handbook (Springer), pp. 875–886. https://doi.org/10.1007/978-0-387-09823–09824.
46. Kotsiantis, S., Kanellopoulos, D., and Pintelas, P. (2006). Handling imbalanced datasets: A review. GESTS Int. Trans. Comput. Sci. Eng. *30*, 25–36. https://doi.org/10.1007/978-0-387-09823-4_45.
47. Wongvorachan, T., He, S., and Bulut, O. (2023). A Comparison of Undersampling, Oversampling, and SMOTE Methods for Dealing with Imbalanced Classification in Educational Data Mining. Information *14*, 54. https://doi.org/10.3390/info14010054.
48. Chawla, N.V., Bowyer, K.W., Hall, L.O., and Kegelmeyer, W.P. (2002). SMOTE: Synthetic Minority Over-sampling Technique. J. Artif. Intell. Res. *16*, 321–357. https://doi.org/10.1613/jair.953.
49. Galar, M., Fernandez, A., Barrenechea, E., Bustince, H., and Herrera, F. (2012). A Review on Ensembles for the Class Imbalance Problem: Bagging-Boosting-and Hybrid-Based Approaches. IEEE Trans. Syst. Man Cybern. C *42*, 463–484. https://doi.org/10.1109/TSMCC.2011.2161285.
50. Lomax, S., and Vadera, S. (2013). A survey of cost-sensitive decision tree induction algorithms. ACM Comput. Surv. *45*, 1–35. https://doi.org/10.1145/2431211.2431215.
51. Khan, S.S., and Madden, M.G. (2010). A Survey of Recent Trends in One Class Classification. In Artificial Intelligence and Cognitive Science Lecture Notes in Computer Science, L. Coyle and J. Freyne, eds. (Springer), pp. 188–197. https://doi.org/10.1007/978-3-642-17080-5_21.
52. Dong, X., Yu, Z., Cao, W., Shi, Y., and Ma, Q. (2020). A survey on ensemble learning. Front. Comput. Sci. *14*, 241–258. https://doi.org/10.1007/s11704-019-8208-z.
53. Sun, L., and Bull, S.B. (2005). Reduction of selection bias in genomewide studies by resampling. Genet. Epidemiol. *28*, 352–367. https://doi.org/10.1002/gepi.20068.
54. Gray, L. (2016). The importance of post hoc approaches for overcoming non-response and attrition bias in population-sampled studies. Soc. Psychiatr. Psychiatr. Epidemiol. *51*, 155–157. https://doi.org/10.1007/s00127-015-1153-8.
55. D'Agostino, R.B., Jr. (1998). Propensity score methods for bias reduction in the comparison of a treatment to a non-randomized control group. Stat. Med. *17*, 2265–2281. https://doi.org/10.1002/(SICI)1097-0258(19981015)17:19<2265::AID-SIM918>3.0.CO;2-B.
56. Kim, J.-Y., and Cho, S.-B. (2022). An information theoretic approach to reducing algorithmic bias for machine learning. Neurocomputing *500*, 26–38. https://doi.org/10.1016/j.neucom.2021.09.081.
57. Chen, Z., Zhang, J.M., Sarro, F., and Harman, M. (2023). A Comprehensive Empirical Study of Bias Mitigation Methods for Machine Learning Classifiers. ACM Trans. Software Eng. Methodol. *32*, 1–30. https://doi.org/10.1145/3583561.
58. Chawla, N.V., Lazarevic, A., Hall, L.O., and Bowyer, K.W. (2003). SMOTEBoost: Improving Prediction of the Minority Class in Boosting. In Knowledge Discovery in Databases: PKDD 2003 Lecture Notes in Computer Science, N. Lavrač, D. Gamberger, L. Todorovski, and H. Blockeel, eds. (Springer), pp. 107–119. https://doi.org/10.1007/978-3-540-39804-2_12.
59. Seiffert, C., Khoshgoftaar, T.M., Van Hulse, J., and Napolitano, A. (2010). RUSBoost: A Hybrid Approach to Alleviating Class Imbalance. IEEE Trans. Syst. Man Cybern. A. *40*, 185–197. https://doi.org/10.1109/TSMCA.2009.2029559.
60. Wei, H., Sun, B., and Jing, M. (2014). BalancedBoost: A hybrid approach for real-time network traffic classification. In 2014 23rd International Conference on Computer Communication and Networks (ICCCN), pp. 1–6. https://doi.org/10.1109/ICCCN.2014.6911833.
61. Arafat, M.Y., Hoque, S., Xu, S., and Farid, D.M. (2019). Machine learning for mining imbalanced data. IAENG Int. J. Comput. Sci. *46*, 332–348.

62. Juwara, L., and El Emam, K. (2024). Bias mitigation using SMA. Open Science Framework. https://doi.org/10.17605/OSF.IO/RKF9T.

63. Emam, K.E., Mosquera, L., and Zheng, C. (2021). Optimizing the synthesis of clinical trial data using sequential trees. J. Am. Med. Inf. Assoc. *28*, 3–13. https://doi.org/10.1093/jamia/ocaa249.

64. Drechsler, J., and Reiter, J.P. (2011). An empirical evaluation of easily implemented, nonparametric methods for generating synthetic datasets. Comput. Stat. Data Anal. *55*, 3232–3243. https://doi.org/10.1016/j.csda.2011.06.006.

65. Arslan, R.C., Schilling, K.M., Gerlach, T.M., and Penke, L. (2021). Using 26,000 diary entries to show ovulatory changes in sexual desire and behavior. J. Pers. Soc. Psychol. *121*, 410–431. https://doi.org/10.1037/pspp0000208.

66. Bonnéry, D., Feng, Y., Henneberger, A.K., Johnson, T.L., Lachowicz, M., Rose, B.A., Shaw, T., Stapleton, L.M., Woolley, M.E., and Zheng, Y. (2019). The Promise and Limitations of Synthetic Data as a Strategy to Expand Access to State-Level Multi-Agency Longitudinal Data. J. Res. Educ. Eff. *12*, 616–647. https://doi.org/10.1080/19345747.2019.1631421.

67. Sabay, A., Harris, L., Bejugama, V., and Jaceldo-Siegl, K. (2018). Overcoming Small Data Limitations in Heart Disease Prediction by Using Surrogate Data. SMU Data Sci. Rev. *1*, 12. https://scholar.smu.edu/datasciencereview/vol1/iss3/12.

68. Freiman, M., Lauger, A., and Reiter, J. (2017). Data Synthesis and Perturbation for the American Community Survey at the (U.S. Census Bureau (US Census Bureau). https://www.census.gov/library/working-papers/2018/adrm/formal-privacy-synthetic-data-acs.html).

69. Nowok, B. (2015). Utility of synthetic microdata generated using tree-based methods. In. https://unece.org/statistics/events/SDC2015.

70. Raab, G.M., Nowok, B., and Dibben, C. (2018). Practical Data Synthesis for Large Samples. J. Priv. Confid. *7*, 67–97. https://doi.org/10.29012/jpc.v7i3.407.

71. Nowok, B., Raab, G.M., and Dibben, C. (2017). Providing bespoke synthetic data for the UK Longitudinal Studies and other sensitive data with the synthpop package for R 1. Stat. J. IAOS *33*, 785–796. https://doi.org/10.3233/SJI-150153.

72. Quintana, D.S. (2020). A synthetic dataset primer for the biobehavioural sciences to promote reproducibility and hypothesis generation. Elife *9*, e53275. https://doi.org/10.7554/eLife.53275.

73. Little, C., Elliot, M., Allmendinger, R., and Samani, S. (2021). Generative adversarial networks for synthetic data generation: A comparative study. In United Nations Economic Commission for Europe, p. 17. https://unece.org/statistics/documents/2021/12/working-documents/generative-adversarial-networks-synthetic-data.

74. Taub, J., Elliot, M., and Sakshaug, W. (2020). The Impact of Synthetic Data Generation on Data Utility with Application to the 1991 UK Samples of Anonymised Records. Trans. Data Priv. *13*, 1–23. http://www.tdp.cat/issues16/abs.a306a18.php.

75. Bühlmann, P., and Hothorn, T. (2007). Boosting Algorithms: Regularization, Prediction and Model Fitting. Stat. Sci. *22*, 477–505. https://doi.org/10.1214/07-STS242.

76. Ke, G., Meng, Q., Finley, T., Wang, T., Chen, W., Ma, W., Ye, Q., and Liu, T.-Y. (2017). LightGBM: A Highly Efficient Gradient Boosting Decision Tree. In Advances in Neural Information Processing Systems 30, I. Guyon, U.V. Luxburg, S. Bengio, H. Wallach, R. Fergus, S. Vishwanathan, and R. Garnett, eds. (Curran Associates, Inc.), pp. 3146–3154. https://papers.nips.cc/paper_files/paper/2017/hash/6449f44a102fde848669bdd9eb6b76fa-Abstract.html.

77. Snoek, J., Larochelle, H., and Adams, R.P. (2012). Practical Bayesian optimization of machine learning algorithms. In Proceedings of the 25th International Conference on Neural Information Processing Systems - Volume 2 NIPS'12 (Curran Associates Inc.)), pp. 2951–2959. https://papers.nips.cc/paper_files/paper/2012/hash/05311655a15b75fab86956663e1819cd-Abstract.html.

78. Jones, M.C. (1993). Simple boundary correction for kernel density estimation. Stat. Comput. *3*, 135–146. https://doi.org/10.1007/BF00147776.

79. Borisov, V., Leemann, T., Seßler, K., Haug, J., Pawelczyk, M., and Kasneci, G. (2022). Deep Neural Networks and Tabular Data: A Survey. IEEE Transact. Neural Networks Learn. Syst. *1–21*, 1–21. https://doi.org/10.1109/TNNLS.2022.3229161.

80. Bojer, C.S., and Meldgaard, J.P. (2021). Kaggle forecasting competitions: An overlooked learning opportunity. Int. J. Forecast. *37*, 587–603. https://doi.org/10.1016/j.ijforecast.2020.07.007.

81. Shwartz-Ziv, R., and Armon, A. (2022). Tabular data: Deep learning is not all you need. Inf. Fusion *81*, 84–90. https://doi.org/10.1016/j.inffus.2021.11.011.

82. Grinsztajn, L., Oyallon, E., and Varoquaux, G. (2022). Why do tree-based models still outperform deep learning on typical tabular data? Adv. Neural Inf. Process. Syst. *35*, 507–520. https://papers.nips.cc/paper_files/paper/2022/hash/0378c7692da36807bdec87ab043cdadc-Abstract-Datasets_and_Benchmarks.html.

83. Pathare, A., Mangrulkar, R., Suvarna, K., Parekh, A., Thakur, G., and Gawade, A. (2023). Comparison of tabular synthetic data generation techniques using propensity and cluster log metric. Int. J. Inf. Manag. Data Insights *3*, 100177. https://doi.org/10.1016/j.jjimei.2023.100177.

84. El Kababji, S., Mitsakakis, N., Fang, X., Beltran-Bless, A.-A., Pond, G., Vandermeer, L., Radhakrishnan, D., Mosquera, L., Paterson, A., Shepherd, L., et al. (2023). Evaluating the Utility and Privacy of Synthetic Breast Cancer Clinical Trial Data Sets. JCO Clin. Cancer Inform. *7*, e2300116. https://doi.org/10.1200/CCI.23.00116.

85. Niculescu-Mizil, A., and Caruana, R.A. (2012). Obtaining Calibrated Probabilities from Boosting. Preprint at arXiv. https://doi.org/10.48550/arXiv.1207.1403.

86. Kull, M., Filho, T.S., and Flach, P. (2017). Beta calibration: a well-founded and easily implemented improvement on logistic calibration for binary classifiers. In Proceedings of the 20th International Conference on Artificial Intelligence and Statistics (PMLR), pp. 623–631. https://proceedings.mlr.press/v54/kull17a.html.

87. Collins, G.S., Reitsma, J.B., Altman, D.G., and Moons, K.G.M. (2015). Transparent reporting of a multivariable prediction model for individual prognosis or diagnosis (TRIPOD): the TRIPOD statement. BMJ *350*, g7594. https://doi.org/10.1136/bmj.g7594.

88. Christodoulou, E., Ma, J., Collins, G.S., Steyerberg, E.W., Verbakel, J.Y., and Van Calster, B. (2019). A systematic review shows no performance benefit of machine learning over logistic regression for clinical prediction models. J. Clin. Epidemiol. *110*, 12–22. https://doi.org/10.1016/j.jclinepi.2019.02.004.

89. Shin, J., Ramdas, A., and Rinaldo, A. (2020). On Conditional Versus Marginal Bias in Multi-Armed Bandits. In Proceedings of the 37th International Conference on Machine Learning (PMLR), pp. 8852–8861. https://proceedings.mlr.press/v119/shin20a.html.

90. Goldberger, A.S. (1981). Linear regression after selection. J. Econom. *15*, 357–366. https://doi.org/10.1016/0304-4076(81)90100-7.

91. Heitjan, D.F., and Basu, S. (1996). Distinguishing "Missing at Random" and "Missing Completely at Random.". Am. Statistician *50*, 207–213. https://doi.org/10.1080/00031305.1996.10474381.

92. Karr, A.F., Kohnen, C.N., Oganian, A., Reiter, J.P., and Sanil, A.P. (2006). A Framework for Evaluating the Utility of Data Altered to Protect Confidentiality. Am. Statistician *60*, 224–232. https://doi.org/10.1198/000313006X124640.

93. Dwork, C., Hardt, M., Pitassi, T., Reingold, O., and Zemel, R. (2012). Fairness through awareness. In Proceedings of the 3rd Innovations in Theoretical Computer Science Conference ITCS '12 (Association for Computing Machinery)), pp. 214–226. https://doi.org/10.1145/2090236.2090255.

94. Hardt, M., Price, E., and Srebro, N. (2016). Equality of Opportunity in Supervised Learning. Preprint at arXiv. https://doi.org/10.48550/arXiv.1610.02413.

CellPress
OPEN ACCESS

95. Yan, S., Kao, H., and Ferrara, E. (2020). Fair Class Balancing: Enhancing Model Fairness without Observing Sensitive Attributes. In Proceedings of the 29th ACM International Conference on Information & Knowledge Management CIKM '20 (Association for Computing Machinery)), pp. 1715–1724. https://doi.org/10.1145/3340531.3411980.

96. Lipsey, M.W., and Wilson, D.B. (1993). The efficacy of psychological, educational, and behavioral treatment: Confirmation from meta-analysis. Am. Psychol. *48*, 1181–1209. https://www.taylorfrancis.com/chapters/edit/10.4324/9781315089256-4/efficacy-psychological-educational-behavioral-treatment-mark-lipsey-david-wilson.

97. Chen, H., Cohen, P., and Chen, S. (2010). How Big is a Big Odds Ratio? Interpreting the Magnitudes of Odds Ratios in Epidemiological Studies. Commun. Stat. Simulat. Comput. *39*, 860–864. https://doi.org/10.1080/03610911003650383.

98. Government of Canada, S.C (2015). Canadian Community Health Survey (CCHS) - 2014. https://www23.statcan.gc.ca/imdb/p3Instr.pl?Function=assembleInstr&Item_Id=214314.

99. Green, A.K., Reeder-Hayes, K.E., Corty, R.W., Basch, E., Milowsky, M.I., Dusetzina, S.B., Bennett, A.V., and Wood, W.A. (2015). The Project Data Sphere Initiative: Accelerating Cancer Research by Sharing Data. Oncol. *20*, 464–e20. https://doi.org/10.1634/theoncologist.2014-0431.

100. El-Hussuna, A., Lytras, T., Bruun, N.H., Klein, M.F., Emile, S.H., and Qvist, N. (2023). Extended Right-Sided Colon Resection Does Not Reduce the Risk of Colon Cancer Local-Regional Recurrence: Nationwide Population-Based Study from Danish Colorectal Cancer Group Database. Dis. Colon Rectum *66*, 1056–1066. https://doi.org/10.1097/DCR.0000000000002358.

101. Zwitter, M., and Soklic, M. (2015). University Medical Centre, Institute of Oncology, Ljubljana, Yugoslavia. UCI Mach. Learn. Repos. https://doi.org/10.24432/C51P4M.

Patterns, Volume *5*

Supplemental information

An evaluation of synthetic data augmentation for mitigating covariate bias in health data

Lamin Juwara, Alaa El-Hussuna, and Khaled El Emam

Supplemental Experimental Procedures

Appendix A: Review of approaches for mitigating data bias

Bias mitigation techniques can broadly be classified into three categories: rebalancing approaches, algorithmic approaches, and post-processing approaches. Some of these approaches are review below.

A.1 Rebalancing Approaches

Rebalancing approaches attempt to construct datasets that are reflective of the true population. Starting with the imbalance dataset D^I, the goal is to produce a balanced dataset D^b that provides a good approximation for the underlying data population D. The approaches can take the form of subsampling (e.g., random undersampling, oversampling, and SMOTE) or matching (e.g., propensity score matching) to remove the effect of imbalance introduced by the biased covariate. Although subsampling remains a popular method for addressing outcome imbalance, we restrict our usage of the approach to rebalance imbalanced covariates distributions.

A.1.1 Random Undersampling (RUS)

RUS is a data pre-processing approach to rebalance unevenly distributed classes in imbalanced datasets. It attains balance by randomly removing excess observations in the majority class of the primary biased covariate [1]. RUS is often extensively applied when analyzing uneven outcome class distributions [2], however, we adopt its use for rebalancing the class distributions in categorical covariates in biased datasets [3]. Although RUS has the advantage of reducing the computational cost associated with learning from excess data, it also introduces several challenges. It has the potential to distort the distributions of other relevant covariates, reduce statistical power especially in extreme class imbalance cases, and can even introduce bias in other covariates in the dataset [4].

A.1.2 Random Oversampling (ROS)

ROS attempts to rebalance covariate distributions by repeating the entries of randomly sampled observations of the minority class. The resulting cohort is a larger dataset that is comprised of repeated observations and well-represented covariate distributions [5]. While ROS has the advantage of improving the stability and performance of learning algorithms applied to the dataset by mitigating convergence issues, it is usually argued that the repeated observations barely add to the accuracy during statistical learning (e.g., classification). This is especially true when the minority class is not well-represented in the biased dataset. Another way of rationalizing the idea of oversampling is that we are assigning higher weights to the minority classes during statistical learning [6].

A.1.3 Synthetic Minority Oversampling Technique (SMOTE)

Sampling with replacement of the minority class of the imbalanced covariate often creates data cohorts that overfit during statistical learning [7]. Hence, an alternative approach to replicating instances of the minority class is to generate synthetic copies through interpolation among neighboring minority instances. This approach is termed SMOTE [8]. The approach is widely used by the data mining community as the standard pre-processing tool for imbalanced datasets.

Unlike ROS, the main appeal of SMOTE is that it adds new plausible observations that are sampled from the neighborhood of the minority class -- and not simply repeated entries. Since the seminal paper by Chawla et al, various modifications and advancements of SMOTE have seen proposed. A thorough review of the major advancements in SMOTE-based approaches to mitigate imbalanced datasets is presented in [9,10].

A.1.4 Propensity Score Matching

While global balancing might not always be possible, it is sometimes more feasible to balance the dataset within strata. Denote the set of disjoint strata making up X by $S = (S_1, S_2, \ldots, S_k)$. Suppose that X is independent of the binary predictor Z (0,1), we can say that the dataset is balanced if

$$f_{X|Z:S}(X=x|Z=1 : x \in S) = f_{X|Z:S}(X=x|Z=0 : x \in S). \qquad \text{(A1)}$$

As it turns out, this is feasible if S could be written in terms of the statistics $e(X)$ such that the conditional distribution $f_{X|Z,e(X)}(x/z,e)$ is functionally independent of z. That is, we have that $Z \perp\!\!\!\perp X|e(X)$. The term $e(X)$ can be expressed as the probability of being treated conditional on the individual's background (baseline) characteristics:

$$e(x) = \Pr[Z=1|x] = \text{expit}(x\beta), \qquad \text{(A2)}$$

where the function expit(t)=exp(t)/(1+exp(t)). Intuitively, the propensity score is a measure of the likelihood that an individual would have been treated based on his or her baseline characteristics. It is usually estimated using logistic regression with baseline characteristics as the predictor variables in the model.

Propensity score adjustment can take place in the form of matching, stratification, or regression (covariance) adjustment. While matching and stratification are usually applied to the dataset before statistical modeling to construct balance and appropriate comparisons among the baseline covariate, adjustment with propensity scores is employed at the analyses stage by including the scores (as weights) directly into the regression model.

In this manuscript, we restrict our evaluation to propensity score adjustment to rebalance the biased data during the analysis phase. This approach estimates the likelihood of an individual's group assignment (minority or majority group) based on the observed covariates, resulting in a propensity score. Subsequently, we match individuals with comparable propensity scores from minority and majority groups of the biasing covariate, effectively minimizing the influence of biased data.

A.2 Algorithmic approaches for mitigating covariate imbalance

Algorithmic methods mitigate the problem of covariate imbalance by targeting the learning stage of the analyses. It encompasses approaches that adapt commonly used learning algorithms to penalize models for learning from the minority class of the imbalanced data. These approaches can broadly be grouped into ensemble-based learning [11], cost-sensitive learning [12], and single-class learning [13]. We briefly describe each learning approach and refer readers to some comprehensive reviews.

A.2.1 Ensemble-based learning

In the data mining literature, ensemble learning refers to a group of methods that combine multiple base learners (or inducers) to make decisions. For biased dataset with imbalanced covariate distribution, the base learners are usually built on decision trees or neural network models due to their flexibility and robustness in handling complex datasets. The approach is often hailed for its ability to utilize the performance of multiple learners to improve overall performance. Dong et al. [14] provide an extensive survey of ensemble-based learning methods for dealing with complex datasets, including imbalanced covariates. Thus, the manuscript evaluates the performance of ensemble-based learning for mitigating the impact of bias in biased datasets.

A.2.2 Other algorithmic methods

Two other methods that we summarize here in this category but do not evaluate are: cost-sensitive learning [15–17] and single class learning.

The former takes the cost of misclassification (e.g., the error rate) into account during model training and is particularly well-suited for developing predictive models. That is, they are built on the idea of improving prediction accuracy rather than ensuring the stability or robustness of the estimated parameters. For example, CS-SVM algorithms allocate different costs for learning from the majority class versus learning from the minority class [18,19]. In a high imbalance binary covariate, this would imply assigning a high misclassification cost for the minority group while giving less weight to misclassifying the majority group. For a detailed survey of CS-L learning methods, see the reviews [12,20].

In the data mining literature, one-class learning is defined as a data modeling approach that attempts to construct an algorithm by learning primarily on a training set comprising only one class [21,22]. In some ways, single-class learning can be viewed as a stratified analysis of the data cohort involving only the minority or majority class [23,24]. For the most part, these algorithms focus on learning the data cohort of the majority class. However, some approaches have also been developed for well-structured cohorts of the minority class [13].

A.3 Post hoc approaches for imbalanced data

When the effect of data bias cannot be addressed during pre-processing (e.g., data cleaning) or the modeling stage, it is sometimes possible to employ post hoc adjustment techniques to account for the effect of learning from biased data. For example, in genetic studies of gene-based rare variant associations, bootstrap resampling approaches are often employed to adjust for bias in single-marker tests on common variants in GWAS studies [25]. In most cases, post hoc adjustments are only recommended as last-resort options when bias could not be mitigated in the early stages of the study [26].

Appendix B: Simulated Data

B.1 Data generation process

First, we consider a simulation setting involving a binary outcome Y, binary predictor variable Z sampled from the binomial(p=0.5), two binary variables X_1 and X_2 with the observations of X_2 given Z=1 sampled from the binomial(p=0.4) while the remaining observations of X2 given Z=0 were sampled from binomial(p=0.35). We also considered a relevant continuous predictor of the outcome U generated from the log-Normal(12,3.5) and independent of the other covariates. An effect modifier term Z*X2 for the interaction between Z and X_2 was also included. We postulate the logistic regression model:

$$P(Y=1) = \text{expit}(\alpha + \beta_Z Z + \beta_{X1} X_1 + \beta_{X2} X_2 + \beta_{ZX2} ZX_2 + \beta_U U), \qquad \text{(B1)}$$

where expit(t) = exp(t)/(1+exp(t)). For the set of fixed parameters {α=log(0.5), β_Z= log(1.25), β_{X1}= log(0.3), β_{X2}= log(2), β_{ZX2}=-0.47, β_U= log(0.5)}, we construct the full cohort data (the ground truth) before inducing bias in the sampled training cohort. The simulation is repeated to generate 500 datasets. A summary of the distributions of the simulated cohort stratified by the predictor variable categories in provided in the Table below.

	level	Stratified by Z		
		0	1	p test
n		2,521	2,479	
Y (mean (SD))		0.04 (0.19)	0.04 (0.21)	0.182
X1 (%)	0	1055 (41.8)	1014 (40.9)	0.516
	1	1466 (58.2)	1465 (59.1)	
X2 (%)	0	1746 (69.3)	1610 (64.9)	0.001
	1	775 (30.7)	869 (35.1)	
ZX2 (%)	0	2521 (100.0)	1610 (64.9)	<0.001
	1	0 (0.0)	869 (35.1)	
U (mean (SD))		3.40 (0.62)	3.43 (0.60)	0.080

Table S1: Full cohort data. Distributions of simulated observations stratified by the biased covariate variable Z=(0,1). Observations are simulated such that the binary covariate X1 is weakly/moderately correlated with the biased covariate Z while X2 is strongly correlated with the binary predictor. Z is balanced among the outcome categories.

We also present the distributions of the variables in the training cohort (sampled simulated example) and the distributions of the corresponding biased datasets (i.e., the marginal and conditionally biased datasets).

		level	Stratified by Z 0	1	p test
Training set	n		1,894	1,856	
	Y (mean (SD))		0.05 (0.21)	0.05 (0.21)	0.921
	X1 (%)	0	781 (41.2)	738 (39.8)	0.376
		1	1113 (58.8)	1118 (60.2)	
	X2 (%)	0	1311 (69.2)	1219 (65.7)	0.023
		1	583 (30.8)	637 (34.3)	
	ZX2 (%)	0	1894 (100.0)	1219 (65.7)	<0.001
		1	0 (0.0)	637 (34.3)	
	U (mean (SD))		3.41 (0.66)	3.43 (0.62)	0.316
Marginal bias	n		947	1,856	
	Y (mean (SD))		0.05 (0.22)	0.05 (0.21)	0.376
	X1 (%)	0	400 (42.2)	738 (39.8)	0.222
		1	547 (57.8)	1118 (60.2)	
	X2 (%)	0	641 (67.7)	1219 (65.7)	0.307
		1	306 (32.3)	637 (34.3)	
	ZX2 (%)	0	947 (100.0)	1219 (65.7)	<0.001
		1	0 (0.0)	637 (34.3)	
	U (mean (SD))		3.40 (0.69)	3.43 (0.62)	0.135
Conditional bias I	n		1,504	1,856	
	Y (mean (SD))		0.04 (0.20)	0.05 (0.21)	0.704
	X1 (%)	0	391 (26.0)	738 (39.8)	<0.001
		1	1113 (74.0)	1118 (60.2)	
	X2 (%)	0	1041 (69.2)	1219 (65.7)	0.033
		1	463 (30.8)	637 (34.3)	
	ZX2 (%)	0	1504 (100.0)	1219 (65.7)	<0.001
		1	0 (0.0)	637 (34.3)	
	U (mean (SD))		3.41 (0.66)	3.43 (0.62)	0.363
Conditional bias II	n		1,894	1,856	
	Y (mean (SD))		0.05 (0.21)	0.05 (0.21)	0.921
	X1 (%)	0	781 (41.2)	738 (39.8)	0.376
		1	1113 (58.8)	1118 (60.2)	
	X2 (%)	0	1311 (69.2)	1219 (65.7)	0.023
		1	583 (30.8)	637 (34.3)	
	ZX2 (%)	0	1894 (100.0)	1219 (65.7)	<0.001
		1	0 (0.0)	637 (34.3)	
	U (mean (SD))		3.41 (0.66)	3.43 (0.62)	0.316

Table S2: Distributed of training samples (original training set, marginal bias, and conditional bias) stratified by the biased covariate Z.

B.2 Additional results for simulated data

Missing	Approach	Marginal bias			Conditional bias I			Conditional bias II		
		**AUC**	**OR_Z (SD)**	**I_Z**	**AUC**	**OR_Z (SD)**	**I_Z**	**AUC**	**OR_Z (SD)**	**I_Z**
	Biased	0.72	1.30 (0.15)	-	0.72	1.30 (0.16)	-	0.72	1.30 (0.15)	-
	RUS	0.72	1.28 (0.15)	0.98	0.72	1.28 (0.16)	0.94	0.71	1.28 (0.15)	0.95
	ROS	0.72	1.27 (0.14)	0.98	0.72	1.29 (0.14)	0.93	0.72	1.27 (0.14)	0.94
15%	SMOTE	0.71	1.14 (1.18)	0.74	0.69	1.19 (1.18)	0.88	0.70	1.18 (1.04)	0.84
	PS-match	0.72	1.19 (0.16)	0.92	0.72	1.18 (0.16)	0.92	0.72	1.23 (0.16)	0.96
	RF	0.70	-	-	0.69	-	-	0.69	-	-
	SMA‡	0.72	1.25 (0.14)	0.98	0.72	1.27 (0.14)	0.95	0.72	1.26 (0.14)	0.95
	Biased	0.71	1.34 (0.17)	-	0.72	1.38 (0.15)	-	0.72	1.27 (0.11)	-
	RUS	0.72	1.36 (0.14)	0.97	0.71	1.36 (0.15)	0.87	0.71	1.26 (0.12)	0.89
	ROS	0.72	1.28 (0.14)	0.98	0.72	1.46 (0.17)	0.84	0.71	1.22 (0.11)	0.90
30%	SMOTE	0.70	0.88 (1.17)	0.34	0.70	1.02 (0.19)	0.60	0.70	0.90 (0.14)	0.24
	PS-match	0.71	1.13 (0.15)	0.84	0.71	1.41 (0.14)	0.94	0.71	1.37 (0.11)	0.71
	RF	0.69	-	-	0.69	-	-	0.69	-	-
	SMA‡	0.72	1.25 (0.14)	0.98	0.72	1.31 (0.15)	0.95	0.72	1.24 (0.07)	0.91
	Biased	0.71	1.49 (0.19)	-	0.72	1.31 (0.15)	-	0.72	1.43 (0.19)	-
	RUS	0.70	1.38 (0.14)	0.85	0.72	1.30 (0.14)	0.89	0.71	1.41 (0.20)	0.84
	ROS	0.71	1.45 (0.16)	0.78	0.72	1.29 (0.16)	0.90	0.71	1.51 (0.19)	0.82
50%	SMOTE	0.68	0.94 (0.21)	0.71	0.69	1.02 (0.21)	0.49	0.68	0.91 (0.18)	0.41
	PS-match	0.70	1.44 (0.26)	0.72	0.71	1.32 (0.13)	0.95	0.70	1.45 (0.24)	0.78
	RF	0.69	-	-	0.68	-	-	0.70	-	-
	SMA‡	0.71	1.24 (0.19)	0.93	0.71	1.29 (0.13)	0.90	0.72	1.39 (0.14)	0.88
	Biased	0.68	1.00 (0.25)	-	0.71	1.32 (0.19)	-	0.70	1.09 (0.22)	-
	RUS	0.68	1.17 (0.28)	0.97	0.71	1.41 (0.21)	0.72	0.70	1.03 (0.27)	0.76
	ROS	0.68	1.02 (0.24)	0.98	0.72	1.28 (0.24)	0.75	0.70	0.97 (0.23)	0.84
80%	SMOTE	0.67	0.73 (0.21)	0.34	0.69	1.05 (0.20)	0.60	0.70	0.90 (1.15)	0.33
	PS-match	0.65	0.50 (0.95)	0.84	0.70	1.54 (0.15)	0.81	0.69	1.08 (0.40)	0.66
	RF	0.66	-	-	0.68	-	-	0.69	-	-
	SMA‡	0.69	0.96 (0.51)	0.98	0.70	1.33 (0.24)	0.77	0.70	1.24 (0.16)	0.91
	Biased	0.64	1.00 (0.94)	-	0.60	1.02 (1.05)	-	0.61	1.00 (0.82)	-
	RUS	0.64	1.17 (0.73)	0.55	0.60	1.01 (1.03)	0.52	0.60	1.01 (0.67)	0.46
	ROS	0.65	1.00 (0.56)	0.60	0.62	1.04 (1.02)	0.55	0.60	0.86 (0.55)	0.49
95%	SMOTE	0.67	0.69 (1.74)	0.44	0.59	1.05 (1.21)	0.52	0.58	0.98 (1.64)	0.53
	PS-match	0.65	0.64 (1.68)	0.49	0.60	1.04 (0.99)	0.54	0.59	1.01 (0.63)	0.52
	RF	0.61	-	-	0.58	-	-	0.60	-	-
	SMA‡	0.65	0.97 (1.05)	0.68	0.60	1.03 (0.81)	0.67	0.61	1.12 (0.46)	0.78

NB: Synthetic Minor Augmentation (SMA)

Table S3: Simulations for the full model. Estimates of the mean AUC, mean Odds Ratio associated with Z and the standard deviation (OR_Z(SD)), and the confidence interval overlaps of the biased covariate effect with the ground truth (I_Z) from 500 repetitions. Missing prop indicates the proportion of observations removed under each bias setting. For the Original data: AUC = 0.72; OR_Z(SD) = 1.24(0.15); I_Z =1.00. The proportion ranges from 15% to 95%. ǂ indicates that the estimates are averaged from m=100 synthetic copies.

**Missing**	**15%**			**30%**			**50%**			**80%**			**95%**		
**Approach**	**MB**	**CB1**	**CBII**	**MB**	**CBI**	**CBII**	**MB**	**CBI**	**CBII**	**MB**	**CBI**	**CBII**	**MB**	**CBI**	**CBII**
Biased	0.70	0.70	0.70	0.70	0.70	0.70	0.69	0.69	0.70	0.67	0.67	0.68	0.50	0.50	0.51
RUS	0.70	0.70	0.70	0.70	0.70	0.70	0.70	0.70	0.70	0.60	0.60	0.61	0.49	0.49	0.52
ROS	0.70	0.70	0.70	0.70	0.70	0.70	0.70	0.70	0.71	0.68	0.68	0.68	0.49	0.49	0.54
SMOTE	0.69	0.69	0.69	0.69	0.69	0.69	0.69	0.69	0.69	0.69	0.69	0.70	0.50	0.50	0.50
PSM	0.71	0.71	0.70	0.71	0.71	0.70	0.70	0.70	0.70	0.62	0.62	0.65	0.50	0.50	0.50
RF	0.69	0.69	0.69	0.67	0.67	0.67	0.66	0.66	0.67	0.65	0.65	0.65	0.51	0.51	0.49
SMAǂ	0.70	0.70	0.70	0.70	0.70	0.70	0.70	0.70	0.70	0.68	0.68	0.67	0.50	0.50	0.50

Table S4: Simulations for the stratified model indicating the AUCs for predicting the minority group of the biased covariate under each bias mitigation method. The proportion of observations removed ranges from 15% to 95%. The reference estimates for the minority category of the original cohort (β_Z = log(1.25)) is provided: Minority category AUC = 0.71.

**Missing prop**	**15%**			**30%**			**50%**			**80%**			**95%**		
**Approach**	**MB**	**CB1**	**CBII**	**MB**	**MB**	**CBI**	**MB**	**CBI**	**CBII**	**MB**	**CBI**	**CBII**	**MB**	**CBI**	**CBII**
Biased data	0.04	0.04	0.04	0.04	0.04	0.04	0.04	0.04	0.04	0.04	0.04	0.04	0.04	0.04	0.04
RUS	0.04	0.04	0.04	0.04	0.04	0.04	0.04	0.04	0.04	0.04	0.04	0.04	0.04	0.04	0.04
ROS	0.04	0.04	0.04	0.04	0.04	0.04	0.04	0.04	0.04	0.04	0.04	0.04	0.04	0.04	0.04
SMOTE	0.04	0.04	0.04	0.04	0.04	0.04	0.04	0.04	0.04	0.05	0.04	0.04	0.04	0.05	0.05
PS-matching	0.04	0.04	0.04	0.04	0.04	0.04	0.04	0.04	0.04	0.04	0.04	0.04	0.04	0.04	0.04
RF ensemble	0.06	0.06	0.06	0.06	0.06	0.06	0.06	0.06	0.06	0.06	0.06	0.06	0.06	0.06	0.06
SMA	0.04	0.03	0.03	0.04	0.03	0.03	0.04	0.03	0.03	0.04	0.03	0.03	0.04	0.03	0.03

Table S5: Simulations for the stratified model indicating the Brier scores for predicting the minority group of the biased covariate under each bias mitigation method. The proportion of observations removed ranges from 15% to 95%. The reference estimates for the minority category of the original cohort (β_Z = log(1.25)) is provided: Minority category Brier Score = 0.03.

Fairness	Type	Missing	Biased	RUS	ROS	SMOTE	PSM	SMA‡
SPD Original = -0.045	Marginal	15%	-0.067	-0.037	-0.065	-0.187	0.069	-0.045
		30%	-0.092	-0.094	-0.098	-0.182	-0.020	-0.053
		50%	-0.051	-0.065	-0.059	-0.132	0.003	-0.024
		80%	-0.076	-0.117	-0.054	-0.091	0.611	0.017
		95%	0.018	0.289	0.066	0.031	0.358	0.092
	Conditional I	15%	-0.077	-0.053	-0.063	-0.123	0.003	-0.045
		30%	-0.071	-0.106	-0.050	-0.055	0.032	-0.053
		50%	-0.073	-0.067	-0.079	-0.072	0.075	-0.024
		80%	-0.102	-0.197	-0.083	-0.066	-0.062	0.017
		95%	-0.098	-0.109	-0.105	-0.162	0.551	0.092
	Conditional II	15%	-0.069	-0.086	-0.076	-0.136	0.035	-0.066
		30%	-0.068	-0.062	-0.048	-0.170	0.037	-0.066
		50%	-0.083	-0.101	-0.097	-0.106	0.013	-0.076
		80%	-0.100	-0.083	-0.092	-0.060	-0.012	-0.079
		95%	-0.095	-0.142	-0.096	-0.119	0.571	-0.046
EOD Original = -0.079	Marginal	15%	-0.081	-0.044	-0.077	-0.208	0.081	-0.054
		30%	-0.111	-0.101	-0.112	-0.201	-0.021	-0.064
		50%	-0.063	-0.071	-0.071	-0.151	0.004	-0.030
		80%	-0.093	-0.104	-0.057	-0.103	0.666	0.021
		95%	0.023	0.353	0.103	0.044	0.407	0.120
	Conditional I	15%	-0.092	-0.064	-0.074	-0.128	0.004	-0.054
		30%	-0.086	-0.114	-0.058	-0.058	0.040	-0.064
		50%	-0.088	-0.076	-0.092	-0.086	0.098	-0.030
		80%	-0.121	-0.197	-0.093	-0.067	-0.066	0.021
		95%	-0.117	-0.115	-0.118	-0.169	0.819	0.120
	Conditional II	15%	-0.083	-0.098	-0.087	-0.138	0.040	-0.080
		30%	-0.083	-0.068	-0.057	-0.171	0.047	-0.079
		50%	-0.100	-0.124	-0.113	-0.123	0.016	-0.092
		80%	-0.120	-0.098	-0.104	-0.067	-0.013	-0.096
		95%	-0.114	-0.149	-0.111	-0.127	0.822	-0.052
AOD Original = 0.071	Marginal	15%	0.073	0.040	0.070	0.196	0.075	0.049
		30%	0.100	0.097	0.104	0.191	0.020	0.058
		50%	0.056	0.068	0.064	0.141	0.004	0.026
		80%	0.084	0.111	0.055	0.096	0.637	0.019
		95%	0.020	0.317	0.082	0.037	0.382	0.104
		15%	0.084	0.058	0.068	0.125	0.003	0.049

	Conditional I	30%	0.077	0.110	0.054	0.057	0.036	0.058
		50%	0.080	0.071	0.085	0.078	0.086	0.026
		80%	0.110	0.197	0.087	0.066	0.064	0.019
		95%	0.106	0.111	0.111	0.165	0.679	0.104
	Conditional II	15%	0.075	0.091	0.081	0.137	0.038	0.072
		30%	0.075	0.065	0.052	0.171	0.042	0.072
		50%	0.091	0.112	0.104	0.113	0.014	0.083
		80%	0.109	0.090	0.098	0.063	0.013	0.087
		95%	0.103	0.145	0.102	0.123	0.691	0.048

Table S6. Estimates of group fairness based on statistical parity difference (SPD), equal opportunity difference (EOD), and average odds difference (AOD). The estimates reported for each metric represent average values computed over 500 simulated samples. Fairness estimates shown for the original data (ground truth) are considered as the benchmark. ǂ indicates that the estimates are averaged from m=100 synthetic copies. PSM is Propensity Score matching.

B.3 Sensitivity analyses results

In this section, we provide additional sensitivity analysis results for estimating the mean Odds ratio of the predictor Z. We conducted 500 repetitions with various effect sizes β_Z =(log(0.5), log(1.01), log(2)), and considered missing proportions ranging from 30% to 80%. All estimates using the Simple Moving Average (SMA) technique were aggregated from a total of m=100 synthetic data samples.

Missing prop	Approach	β_2 = log (0.5)			β_2 = log (1.01)			β_2 = log (2.0)		
		MB	CB1	CBII	MB	CB1	CBII	MB	CB1	CBII
15%	Biased data	0.50	0.48	0.47	1.04	1.07	0.88	1.99	1.90	2.26
	RUS	0.48	0.38	0.55	0.99	1.06	0.91	1.95	2.05	1.90
	ROS	0.47	0.42	0.48	1.05	0.99	0.97	2.31	2.02	2.11
	SMOTE	0.44	0.29	0.34	1.02	0.95	1.14	1.32	1.74	1.98
	PS-	0.56	0.49	0.58	1.00	0.99	1.32	1.97	2.30	2.23
	RF	-	-	-	-	-	-	-	-	-
	SMA	0.50	0.49	0.51	1.03	0.97	0.95	1.99	2.08	1.94
30%	Biased data	0.51	0.47	0.34	1.01	0.95	1.11	2.06	1.82	1.75
	RUS	0.37	0.48	0.38	0.92	0.97	1.10	2.03	1.77	1.65
	ROS	0.57	0.46	0.31	0.85	1.00	1.15	2.03	1.78	1.87
	SMOTE	0.37	0.43	0.30	0.71	0.75	0.73	1.66	1.21	1.50
	PS-	0.57	0.49	0.35	1.01	0.89	1.33	2.20	1.91	1.51
	RF	-	-	-	-	-	-	-	-	-
	SMA	0.51	0.48	0.33	1.01	0.99	1.11	2.13	1.83	1.87
50%	Biased data	0.44	0.51	0.39	1.01	0.97	1.46	1.88	2.14	1.75
	RUS	0.33	0.54	0.40	0.96	0.98	1.40	1.80	1.93	1.82
	ROS	0.43	0.51	0.39	0.75	0.93	1.09	1.83	2.15	1.89
	SMOTE	0.49	0.46	0.26	0.53	0.76	0.93	1.84	1.84	1.36
	PS-	0.36	0.54	0.37	0.95	1.00	1.32	2.02	2.39	1.69
	RF	-	-	-	-	-	-	-	-	-
	SMA	0.51	0.49	0.43	1.01	0.85	1.58	1.88	2.36	1.63
80%	Biased data	0.41	0.48	0.53	0.94	1.01	1.16	1.98	1.59	1.65
	RUS	0.39	0.46	0.55	0.72	0.89	0.95	2.06	1.69	1.92
	ROS	0.42	0.51	0.42	0.80	0.91	1.09	2.04	1.56	1.92
	SMOTE	0.42	0.45	0.23	0.64	0.74	0.83	1.78	1.72	1.13
	PS-	0.39	0.53	0.57	1.34	1.02	0.97	2.70	1.72	1.37
	RF	-	-	-	-	-	-	-	-	-
	SMA	0.47	0.52	0.82	0.98	0.89	1.01	1.57	1.32	1.21
95%	Biased data	0.40	0.38	0.37	1.14	1.07	0.78	1.79	1.50	2.36
	RUS	0.38	0.33	0.71	0.79	1.06	0.91	1.85	2.55	1.50
	ROS	0.37	0.37	0.33	1.20	0.95	0.77	2.31	2.42	2.21
	SMOTE	0.34	0.21	0.24	1.12	0.99	0.49	1.32	1.74	1.98
	PS-	0.61	0.44	0.78	1.15	0.65	1.54	1.79	1.30	2.43
	RF	-	-	-	-	-	-	-	-	-
	SMA	0.45	0.36	0.41	1.11	0.87	0.75	1.59	2.38	1.19

Table S7: Estimates of the Odds Ratio of Z of the primary predictor Z from 500 repetitions. For the ground truth, the fixed values of β_Z = (log(0.5), log(1.01), log(2)). The proportion of observations removed ranges from 15% to 95%. The ground truth OR estimates for Z = (log(0.5), log(1.01), log(2)) were 0.50, 1.01, and 1.99, respectively.

Missing prop	Approach	βZ = log(0.5)			βZ = log(1.01)			βZ = log(2.0)		
		MB	CB1	CBII	MB	CBI	CBII	MB	CBI	CBII
15%	Biased data	0.70	0.70	0.70	0.70	0.70	0.70	0.70	0.70	0.70
	RUS	0.69	0.70	0.70	0.70	0.70	0.70	0.70	0.70	0.70
	ROS	0.70	0.70	0.71	0.70	0.70	0.70	0.70	0.70	0.70
	SMOTE	0.71	0.69	0.69	0.70	0.71	0.71	0.69	0.69	0.69
	PS-matching	0.71	0.70	0.70	0.69	0.69	0.69	0.71	0.70	0.70
	RF ensemble	0.66	0.67	0.67	0.70	0.70	0.70	0.69	0.69	0.68
	SMA	0.70	0.69	0.71	0.70	0.70	0.71	0.70	0.70	0.71
30%	Biased data	0.70	0.69	0.69	0.70	0.70	0.70	0.70	0.70	0.70
	RUS	0.69	0.70	0.70	0.70	0.70	0.70	0.70	0.70	0.70
	ROS	0.70	0.70	0.70	0.70	0.70	0.70	0.70	0.70	0.70
	SMOTE	0.71	0.69	0.69	0.69	0.69	0.69	0.69	0.69	0.69
	PS-matching	0.71	0.70	0.70	0.71	0.70	0.70	0.71	0.70	0.70
	RF ensemble	0.66	0.67	0.67	0.67	0.67	0.67	0.67	0.67	0.67
	SMA	0.70	0.69	0.71	0.70	0.70	0.70	0.70	0.70	0.71
50%	Biased data	0.69	0.69	0.69	0.70	0.70	0.70	0.69	0.70	0.70
	RUS	0.70	0.70	0.70	0.70	0.70	0.70	0.70	0.70	0.70
	ROS	0.70	0.71	0.71	0.70	0.71	0.71	0.70	0.71	0.71
	SMOTE	0.69	0.69	0.69	0.69	0.69	0.69	0.69	0.69	0.69
	PS-matching	0.70	0.70	0.70	0.70	0.70	0.70	0.70	0.70	0.70
	RF ensemble	0.66	0.67	0.67	0.66	0.67	0.67	0.66	0.67	0.67
	SMA	0.70	0.70	0.70	0.70	0.70	0.70	0.70	0.70	0.70
80%	Biased data	0.66	0.66	0.65	0.69	0.69	0.68	0.67	0.68	0.67
	RUS	0.60	0.61	0.60	0.60	0.61	0.58	0.60	0.61	0.58
	ROS	0.67	0.67	0.66	0.68	0.68	0.67	0.68	0.68	0.67
	SMOTE	0.69	0.65	0.69	0.69	0.70	0.69	0.69	0.70	0.69
	PS-matching	0.60	0.63	0.68	0.62	0.65	0.70	0.62	0.65	0.70
	RF ensemble	0.65	0.65	0.65	0.65	0.65	0.65	0.65	0.65	0.65
	SMA	0.66	0.67	0.65	0.68	0.67	0.68	0.68	0.67	0.65
95%	Biased data	0.61	0.61	0.53	0.50	0.50	0.52	0.50	0.51	0.51
	RUS	0.59	0.60	0.56	0.50	0.50	0.55	0.49	0.52	0.48
	ROS	0.62	0.54	0.63	0.51	0.49	0.53	0.49	0.54	0.52
	SMOTE	0.61	0.60	0.65	0.50	0.50	0.51	0.50	0.50	0.52
	PS-matching	0.60	0.57	0.59	0.50	0.50	0.52	0.50	0.50	0.55
	RF ensemble	0.59	0.56	0.55	0.50	0.50	0.55	0.51	0.49	0.53
	SMA	0.60	0.59	0.56	0.51	0.51	0.53	0.50	0.50	0.51

Table S8: Simulations for the stratified model. AUCs for predicting the minority group of the biased covariate under each bias mitigation method are shown. The proportion of observations removed ranges from 15% to 95%. The ground truth AUC estimates for Z = (log(0.5), log(1.01), log(2)) were 0.72, 0.71, and 0.72, respectively.

Appendix C: Real Datasets

This section presents a detailed description of four case studies and how data bias (marginal and conditional) is induced. While the overall summaries of the results are included in the main manuscript, the evaluations of the individual datasets have been relegated to the appendix for brevity.

C.1 Colon Cancer N0147 trial

N0147 was a Colon Cancer trial conducted between 2004 and 2009 that assigned patients with ``stage III colon cancer to adjuvant regimens of folinic acid, fluorouracil, and oxaliplatin or fluorouracil, leucovorin, and irinotecan, with or without cetuximab." Patient-level data from the control arms (chemotherapy-only) were obtained which comprised 1543 observations and 10 variables. Our analysis considered death as the outcome variable while the biased covariate of interest was bowel obstruction. The remaining predictors were all included in the postulated model except the time variable.

In the marginal bias setting, observations belonging to one arm of bowel obstruction (e.g., no bowel obstruction) were randomly sampled and removed from the training data, independent of other covariates. For the first conditional bias case, gender (which is weakly associated with bowel obstruction, $\beta = 0.167$, p-value = 0.1968) is conditional on the type I case. Thus, observations were randomly sampled and excluded from the training set if they were female and had no bowel obstruction. In the second conditional bias case, we used bmi (which is strongly associated with bowel obstruction, $\beta = 1.092$, p-value < 0.001) as the conditioning variable to induce bias in the training data. For marginal bias, observations that had no bowel obstruction were excluded from the training data, independent of other covariates in the dataset.

The amount of sampling bias was also varied by increasing the proportion of samples (sampled observations) removed under each biasing scheme. In the marginal bias case, for example, this amounts to increasing the proportion of observations removed in the minority class from low to high. In the conditional bias cases, the proportion of observations removed from each conditional case is increased from low to high.

C.2 Danish Colorectal Cancer data (DCCG)

The Danish Colorectal Cancer Group (DCCG) database is a prospectively maintained dataset of all danish patients with a first-time diagnosis of right-sided colonic cancer between 2001 and 2018. The dataset is comprised of 12855 observations and 192 attributes. Our analyses included 9 relevant predictors of "post medication complications" based on previous studies.

In the marginal bias setting, observations were randomly sampled and removed from the training set if they were female irrespective of the other covariates. We next considered two conditional bias settings by conditioning on covariates that were weakly or strongly associated with gender. In the first instance, female participants were only excluded from the training set if they were in the first category of P-PN stadium. In the second conditional case, observations were removed from the training set if they were female and had ASA. The amount of sampling

bias was also varied by increasing the proportion of samples (sampled observations) removed under each biasing scheme.

C.3 Breast Cancer (UCI)

This UCI data is provided by the Oncology Institute to predict the breast cancer [27]. It is comprised of 277 observations and 10 attributes. Our analyses included all predictors based on previous studies.

In the marginal bias case, observations were randomly sampled and removed from the training set if they belonged to the 20-49 group age category, irrespective of the other covariates. We considered two conditional bias settings: In the first conditional bias case, 20–49-year-old participants were only excluded from the training set if they the breast category was left. In the second conditional case, observations were removed from the biased covariate category (20-49 years) if they had menopause. The amount of sampling bias was also varied by increasing the proportion of samples (sampled observations) removed under each biasing scheme.

C.4 Cardiovascular Health data from CCHS-2014

For the cardiovascular health dataset, the outcome considered was the binary variable for cardiovascular health status and the biased covariate of interest was gender. The model included other relevant predictors (age, education, household income, household size, and whether the participant is a new immigrant or not) which were selected based on previous studies [28,29].

In the marginal bias case, observations were randomly sampled and removed from the training set if they were female irrespective of their other covariate values (or conversely, the same could be applied to male participants). We next considered two conditional bias settings by conditioning on covariates that were weakly or strongly associated with gender. In the first instance, female participants were only excluded from the training set if they were not new immigrants (new immigration is weakly associated with gender, p-value 0.294). In the second case, observations were removed from the training set if they were female and belonged to a specific marital category (e.g., marital status = 1). Marital status was selected as it is strongly associated with gender.

The amount of sampling bias was also varied by increasing the proportion of samples (sampled observations) removed under each biasing scheme. In the marginal bias case, for example, this amounts to increasing the proportion of observations removed in the minority class from low to high. In the conditional bias cases, the proportion of observations removed from each conditional case is increased from low to high.

Appendix D: Results for real datasets

D.1 Colon cancer N0147 trial

Missing prop	Approach	Marginal bias			Conditional bias I			Conditional bias II		
		AUC	OR_Z (SE)	I_Z	AUC	OR_Z (SE)	I_Z	AUC	OR_Z (SE)	I_Z
15%	Biased data	0.69	0.59(-)	-	0.70	0.56(-)	-	0.69	0.52(-)	-
	RUS	0.67	0.66(0.36)	0.82	0.70	0.49(0.35)	0.86	0.66	0.74(0.32)	0.71
	ROS	0.69	0.65(0.14)	0.86	0.65	0.55(0.14)	0.80	0.66	0.51(0.14)	0.76
	SMOTE	0.68	0.35(0.09)	0.33	0.69	0.55 (0.23)	0.98	0.65	0.30(0.23)	0.31
	PS-matching	0.68	1.03(0.66)	0.58	0.65	1.15(0.62)	0.57	0.65	1.13(0.61)	0.58
	RF ensemble	0.67	-	-	0.66	-	-	0.66	-	-
	SMA	0.70	0.58(0.23)	0.94	0.70	0.58(0.23)	0.94	0.70	0.53(0.23)	0.93
30%	Biased data	0.69	0.51(-)	-	0.70	0.52(-)	-	0.69	0.52(-)	-
	RUS	0.66	0.44(0.52)	0.82	0.68	0.54(0.35)	0.82	0.69	0.44(0.52)	0.82
	ROS	0.68	0.85(0.14)	0.78	0.67	0.49(0.14)	0.76	0.66	0.85(0.14)	0.78
	SMOTE	0.68	0.29(0.09)	0.00	0.66	0.29(0.09)	0.00	0.67	0.19(0.27)	0.00
	PS-matching	0.71	1.28(0.80)	0.54	0.60	1.28(0.80)	0.54	0.60	1.43(0.67)	0.52
	RF ensemble	0.66	-	-	0.65	-	-	0.65	-	-
	SMA	0.70	0.58(0.23)	0.94	0.70	0.58(0.24)	0.93	0.70	0.54(0.23)	0.98
50%	Biased data	0.69	0.62(-)	-	0.70	0.49(-)	-	0.70	0.50(-)	-
	RUS	0.67	0.57(0.52)	0.69	0.66	0.52(0.34)	0.81	0.65	0.40(0.41)	0.87
	ROS	0.67	0.85(0.15)	0.46	0.67	0.51(0.14)	0.77	0.71	0.66(0.14)	0.86
	SMOTE	0.66	0.38(0.11)	0.45	0.69	0.51(0.24)	0.90	0.66	0.63(0.24)	0.84
	PS-matching	0.63	2.48(1.08)	0.51	0.69	1.02(0.77)	0.56	0.62	1.64(0.89)	0.53
	RF ensemble	0.64	-	-	0.64	-	-	0.63	-	-
	SMA	0.70	0.59(0.24)	0.86	0.70	0.61(0.25)	0.88	0.70	0.53(0.24)	0.97
80%	Biased data	0.67	0.78(-)	-	0.70	0.49(-)	-	0.69	0.50(-)	-
	RUS	0.67	3.06(0.80)	0.24	0.71	0.68(0.42)	0.71	0.67	0.62(0.42)	0.72
	ROS	0.70	1.22(0.17)	0.01	0.68	0.55(0.14)	0.81	0.68	0.52(0.14)	0.79
	SMOTE	0.72	1.11(0.20)	0.19	0.68	0.49(0.26)	0.89	0.64	0.60(0.28)	0.87
	PS-matching	0.67	2.59(1.24)	0.50	0.58	1.99(0.78)	0.51	0.55	2.42(1.24)	0.51
	RF ensemble	0.63	-	-	0.60	-	-	0.56	-	-
	SMA	0.68	1.17(0.96)	0.06	0.67	1.69(0.52)	0.00	0.70	0.55(0.26)	0.94

Table S9: Estimates of mean AUC, Odds Ratio of Z [OR_Z(SE)], and interval overlaps (I_Z) of the biased covariate Z for each bias mitigating approach on the Colon Cancer data. For the Original data: AUC = 0.70; OR_Z(SE) = 0.55 (0.23); I_Z =1.00. Note. Synthetic Minor Augmentation (SMA).

Colon Cancer **Missing Prop**	**Approach**	**Marginal bias**			**Conditional bias I**			**Conditional bias II**		
		**SPD**	**EOD**	**AOD**	**SPD**	**EOD**	**AOD**	**SPD**	**EOD**	**AOD**
15%	Biased	-0.067	-0.081	0.073	-0.077	-0.092	0.084	-0.069	-0.083	0.075
	RUS	-0.037	-0.044	0.040	-0.053	-0.064	0.058	-0.086	-0.098	0.091
	ROS	-0.065	-0.077	0.070	-0.063	-0.074	0.068	-0.076	-0.087	0.081
	PS-matching	0.069	0.081	0.075	0.003	0.004	0.003	0.035	0.040	0.038
	SMOTE	-0.187	-0.208	0.196	-0.123	-0.128	0.125	-0.136	-0.138	0.137
	SMA	-0.045	-0.054	0.049	-0.045	-0.054	0.049	-0.066	-0.080	0.072
30%	Biased	-0.092	-0.111	0.100	-0.071	-0.086	0.077	-0.068	-0.083	0.075
	RUS	-0.094	-0.101	0.097	-0.106	-0.114	0.110	-0.062	-0.068	0.065
	ROS	-0.098	-0.112	0.104	-0.050	-0.058	0.054	-0.048	-0.057	0.052
	PS-matching	-0.020	-0.021	0.020	0.032	0.040	0.036	0.037	0.047	0.042
	SMOTE	-0.182	-0.201	0.191	-0.055	-0.058	0.057	-0.170	-0.171	0.171
	SMA	-0.053	0.079	0.058	-0.053	-0.064	0.058	-0.066	-0.079	0.072
50%	Biased	-0.051	-0.063	0.056	-0.073	-0.088	0.080	-0.083	-0.100	0.091
	RUS	-0.065	-0.071	0.068	-0.067	-0.076	0.071	-0.101	-0.124	0.112
	ROS	-0.059	-0.071	0.064	-0.079	-0.092	0.085	-0.097	-0.113	0.104
	PS-matching	0.003	0.004	0.004	0.075	0.098	0.086	0.013	0.016	0.014
	SMOTE	-0.132	-0.151	0.141	-0.072	-0.086	0.078	-0.106	-0.123	0.113
	SMA	-0.024	-0.030	0.026	-0.024	-0.030	0.026	-0.076	-0.092	0.083
80%	Biased	-0.076	-0.093	0.084	-0.102	-0.121	0.110	-0.100	-0.120	0.109
	RUS	-0.117	-0.104	0.111	-0.197	-0.197	0.197	-0.083	-0.098	0.090
	ROS	-0.054	-0.057	0.055	-0.083	-0.093	0.087	-0.092	-0.104	0.098
	PS-matching	0.611	0.666	0.637	-0.062	-0.066	0.064	-0.012	-0.013	0.013
	SMOTE	-0.091	-0.103	0.096	-0.066	-0.067	0.066	-0.060	-0.067	0.063
	SMA	0.017	0.021	0.019	0.017	0.021	0.019	-0.079	-0.096	0.087

Table S10: Fairness metrics: the statistical parity difference (SPD), equal opportunity difference (EOD), and average odds difference (AOD) from the logistic regression model on the colon cancer data. The proportion of samples removed varied from 15% to 80%. Original cohort: SPD = -0.066, EOD = -0.079, and AOD = 0.071

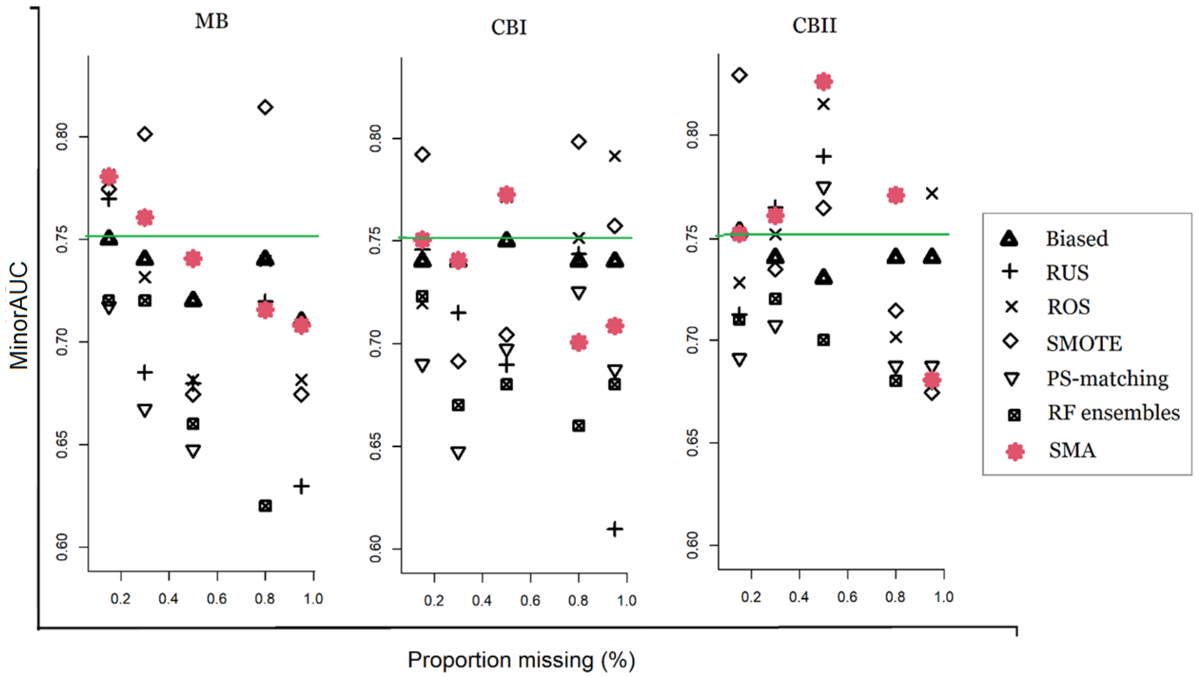

Figure S1: Colon Cancer data used for the stratified model. Plots of AUC estimates of the minor categories of the biased covariate. MB denotes marginal bias, CBI for conditional bias I, and CBII for conditional bias II. The horizontal line is the ground truth estimate (AUC =0.76).

Missing	***15%***			***30%***			***50%***			***80%***			***95%***		
**Approach**	**MB**	**CB1**	**CBII**	**MB**	**MB**	**CBI**	**MB**	**CBI**	**CBII**	**MB**	**CBI**	**CBII**	**MB**	**CBI**	**CBII**
Biased data	0.04	0.04	0.04	0.04	0.04	0.04	0.04	0.04	0.04	0.04	0.04	0.04	0.04	0.04	0.04
RUS	0.04	0.04	0.04	0.04	0.04	0.04	0.04	0.04	0.04	0.04	0.04	0.04	0.04	0.04	0.04
ROS	0.04	0.04	0.04	0.04	0.04	0.04	0.04	0.04	0.04	0.04	0.04	0.04	0.04	0.04	0.04
SMOTE	0.05	0.05	0.05	0.05	0.07	0.06	0.05	0.05	0.05	0.05	0.04	0.06	0.05	0.04	0.04
PS-	0.04	0.04	0.03	0.03	0.04	0.03	0.04	0.04	0.03	0.04	0.03	0.03	0.04	0.04	0.03
RF	0.06	0.06	0.06	0.06	0.06	0.06	0.06	0.06	0.06	0.06	0.06	0.05	0.05	0.05	0.06
SMA	0.03	0.03	0.04	0.03	0.03	0.03	0.03	0.03	0.03	0.04	0.04	0.04	0.04	0.04	0.04

Table S11: Brier scores for predicting the minority group of the biased covariate under each bias mitigation method. The proportion of observations removed ranges from 15% to 95%. The reference value for the brier score of the minority group of the original cohort for the Colon Cancer N0147 trial data was 0.03. MB-CBII denotes marginal bias, conditional bias I, and conditional bias II, respectively.

D.2 Danish colorectal cancer data

DCCG	Approach	Marginal bias			Conditional bias I			Conditional bias II		
		**AUC**	**OR_Z (SE)**	**I_Z**	**AUC**	**OR_Z (SE)**	**I_Z**	**AUC**	**OR_Z (SE)**	**I_Z**
Missing prop = 15%	Biased data	0.71	1.50(-)	-	0.71	1.41(-)	-	0.71	1.50(-)	-
	RUS	0.71	1.59(0.11)	0.83	0.71	1.40(0.10)	0.84	0.71	1.53(0.10)	0.93
	ROS	0.71	1.49(0.10)	0.97	0.71	1.41(0.10)	0.87	0.72	1.49(0.09)	0.98
	SMOTE	0.54	1.14(0.05)	0.00	0.67	3.67(0.06)	0.00	0.67	3.48(0.06)	0.00
	PS-matching	0.70	1.38(0.11)	0.84	0.70	1.40(0.10)	0.85	0.71	1.42(0.10)	0.88
	RF ensemble	0.69	-	-	0.69	-	-	0.66	-	-
	SMA	0.72	1.48(0.10)	0.97	0.72	1.44(0.10)	0.94	0.72	1.49(0.09)	0.99
Missing prop = 30%	Biased data	0.71	1.51(-)	-	0.71	1.41(-)	-	0.71	1.51(0.10)	-
	RUS	0.71	1.59(0.11)	0.83	0.71	1.40(0.10)	0.85	0.71	1.53(0.10)	0.94
	ROS	0.71	1.49(0.10)	0.97	0.71	1.41(0.10)	0.87	0.72	1.49(0.09)	0.98
	SMOTE	0.53	2.20(0.02)	0.00	0.67	3.67(0.06)	0.00	0.67	3.48(0.06)	0.00
	PS-matching	0.70	1.38(0.11)	0.84	0.70	1.40(0.10)	0.85	0.71	1.42(0.10)	0.88
	RF ensemble	0.69	-	-	0.69	-	-	0.66	-	-
	SMA	0.72	1.48(0.10)	0.97	0.72	1.44(0.10)	0.94	0.71	1.49(0.09)	0.99
Missing prop = 50%	Biased data	0.71	1.55(-)	-	0.72	1.50(0.11)	-	0.71	1.45(-)	-
	RUS	0.71	1.53(0.13)	0.86	0.72	1.56(0.11)	0.87	0.72	1.53(0.10)	0.93
	ROS	0.70	1.56(0.10)	0.87	0.72	1.53(0.10)	0.91	0.71	1.52(0.12)	0.91
	SMOTE	0.54	0.26(0.03)	0.00	0.59	0.41(0.06)	0.00	0.66	4.10(0.06)	0.00
	PS-matching	0.70	1.58(0.15)	0.80	0.71	1.49(0.12)	0.90	0.71	1.40(0.14)	0.85
	RF ensemble	0.69	-	-	0.68	-	-	0.65	-	-
	SMA	0.70	1.53(0.10)	0.90	0.70	1.54(0.10)	0.91	0.71	1.49(0.10)	0.95
Missing prop = 80%	Biased data	0.71	1.44(-)	-	0.71	1.53(-)	-	0.71	1.49(-)	-
	RUS	0.71	1.64(0.20)	0.72	0.71	1.70(0.14)	0.72	0.72	1.46(0.10)	0.95
	ROS	0.71	1.39(0.10)	0.82	0.72	1.55(0.11)	0.89	0.71	1.48(0.10)	0.98
	SMOTE	0.53	0.27(0.04)	0.00	0.60	0.47(0.08)	0.00	0.66	3.58(0.06)	0.00
	PS-matching	0.69	1.75(0.29)	0.63	0.70	1.70(0.21)	0.69	0.71	1.45(0.10)	0.93
	RF ensemble	0.68	-	-	0.68	-	-	0.66	-	-
	SMA	0.68	1.62(0.15)	0.81	0.68	1.61(0.10)	0.83	0.71	1.51(0.09)	0.91

Table S12: Danish Colorectal Cancer data. Estimates of mean AUC, Odds Ratio of Z [OR_Z(SE)], and interval overlaps (I_Z) of the biased covariate Z for each bias mitigating approach. For the Original data: AUC = 0.72; OR_Z(SE) = 1.49 (0.09); I_Z =1.00.

		Marginal bias			Conditional bias I			Conditional bias II		
		SPD	EOD	AOD	SPD	EOD	AOD	SPD	EOD	AOD
15%	Original	0.03468991	0.04381693	0.03846661	0.03468991	0.04381693	0.03846661	0.03468991	0.04381693	0.03846661
	Biased	0.03767215	0.04733844	0.04167199	0.03373957	0.04275114	0.03746850	0.03512007	0.04442725	0.03897132
	RUS	0.03867908	0.04887051	0.04289622	0.03344996	0.04229745	0.03711099	0.03767345	0.04816579	0.04201511
	ROS	0.03687484	0.04608132	0.04068442	0.03161668	0.03988646	0.03503866	0.03715609	0.04770401	0.04152074
	PS-matching	0.03677701	0.04627522	0.04070730	0.03334411	0.04236484	0.03707683	0.03491752	0.04433677	0.03881514
	SMOTE	0.17227266	0.19202503	0.18044605	0.05116598	0.06426332	0.05658557	0.04165021	0.05138717	0.04567930
	SMA	0.03992645	0.05056718	0.04432951	0.03992645	0.05056718	0.04432951	0.03538592	0.04494336	0.03934072
		SPD	EOD	AOD	SPD	EOD	AOD	SPD	EOD	AOD
30%	Original	0.03468991	0.04381693	0.03846661	0.03468991	0.04381693	0.03846661	0.03468991	0.04381693	0.03846661
	Biased	0.03203714	0.04030872	0.03545986	0.03481905	0.04384714	0.03855481	0.03498329	0.04417260	0.03878577
	RUS	0.03128850	0.03966488	0.03475459	0.03366507	0.04236279	0.03726413	0.03919482	0.04985141	0.04360445
	ROS	0.03177978	0.04011701	0.03522967	0.03444180	0.04336943	0.03813599	0.03743532	0.04720489	0.04147790
	PS-matching	0.02206079	0.02755445	0.02433403	0.03435630	0.04328908	0.03805262	0.03724712	0.04733350	0.04142080
	SMOTE	-0.10321145	-0.11510958	0.10813481	0.07077568	0.08715794	0.07755454	0.04244586	0.05431576	0.04735754
	SMA	0.03726544	0.04709545	0.04133303	0.03726544	0.04709545	0.04133303	0.03723513	0.04728223	0.04139255
		SPD	EOD	AOD	SPD	EOD	AOD	SPD	EOD	AOD
50%	Original	0.03468991	0.04381693	0.03846661	0.03468991	0.04381693	0.03846661	0.03468991	0.04381693	0.03846661
	Biased	0.03845515	0.04887808	0.04276809	0.03940540	0.04999730	0.04378825	0.03415492	0.04310667	0.03785909
	RUS	0.03657366	0.04634529	0.04061710	0.04181364	0.05390640	0.04681754	0.03315650	0.04162578	0.03666103
	ROS	0.04088668	0.05180323	0.04540387	0.04175157	0.05306473	0.04643288	0.03171702	0.04060627	0.03539533
	PS-matching	0.02616516	0.03313336	0.02904855	0.04096670	0.05189513	0.04548881	0.03283309	0.04152021	0.03642776
	SMOTE	-0.10770606	-0.12434163	0.11458975	0.01041892	0.01291407	0.01145140	0.03622737	0.04423755	0.03954193
	SMA	0.03883736	0.04988791	0.04341000	0.03883736	0.04988791	0.04341000	0.03365625	0.04270241	0.03739949
		SPD	EOD	AOD	SPD	EOD	AOD	SPD	EOD	AOD
80%	Original	0.03468991	0.04381693	0.03846661	0.03468991	0.04381693	0.03846661	0.03468991	0.04381693	0.03846661
	Biased	0.04247477	0.05394048	0.04721921	0.04048667	0.05144015	0.04501915	0.03341556	0.04196684	0.03695402
	RUS	0.03157968	0.04077064	0.03538283	0.03924626	0.04972774	0.04358343	0.03386296	0.04250255	0.03743796
	ROS	0.04372780	0.05599645	0.04880448	0.03897504	0.04882099	0.04304923	0.03400812	0.04291132	0.03769220
	PS-matching	0.01331130	0.01633023	0.01456051	0.05925348	0.07499481	0.06576714	0.03225525	0.04056767	0.03569487
	SMOTE	-0.10802334	-0.11898754	0.11256025	0.04887621	0.05969831	0.05335432	0.04344487	0.05363942	0.04766330
	SMA	0.05119520	0.06634382	0.05746359	0.05119520	0.06634382	0.05746359	0.03214566	0.04082427	0.03573681

Table S13: Measure of fairness. Estimates of the statistical parity difference (SPD), equal opportunity difference (EOD), and average odds difference (AOD) on the Danish Colorectal Cancer data.

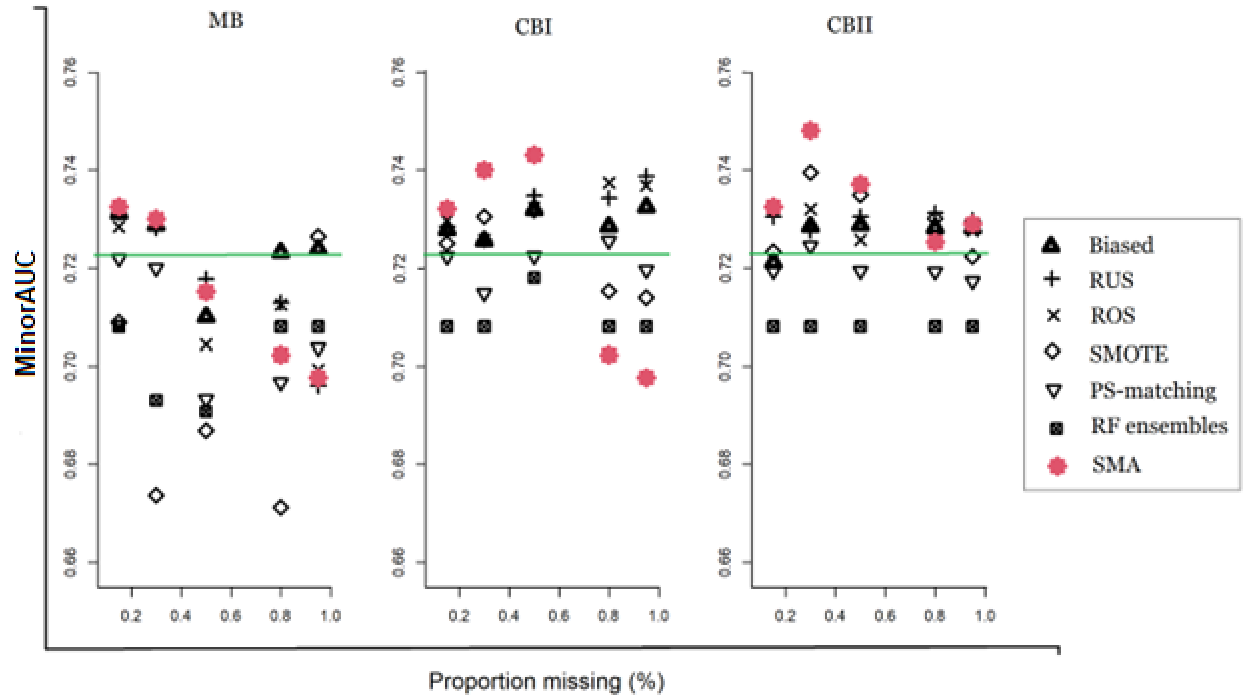

Figure S2. Danish Colorectal Cancer data. Plots of AUC estimates of the minority group of the biased covariate variable. MB denotes marginal bias, CBI for conditional bias I, and CBII for conditional bias II. Horizontal line is the ground truth estimate.

Missing	***15%***			***30%***			***50%***			***80%***			***95%***		
**Approach**	**MB**	**CB1**	**CBII**	**MB**	**MB**	**CBI**	**MB**	**CBI**	**CBII**	**MB**	**CBI**	**CBII**	**MB**	**CBI**	**CBII**
Biased data	0.07	0.07	0.07	0.07	0.07	0.07	0.07	0.07	0.06	0.06	0.06	0.06	0.07	0.07	0.07
RUS	0.07	0.07	0.07	0.07	0.07	0.07	0.07	0.07	0.07	0.07	0.07	0.07	0.07	0.07	0.07
ROS	0.07	0.07	0.07	0.07	0.07	0.07	0.07	0.07	0.07	0.07	0.07	0.07	0.07	0.07	0.07
SMOTE	0.08	0.08	0.07	0.08	0.09	0.09	0.08	0.08	0.08	0.09	0.07	0.08	0.07	0.07	0.09
PS-	0.07	0.07	0.07	0.05	0.05	0.05	0.07	0.07	0.07	0.07	0.07	0.07	0.07	0.07	0.07
RF	0.09	0.09	0.09	0.09	0.09	0.09	0.09	0.09	0.09	0.09	0.09	0.09	0.09	0.08	0.09
SMA	0.07	0.07	0.07	0.07	0.07	0.06	0.06	0.06	0.06	0.07	0.07	0.07	0.07	0.07	0.07

Table S14: Brier scores for predicting the minority group of the biased covariate under each bias mitigation method. The proportion of observations removed ranges from 15% to 95%. The reference value for the brier score of the minority group of the original cohort for Danish Colorectal Cancer data was 0.07. MB-CBII denotes marginal bias, conditional bias I, and conditional bias II, respectively.

D.3 Breast Cancer data

Breast Cancer	Approach	Marginal bias			Conditional bias I			Conditional bias II		
		**AUC**	**OR_Z (SE)**	**I_Z**	**AUC**	**OR_Z (SE)**	**I_Z**	**AUC**	**OR_Z (SE)**	**I_Z**
Missing prop = 15%	Biased data	0.81	0.81(-)	-	0.83	0.82 (-)	-	0.85	0.77(-)	-
	RUS	0.86	0.83(0.61)	0.83	0.82	1.11(0.52)	0.74	0.86	0.86(0.49)	0.89
	ROS	0.81	0.53(0.45)	0.77	0.84	0.82(0.46)	0.89	0.84	0.75(0.47)	0.99
	SMOTE	0.78	0.62(0.19)	0.68	0.78	0.52(0.13)	0.59	0.80	0.36(0.36)	0.52
	PS-matching	0.85	0.87(0.52)	0.87	0.85	0.81(0.89)	0.68	0.88	0.78(0.70)	0.77
	RF ensemble	0.82	-	-	0.82	-	-	0.83	-	-
	SMA	0.83	0.75(0.44)	0.96	0.84	0.75(0.51)	0.93	0.85	0.76(0.47)	0.99
Missing prop = 30%	Biased data	0.81	0.73(-)	-	0.83	0.82 (-)	-	0.84	0.83(-)	-
	RUS	0.82	0.84(0.61)	0.82	0.83	1.11(0.52)	0.74	0.86	0.83(0.51)	0.87
	ROS	0.81	0.53(0.45)	0.77	0.84	0.89(0.46)	0.89	0.84	0.75(0.47)	0.98
	SMOTE	0.78	0.52(0.13)	0.59	0.83	0.77(0.31)	0.82	0.81	0.36(0.36)	0.52
	PS-matching	0.86	0.67(0.52)	0.86	0.85	0.81(0.89)	0.68	0.88	0.78(0.70)	0.77
	RF ensemble	0.82	-	-	0.82	-	-	0.83	-	-
	SMA	0.83	0.75(0.45)	0.95	0.84	0.75(0.51)	0.93	0.85	0.76(0.47)	0.99
Missing prop = 50%	Biased data	0.80	0.58(-)	-	0.83	0.80 (-)	-	0.84	0.68(-)	-
	RUS	0.83	0.69(0.72)	0.82	0.82	1.11(0.52)	0.74	0.83	0.53 (0.52)	0.84
	ROS	0.79	0.67(0.46)	0.91	0.82	0.78(0.46)	0.98	0.85	0.67(0.49)	0.94
	SMOTE	0.78	0.52(0.13)	0.59	0.82	0.47(0.38)	0.66	0.81	0.36(0.36)	0.52
	PS-matching	0.87	0.41(1.44)	0.61	0.81	0.40(1.04)	0.76	0.89	0.42(0.72)	0.91
	RF ensemble	0.83	-	-	0.81	-	-	0.80	-	-
	SMA	0.83	0.75(0.47)	0.94	0.82	0.77(0.51)	0.98	0.85	0.70(0.49)	0.95
Missing prop = 80%	Biased data	0.82	1.48(-)	-	0.81	0.82 (-)	-	0.84	0.77(-)	-
	RUS	0.80	0.83(0.61)	0.82	0.79	0.75(0.52)	0.80	0.84	0.70(0.55)	0.94
	ROS	0.86	1.58(0.58)	0.59	0.82	0.81(0.43)	1.00	0.86	0.76(0.50)	0.95
	SMOTE	0.78	2.77(0.13)	0.24	0.79	0.77(0.31)	0.82	0.80	0.60(0.35)	0.78
	PS-matching	0.86	1.05(1.46)	0.54	0.82	0.08(0.89)	0.66	0.87	0.82(0.70)	0.68
	RF ensemble	0.80	-	-	0.79	-	-	0.77	-	-
	SMA	0.86	0.95(0.54)	0.65	0.81	0.75(0.51)	0.95	0.87	0.75(0.51)	0.94

Table S15: Breast Cancer data. Estimates of mean AUC, Odds Ratio of Z [OR_Z(SE)], and confidence interval overlaps (I_Z) of the biased covariate Z for each bias mitigating approach. For the original data: AUC = 0.84; OR_Z(SE) = 0.76 (0.46); I_Z =1.00.

		Marginal bias			Conditional bias I			Conditional bias II		
		SPD	EOD	AOD	SPD	EOD	AOD	SPD	EOD	AOD
15%	Original	-0.04843516	-0.05803158	0.05093023	-0.04843516	-0.05803158	0.05093023	-0.04843516	-0.05803158	0.05093023
	Biased	-0.07819081	-0.09010742	0.08128913	-0.06545581	-0.07753269	0.06859580	-0.03465149	-0.04224985	0.03662706
	RUS	-0.12121716	-0.12780123	0.12292902	-0.05770824	-0.06944574	0.06075999	-0.04565305	-0.05474187	0.04801614
	ROS	-0.11683552	-0.12741212	0.11958544	-0.07594085	-0.08867105	0.07925070	-0.04922790	-0.05894357	0.05175397
	PS-matching	-0.23071950	-0.24536109	0.23474593	-0.09304108	-0.10899817	0.09742928	-0.07263817	-0.08916006	0.07718169
	SMOTE	-0.07528525	-0.08502369	0.07781724	-0.10525716	-0.11531926	0.10787331	0.07940146	0.07911960	0.07932817
	SMA	-0.05224629	-0.06207177	0.05480091	-0.05224629	-0.06207177	0.05480091	-0.03596337	-0.04402445	0.03805925
		SPD	EOD	AOD	SPD	EOD	AOD	SPD	EOD	AOD
30%	Original	-0.04843516	-0.05803158	0.05093023	-0.04843516	-0.05803158	0.05093023	-0.04843516	-0.05803158	0.05093023
	Biased	-0.10026835	-0.11657459	0.10450797	-0.04297181	-0.05070168	0.04498158	-0.07586980	-0.08798721	0.07902033
	RUS	-0.07528107	-0.08822990	0.07864777	-0.08769870	-0.09711186	0.09014612	-0.06596229	-0.07732908	0.06891765
	ROS	-0.12281645	-0.14568468	0.12876219	-0.02887323	-0.03477914	0.03040877	-0.07953287	-0.09209151	0.08279811
	PS-matching	-0.34030215	-0.36761277	0.34781257	-0.14585204	-0.16110126	0.15004558	-0.13021999	-0.14707246	0.13485442
	SMOTE	-0.11134809	-0.12739281	0.11551971	-0.35858289	-0.38468826	0.36537029	-0.19846967	-0.20572591	0.20035629
	SMA	-0.21124876	-0.22964741	0.21603241	-0.21124876	-0.22964741	0.21603241	-0.10403607	-0.11621859	0.10720353
		SPD	EOD	AOD	SPD	EOD	AOD	SPD	EOD	AOD
50%	Original	-0.04843516	-0.05803158	0.05093023	-0.048435164	-0.058031579	0.050930232	-0.04843516	-0.05803158	0.05093023
	Biased	-0.02430764	-0.02825330	0.02533351	0.002928406	0.003599259	0.003102828	-0.06694935	-0.07822892	0.06988204
	RUS	0.04723793	0.04789455	0.04740865	0.017476690	0.021570967	0.018541202	-0.05959601	-0.06826943	0.06185110
	ROS	-0.03311307	-0.03832999	0.03446947	-0.030395213	-0.035537398	0.031732181	-0.11362300	-0.12639483	0.11694368
	PS-matching	-0.02998319	-0.04404133	0.03384918	-0.152208783	-0.184100211	0.160978925	-0.16306363	-0.18107604	0.16801704
	SMOTE	-0.05402994	-0.06255927	0.05624756	-0.124482415	-0.163294073	0.134573446	-0.29961613	-0.31886018	0.30461958
	SMA	-0.03691470	-0.04057452	0.03786626	-0.036914705	-0.040574521	0.037866257	-0.07102527	-0.08056575	0.07350579
		SPD	EOD	AOD	SPD	EOD	AOD	SPD	EOD	AOD
80%	Original	-0.04843516	-0.05803158	0.05093023	-0.048435164	-0.05803158	0.05093023	-0.04843516	-0.05803158	0.05093023
	Biased	-0.06735357	-0.07465980	0.06925319	-0.050101338	-0.05913962	0.05245129	-0.07408776	-0.08668486	0.07736301
	RUS	-0.12122316	-0.14977156	0.12864575	0.008897549	0.01024975	0.00924912	-0.12564678	-0.14446418	0.13053930
	ROS	-0.03893138	-0.04656619	0.04091643	-0.100314735	-0.11559274	0.10428702	-0.08189089	-0.09590478	0.08553450
	PS-matching	0.24724283	0.29330562	0.25991010	-0.375004880	-0.37887048	0.37606792	-0.11436521	-0.13173354	0.11914150
	SMOTE	-0.06828342	-0.08546733	0.07275124	-0.354386306	-0.32044730	0.34556216	-0.19241255	-0.18105866	0.18946054
	SMA	-0.05486941	-0.06432072	0.05732675	-0.054869410	-0.06432072	0.05732675	-0.07344795	-0.08422348	0.07624959

Table S16: Measure of fairness. Estimates of the statistical parity difference (SPD), equal opportunity difference (EOD), and average odds difference (AOD) on the Breast Cancer data.

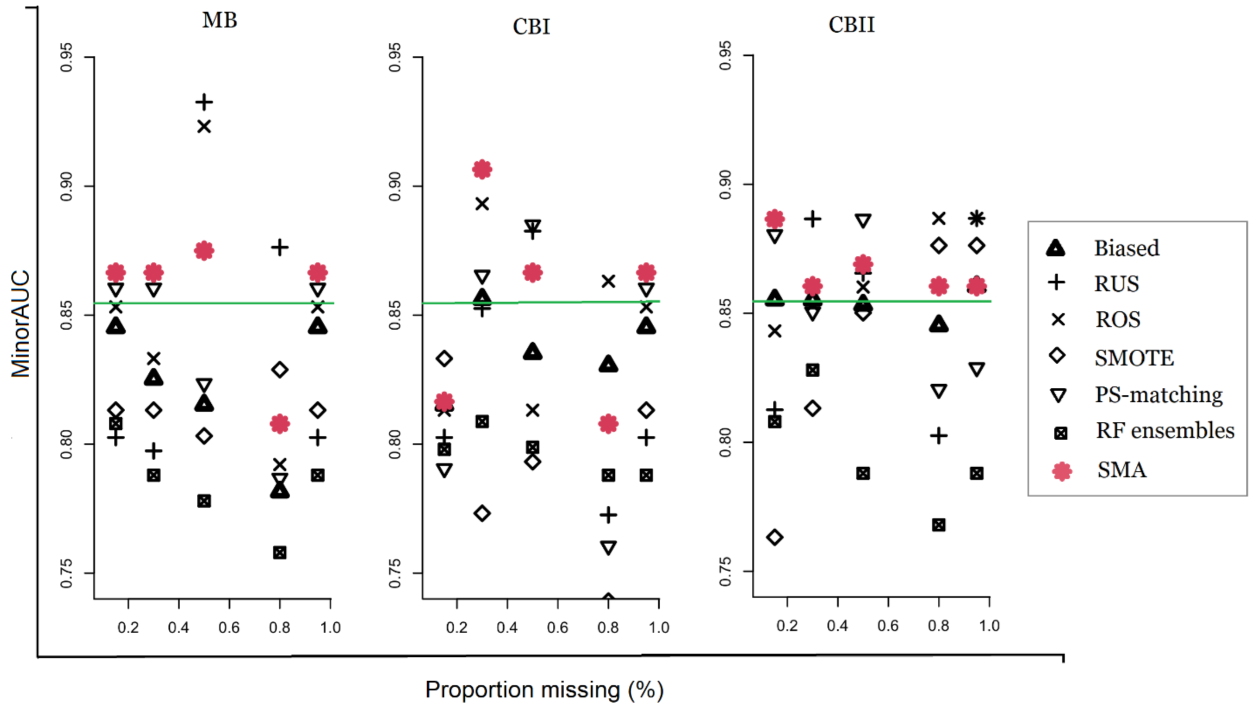

Figure S3. Breast Cancer data. Plot of AUC estimates of the minor categories of the biased covariate. MB denotes marginal bias, CBI for conditional bias I, and CBII for conditional bias II. Horizontal line is the ground truth estimate.

Missing	***15%***			***30%***			***50%***			***80%***			***95%***		
**Approach**	**MB**	**CB1**	**CBII**	**MB**	**MB**	**CBI**	**MB**	**CBI**	**CBII**	**MB**	**CBI**	**CBII**	**MB**	**CBI**	**CBII**
Biased data	0.07	0.07	0.07	0.07	0.07	0.07	0.07	0.07	0.07	0.07	0.07	0.07	0.07	0.07	0.07
RUS	0.07	0.07	0.07	0.07	0.07	0.07	0.07	0.07	0.07	0.07	0.07	0.07	0.07	0.07	0.07
ROS	0.07	0.07	0.07	0.07	0.07	0.07	0.07	0.07	0.07	0.07	0.07	0.07	0.07	0.07	0.07
SMOTE	0.09	0.09	0.09	0.09	0.12	0.1	0.09	0.09	0.09	0.09	0.07	0.1	0.09	0.07	0.07
PS-	0.07	0.07	0.05	0.05	0.07	0.05	0.07	0.07	0.05	0.07	0.05	0.05	0.07	0.07	0.05
RF	0.1	0.1	0.1	0.1	0.1	0.1	0.1	0.1	0.1	0.1	0.1	0.09	0.09	0.09	0.1
SMA	0.05	0.05	0.07	0.05	0.05	0.05	0.05	0.05	0.05	0.07	0.07	0.07	0.07	0.07	0.07

Table S17: Brier scores for predicting the minority group of the biased covariate under each bias mitigation method. The proportion of observations removed ranges from 15% to 95%. The reference value for the brier score of the minority group of the original cohort for Breast Cancer data was 0.05. MB-CBII denotes marginal bias, conditional bias I, and conditional bias II, respectively.

D.4 Cardiovascular Health data

CCHS data Missing Prop	Approach	Marginal bias			Conditional bias I			Conditional bias II		
		AUC	OR_Z (SE)	I_Z	AUC	OR_Z (SE)	I_Z	AUC	OR_Z (SE)	I_Z
15%	Biased data	0.70	1.57(-)	-	0.70	1.57(-)	-	0.70	1.58(-)	-
	RUS	0.70	1.58(0.03)	0.94	0.70	1.58(0.03)	0.94	0.70	1.57(0.02)	0.97
	ROS	0.70	1.57(0.02)	0.97	0.70	1.57(0.02)	0.95	0.70	1.58(0.02)	0.97
	SMOTE	0.70	1.52(0.04)	0.77	0.70	1.56(0.04)	0.76	0.70	1.57(0.04)	0.78
	PS-matching	0.69	1.50(0.03)	0.56	0.69	1.50(0.03)	0.53	0.69	1.54(0.03)	0.73
	RF ensemble	0.70	-	-	0.70	-	-	0.70	-	-
	SMA‡	0.70	1.57(0.03)	0.97	0.70	1.57(0.03)	0.94	0.70	1.58(0.03)	0.97
30%	Biased data	0.70	1.59(-)	-	0.70	1.61(-)	-	0.70	1.59(-)	-
	RUS	0.70	1.59(0.03)	0.89	0.70	1.61(0.03)	0.78	0.70	1.58(0.03)	0.94
	ROS	0.70	1.60(0.02)	0.86	0.70	1.60 (0.02)	0.84	0.70	1.60 (0.02)	0.84
	SMOTE	0.69	1.81(0.02)	0.00	0.69	1.88(0.02)	0.00	0.70	1.83(0.04)	0.00
	PS-matching	0.69	1.48(0.03)	0.44	0.69	1.51(0.03)	0.54	0.69	1.56(0.03)	0.92
	RF ensemble	0.70	-	-	0.68	-	-	0.69	-	-
	SMA‡	0.70	1.59(0.02)	0.91	0.70	1.59(0.02)	0.94	0.70	1.80(0.02)	0.95
50%	Biased data	0.70	1.61(-)	-	0.69	1.63(-)	-	0.69	1.61(-)	-
	RUS	0.70	1.59(0.03)	0.83	0.70	1.61(0.03)	0.81	0.70	1.60(0.03)	0.82
	ROS	0.70	1.59(0.02)	0.87	0.70	1.61(0.02)	0.77	0.70	1.61(0.02)	0.74
	SMOTE	0.68	1.85(0.02)	0.66	0.69	1.89(0.02)	0.00	0.69	1.85(0.02)	0.00
	PS-matching	0.69	1.44(0.04)	0.29	0.69	1.49(0.04)	0.60	0.69	1.57(0.03)	0.89
	RF ensemble	0.69	-	-	0.69	-	-	0.68	-	-
	SMA‡	0.70	1.59(0.02)	0.89	0.70	1.59(0.02)	0.90	0.70	1.59(0.02)	0.90
80%	Biased data	0.69	1.66(-)	-	0.69	1.66(-)	-	0.69	1.61(-)	-
	RUS	0.70	1.60(0.05)	0.75	0.70	1.60(0.05)	0.75	0.70	1.61(0.03)	0.77
	ROS	0.70	1.65(0.02)	0.59	0.70	1.65(0.02)	0.49	0.70	1.62(0.02)	0.67
	SMOTE	0.68	1.82(0.03)	0.00	0.69	1.81(0.03)	0.00	0.69	1.79(0.02)	0.00
	PS-matching	0.70	1.50(0.07)	0.67	0.69	1.50 (0.07)	0.67	0.69	1.49(0.03)	0.49
	RF ensemble	0.69	-	-	0.69	-	-	0.68	-	-
	SMA‡	0.70	1.56(0.02)	0.78	0.70	1.57(0.02)	0.82	0.70	1.56(0.02)	0.80

Table S18: Estimates of mean AUC, Odds Ratio of Z [OR_Z(SE)], and interval overlaps (I_Z) of the biased covariate Z for each bias mitigating approach on the Cardiovascular Health (CCHS) data. For the Original data: AUC = 0.70; OR_Z(SE) = 1.58 (0.02); I_Z =1.00. Note. Synthetic Minor Augmentation (SMA). ‡ indicates that the estimates are averaged from m=100 synthetic copies.

Missing Prop	Approach	SPD			EOD			AOD		
		MB	CB1	CBII	MB	CB1	CBII	MB	CB1	CBII
15%	Biased	0.095	0.097	0.097	0.091	0.093	0.093	0.097	0.099	0.099
	RUS	0.097	0.098	0.096	0.093	0.094	0.092	0.099	0.099	0.097
	ROS	0.096	0.098	0.096	0.092	0.094	0.092	0.098	0.099	0.098
	PS-matching	0.091	0.090	0.094	0.089	0.088	0.092	0.092	0.091	0.095
	SMOTE	0.098	0.092	0.108	0.095	0.089	0.104	0.100	0.093	0.110
	SMA‡	0.095	0.096	0.097	0.092	0.092	0.093	0.097	0.097	0.099
30%	Biased	0.099	0.095	0.098	0.096	0.092	0.094	0.101	0.097	0.100
	RUS	0.099	0.093	0.097	0.095	0.089	0.094	0.101	0.094	0.099
	ROS	0.097	0.096	0.098	0.093	0.092	0.094	0.099	0.097	0.099
	PS-matching	0.088	0.087	0.096	0.085	0.084	0.094	0.088	0.087	0.097
	SMOTE	0.115	0.097	0.107	0.110	0.094	0.103	0.117	0.099	0.108
	SMA‡	0.102	0.093	0.098	0.094	0.090	0.094	0.104	0.095	0.100
50%	Biased	0.098	0.104	0.098	0.095	0.100	0.095	0.100	0.106	0.100
	RUS	0.096	0.101	0.096	0.092	0.097	0.093	0.098	0.103	0.098
	ROS	0.100	0.099	0.095	0.096	0.095	0.091	0.102	0.101	0.096
	PS-matching	0.088	0.089	0.092	0.085	0.086	0.090	0.088	0.089	0.093
	SMOTE	0.098	0.097	0.097	0.094	0.093	0.094	0.099	0.098	0.098
	SMA‡	0.103	0.101	0.096	0.099	0.094	0.093	0.105	0.103	0.098
80%	Biased	0.111	0.103	0.104	0.107	0.099	0.100	0.112	0.105	0.105
	RUS	0.106	0.098	0.105	0.102	0.094	0.102	0.107	0.099	0.107
	ROS	0.113	0.095	0.100	0.109	0.092	0.096	0.115	0.097	0.101
	PS-matching	0.115	0.092	0.092	0.112	0.090	0.090	0.116	0.093	0.093
	SMOTE	0.094	0.100	0.104	0.091	0.097	0.101	0.095	0.102	0.106
	SMA‡	0.104	0.106	0.100	0.100	0.102	0.096	0.106	0.108	0.102

Table S19: Fairness metrics: the statistical parity difference (SPD), equal opportunity difference (EOD), and average odds difference (AOD) from the logistic regression model on the cardiovascular health data. The proportion of samples removed varied from 15% to 80%. Original cohort: SPD = 0.096, EOD = 0.092, and AOD = 0.098. ‡ indicates that the estimates are averaged from m=100 synthetic copies.

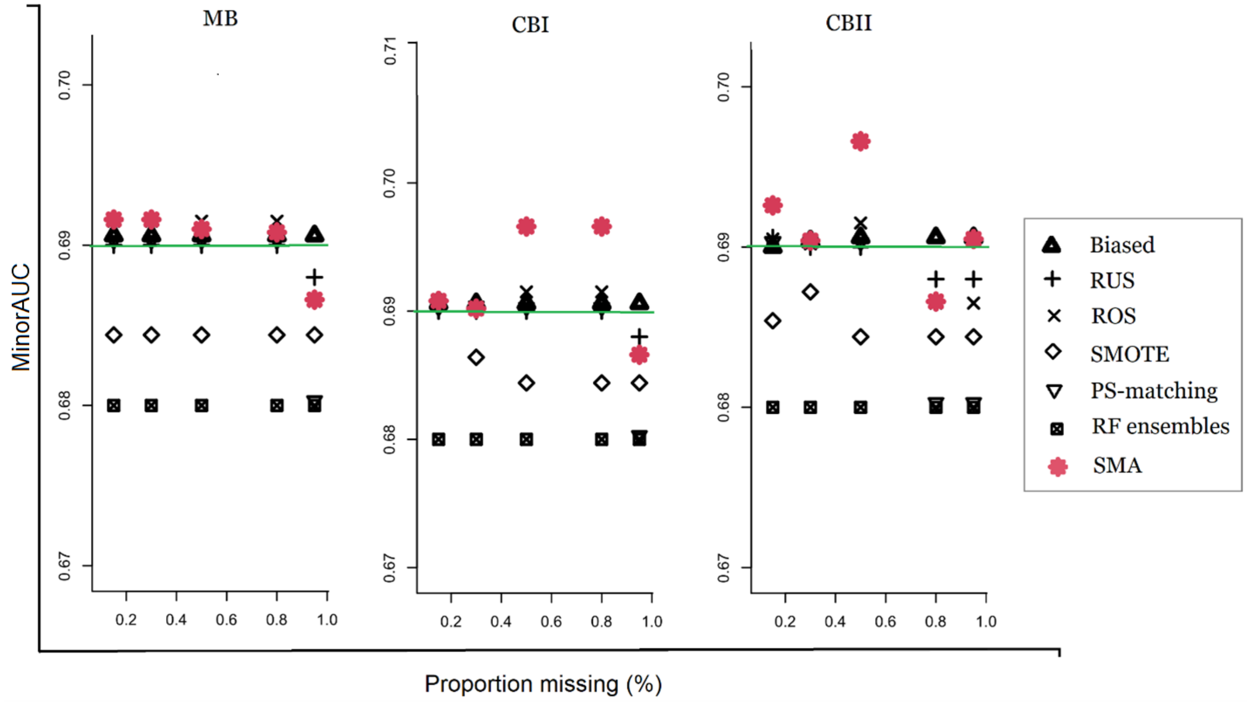

Figure S4: Cardiovascular Health data used for the stratified model. Plots of AUC estimates of the minor categories of the biased covariate. MB denotes marginal bias, CBI for conditional bias I, and CBII for conditional bias II. The horizontal line is the ground truth estimate (AUC =0.69).

Missing	***15%***			***30%***			***50%***			***80%***			***95%***		
**Approach**	**MB**	**CB1**	**CBII**	**MB**	**MB**	**CBI**	**MB**	**CBI**	**CBII**	**MB**	**CBI**	**CBII**	**MB**	**CBI**	**CBII**
Biased data	0.04	0.05	0.05	0.04	0.05	0.05	0.04	0.05	0.05	0.04	0.05	0.05	0.04	0.05	0.05
RUS	0.04	0.04	0.04	0.04	0.04	0.04	0.04	0.04	0.04	0.04	0.04	0.04	0.04	0.04	0.04
ROS	0.04	0.04	0.04	0.04	0.04	0.04	0.04	0.04	0.04	0.04	0.04	0.04	0.04	0.04	0.04
SMOTE	0.05	0.05	0.04	0.05	0.05	0.05	0.05	0.05	0.05	0.07	0.04	0.04	0.04	0.04	0.08
PS-	0.04	0.04	0.04	0.04	0.04	0.04	0.04	0.04	0.04	0.04	0.04	0.04	0.04	0.04	0.04
RF	0.05	0.05	0.05	0.05	0.05	0.05	0.06	0.05	0.05	0.06	0.05	0.06	0.06	0.05	0.06
SMA	0.04	0.04	0.04	0.04	0.04	0.04	0.04	0.04	0.04	0.04	0.04	0.04	0.04	0.04	0.04

Table S20: Brier scores for predicting the minority group of the biased covariate under each bias mitigation method. The proportion of observations removed ranges from 15% to 95%. The reference value for the brier score of the minority group of the original cohort for Cardiovascular health data was 0.04, respectively. MB-CBII denotes marginal bias, conditional bias I, and conditional bias II, respectively.

D.5 Summaries of all real data results

We present plots of the relative performance of the metrics over the four real datasets for (I) low to medium bias, and (II) high bias. The plots include summaries for overall model effect estimates (Odds ratios), AUCs, Brier scores, Fairness estimates, and AUC of the minority group.

D.5.1 Overall AUCs

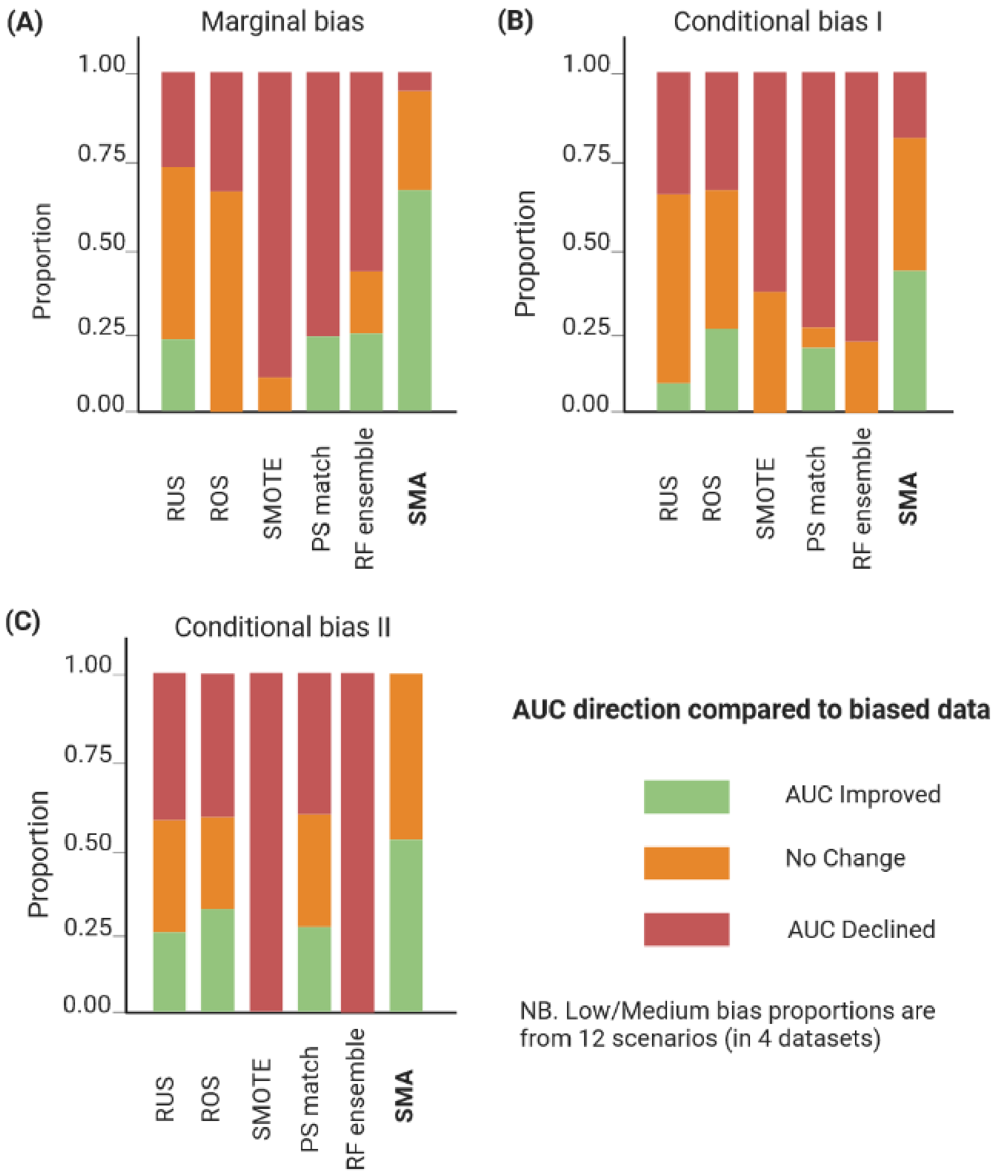

Figure S5: Low to Medium bias setting. Summaries of the model AUC for **low to medium bias** over the four real datasets. The relative performance of each bias mitigating approach compared to biased data results is shown. The model AUC is considered improved if the difference between the model AUC and the ground truth estimate is less than the difference between the biased data AUC and the ground truth. Summaries are over bias proportions: 15%, 30%, and 50%.

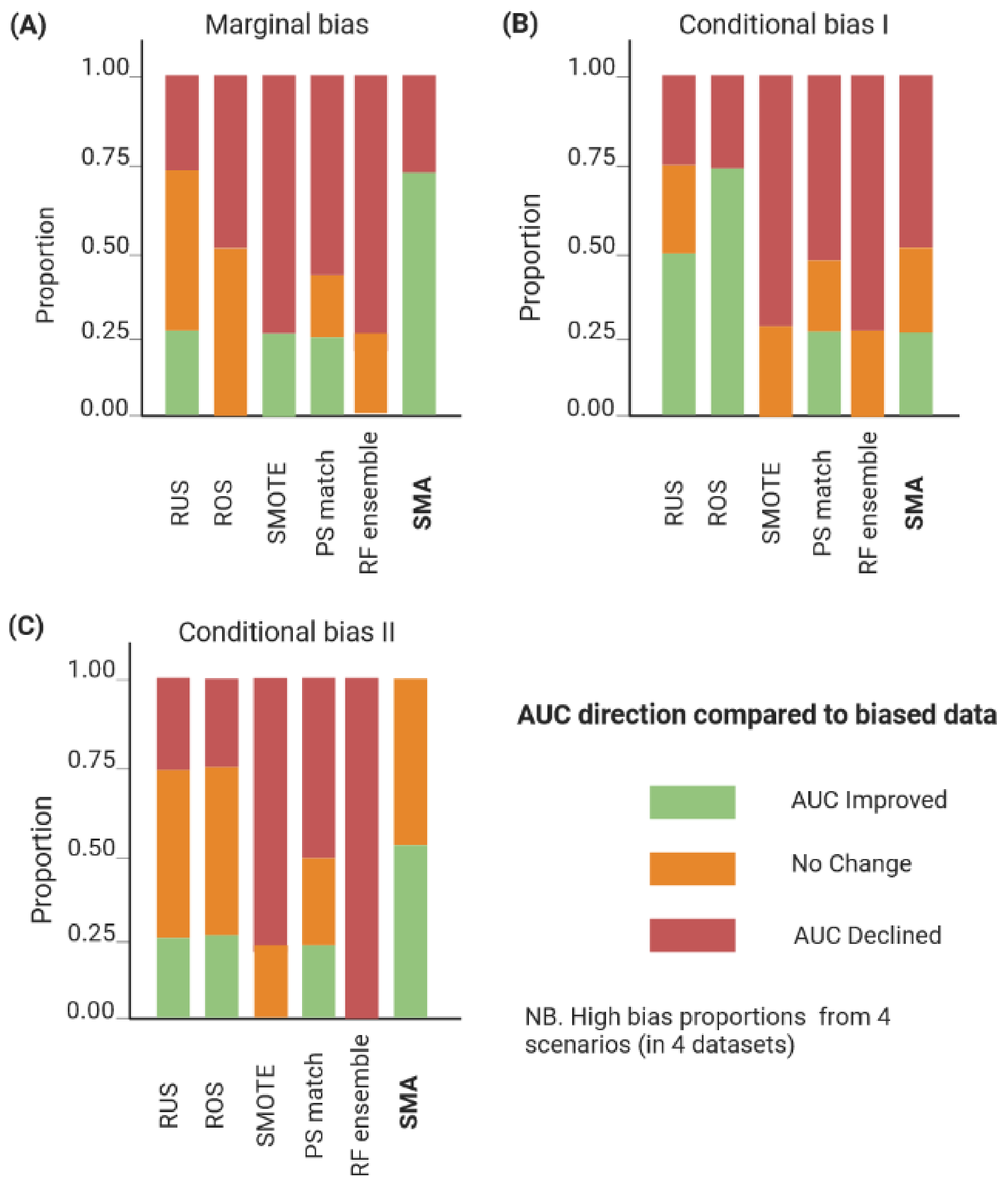

Figure S6: High bias setting. Summaries of the model AUC for **high bias** over the four real datasets. The relative performance of each bias mitigating approach compared to biased data results is shown. The model AUC is considered improved if the difference between the model AUC and the ground truth estimate is less than the difference between the biased data AUC and the ground truth. Summaries are over bias proportion of 80%.

D.5.2 Odds Ratio

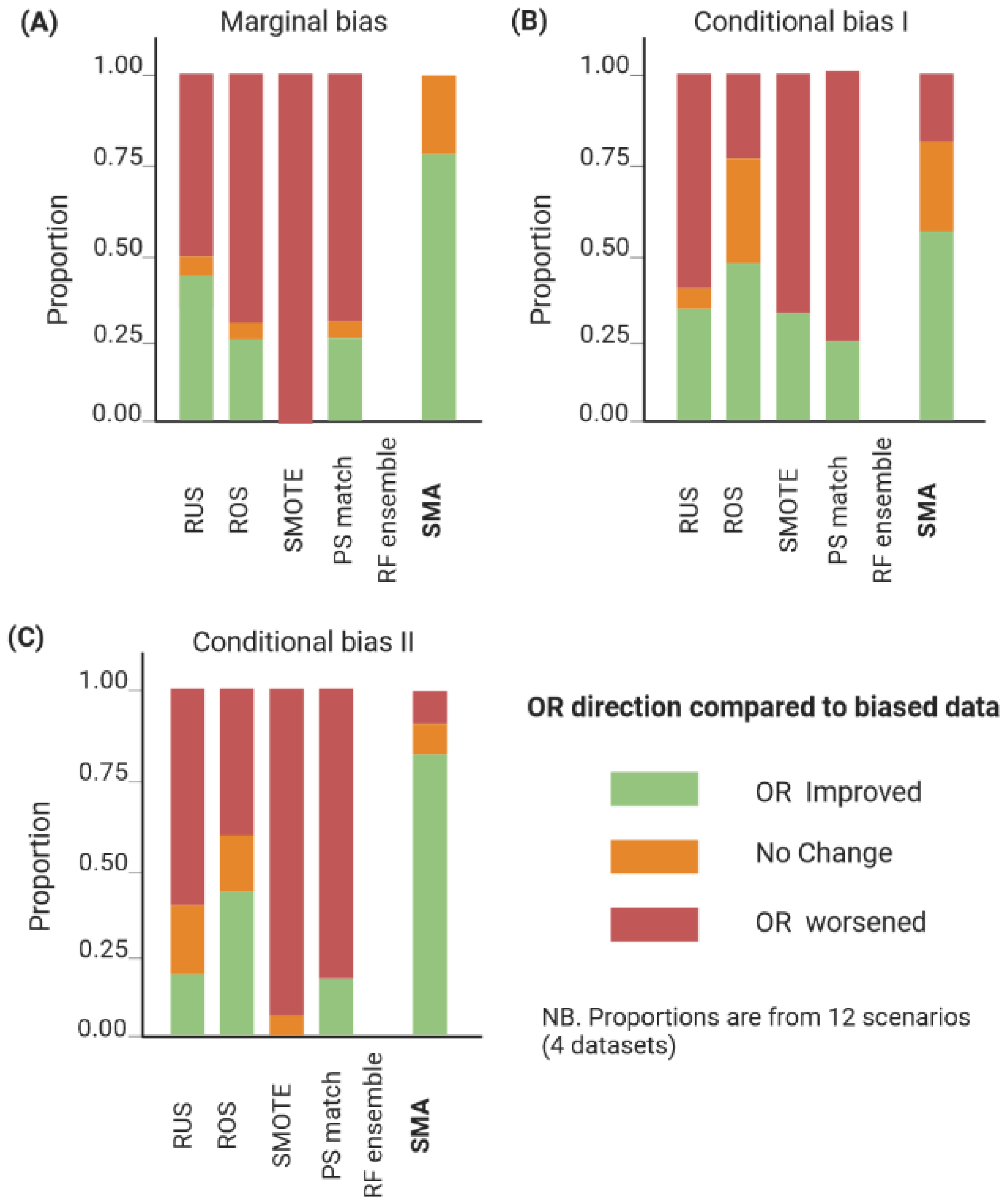

Figure S7: Low to Medium bias setting. Summaries of the Odds Ratio (OR) of the biasing covariate for **low to medium bias** over the four real datasets. The relative performance of each bias mitigating approach compared to the biased data results is shown. The OR direction is considered improved if the difference between the model OR and the ground truth estimate is less than the difference between the biased data OR and the ground truth. Summaries are over bias proportions: 15%, 30%, and 50%.

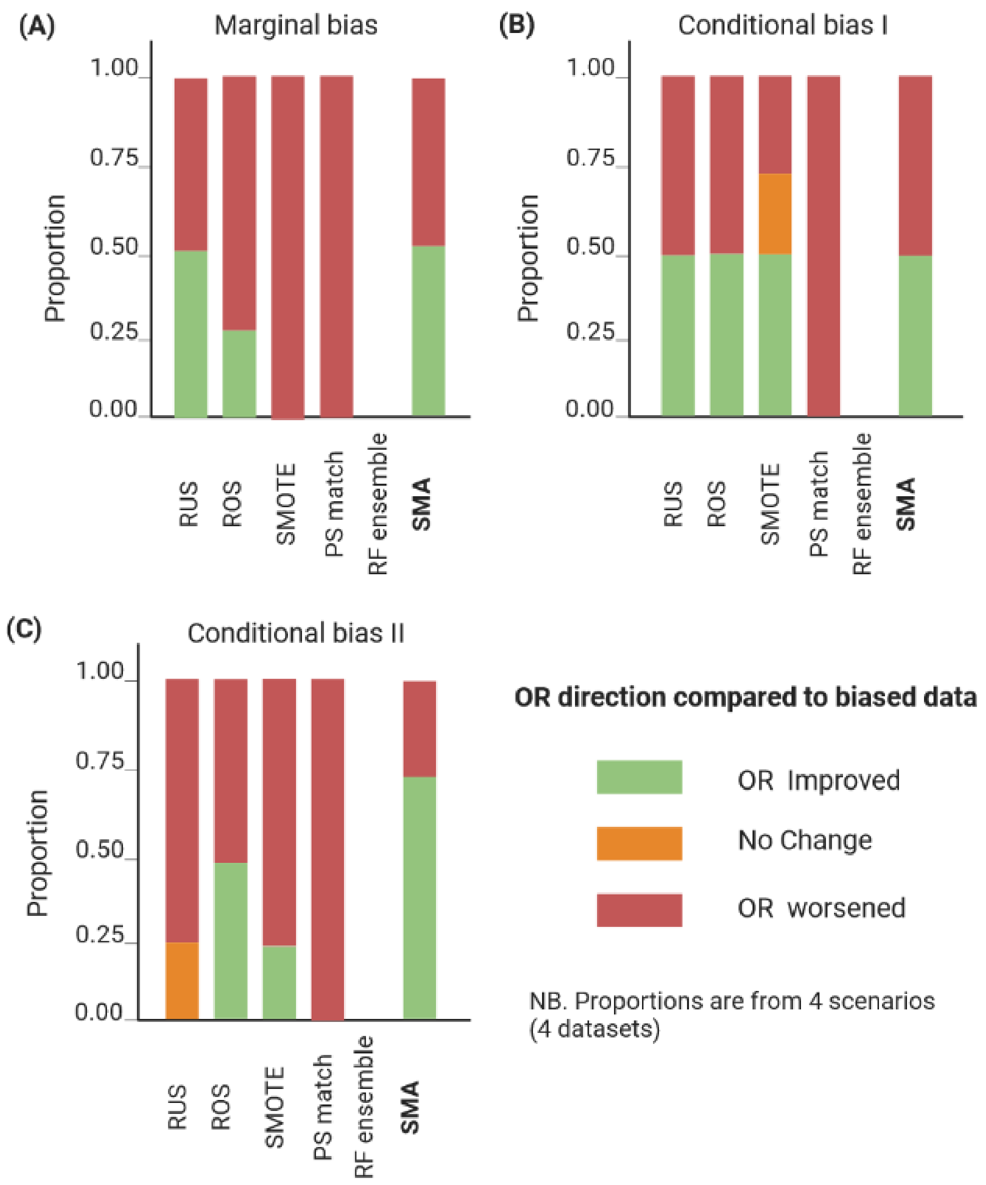

Figure S8: High bias setting. Summaries of the Odds Ratio (OR) of the biasing covariate for **high bias** over the four real datasets. The relative performance of each bias mitigating approach compared to the biased data results is shown. The OR direction is considered improved if the difference between the model OR and the ground truth estimate is less than the difference between the biased data OR and the ground truth. Summaries are over bias proportion of 80%.

D.5.3 Brier Score

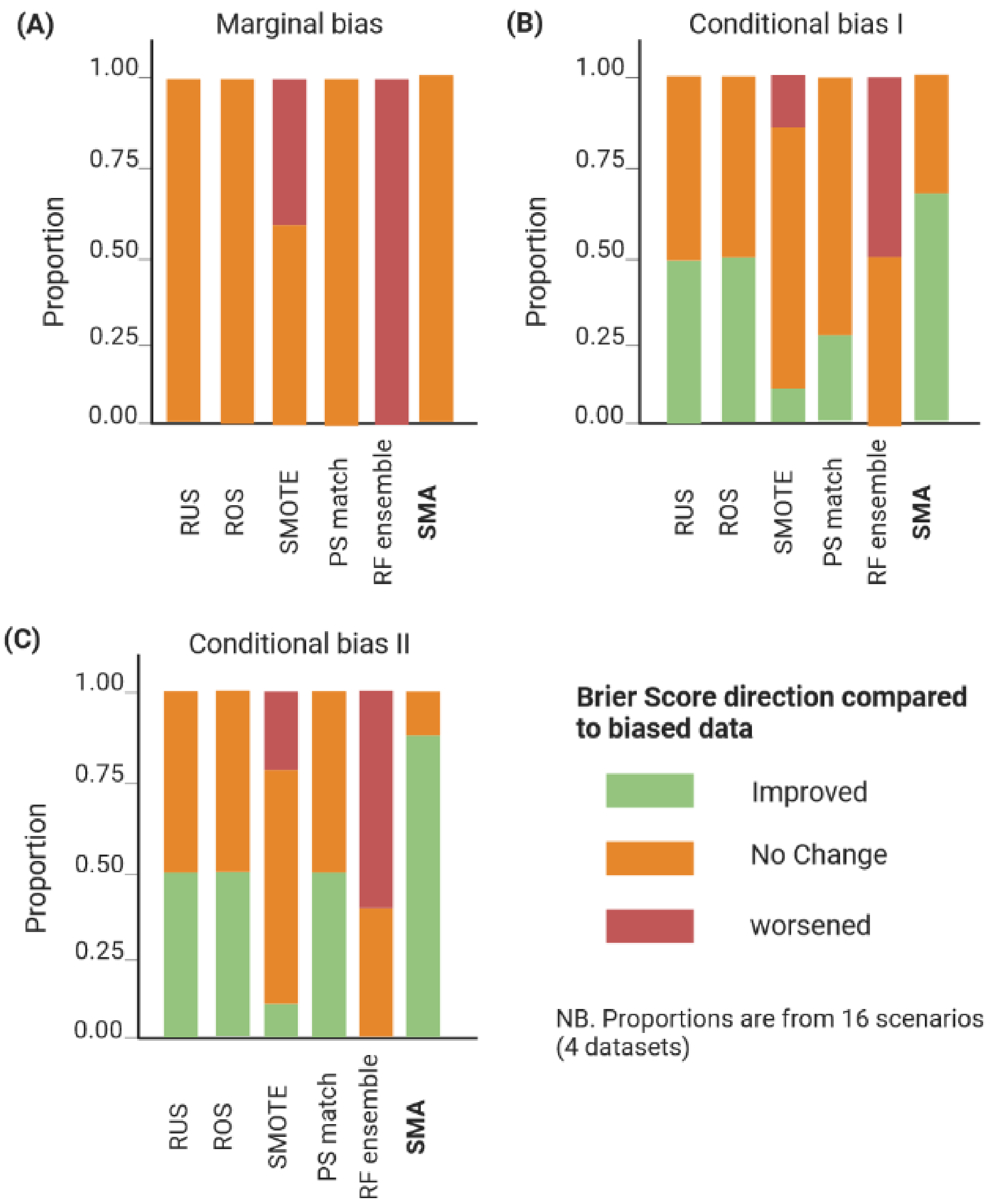

Figure S9: Overall summaries of the model Brier scores over the four real datasets. The relative performance of each bias mitigating approach compared to biased data results is shown. The Brier score is considered improved if the difference between the model brier score and the ground truth estimate is less than the difference between the biased data Brier score and the ground truth. Summaries are over all bias proportions: 15%, 30%, 50%, and 80%.

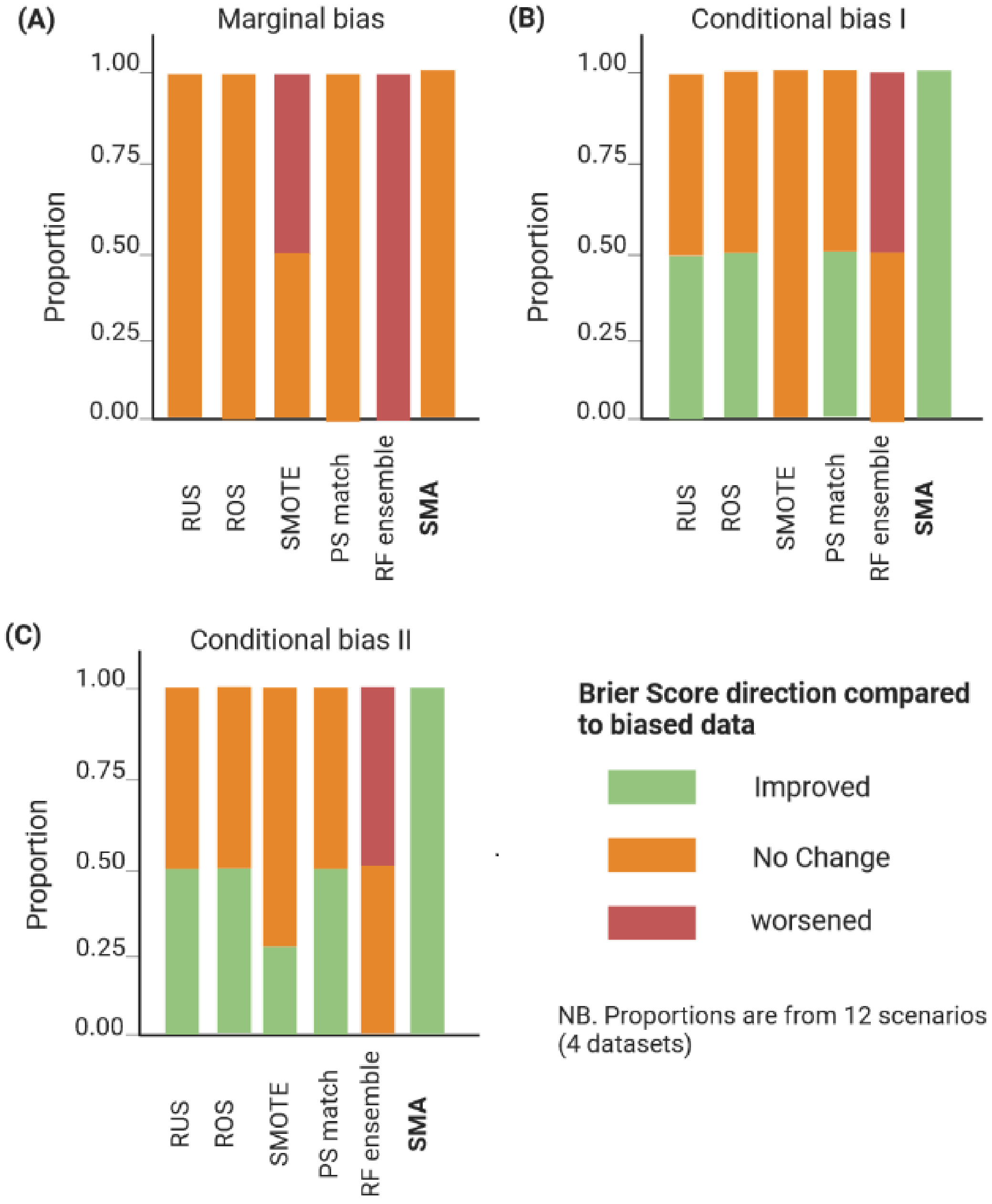

Figure S10: Low to Medium bias setting. Summaries of the model Brier scores over the four real datasets for **low to medium bias**. The relative performance of each bias mitigating approach compared to biased data results is shown. The Brier score is considered improved if the difference between the model brier score and the ground truth estimate is less than the difference between the biased data Brier score and the ground truth. Summaries are over all bias proportions: 15%, 30%, and 50%.

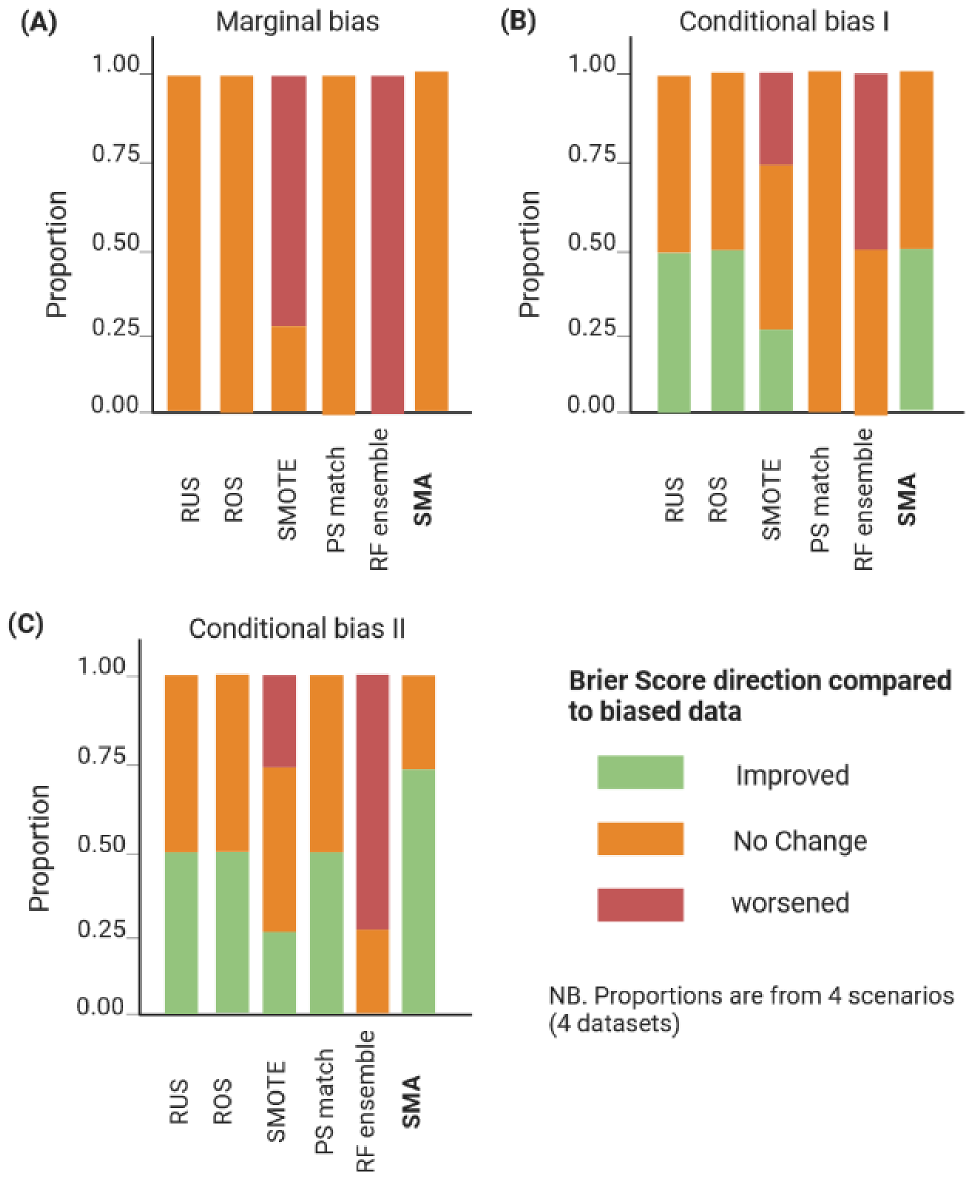

Figure S11: High bias setting. Summaries of the model Brier scores over the four real datasets for **high bias**. The relative performance of each bias mitigating approach compared to biased data results is shown. The Brier score is considered improved if the difference between the model brier score and the ground truth estimate is less than the difference between the biased data Brier score and the ground truth. Summaries are over bias proportion of 80%.

D.5.4 Fairness Metrics

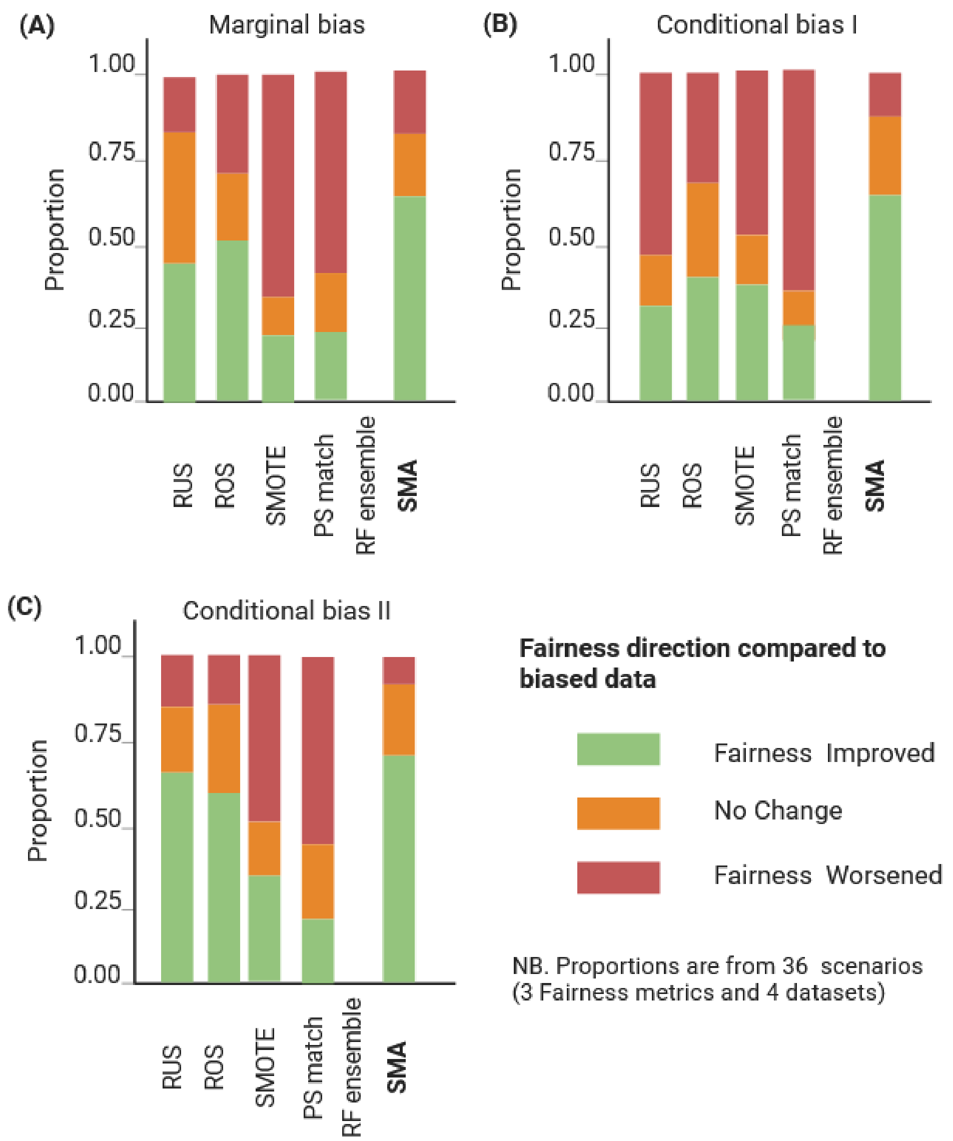

Figure S12: Low to Medium bias setting. Summaries of the Fairness metrics: the statistical parity difference (SPD), equal opportunity difference (EOD), and average odds difference (AOD) over the four real datasets for **low to medium bias**. The relative performance of each bias approach compared to biased data results is shown. Fairness is considered improved if the difference between the model Fairness and the ground truth estimate is less than the difference between the biased data Fairness and the ground truth. Summaries are over all bias proportions: 15%, 30%, and 50%.

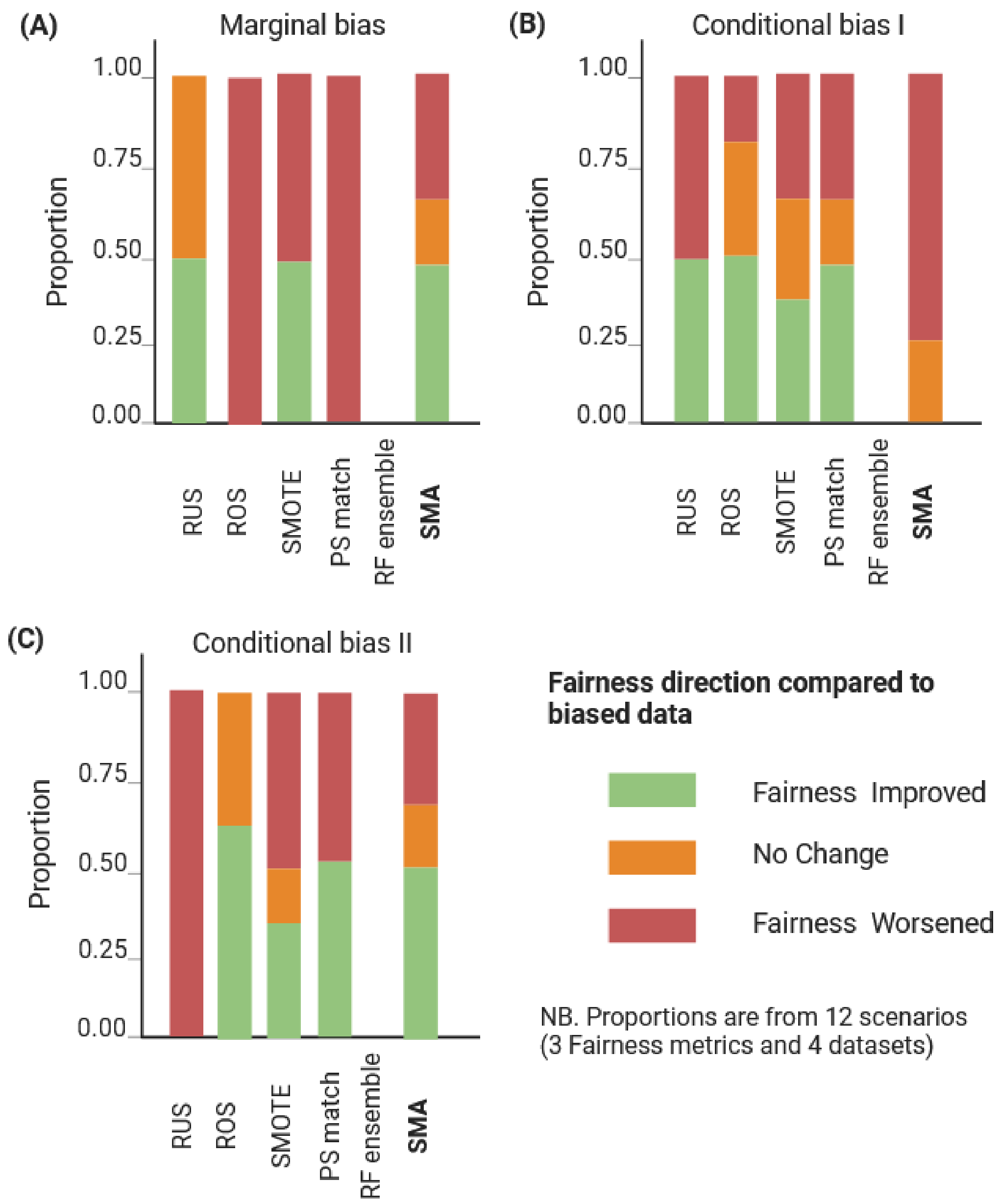

Figure S13: High bias setting. Summaries of the Fairness metrics: the statistical parity difference (SPD), equal opportunity difference (EOD), and average odds difference (AOD) over the four real datasets for **high bias**. The relative performance of each bias approach compared to biased data results is shown. Fairness is considered improved if the difference between the model Fairness and the ground truth estimate is less than the difference between the biased data Fairness and the ground truth. Summaries are over bias proportion of 80%.

D.5.5 AUC for the minority group of the biasing covariate

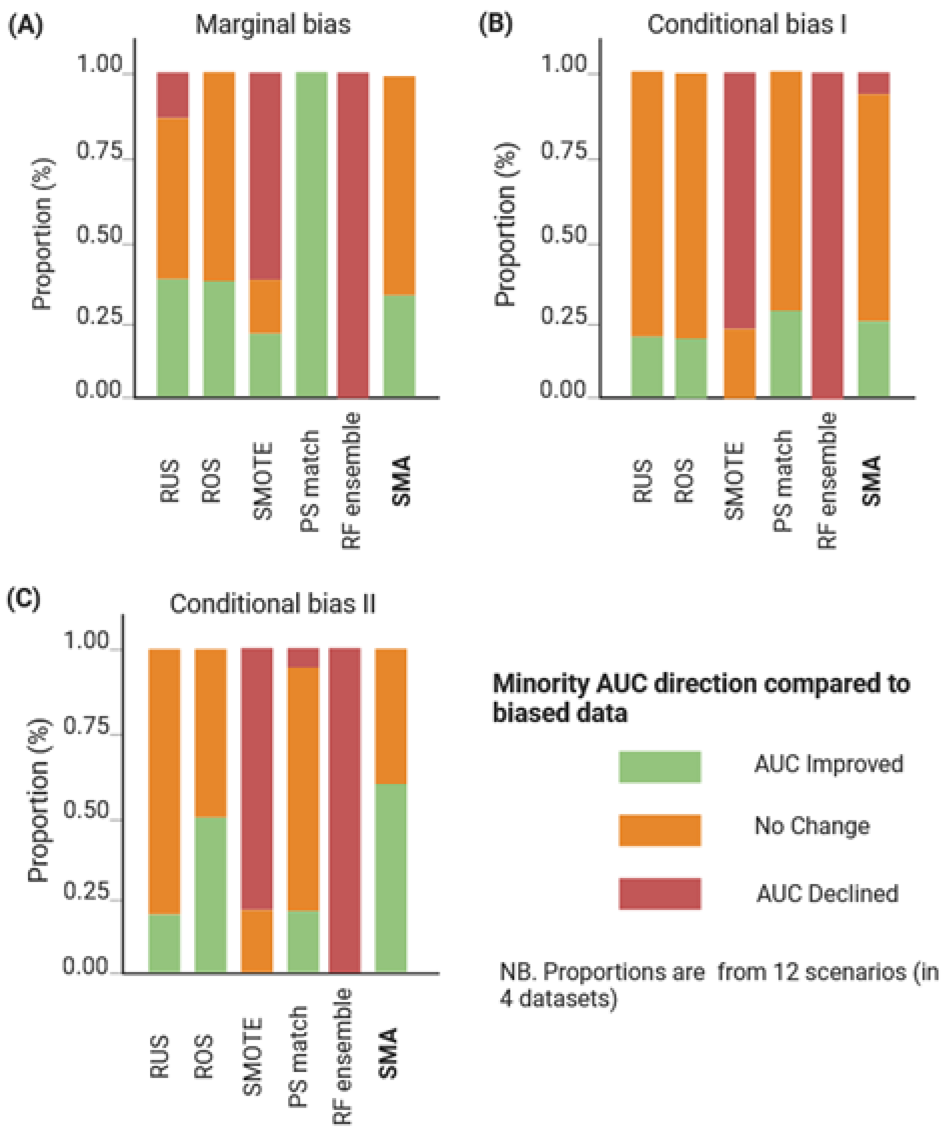

Figure S14: Low to Medium bias setting. Summaries of the model minority AUC for low to medium bias over the four real datasets for **low to medium bias**. The relative performance of each bias mitigating approach compared to biased data results is shown. The minority AUC is considered improved if the difference between the model minority AUC and the ground truth estimate is less than the difference between the biased data minority AUC and the ground truth. Summaries are over all bias proportions: 15%, 30%, and 50%.

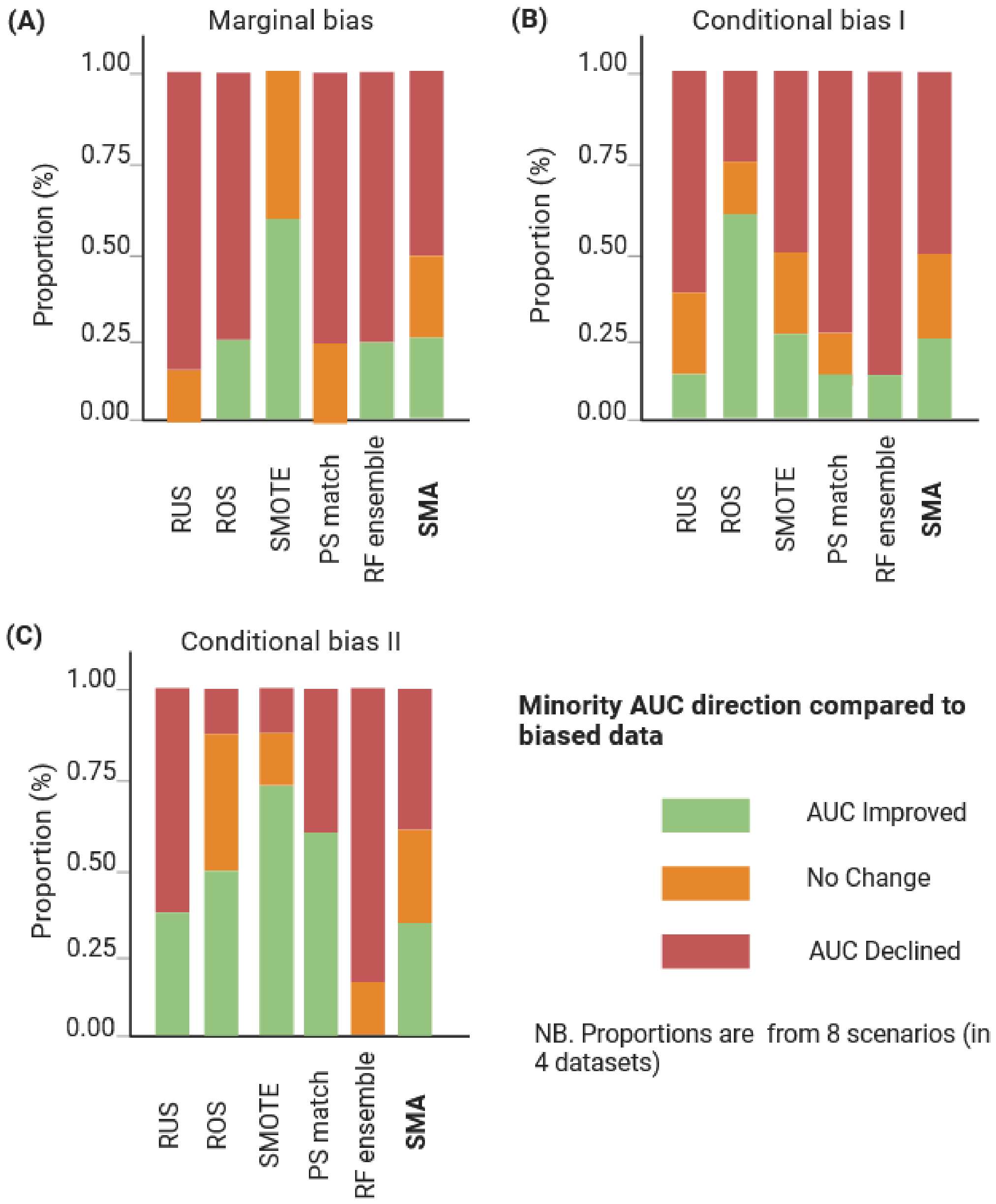

Figure S15: High bias setting. Summaries of the model minority AUC for high bias over the four real datasets for **high bias**. The relative performance of each bias mitigating approach compared to biased data results is shown. The minority AUC is considered improved if the difference between the model minority AUC and the ground truth estimate is less than the difference between the biased data minority AUC and the ground truth. Summaries are over bias proportion of 80% and 95%.

References

1. Chawla, N.V. (2009). Data mining for imbalanced datasets: An overview. Data Min. Knowl. Discov. Handb., 875–886.

2. Sun, Y., Wong, A.K.C., and Kamel, M.S. (2009). Classification of imbalanced data: a review. Int. J. Pattern Recognit. Artif. Intell. *23*, 687–719. 10.1142/S0218001409007326.

3. Glauner, P., Valtchev, P., and State, R. (2018). Impact of Biases in Big Data. Preprint at arXiv, 10.48550/arXiv.1803.00897 10.48550/arXiv.1803.00897.

4. Blagus, R., and Lusa, L. (2013). SMOTE for high-dimensional class-imbalanced data. BMC Bioinformatics *14*, 106. 10.1186/1471-2105-14-106.

5. Kotsiantis, S., Kanellopoulos, D., and Pintelas, P. (2006). Handling imbalanced datasets: A review. 13.

6. Mohammed, R., Rawashdeh, J., and Abdullah, M. (2020). Machine Learning with Oversampling and Undersampling Techniques: Overview Study and Experimental Results. In 2020 11th International Conference on Information and Communication Systems (ICICS), pp. 243–248. 10.1109/ICICS49469.2020.239556.

7. Megahed, F.M., Chen, Y.-J., Megahed, A., Ong, Y., Altman, N., and Krzywinski, M. (2021). The class imbalance problem. Nat. Methods *18*, 1270–1272. 10.1038/s41592-021-01302-4.

8. Chawla, N.V., Bowyer, K.W., Hall, L.O., and Kegelmeyer, W.P. (2002). SMOTE: Synthetic Minority Over-sampling Technique. J. Artif. Intell. Res. *16*, 321–357. 10.1613/jair.953.

9. Fernandez, A., Garcia, S., Herrera, F., and Chawla, N.V. (2018). SMOTE for Learning from Imbalanced Data: Progress and Challenges, Marking the 15-year Anniversary. J. Artif. Intell. Res. *61*, 863–905. 10.1613/jair.1.11192.

10. Raghuwanshi, B.S., and Shukla, S. (2020). SMOTE based class-specific extreme learning machine for imbalanced learning. Knowl.-Based Syst. *187*, 104814. 10.1016/j.knosys.2019.06.022.

11. Galar, M., Fernandez, A., Barrenechea, E., Bustince, H., and Herrera, F. (2012). A Review on Ensembles for the Class Imbalance Problem: Bagging-, Boosting-, and Hybrid-Based Approaches. IEEE Trans. Syst. Man Cybern. Part C Appl. Rev. *42*, 463–484. 10.1109/TSMCC.2011.2161285.

12. Lomax, S., and Vadera, S. (2013). A survey of cost-sensitive decision tree induction algorithms. ACM Comput. Surv. CSUR *45*, 1–35.

13. Khan, S.S., and Madden, M.G. (2010). A Survey of Recent Trends in One Class Classification. In Artificial Intelligence and Cognitive Science Lecture Notes in Computer Science., L. Coyle and J. Freyne, eds. (Springer), pp. 188–197. 10.1007/978-3-642-17080-5_21.

14. Dong, X., Yu, Z., Cao, W., Shi, Y., and Ma, Q. (2020). A survey on ensemble learning. Front. Comput. Sci. *14*, 241–258. 10.1007/s11704-019-8208-z.

15. Witten, I.H., Frank, E., Hall, M.A., Pal, C.J., and DATA, M. (2005). Practical machine learning tools and techniques. In Data Mining.

16. Chai, X., Deng, L., Yang, Q., and Ling, C.X. (2004). Test-cost sensitive naive Bayes classification. In Fourth IEEE International Conference on Data Mining (ICDM'04), pp. 51–58. 10.1109/ICDM.2004.10092.

17. Drummond, C., and Holte, R.C. Exploiting the Cost (In)sensitivity of Decision Tree Splitting Criteria. 8.

18. Fumera, G., and Roli, F. (2002). Cost-sensitive learning in support vector machines. VIII Convegno Assoc. Ital. L'Intelligenza Artif.

19. Cao, P., Zhao, D., and Zaiane, O. (2013). An optimized cost-sensitive SVM for imbalanced data learning. In Pacific-Asia conference on knowledge discovery and data mining (Springer), pp. 280–292.

20. Ling, C.X., and Sheng, V.S. (2007). Comparative study of cost-sensitive classifiers. Jisuanji XuebaoChinese J. Comput. *30*, 1203–1212.

21. Oza, P., and Patel, V.M. (2019). One-Class Convolutional Neural Network. IEEE Signal Process. Lett. *26*, 277–281. 10.1109/LSP.2018.2889273.

22. Binbusayyis, A., and Vaiyapuri, T. (2021). Unsupervised deep learning approach for network intrusion detection combining convolutional autoencoder and one-class SVM. Appl. Intell. *51*, 7094–7108. 10.1007/s10489-021-02205-9.

23. May, R.J., Maier, H.R., and Dandy, G.C. (2010). Data splitting for artificial neural networks using SOM-based stratified sampling. Neural Netw. *23*, 283–294. 10.1016/j.neunet.2009.11.009.

24. Mac Namee, B., Cunningham, P., Byrne, S., and Corrigan, O.I. (2002). The problem of bias in training data in regression problems in medical decision support. Artif. Intell. Med. *24*, 51–70. 10.1016/S0933-3657(01)00092-6.

25. Sun, L., and Bull, S.B. (2005). Reduction of selection bias in genomewide studies by resampling. Genet. Epidemiol. *28*, 352–367. 10.1002/gepi.20068.

26. Gray, L. (2016). The importance of post hoc approaches for overcoming non-response and attrition bias in population-sampled studies. Soc. Psychiatry Psychiatr. Epidemiol. *51*, 155–157. 10.1007/s00127-015-1153-8.

27. Zwitter, M., and Soklic, M. (2015). University Medical Centre, Institute of Oncology, Ljubljana, Yugoslavia. UCI Mach. Learn. Repos. Irvine CA USA Available Online Httpsarchive Ics Uci Edumldatasetsbreast Cancer Accessed 1 June 2021.

28. Tu, J.V., Chu, A., Donovan, L.R., Ko, D.T., Booth, G.L., Tu, K., Maclagan, L.C., Guo, H., Austin, P.C., Hogg, W., et al. (2015). The Cardiovascular Health in Ambulatory Care Research Team (CANHEART). Circ. Cardiovasc. Qual. Outcomes *8*, 204–212. 10.1161/CIRCOUTCOMES.114.001416.

29. Azizi, Z., Lindner, S., Shiba, Y., Raparelli, V., Norris, C.M., Kublickiene, K., Herrero, M.T., Kautzky-Willer, A., Klimek, P., Gisinger, T., et al. (2023). A comparison of synthetic data generation and federated analysis for enabling international evaluations of cardiovascular health. Sci. Rep. *13*, 11540. 10.1038/s41598-023-38457-3.
